# Regioselective Bromine/Magnesium Exchange for the Selective Functionalization of Polyhalogenated Arenes and Heterocycles

**DOI:** 10.1002/anie.202012496

**Published:** 2020-11-19

**Authors:** Alexandre Desaintjean, Tobias Haupt, Leonie J. Bole, Neil R. Judge, Eva Hevia, Paul Knochel

**Affiliations:** ^1^ Ludwig-Maximilians-Universität München Department Chemie Butenandtstrasse 5–13, Haus F 81377 München Germany; ^2^ Department für Chemie und Biochemie Universität Bern 3012 Bern Switzerland

**Keywords:** alkoxides, bromine/magnesium exchange, lewis bases, lithium, magnesiates

## Abstract

Using the bimetallic combination sBu_2_Mg⋅2 LiOR (R=2‐ethylhexyl) in toluene enables efficient and regioselective Br/Mg exchanges with various dibromo‐arenes and ‐heteroarenes under mild reaction conditions and provides bromo‐substituted magnesium reagents. Assessing the role of Lewis donor additives in these reactions revealed that N,N,N′,N′′,N′′‐pentamethyldiethylenetriamine (PMDTA) finely tunes the regioselectivity of the Br/Mg exchange on dibromo‐pyridines and quinolines. Combining spectroscopic with X‐ray crystallographic studies, light has been shed on the mixed Li/Mg constitution of the organometallic intermediates accomplishing these transformations. These systems reacted effectively with a broad range of electrophiles, including allyl bromides, ketones, aldehydes, and Weinreb amides in good yields.

## Graphical abstract

Using the bimetallic combination *s*Bu_2_Mg⋅2 LiOR (R=2‐ethylhexyl) in toluene enables a very fast regioselective Br/Mg exchange of dibromo(hetero)arenes in toluene. The regioselectivity of the exchange can be finely tuned by the coordination preference of lithium, which can be switched, in some cases, by the addition of Lewis donors such as PMDTA.

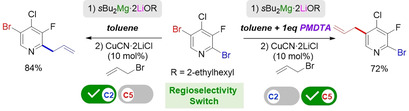

Functionalized halogenated arenes and heteroarenes are key tools for constructing pharmaceuticals, materials, and natural products.^[1]^ Several metal‐mediated approaches for the functionalization of polyhalogenated substrates have been developed to access these valuable molecules,^[2]^ including regioselective zinc insertion in the presence of LiCl on dihalogenated (hetero)arenes.^[3]^ Contrastingly, halogen/magnesium exchange, one of the most powerful methods to functionalize haloarenes, has shown limited success for this type of substrates in terms of versatility and regioselective tunability. Some exceptions include the use of *i*PrMgCl⋅LiCl (**1 a**, turbo‐Grignard reagent),^[4]^ which can promote selective Br/Mg exchanges in THF.^[5]^ Improved regioselectivities have also been achieved using bulkier variations of **1 a** containing mesityl or 2,4,6‐triisopropylphenyl substituents.^[6]^

Recently, it was shown by some of us that mixed‐metal compositions *s*BuMgOR⋅LiOR (**1 b**) and to a greater extent the stoichiometric variant *s*Bu_2_Mg⋅2 LiOR (R=2‐ethylhexyl, **1 c**) can promote Br/Mg exchanges in toluene or other non‐polar solvents with an excellent substrate scope when operated at room temperature.[^7^, ^8^] While formation of lithium magnesiates was postulated, the constitution of the organometallic intermediates involved has not yet been determined. Expanding further the synthetic utility of these alkyl/alkoxide s‐block metal combinations, herein, we report fast and highly regioselective Br/Mg exchanges on various dibromo‐arenes and ‐heterocycles using *s*Bu_2_Mg⋅2 LiOR (R=2‐ethylhexyl, **1 c**) in toluene. Interestingly, in some cases, the addition of Lewis donors such as PMDTA activates a regioselectivity switch, an operation that can be rationalized on consideration of the bimetallic constitution of the organometallic intermediates in these exchanges.

We commenced our studies assessing the regioselectivity of the Br/Mg exchange on 2,4‐dibromoanisole (**2 a**) with several mixed Li/Mg combinations (Table 1).

**Table 1:** Screening of the regioselective Br/Mg exchange on 2,4‐dibromoanisole (**2 a**). 
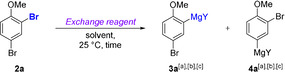

Entry	Exchange reagent^[d]^	Solvent	*t* [min]	Ratio **3 a**/**4 a**	Conv. [%]^[e]^
1	*i*PrMgCl⋅LiCl (**1 a**)	THF	120	85:15	87^[a]^
2	*s*BuMgOR⋅LiOR (**1 b**)	toluene	30	99:1	75^[b]^
3	*s*Bu_2_Mg⋅2 LiOR (**1 c**)	toluene	5	99:1	99^[c]^

[a] Y=Cl⋅LiCl. [b] Y=OR⋅LiOR. [c] Y=anisyl⋅2 LiOR. [d] R=2‐ethylhexyl, these reactions were carried out at 0.50 m using 1.2 equiv of alkylmagnesium species. Reagents are displayed according to their stoichiometry and not to their actual structure. [e] Conversion determined by GC‐analysis of reaction aliquots after aqueous quench.

First, we treated **2 a** with *i*PrMgCl⋅LiCl^[4a]^ (**1 a**) in THF at 25 °C for 2 h, giving an 85:15 ratio of the two regioisomeric magnesium species **3 a** and **4 a**, respectively, with a conversion of 87 % (Table 1, entry 1). The preferential formation of **3 a** may be explained by assuming a coordination of the exchange reagent to the neighboring methoxy substituent, reminiscent of the complex‐induced proximity effect (CIPE) in aromatic *ortho*‐lithiations.^[9]^ In an attempt to improve the regioselectivity by maximizing coordination effects between the substrate and the exchange reagent, ethereal THF was replaced by non‐polar toluene^[10]^ and *s*BuMgOR⋅LiOR^[7]^ (R=2‐ethylhexyl, **1 b**) was used as exchange reagent. Thus, treatment of **2 a** with **1 b** led after 30 min to the regioselective formation of 2‐anisylmagnesium species **3 a** (**3 a**/**4 a**=99:1) although with a lower conversion than **1 a** (75 %, Table 1, entry 2). However, using the more activated reagent *s*Bu_2_Mg⋅2 LiOR (**1 c**, 0.6 equiv), which was readily prepared by mixing *s*BuLi (2.0 equiv) with Mg(OR)_2_,^[7]^ magnesiation of **2 a** with **1 c** was complete after just 5 min affording **3 a** (**3 a**/**4 a**=99:1, Table 1, entry 3).

Different sets of substrates and electrophiles were investigated next. Thus, Cu‐catalyzed allylation^[11]^ of **3 a** furnished **5 a** in 72 % yield (Scheme 1). Similarly, electron‐rich 2‐bromoaryl ethers **2 b**–**2 d** underwent complete Br/Mg exchange at the C(2) position upon treatment with **1 c** (25 °C, 5 min). The corresponding diarylmagnesium (**3 b**–**3 d**) was smoothly thiomethylated with MeSO_2_SMe, acylated with *N*‐methoxy‐*N*‐methylacetamide or allylated with methallyl bromide, producing the bromoaryl ethers **5 b**–**5 d** in 64–87 % yield. Analogously, 3,5‐dibromo‐2‐methoxypyridine (**2 e**) was regioselectively converted into the *ortho*‐metalated compound **3 e**. After allylation with methallyl bromide, addition to a ketone, or transmetalation with ZnCl_2_ ^[12]^ followed by Pd‐catalyzed Negishi cross‐coupling with 4‐iodobenzonitrile,^[13]^ the functionalized bromopyridines **5 ea**–**5 ec** were isolated in 53–81 % yield. In addition, 2‐bromopyridines (**2 f**–**2 g**) led to the corresponding 2‐magnesiated pyridines (**3 f**–**3 g**), which gave after thiomethylation or acylation with a Weinreb amide^[14]^ the products **5 f**–**5 g** in 60–66 % yield. As an application, we have prepared the xanthone **5 ab**, a precursor of a type II dehydroquinase inhibitor (antibacterial properties).^[15]^ Thus, the selective magnesiation of **2 a** followed by a Cu‐catalyzed acylation with 2‐fluorobenzoyl chloride produced the benzophenone **5 aa** in 75 % yield. BBr_3_‐deprotection of the methoxy group and mild K_2_CO_3_‐mediated ring closure furnished the target xanthone in 96 % yield (Scheme 1).^[16]^

**Scheme 1:**
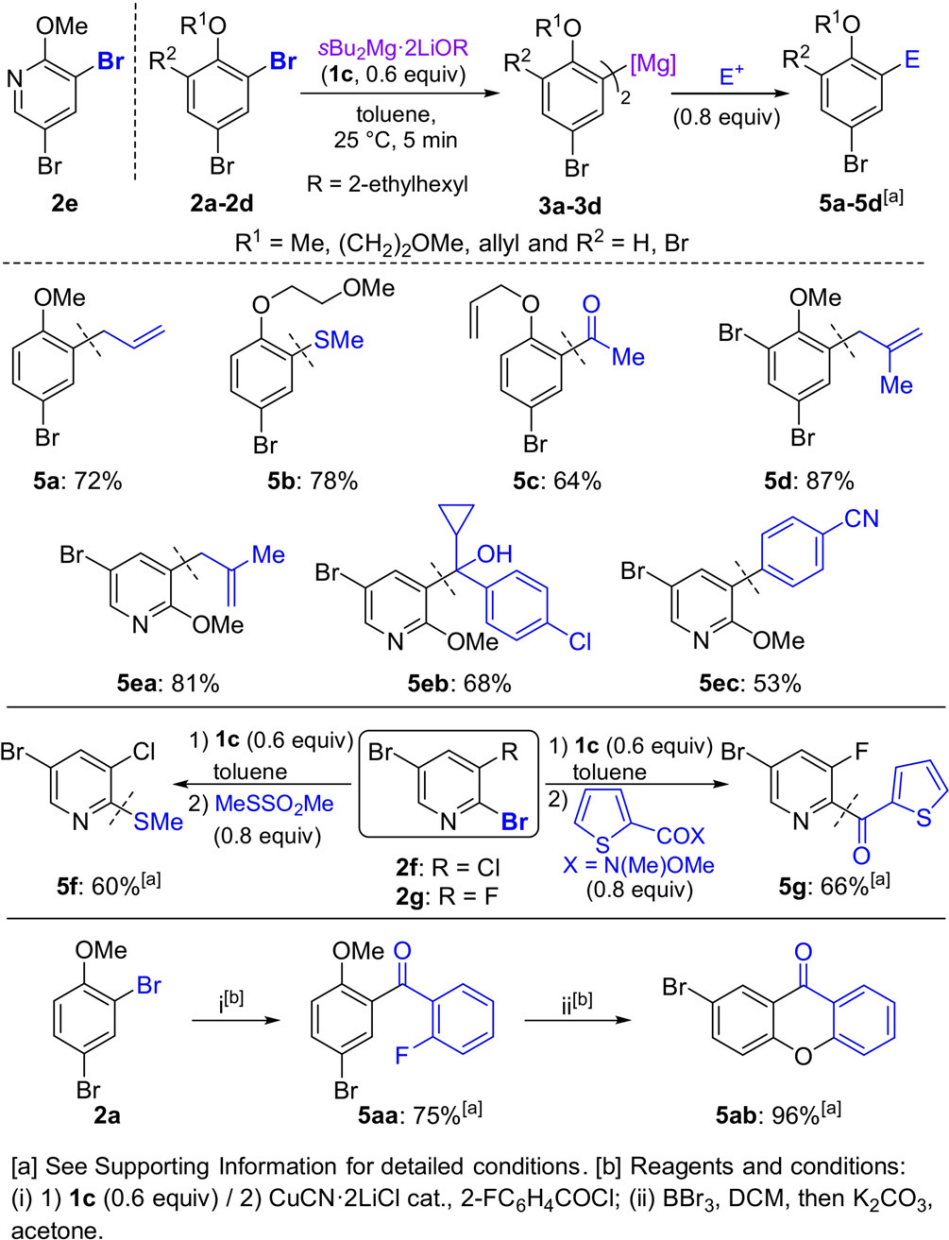
Reaction of various polybrominated (hetero)arenes with *s*Bu_2_Mg⋅2 LiOR (**1 c**), followed by electrophilic functionalization.

We next turned our attention to 2,5‐dibromo‐3‐methylthiophene (**6 a**), for which the exchange reagent **1 c** did not provide satisfactory regioselectivity (99 % conversion, **7 a**/**8 a**=90:10, Scheme 2).^[17]^ Since previous works have shown that, used as additives, Lewis donors[^6a^, ^10^] can enhance regioselectivities in halogen/metal exchange processes, we next probed the effect of adding *N*,*N*,*N′*,*N′*‐tetramethylethylenediamine (TMEDA)^[18]^ or PMDTA (0.6 equiv) to **1 c**, which led to the formation of **7 a** with a better control of regioselectivity (96:4, 99 % conversion for TMEDA, and 99:1, 99 % conversion for PMDTA).

**Scheme 2:**
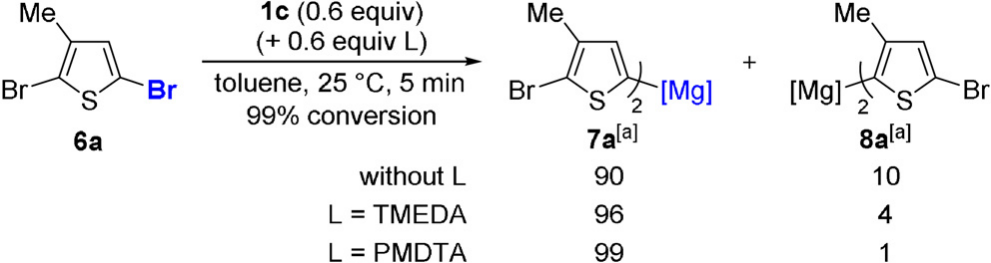
Screening of the regioselective Br/Mg exchange on 2,5‐dibromo‐3‐methylthiophene (**6 a**).

Trapping of **7 a** with 3‐methoxybenzaldehyde afforded the alcohol **9 a** in 80 % yield (Scheme 3). This donor effect was quite general and the same procedure was extended to other polyhalogenated (hetero)arenes. Thus, **6 b**–**6 d** underwent complete Br/Mg exchange upon treatment with **1 c⋅PMDTA**, leading to the less sterically hindered magnesium species. After allylation or addition to Michler's ketone, the polyfunctionalized products **9 b**–**9 d** were isolated in 61–83 % yield.

**Scheme 3:**
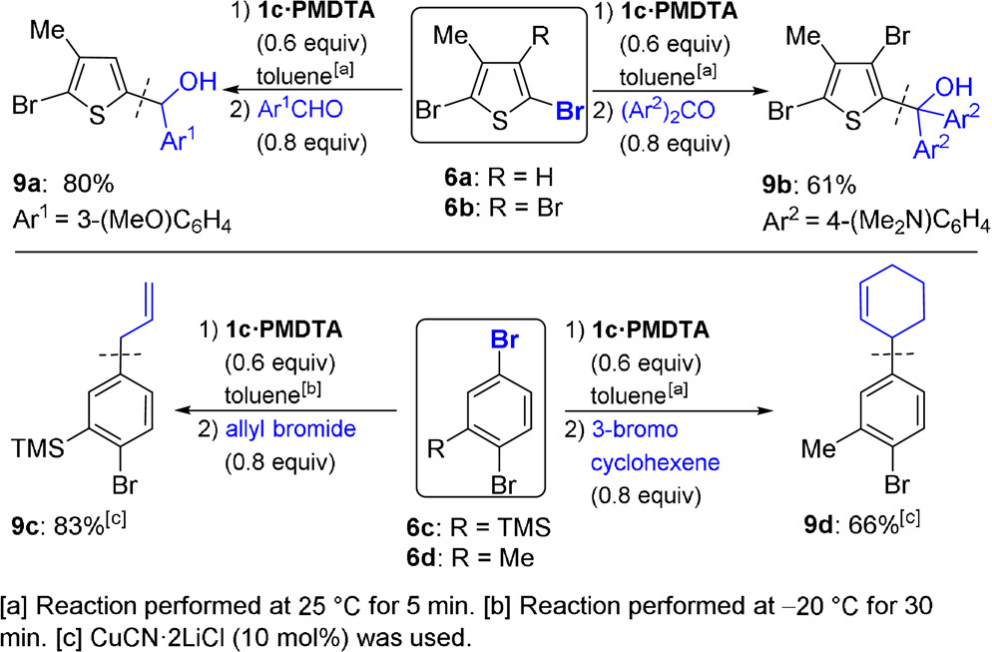
Reaction of various polybrominated (hetero)arenes with *s*Bu_2_Mg⋅2 LiOR in the presence of PMDTA (**1 c⋅PMDTA**), followed by electrophilic functionalization.

Interestingly, investigating the reactivity of **1 c** towards 2,5‐dibromopyridine (**10 a**)^[10]^ established that the regioselectivity of the Br/Mg exchange can be finely tuned, switching from C(2) to C(5) in the presence of Lewis donor PMDTA (Table 2).^[17]^

**Table 2:** Br/Mg exchange on 2,5‐dibromopyridine (**10 a**) using various exchange reagents. 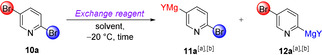

Entry	Exchange reagent^[c]^	Solvent	*t* [min]	Ratio **11 a**/**12 a**	Conv. [%]^[d]^
1	*i*PrMgCl⋅LiCl (**1 a**)	THF	120	99:1	94^[a]^
2	*s*Bu_2_Mg⋅2 LiOR (**1 c**)	toluene	30	1:99	99^[b]^
3	**1 c⋅PMDTA**	toluene	30	99:1	99^[b]^

[a] Y=Cl⋅LiCl. [b] Y=pyridyl⋅2 LiOR(⋅PMDTA). [c] R=2‐ethylhexyl, these reactions were carried out at 0.50 m using 1.2 equiv of alkylmagnesium species. Reagents are displayed according to their stoichiometry and not to their actual structure. [d] Conversion determined by GC‐analysis of reaction aliquots after aqueous quench.

Thus, **10 a** underwent selective Br/Mg exchange with turbo‐Grignard *i*PrMgCl⋅LiCl (**1 a**) at C(5) position to give the thermodynamically more favored product **11 a** (Table 2, entry 1). Alternatively, using *s*Bu_2_Mg⋅2 LiOR (**1 c**) in neat toluene furnished the kinetic C(2)‐magnesiation product **12 a** (Table 2, entry 2). While this regioselectivity is unprecedented for Br/Mg exchanges,^[19]^ previous studies using organolithium reagents have shown that the C(2)‐lithiation product isomerises quickly to the more stable C(5)‐lithiated species.^[10]^ Furthermore this unusual regioselectivity can be switched to C(5)‐magnesiation by adding PMDTA (0.6 equiv) to **1 c** (Table 2, entry 3). Conditions A and B described in entries 3 and 2, respectively, of Table 2 were then applied to various dibromopyridines and ‐quinolines (Scheme 4).

**Scheme 4:**
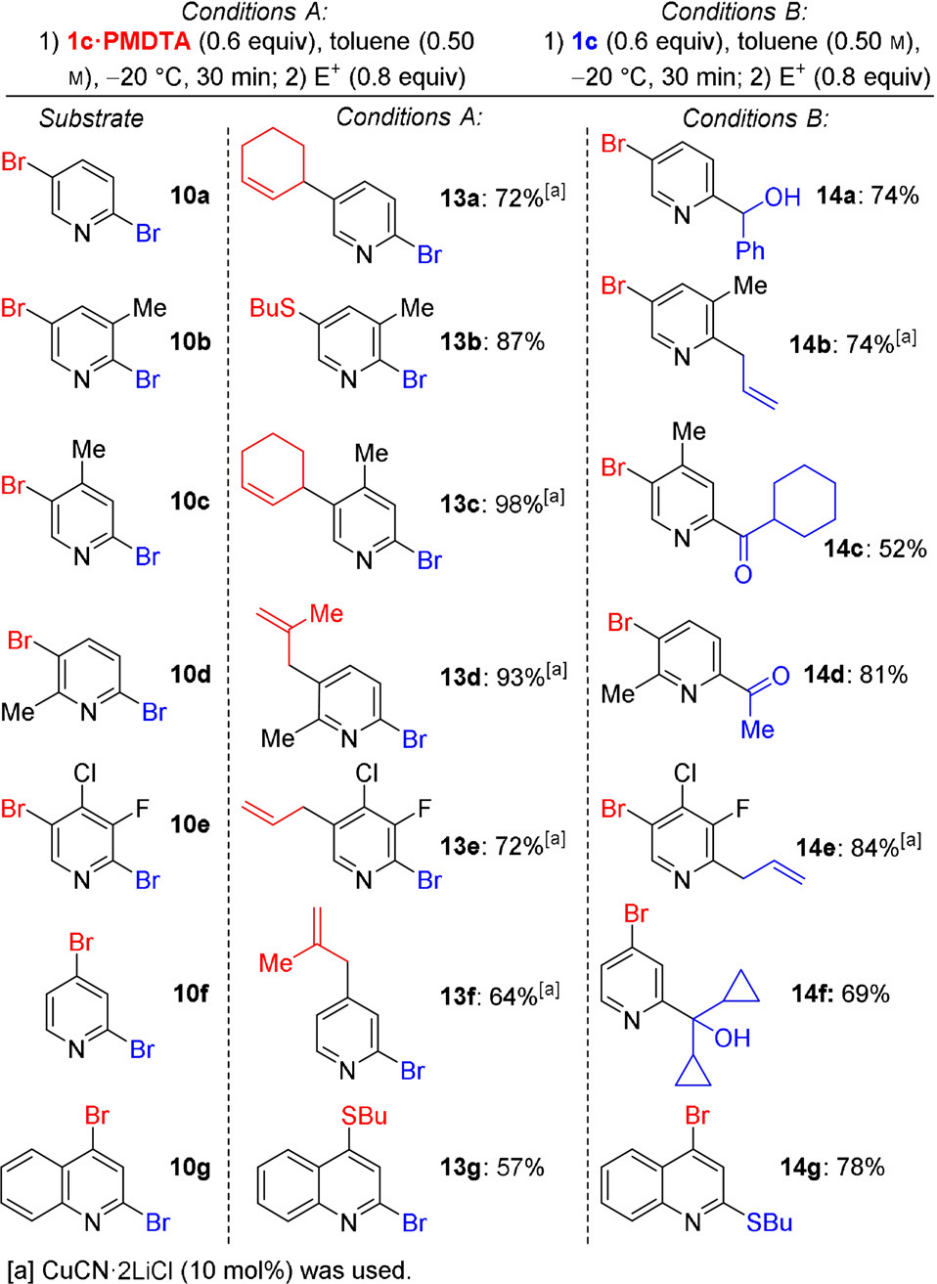
Reaction of various dibrominated heteroarenes with *s*Bu_2_Mg⋅2 LiOR⋅PMDTA (**1 c⋅PMDTA**, Conditions A) or **1 c** alone (Conditions B), followed by electrophilic functionalization.

Thus, following Conditions A (**1 c⋅PMDTA**, 0.6 equiv, toluene, −20 °C, 30 min), **10 a** was regioselectively converted into **11 a** which was trapped with 3‐bromocyclohexene, affording the C(5)‐allylated product **13 a** in 72 % yield. Using Conditions B (**1 c**, 0.6 equiv, toluene, −20 °C, 30 min), **10 a** was regioselectively converted into **12 a**, which was quenched with benzaldehyde, leading to the alcohol **14 a** in 74 % yield. Analogously, the methyl‐substituted pyridines **10 b**–**10 d**, either using Conditions A or B, produced the expected regioisomeric pyridylmagnesium derivatives, which were trapped by allylation, thioalkylation or acylation, affording **13 b**–**13 d** and **14 b**–**14 d** in 52–98 % yield. The electron‐deficient 2,5‐dibromo‐4‐chloro‐3‐fluoropyridine (**10 e**) underwent smooth Br/Mg exchange under Conditions A or B, forming—after addition of allyl bromide—the allylated compounds **13 e**–**14 e** in 72–84 % yield. This Br/Mg exchange was extended to 2,4‐dibromopyridine (**10 f**) and 2,4‐dibromoquinoline (**10 g**). The expected regioisomeric products **13 f**–**13 g** and **14 f**–**14 g** were isolated after thioalkylation, allylation or addition of dicyclopropyl ketone in 57–78 % yield.

Intrigued by this unique reactivity and the profound effect that Lewis donors cause on the regioselectivity of the Br/Mg exchange reactions, we next studied the constitution of these organometallic intermediates prior to electrophilic interception. Firstly, **1 c** was prepared in situ and reacted with 2‐bromoanisole (**15**, 2.0 equiv, toluene, 25 °C, 30 min), affording a pale yellow solution which deposited colourless crystals of [Ar_2_(OR)MgLi]_2_ (**16**, Ar=*o*‐MeO‐C_6_H_4_, R=2‐ethylhexyl, Figure 1).

**Figure 1:**
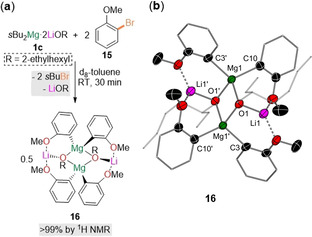
a) Formation of **16** via Br/Mg exchange in [D_8_]toluene at room temperature by reacting **1 c** with 2.0 equiv of **15** with concomitant elimination of LiOR and 2.0 equiv of *s*BuBr. b) Molecular structure of **16** with displacement ellipsoids at 50 % probability, all hydrogen atoms omitted, and with C atoms in 2‐ethylhexyl substituents and anisyl rings drawn as wire frames (except for C_*ipso*_ and C_*ortho*_) for clarity.^[20]^

X‐ray crystallographic studies confirmed the bimetallic constitution of **16**, which exists as a centrosymmetric contact‐ion‐pair dimer. Demonstrating that these reactions are genuine Br/Mg exchanges, the Mg is attached to two *ortho*‐metalated anisole groups, occupying the position previously filled by Br atoms in **15**. Alkoxide bridges complete the Mg coordination sphere. Contrastingly, the Li atom only binds to one OR ligand, achieving further coordinative stabilization via two OMe groups from the metalated anisole molecules. This special coordination of the Li atoms could be responsible for the marked Lewis donor effect observed in the regioselective control in these Br/Mg exchanges (see above). Thus, PMDTA could preferentially chelate the Li atoms, precluding their interaction with the donor substituents of the substrate, ultimately favoring the formation of solvent‐separated ion pair species, which would suppress any possible Li/Mg communication. Notably, Mulvey has recently stressed that bimetallic cooperation in deprotonative metalation reactions is key in order to achieve unique regioselectivities that cannot be replicated by single‐metal reagents,^[21]^ as illustrated by the *meta*‐magnesiation of toluene using a sodium magnesiate base in hexane.^[22]^ In these systems, Na acts as an intramolecular Lewis acid to engage the substrate, which, in turn, is deprotonated by the complexed magnesiate anion.

Another significant feature of **16** is that only one equivalent of the lithium alkoxide is incorporated into the final molecular arrangement despite two being present in the exchange reagent *s*Bu_2_Mg⋅2 LiOR (**1 c**). Further insight into the formation of **16** was gained by monitoring the reactions of **1 c** with **15** (2.0 equiv) in [D_8_]toluene (Figure 1), which showed that **16** is obtained quantitatively along with the concomitant formation of *s*BuBr and one equivalent of free LiOR.^[17]^ ^1^H‐DOSY NMR supports that the solid state structure of **16** is retained in toluene solution. The activation of both *s*Bu groups in **1 c** contrasts with the sluggish reactivity of *s*Bu_2_Mg or *s*BuMg(OR) towards **15**, showcasing the mediating role of lithium through forming a contacted anionically activated magnesiate species of enhanced Br/Mg exchange ability.^[17]^

Building on these findings we next assessed the reactivities of *i*PrMgCl⋅LiCl (**1 a**) and *n*Bu_2_Mg⋅2 LiOR (**1 d**) towards 2‐bromo‐4‐iodoanisole (**17**, Scheme 5). For this substrate, Li‐directing effects should favor the Br/Mg exchange *ortho* to the donating OMe group, whereas considering purely the activation of the C−halogen bond, functionalization at the C(4) position via I/Mg exchange should be preferred. Unsurprisingly, turbo‐Grignard **1 a** in neat THF reacts with the most activated site of **17**, undergoing exclusively I/Mg exchange, affording, after allylation, the anisole derivative **18** in 85 % yield. However, a completely different scenario plays out for **1 d** in toluene, where coordination effects dominate, encouraging reactivity *ortho* to the directing OMe group and hence triggering a Br/Mg exchange with a selectivity of 4:1. Subsequent allylation and chromatographical separation furnished **19** in 65 % yield (Scheme 5). Supporting this interpretation, and demonstrating the importance of non‐coordinating solvent toluene, addition of polydentate donor PMDTA which can chelate Li, switches off this Br/Mg exchange preference, offering an I/Mg exchange only.^[17]^

**Scheme 5:**
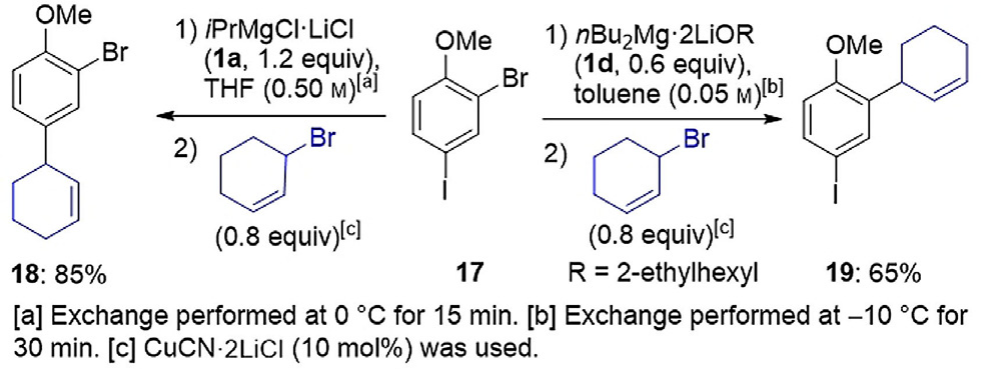
Selective Br/Mg exchange on 2‐bromo‐4‐iodoanisole (**17**) with *n*Bu_2_Mg⋅2 LiOR (**1 d**) followed by allylation reaction: comparison with *i*PrMgCl⋅LiCl (**1 a**).

Finally, NMR monitoring of the reaction of 2,5‐dibromopyridine (**10 a**) with *s*Bu_2_Mg⋅2 LiOR (**1 c**) in [D_8_]toluene at −20 °C for 30 min revealed complete consumption of the starting material, as evidenced by the presence of *s*BuBr and a distinct set of new resonances which we can attribute to **12 a**, the product of regioselective C(2) Br/Mg exchange.^[17]^ The most informative signals are those for the C(2) and C(5) positions in the ^13^C{^1^H} NMR spectra which appear at 140.5 and 119.8 ppm, respectively for **10 a** (Figure 2). After 30 min, complete disappearance of the signal assigned for C(2)−Br is accompanied by emergence of a new resonance in the aromatic region at 203.5 ppm,^[23]^ which is assigned to C(2)−Mg in **12 a**; whereas the chemical shift of the C(5)−Br hardly changes (118.5 ppm) with respect to the one observed for **10 a**.

**Figure 2:**
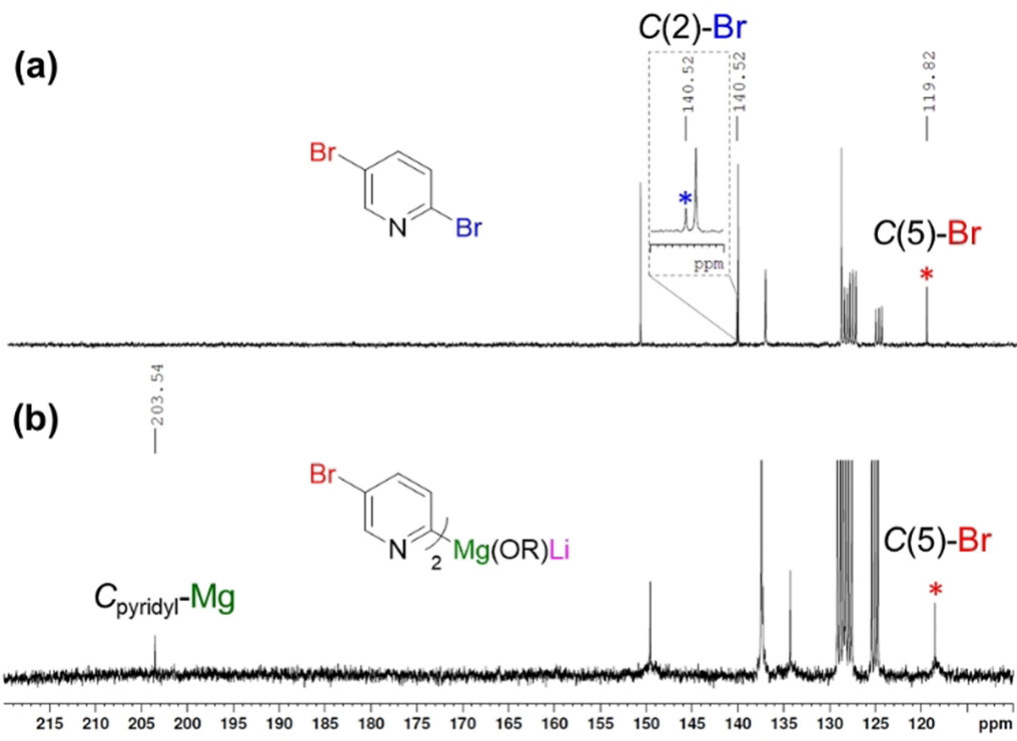
Aromatic region of the ^13^C{^1^H} NMR spectra in [D_8_]toluene of a) 2,5‐dibromopyridine (**10 a**) and b) **12 a**.

Additionally, ^1^H‐DOSY NMR displays co‐diffusion of the three new aromatic resonances related to the metalated arene alongside the signals defined for 2‐ethylhexanolate, consistent with them belonging to the same molecular entity in toluene solution with a mean diffusion coefficient of *D*=4.349×10^−10^ m^2^ s^−1^.^[17]^ Final observations revealed a second set of alkoxide‐related resonances in the aliphatic region of the ^13^C{^1^H} NMR, which did not belong to **12 a**, but bore a striking similarity to uncomplexed LiOR, as previously observed in the formation of **16**.

While **12 a** is thermally unstable, which precluded its crystallization, on the basis of these studies we can propose a structure similar to that of **16** (Scheme 6) but in this case the C(2) selectivity is driven by the coordination of Li to the pyridine N, guiding the Br/Mg exchange to the C(2) position. If a Lewis donor is added, this lithium‐directing effect no longer operates and, as shown in Table 2 and Scheme 4, the selectivity of the Br/Mg exchange switches to the C(5) position.

**Scheme 6:**
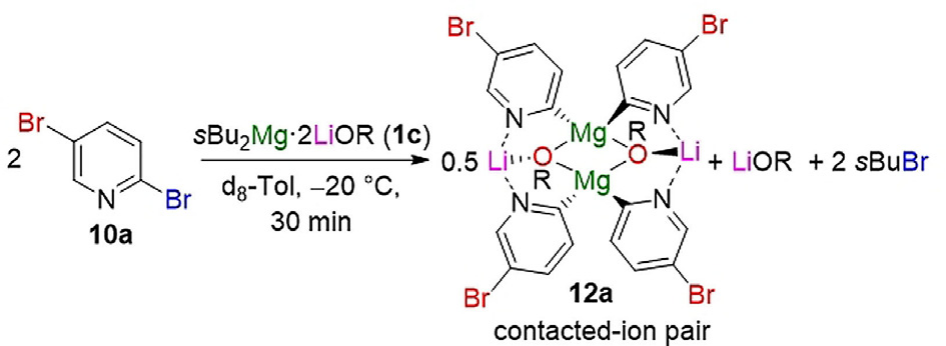
Reaction between **1 c** and **10 a** displaying regioselective C(2)−Br exchange facilitated by the Li−N interaction to give contacted ion pair lithium magnesiate **12 a**.

In conclusion, we have reported regioselective Br/Mg exchanges of dibromo(hetero)arenes performed by reagents of the type R_2_Mg⋅2 LiOR^1^ (R=*s*Bu, *n*Bu, R^1^=2‐ethylhexyl) in toluene. Addition of a chelating ligand such as PMDTA allowed in certain cases a regioselectivity switch of the exchange. This switch can be rationalized in terms of the bimetallic cooperation between Li and Mg. The preference of Li to coordinate to the Lewis basic sites of the substrate in toluene in the absence of any donor additives guides the Br/Mg exchange to the position adjacent to these basic sites, akin to the CIPE mechanism in metalation chemistry, thus enabling new regioselectivities not available using turbo‐Grignard reagents.

## Conflict of interest

The authors declare no conflict of interest.

## Supporting information

As a service to our authors and readers, this journal provides supporting information supplied by the authors. Such materials are peer reviewed and may be re‐organized for online delivery, but are not copy‐edited or typeset. Technical support issues arising from supporting information (other than missing files) should be addressed to the authors.

Supplementary
