# Supplementary material for: Regioselective Bromine/Magnesium Exchange for the Selective Functionalization of Polyhalogenated Arenes and Heterocycles
Source: Angew Chem Int Ed Engl. 2020 Nov 19;60(3):1513–8. doi: 10.1002/anie.202012496 (PMC7839478; doi:10.1002/anie.202012496)
Supplement: Supplementary file 1 — Supplementary [file ANIE-60-1513-s001.pdf]

Angewandte Chemie
Eine Zeitschrift der Gesellschaft Deutscher Chemiker

Supporting Information

Regioselective Bromine/Magnesium Exchange for the Selective Functionalization of Polyhalogenated Arenes and Heterocycles

Alexandre Desaintjean, Tobias Haupt, Leonie J. Bole, Neil R. Judge, Eva Hevia, and Paul Knochel**

anie_202012496_sm_miscellaneous_information.pdf

Supporting Information

Table of Content

General Information......SI-2
Typical Procedures......SI-13
Additional Results......SI-14
Preparation of Compounds 5, 9, 13-14, 16-17, SM1-4 and A-D......SI-18
X-Ray Crytallographic Studies......SI-52
NMR Studies......SI-54
Control Experiments......SI-65
NMR Spectra of Compounds 2, 5-6, 9-10, 13-14, 16-17, SM1-4 and A-D......SI-70

General Information

All reactions were carried out under argon or nitrogen atmosphere in glassware dried with a heat gun (650 °C) under high vacuum (<1 mbar) or under standard glove box techniques. Syringes which were used to transfer anhydrous solvents or reagents were purged thrice with argon or nitrogen prior to use. The Br/Mg-exchange on (hetero)aryl bromides was checked by quenching a reaction aliquot with an aq. solution of sat. NH_4Cl, followed by GC/GC-MS analysis. Indicated yields are isolated yields of compounds estimated to be >95% pure as determined by ^{1}H-NMR (25 °C) and capillary GC-analyses. Unless otherwise indicated, all reagents were obtained from commercial sources. GC-spectra were obtained using an Agilent Technologies 7890A GC System, Agilent Technologies 5975C Inert XL EI/CI MSD with Triple-Axis Detector, Agilent Technologies 7693 Autosampler and Restek GC Column (30 m, 0.25 mm i.d., 0.25 µm).

Solvents

Solvents were dried according to standard procedures by distillation over drying agents and stored under argon.

Tetrahydrofuran (THF) was continuously refluxed and freshly distilled from sodium benzophenone ketyl under nitrogen and stored over molecular sieves (3 Å).

Toluene was continuously refluxed and freshly distilled from sodium under nitrogen and stored over molecular sieves (3 Å).

CH_2Cl_2 (DCM) and **Me_2NCHO (DMF)** were distilled from CaH_2 and stored over molecular sieves (3 Å).

Solvents for column chromatography were distilled on a rotary evaporator prior to use.

Et_2O was predried over calcium hydride and dried with the solvent purification system SPS-400-2 from Innovative Technologies Inc.

Reagents

All reagents were obtained from commercial sources and used without further purification unless otherwise stated.

***n*BuLi** solution was purchased from Albemarle (Hoechst, Germany).

***s*BuLi** solution in cyclohexane was purchased from Albemarle or Sigma Aldrich.

***t*BuLi** solution in pentane was purchased from Albemarle or Sigma Aldrich.

***n*Bu_2Mg** solution in hexane was purchased from Albemarle.

Magnesium 2-ethylhexanolate solution in *n*heptane was purchased from Albemarle.

0.50 M LiCl solution in THF: LiCl (5 mmol) was dried in vacuo using a heatgun (400 °C) for 10 min. After cooling to room tempetature, dry THF (10 mL) was added and the mixture stirred until the salt was dissolved completely.

1.00 M CuCN·2LiCl solution in THF: CuCN (80.0 mmol, 7.17 g) and LiCl (160 mmol, 6.77 g) were dried in a *Schlenk*-flask under vacuum at 140 °C for 12 h. After cooling, dry THF (80 mL) was added and stirring continued until the salts were dissolved.[1]

1.00 M $ZnCl_2$ solution in THF: $ZnCl_2$ (100 mmol, 13.6 g) was dried in a *Schlenk*-flask under vacuum at 140 °C for 5 h. After cooling, 100 mL dry THF were added and stirring was continued until the salt was dissolved.

***i*PrMgCl·LiCl (1a):** Magnesium turnings (2.67 g, 110 mmol) and anhydrous LiCl (4.66 g, 100 mmol) were placed in an argon-flushed-*flask* and THF (50 mL) was added. A solution of *i*PrCl (9.13 mL, 100 mmol) in THF (50 mL) was slowly added at 25 °C. The reaction starts within a few minutes. After complete addition, the reaction mixture was stirred for 12 h at 25 °C. The grey solution of *i*PrMgCl·LiCl was cannulated to another-*flask* under argon and removed in this way from excess of magnesium. A yield of ca. 95-98% of *i*PrMgCl·LiCl was obtained.[2]

Content determination of organometallic reagents

***i*PrMgCl·LiCl** was titrated with I_2 in THF at 0 °C.[3]

---

[1] P. Knochel, M. C. P. Yeh, S. C. Berk, J. Talbert, *J. Org. Chem.* **1988**, *53*, 2390-2392.
[2] A. Krasovskiy, P. Knochel, *Angew. Chem. Int. Ed.* **2004**, *43*, 3333-3336.
[3] A. Krasovskiy, P. Knochel, *Synthesis* **2006**, 890-891.

***s*BuLi** was titrated with *N*-benzylbenzamide in THF at -40 °C.[4]

Organolithium (*n*BuLi, *t*BuLi) reagents were titrated with menthol and 1,10-phenanthroline as indicator in THF at 0 °C.[5]

***n*Bu$_2$Mg** was titrated with I_2 in a 0.50 M LiCl THF solution at 0 °C.

Magnesium 2-ethylhexanolate was titrated by acidimetric titration with 4-(phenylazo)-diphenylamine and CF_3CO_2H (TFA) in toluene at 0 °C.

Chromatography

Flash column chromatographical purifications were performed using silica gel 60 (0.040-0.063 mm) from Merck.

Thin layer chromatography was performed using SiO_2 pre-coated aluminum plates (Merck 60, F-254). The chromatograms were examined under 254 nm UV irradiation, by incubating the plates in an iodine chamber and/or by staining the TLC plate with a $KMnO_4$ solution followed by heating with a heat gun.

Analytical Data

^{1}H-NMR, ^{13}C-NMR, ^{19}F-NMR and **2D-NMR** spectra were recorded on Varian Mercury 200, Bruker ARX 300, Varian VXR 400 S and Bruker AMX 600 instruments. Chemical shifts are reported as values in ppm relative to tetramethylsilane. $CDCl_3$ peaks were set to 7.26 ppm in ^{1}H-NMR and 77.16 ppm in ^{13}C-NMR experiments. D_8-Toluene peaks were set to 2.08 ppm in ^{1}H-NMR and 20.43 ppm in ^{13}C-NMR experiments. The following abbreviations were used to characterize signal multiplicities: s (singlet), d (doublet), t (triplet), q (quartet), qn (quintet), spt (septet), dd (doublet of doublets), dt (doublet of triplets), as well as m (multiplet).

Mass spectroscopy: High resolution (HRMS) and low resolution (MS) spectra were recorded on a Finnigan Mat 95Q instrument. Electron impact ionization (EI) was conducted with an ionization energy of 70 eV. For coupled gas chromatography/mass spectrometry, a Hewlett-

---

[4] Burchat, A. F.; Chong, J. M.; Nielsen, N., *J. Organomet. Chem.* **1997**, *542*, 281-283.
[5] a) H.-S. Lin, L. A. Paquette, *Synth. Commun.* **1994**, *24*, 2503-2506; b) S. C. Watson, J. F. Eastham, *J. Organomet. Chem.* **1967**, *9*, 165-168.

Packard HP 6890/MSD 5973 GC/MS system was used. Molecular fragments are reported starting at a relative intensity of 10-20%.

Infrared spectra (IR) were recorded from 4500 cm^{-1} to 650 cm^{-1} on a Perkin Elmer Spectrum BX-59343 instrument. For detection a Smiths Detection DuraSampl*IR* II Diamond ATR sensor was used. The main absorption peaks are reported in cm^{-1}.

Melting points (M.p.) were determined on a Büchi B-540 melting point apparatus and are uncorrected.

^{1}H-NMR, ^{13}C-NMR and **^{7}Li-NMR spectra** for NMR studies were recorded on a Bruker DPX 300 MHz spectrometer, operating at 300.1 MHz for ^{1}H, 75.5 MHz for ^{13}C{1H} and 116.6 MHz for ^{7}Li.

Elemental analysis was obtained with a Flash 2000 Organic Elemental Analyser (Thermo Scientific).

Preparation of sBuMgOCH$_2$CH(Et)Bu·LiOCH$_2$CH(Et)Bu (1b):[6]

Method A:

A dry and argon-flushed *Schlenk*-flask, equipped with a magnetic stirring bar and a septum, was charged with nBu$_2$Mg (0.66 M in hexane, 15.0 mL, 9.90 mmol) and the reaction mixture was cooled to 0 °C. Then, 2-ethylhexanol (3.10 mL, 19.8 mmol) was added dropwise. After 12 h a gelatinous solution was obtained. To the reaction mixture sBuLi (1.21 M in hexane, 8.18 mL, 9.9 mmol) was added dropwise. After the addition was complete, the reaction mixture was allowed to warm to room temperature for 2 h. The solvents were removed under vaccum affording a lightly yellow foam. Freshly distilled toluene (9 mL) was added under vigourous stirring at 0 °C. The freshly prepared sBuMgOCH$_2$CH(Et)Bu·LiOCH$_2$CH(Et)Bu was titrated prior to use at 0 °C by iodometric titration.[3] The sBuMgOCH$_2$CH(Et)Bu·LiOCH$_2$CH(Et)Bu concentration of the resulting clear solution was 1.00-1.50 M.

Method B:

A dry and argon-flushed *Schlenk*-flask, equipped with a magnetic stirring bar and a septum, was charged with Mg[OCH$_2$CH(Et)Bu]$_2$ (0.85 M in heptane, 15.0 mL, 12.8 mmol)[7] and was cooled to 0 °C. Then, sBuLi (1.21 M in hexane, 10.6 mL, 12.8 mmol) was added dropwise. After the addition was complete, the reaction mixture was allowed to warm to room temperature for 2 h. The solvents were removed under vaccum affording a lightly yellow foam. Freshly distilled toluene (9 mL) was added under vigourous stirring at 0 °C. The prepared sBuMgOCH$_2$CH(Et)Bu·LiOCH$_2$CH(Et)Bu was titrated prior to use at 0 °C by iodometric titration.[3] The sBuMgOCH$_2$CH(Et)Bu·LiOCH$_2$CH(Et)Bu concentration of the resulting clear solution was 1.00-1.50 M.

Preparation of sBu$_2$Mg·2LiOCH$_2$CH(Et)Bu (1c):[6]

Method A:

A dry and argon-flushed *Schlenk*-flask, equipped with a magnetic stirring bar and a septum, was charged with nBu$_2$Mg (0.66 M in hexane, 15.0 mL, 9.90 mmol) and the reaction mixture

[6] D. S. Ziegler, K. Karaghiosoff, P. Knochel, *Angew. Chem. Int. Ed.* **2018**, *57*, 6701.
[7] This magnesium alkoxide solution (0.94 M in $n$heptane) is commercially available from Albemarle, Frankfurt: U. Wietelmann, U. Emmel, J. Roeder, M. Steinbild, K. Papstein (Albemarle), WO-2010146122, **2010**.

was cooled to 0 °C. Then, 2-ethylhexanol (3.10 mL, 19.8 mmol) was added dropwise. After 12 h a gelatinous solution was obtained. To the reaction mixture *s*BuLi (1.21 M in hexane, 16.36 mL, 19.8 mmol) was added dropwise. After the addition was complete, the reaction mixture was allowed to warm to room temperature for 2 h. The solvents were removed under vaccum affording a lightly yellow foam. Freshly distilled toluene (9 mL) was added under vigourous stirring at 0 °C. The prepared $sBu_2Mg{\cdot}2LiOCH_2CH(Et)Bu$ was titrated prior to use at 0 °C by iodometric titration.[3] The $sBu_2Mg{\cdot}2LiOCH_2CH(Et)Bu$ concentration of the resulting clear solution was 0.60-0.85 M.

Method B:

A dry and argon-flushed *Schlenk*-flask, equipped with a magnetic stirring bar and a septum, was charged with $Mg[OCH_2CH(Et)Bu]_2$ (0.85 M in heptane, 15.0 mL, 12.8 mmol)[7] and was cooled to 0 °C. Then, *s*BuLi (1.21 M in hexane, 21.2 mL, 25.6 mmol) was added dropwise. After the addition was complete, the reaction mixture was allowed to warm to room temperature for 2 h. The solvents were removed under vaccum affording a lightly yellow foam. Freshly distilled toluene (9 mL) was added under vigourous stirring at 0 °C. The freshly prepared $sBu_2Mg{\cdot}2LiOCH_2CH(Et)Bu$ was titrated prior to use at 0 °C by iodometric titration.[3] The $sBu_2Mg{\cdot}2LiOCH_2CH(Et)Bu$ concentration of the resulting clear solution was 0.60-0.85 M.

Note 1: Analogous reagents $tBu_2Mg{\cdot}2LiOR$ and $nBu_2Mg{\cdot}2LiOR$ (**1d**) were prepared following the same procedures using *t*BuLi or *n*BuLi instead of *s*BuLi and gave similar concentrations.

Note 2: All reagents should be storred at -20 °C and used within 2 weeks.

Titration Using Iodine[3]

A dry-*flask* was charged with accurately weighed I_2 (128 mg, 0.504 mmol), fitted with a rubber septum, and flushed with argon. THF (2 mL) was added and stirring was started. After the iodine was completely dissolved, the resulting brown solution was cooled to 0 °C in an ice bath and the organomagnesium reagent was added dropwise via a 1.00-mL syringe (0.01-mL graduations) until the brown color disappeared. The amount consumed contains 1.0 equiv of the organometallic reagent relative to iodine in the case of monoorganometallic reagents and 0.5 equiv for diorganometallic reagents.

Starting Materials

Synthesis of 2,4-dibromo-1-(2-methoxyethoxy)benzene (2b)

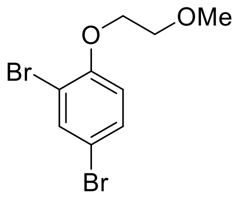

A dry and argon-flushed *Schlenk*-flask, equipped with a magnetic stirring bar and a septum, was charged with 2,4-dibromophenol (1.00 g, 3.97 mmol) and DMF (10 mL) and was cooled to 0 °C. NaH (60%, 191 mg, 4.76 mmol) was slowly added at 0 °C and the reaction mixture was stirred for 30 min. 1-Chloro-2-methoxyethane (451 mg, 4.76 mmol) was then added at 0 °C and the reaction mixture was stirred at 100 °C overnight. The mixture was quenched with a sat. aq. NH_4Cl solution (10 mL), diluted with water (10 mL) and extracted with ethyl acetate (3 x 30 mL). The combined organic extracts were dried over Na_2SO_4, filtered and concentrated *in vacuo*. The crude product was purified *via* column chromatography (*iso*hexane:ethyl acetate = 9:1, R_f = 0.43) to give the product **2b** (1.22 g, 3.94 mmol, 99% yield) as a brown oil.

^{1}H-NMR (400 MHz, $CDCl_3$, ppm): δ = 7.66 (d, *J* = 2.4 Hz, 1H), 7.35 (dd, *J* = 8.8, 2.4 Hz, 1H), 6.80 (d, *J* = 8.8 Hz, 1H), 4.31 – 3.99 (m, 2H), 3.86 – 3.74 (m, 2H), 3.47 (s, 3H).
^{13}C-NMR (101 MHz, $CDCl_3$, ppm): δ = 154.8, 135.7, 131.3, 114.9, 113.4, 70.9, 69.4, 59.7.
IR (ATR, cm^{-1}) $\tilde{\nu}$ = 2926, 2879, 1579, 1474, 1449, 1382, 1369, 1283, 1264, 1246, 1197, 1150, 1127, 1097, 1083, 1057, 1041, 926, 866, 798, 696, 677.
MS (EI, 70 eV, %) m/z = 312 (48), 310 (97), 308 (50), 254 (47), 252 (100), 251 (12), 250 (53), 225 (16), 223 (33), 221 (17), 156 (13), 154 (13), 145 (10), 143 (10), 75 (11), 63 (12), 59 (64).
HRMS (EI, 70 eV) m/z: calc. for **$C_9H_{10}Br_2O_2$**: 307.9048; found: 307.9041.

Synthesis of 1-(allyloxy)-2,4-dibromobenzene (2c)

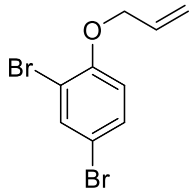

A dry and argon-flushed *Schlenk*-flask, equipped with a magnetic stirring bar and a septum, was charged with 2,4-dibromophenol (5.04 g, 20.0 mmol), DMF (10 mL) and K_2CO_3 (3.32 g, 24.0 mmol) and stirred for 5 min. Allyl bromide (2.42 g, 20.0 mmol) was slowly added and the reaction mixture was stirred overnight. The mixture was diluted with water (100 mL) and extracted with hexanes (3 x 100 mL). The combined organic extracts were dried over Na_2SO_4, filtered and concentrated *in vacuo*. The crude product was purified *via* silica plug (*iso*hexane) to give the product **2c** (4.87 g, 16.7 mmol, 84% yield) as a colorless oil.

^{1}H-NMR (400 MHz, $CDCl_3$, ppm): δ = 7.67 (d, *J* = 2.4 Hz, 1H), 7.35 (dd, *J* = 8.8, 2.4 Hz, 1H), 6.76 (d, *J* = 8.8 Hz, 1H), 6.04 (ddt, *J* = 17.3, 10.2, 5.0 Hz, 1H), 5.47 (dq, *J* = 17.3, 1.7 Hz, 1H), 5.32 (dq, *J* = 10.6, 1.5 Hz, 1H), 4.59 (dt, *J* = 5.0, 1.6 Hz, 2H).
^{13}C-NMR (101 MHz, $CDCl_3$, ppm): δ = 154.4, 135.7, 132.3, 131.3, 118.2, 114.8, 113.3 (2C), 70.0.

The spectra matched those of the literature.[8]

[8] W. Lasek, M. Makosza, *Synthesis* **1993**, 780-782.

Synthesis of (2,5-dibromophenyl)trimethylsilane (6c)

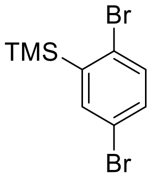

A dry and argon-flushed *Schlenk*-flask, equipped with a magnetic stirring bar and a septum, was charged with *i*PrMgCl·LiCl (7.9 mL, 8.70 mmol) and was cooled to -50 °C. 1,2,4-Tribromobenzene (2.50 g, 7.90 mmol) was added at -50 °C and the reaction mixture was stirred for 2 h. The completion of the bromine/magnesium-exchange was checked by GC-analysis of reaction aliquots quenched with water, using undecane as internal standard. Trimethylsilyl chloride (2.0 mL, 15.8 mmol) was then added at -20 °C and the reaction mixture was allowed to warm to room temperature overnight. After complete conversion, the mixture was quenched with a sat. aq. NH_4Cl solution (10 mL), diluted with water (10 mL) and extracted with ethyl acetate (3 x 30 mL). The combined organic extracts were dried over Na_2SO_4, filtered and concentrated *in vacuo*. The crude product was purified *via* column chromatography (*iso*hexane, R_f = 0.90) to give the product **6c** (2.08 g, 6.75 mmol, 85% yield) as a colorless oil.

^{1}H-NMR (400 MHz, $CDCl_3$, ppm): δ = 7.40 (d, *J* = 2.5 Hz, 1H), 7.28 (s, 1H), 7.19 (dd, *J* = 8.4, 2.5 Hz, 1H), 0.30 (s, 9H).

^{13}C-NMR (101 MHz, $CDCl_3$, ppm): δ = 144.2, 138.7, 134.5, 133.7, 128.9, 121.6, -0.6.

IR (ATR, cm^{-1}) ṽ = 2954, 2899, 1560, 1541, 1535, 1438, 1407, 1355, 1260, 1249, 1136, 1102, 1084, 1045, 1024, 1013, 886, 837, 810, 761, 751, 703, 690, 661.

MS (EI, 70 eV, %) m/z = 308 (12), 295 (50), 293 (100), 291 (52), 213 (23), 211 (28), 171 (13), 169 (13), 149 (27), 131 (50), 105 (22).

HRMS (EI, 70 eV) m/z: calc. for **$C_9H_{12}Br_2Si$**: 305.9075; found: 305.9066.

Synthesis of 2,5-dibromo-4-chloro-3-fluoropyridine (10e)

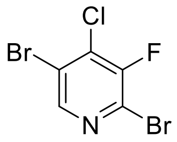

A dry and argon flushed round-bottomed-*flask*, equipped with a magnetic stirring bar, was charged with diisopropylamine (2.18 mL, 15.5 mmol) and freshly distilled THF (45 mL). The mixture was cooled to -78 °C and *n*BuLi (6.62 mL, 14.1 mmol) was slowly added. The reaction mixture was stirred for 10 min then cooled to 0 °C for 5 min. Then, 2,5-dibromo-3-fluoropyridine (**2g**, 3.6 g, 14.1 mmol, dissolved in 10 mL THF) was slowly added at -78 °C and the mixture was stirred for 1 h. After completion of the deprotonation, 1,1,2-trichloro-1,2,2-trifluoroethane (2.51 mL, 21.2 mmol) was slowly added at -78 °C. The reaction mixture was allowed to warm to room temperature overnight. The mixture was then quenched with a sat. aq. NH_4Cl solution (10 mL), diluted with water (10 mL) and extracted with ethyl acetate (3 x 30 mL). The combined organic extracts were dried over Na_2SO_4, filtered and concentrated *in vacuo*. The crude product was purified *via* column chromatography (*iso*hexane, R_f = 0.28) to give the product **10e** (2.67 g, 9.23 mmol, 65% yield) as a colorless oil.

^{1}H-NMR (400 MHz, $CDCl_3$, ppm): δ = 8.38 (s, 1H).
^{13}C-NMR (101 MHz, $CDCl_3$, ppm): δ = 153.1 (d, *J* = 265.7 Hz), 147.0 (d, *J* = 6.6 Hz), 132.8 (d, *J* = 17.9 Hz), 129.0 (d, *J* = 23.8 Hz), 121.4 (d, *J* = 4.5 Hz).
^{19}F-NMR (377 MHz, $CDCl_3$, ppm): δ = -107.3.
IR (ATR, cm^{-1}) $\tilde{v}$ = 1540, 1418, 1394, 1280, 1212, 1190, 1121, 1102, 907, 891, 807, 784.
MS (EI, 70 eV, %) m/z = 293 (12), 291 (70), 289 (100), 287 (42), 212 (16), 210 (64), 208 (49), 131 (21), 129 (62), 94 (11).
HRMS (EI, 70 eV) m/z: calc. for **C_5HBr_2ClFN**: 288.8128; found: 288.8124.

Synthesis of *S*-butyl benzenesulfonothioate[9]

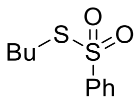

A dry and argon-flushed *Schlenk*-flask, equipped with a magnetic stirring bar and a septum, was charged with butane-1-thiol (4.28 mL, 40.0 mmol), DCM (50 mL), water (25 mL) and iodine (5.58 g, 22 mmol) and stirred at room temperature for 30 min. The mixture was extracted with DCM (3 x 30 mL). The combined organic extracts were washed with water and dried over Na_2SO_4, filtered and concentrated *in vacuo*. The resulting solution of dibutyl disulfide was mixed with sodium benzenesulfinate (10.5 g, 64.0 mmol), iodine (10.1 g, 40 mmol) and DCM (50 mL) and stirred for 22 h at room temperature. Then, a 0.10 M $Na_2S_2O_3$ solution was added to quench the excess of iodine. The organic phase was washed and dried and the solvent removed under reduced pressure to afford *S*-butyl benzenesulfonothioate (8.77 g, 38.1 mmol, 95% yield) as a yellow oil.

^{1}H-NMR (400 MHz, $CDCl_3$, ppm): δ = 8.10 – 7.81 (m, 2H), 7.69 – 7.60 (m, 1H), 7.60 – 7.50 (m, 2H), 3.00 (t, *J* = 7.4 Hz, 2H), 1.57 (tt, *J* = 8.8, 6.9 Hz, 2H), 1.32 (dq, *J* = 14.6, 7.3 Hz, 2H), 0.84 (t, *J* = 7.4 Hz, 3H).
^{13}C-NMR (101 MHz, $CDCl_3$, ppm): δ = 145.0, 133.7, 129.4, 127.1, 35.9, 30.7, 21.8, 13.5.
IR (ATR, cm^{-1}) $\tilde{\nu}$ = 2960, 2874, 1465, 1458, 1447, 1322, 1307, 1292, 1139, 1099, 1077, 1023, 999, 754, 714, 685, 670.
MS (EI, 70 eV, %) m/z = 141 (19), 125 (18), 97 (13), 89 (56), 77 (100), 55 (39).
HRMS (EI, 70 eV) m/z: calc. for $\mathbf{C_{10}H_{14}O_2S_2}$: 230.0435; found: 230.0507.

9 Adapted procedure from: K. Fujiki, N. Tanifuji, Y. Sasaki, T. Yokoyama, *Synthesis* **2002**, 343-348.

Typical Procedures

Typical Procedure 1: Preparation of Di(hetero)arylmagnesium Alkoxides via a Bromine/Magnesium-Exchange Followed by Electrophilic Functionalization

A dry and argon-flushed *Schlenk*-flask, equipped with a magnetic stirring bar and a septum, was charged with the corresponding (hetero)aryl bromide (1.0 equiv) and dissolved in dry toluene (0.50 M or 0.05 M, specified for every single procedure). When needed, *N,N*,*N'*,*N''*,*N''*-pentamethyldiethylenetriamine (PMDTA, 0.6 equiv, specified for every single procedure) was added. Then, the exchange reagent $R'_2Mg\cdot 2LiOR$ (R = 2-ethylhexyl, R' = *s*Bu for **1c** or *n*Bu for **1d**, 0.6 equiv) was added dropwise at the specified temperature and the reaction stirred for the indicated time. The completion of the bromine/magnesium-exchange was checked by GC-analysis of reaction aliquots quenched with a sat. aq. NH_4Cl solution, using undecane as internal standard. Subsequent reactions with electrophiles were carried out under the indicated conditions. After complete conversion, the mixture was quenched with a sat. aq. NH_4Cl solution (10 mL), diluted with water (10 mL) and extracted with ethyl acetate (3 x 30 mL). The combined organic extracts were dried over Na_2SO_4, filtered and concentrated *in vacuo*. The crude product was purified by flash chromatography on silica gel using the appropriate eluent.

Additional Results

Complete table of optimization for Scheme 2

Table S1. Screening of the regioselective Br/Mg-exchange on 2,5-dibromo-3-methylthiophene (**6a**).

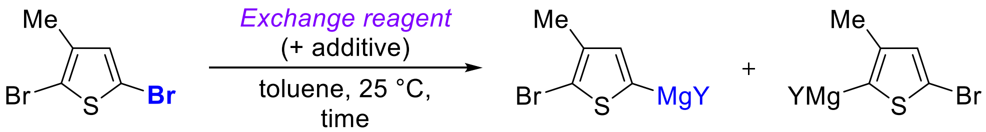

6a **7a**[a],[b],[c] **8a**[a],[b],[c]

Entry	Exchange reagent[d]	Time (min)	Ratio **7a**:**8a**	Conv. [%][e]
1	*i*PrMgCl·LiCl (**1a**)[f]	60	80:20	99[a]
2	*s*BuMgOR·LiOR (**1b**)	30	76:24	40[b]
3	**1b·TMEDA**	30	99:1	66[b]
4	*s*Bu_2Mg·2LiOR (**1c**)	5	90:10	99[c]
5	*t*Bu_2Mg·2LiOR	5	70:30	99[c]
6	*n*Bu_2Mg·2LiOR (**1d**)	5	84:16	99[c]
7	**1c·TMEDA**	5	96:4	99[c]
8[g]	**1c·PMDTA**	5	99:1	99[c]

[a] Y = Cl·LiCl. [b] Y = OR·LiOR. [c] Y = thienyl·2LiOR(·ligand). [d] R = 2-ethylhexyl, these reactions were carried out at 0.50 M using 1.2 equiv of alkylmagnesium species. Reagents are displayed accordingly to their stoichiometry and not their actual structure. [e] Conversion determined by GC-analysis of reaction aliquots after aqueous quench. [f] Reaction performed in THF at −20 °C. [g] When performed in THF, a ratio **7a**:**8a** = 71:29 and a conversion of 53% were obtained.

Complete table of optimization for Table 2

Table S2. Br/Mg-exchange on 2,5-dibromopyridine (**10a**) using various exchange reagents.

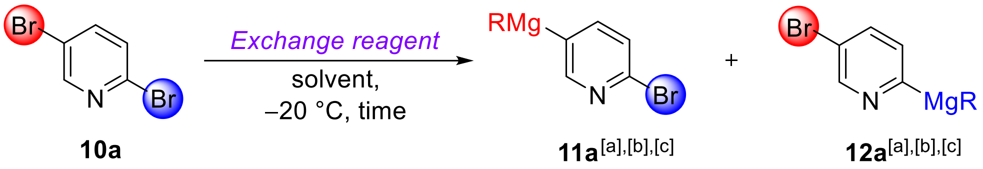

Entry	Exchange reagent[d]	Solvent	Time (min)	Ratio **11a**:**12a**	Conv. [%][e]
1	*i*PrMgCl·LiCl (**1a**)	THF	120	99:1	94[a]
2	*s*BuMgOR·LiOR (**1b**)	toluene	60	1:99	20[b]
3	*s*Bu$_2$Mg·2LiOR (**1c**)	toluene	30	1:99	99[c]
4	*s*BuMgOR·LiOR·**TMEDA** (**1b**·**TMEDA**)	toluene	60	99:1	81[b]
5	*s*Bu$_2$Mg·2LiOR·**PMDTA** (**1c**·**PMDTA**)	toluene	30	99:1	99[c]

[a] R = Cl·LiCl. [b] R = OR·LiOR(·ligand). [c] R = pyridyl·2LiOR(·ligand). [d] R = 2-ethylhexyl, reactions were carried out at 0.5 M using 1.2 equiv of alkylmagnesium species. Reagents are displayed accordingly to their stoichiometry and not their actual structure. [e] Conversion determined by GC-analysis of reaction aliquots after aqueous quench.

Note: The addition of 12-crown-4 (2.4 equiv)[10] had the same effect as a chelating ligand, producing a majority of **11a** (**11a**:**12a** = 90:10) with 57% of conversion.

[10] For literature about 12-crown-4, a specific lithium cation ionophore, see: a) C. J. Pedersen, *J. Am. Chem. Soc.* **1967**, *89*, 2495-2496; b) C. J. Pedersen, *J. Am. Chem. Soc.* **1967**, *89*, 7017-7036; c) F. A. L. Anet, J. Krane, J. Dale, K. Daasvatn, P. O. Kristiansen, *Acta Chem. Scand.* **1973**, *27*, 3395-3402; d) A. Pullman, C. Giessner-Prettre, Y. V. Kruglyak, *Chem. Phys. Lett.* **1975**, *35*, 156-160.

Additional results Br/Mg vs. I/Mg-exchanges

Table S3. Br/Mg-exchange in the presence of an iodine on 2-bromo-4-iodoanisole (**15**).

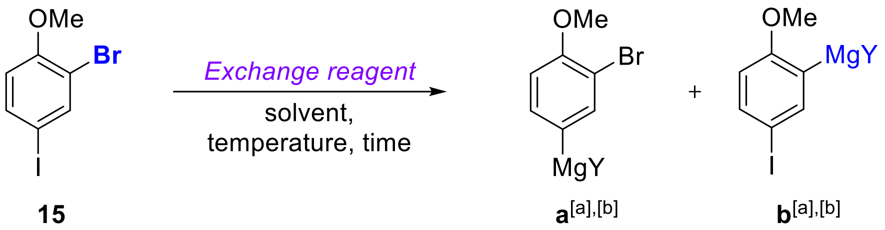

Entry	Exchange reagent[c]	Time (min)	Ratio **a**:**b**[d]	Conv. [%][e]
1	*i*PrMgCl·LiCl (**1a**)	60	99:1	81[a]
2	*s*Bu_2Mg·2LiOR (**1c**)	30	24:76	99[b]
3[f]	*n*Bu_2Mg·2LiOR (**1d**)	30	20:80	99[b] (71)
4	**1d·PMDTA**	30	99:1	99[b]

[a] Y = Cl·LiCl. [b] Y = anisyl·2LiOR(·ligand). [c] R = 2-ethylhexyl, reactions were carried out at 0.05 M and −10 °C using 1.2 equiv of alkylmagnesium species. Reagents are displayed accordingly to their stoichiometry and not their actual structure. [d] A mixture of 2-bromoanisole and 4-bromoanisole was obtained by halogen dance when the reactions were done at 25 °C. An increase in concentration (0.5 M in toluene) hampered the selectivity. [e] Conversion determined by GC-analysis of reaction aliquots after aqueous quench. Isolated yield in parenthesis. [f] A regioselectivity of **a**:**b** = 80:20 was observed using 2.4 equiv of 12-crown-4 as an additive.

Scheme S1. Selective Br/Mg-exchange with 2-bromo-4-iodoanisole (**15**) followed by allylation[a] reaction: comparison with *i*PrMgCl·LiCl.

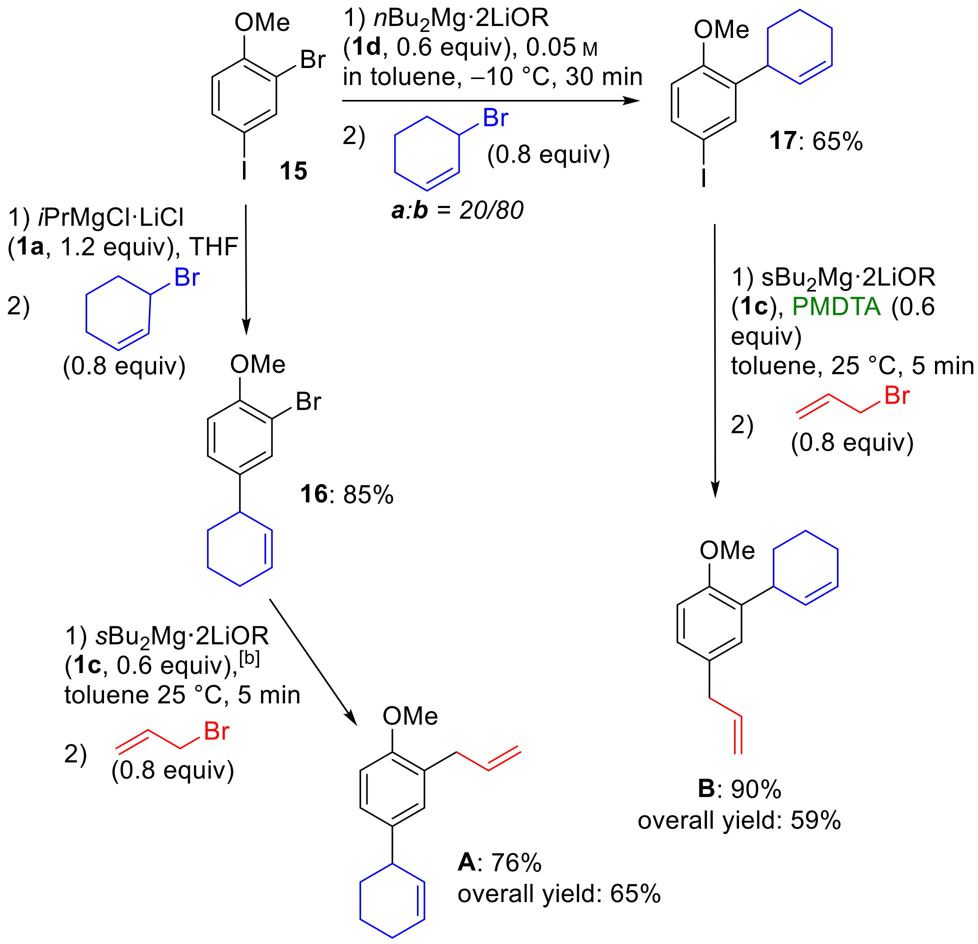

[a] CuCN·2LiCl (10 mol%) was used. [b] After 33 h of reaction at 25 °C, 67% of **16** remained when the exchange was carried out with *i*PrMgCl·LiCl (**1a**, 1.2 equiv) and **1c** had to be employed.

Scheme S2. Reaction of 2-bromo-4-iodo-5-methylanisole (**SM1**) and 2-bromo-4-iodo-5-isopropylanisole (**SM4**) with *n*Bu_2Mg·2LiOR (**1d**), followed by electrophilic functionalization.

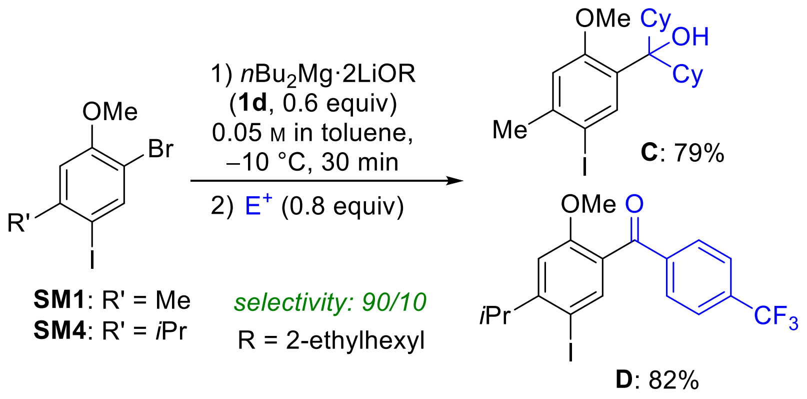

The experimental data can be found on pages SI-45‑SI-51 and SI-123‑SI-131.

Preparation of Compounds 5, 9, 13-14, 16-17, SM1-4 and A-D

Synthesis of 2-allyl-4-bromo-1-methoxybenzene (5a)

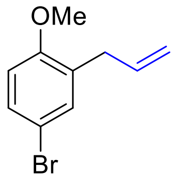

Compound **5a** was prepared *via* **TP1** using 2,4-dibromoanisole (**2a**, 133 mg, 0.50 mmol) and dry toluene (1.0 mL). Then, *s*Bu_2Mg·2LiOR (**1c**, 0.36 mL, 0.30 mmol) was added at 25 °C. After stirring at 25 °C for 5 min, CuCN·2LiCl (1.00 M in THF, 50 µL, 50 µmol) and allyl bromide (34 µL, 0.40 mmol) were added at 0 °C and the reaction mixture was allowed to warm to room temperature overnight. After work-up, the crude product was purified *via* column chromatography (*iso*hexane, R_f = 0.43) to give the product **5a** (65 mg, 286 µmol, 72% yield) as a colorless oil.

^{1}H-NMR (400 MHz, $CDCl_3$, ppm): δ = 7.29 (dd, *J* = 8.6, 2.6 Hz, 1H), 7.26 – 7.21 (m, 1H), 6.72 (d, *J* = 8.6 Hz, 1H), 5.95 (ddt, *J* = 15.7, 11.2, 6.6 Hz, 1H), 5.09 (s, 1H), 5.06 (dt, *J* = 5.6, 1.8 Hz, 1H), 3.81 (s, 3H), 3.34 (dt, *J* = 6.6, 1.6 Hz, 2H).
^{13}C-NMR (101 MHz, $CDCl_3$, ppm): δ = 156.5, 136.1, 132.5, 131.1, 130.0, 116.3, 112.8, 112.0, 55.7, 34.0.
IR (ATR, cm^{-1}) ṽ = 2938, 2836, 1638, 1592, 1488, 1463, 1440, 1432, 1401, 1322, 1304, 1278, 1243, 1172, 1135, 1127, 1032, 996, 917, 861, 804.
MS (EI, 70 eV, %) m/z = 228 (45), 226 (46), 199 (24), 197 (26), 148 (11), 147 (100), 132 (84), 131 (70), 119 (17), 118 (91), 117 (12), 115 (36), 104 (11), 103 (18), 91 (73), 90 (12), 89 (13), 77 (12).
HRMS (EI, 70 eV) m/z: calc. for **$C_{10}H_{11}BrO$**: 225.9993; found: 225.9978.

Synthesis of (5-bromo-2-(2-methoxyethoxy)phenyl)(methyl)sulfane (5b)

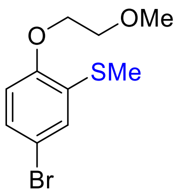

Compound **5b** was prepared *via* **TP1** using 2,4-dibromo-1-(2-methoxyethoxy)benzene (**2b**, 155 mg, 0.50 mmol) and dry toluene (1.0 mL). Then, *s*Bu_2Mg·2LiOR (**1c**, 0.36 mL, 0.30 mmol) was added at 25 °C. After stirring at 25 °C for 5 min, *S*-methyl methanesulfonothioate (51 mg, 0.40 mmol) was added and the reaction mixture was allowed to stir at room temperature overnight. After work-up, the crude product was purified *via* column chromatography (*iso*hexane:ethyl acetate = 9:1, R_f = 0.44) to give the product **5b** (86 mg, 310 µmol, 78% yield) as a colorless oil.

^{1}H-NMR (400 MHz, $CDCl_3$, ppm): δ = 7.23 – 7.11 (m, 2H), 6.70 (d, *J* = 8.2 Hz, 1H), 4.18 – 4.11 (m, 2H), 3.90 – 3.68 (m, 2H), 3.46 (s, 3H), 2.41 (s, 3H).
^{13}C-NMR (101 MHz, $CDCl_3$, ppm): δ = 154.6, 130.6, 128.2, 128.0, 114.0, 113.1, 71.0, 68.8, 59.6, 14.6.
IR (ATR, cm^{-1}) ṽ = 2923, 2879, 1573, 1472, 1450, 1438, 1382, 1368, 1300, 1266, 1238, 1198, 1128, 1087, 1074, 1050, 1033, 924, 854, 799, 733, 713.
MS (EI, 70 eV, %) m/z = 278 (20), 276 (20), 220 (100), 218 (99), 205 (16), 203 (16), 138 (14).
HRMS (EI, 70 eV) m/z: calc. for **$C_{10}H_{13}BrO_2S$**: 275.9820; found: 275.9813.

Synthesis of 1-(2-(allyloxy)-5-bromophenyl)ethan-1-one (5c)

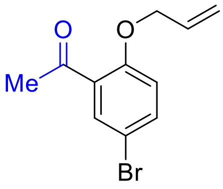

Compound **5c** was prepared *via* **TP1** using 1-(allyloxy)-2,4-dibromobenzene (**2c**, 146 mg, 0.50 mmol) and dry toluene (1.0 mL). Then, *s*Bu_2Mg·2LiOR (**1c**, 0.36 mL, 0.30 mmol) was added at 25 °C. After stirring at 25 °C for 5 min, *N*-methoxy-*N*-methylacetamide (41 mg, 0.40

mmol) was added and the reaction mixture was allowed to warm to room temperature overnight. After work-up, the crude product was purified *via* column chromatography (*iso*hexane:ethyl acetate = 98:2, R_f = 0.27) to give the product **5c** (65 mg, 255 µmol, 64% yield) as a white solid.

M.p. (°C): 56-58.
^{1}H-NMR (400 MHz, CDCl$_3$, ppm): δ = 7.83 (d, *J* = 2.6 Hz, 1H), 7.51 (dd, *J* = 8.8, 2.6 Hz, 1H), 6.84 (d, *J* = 8.8 Hz, 1H), 6.06 (ddt, *J* = 17.3, 10.6, 5.4 Hz, 1H), 5.42 (dq, *J* = 17.3, 1.5 Hz, 1H), 5.34 (dq, *J* = 10.5, 1.3 Hz, 1H), 4.62 (dt, *J* = 5.3, 1.5 Hz, 2H), 2.62 (s, 3H).
^{13}C-NMR (101 MHz, CDCl$_3$, ppm): δ = 198.5, 157.0, 136.1, 133.2, 132.3, 130.2, 118.8, 114.9, 113.5, 69.9, 32.0.
IR (ATR, cm^{-1}) ṽ = 3093, 2931, 1660, 1628, 1588, 1566, 1481, 1460, 1423, 1409, 1398, 1369, 1352, 1296, 1280, 1233, 1223, 1153, 1064, 1021, 1002, 992, 976, 931, 906, 816, 655.
MS (EI, 70 eV, %) m/z = 241 (38), 239 (41), 213 (20), 211 (21), 201 (96), 199 (100), 160 (16), 132 (32), 131 (17), 129 (15), 78 (15).
HRMS (EI, 70 eV) m/z: calc. for **$C_{11}H_{11}BrO_2$**: 253.9942; found: 253.9937.

Synthesis of 1,5-dibromo-2-methoxy-3-(2-methylallyl)benzene (5d)

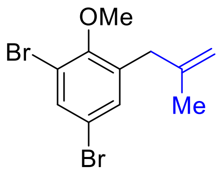

Compound **5d** was prepared *via* **TP1** using 2,4,6-tribromoanisole (**2d**, 172 mg, 0.50 mmol) and dry toluene (1.0 mL). Then, *s*Bu$_2$Mg·2LiOR (**1c**, 0.36 mL, 0.30 mmol) was added at 25 °C. After stirring at 25 °C for 5 min, CuCN·2LiCl (1.00 M in THF, 50 µL, 50 µmol) and 3-bromo-2-methylprop-1-ene (40 µL, 0.40 mmol) were added at 0 °C and the reaction solution was allowed to warm to room temperature overnight. After work-up, the crude product was purified *via* column chromatography (*iso*hexane, R_f = 0.39) to give the product **5d** (111 mg, 347 µmol, 87% yield) as a colorless oil.

^{1}H-NMR (400 MHz, CDCl$_3$, ppm): δ = 7.56 (d, *J* = 2.4 Hz, 1H), 7.31 – 7.14 (m, 1H), 4.89 (s, 1H), 4.67 (s, 1H), 3.79 (s, 3H), 3.35 (s, 2H), 1.72 (s, 3H).

^{13}C-NMR (101 MHz, CDCl$_3$, ppm): δ = 154.9, 143.7, 136.8, 133.9, 132.8, 118.4, 117.2, 113.2, 61.2, 38.2, 22.6.

IR (ATR, cm^{-1}) ṽ = 2938, 1652, 1576, 1553, 1461, 1438, 1417, 1393, 1375, 1276, 1251, 1220, 1147, 1001, 895, 859, 810, 805, 753, 667.

MS (EI, 70 eV, %) m/z = 322 (15), 320 (32), 318 (17), 241 (12), 239 (13), 226 (34), 225 (13), 224 (31), 212 (11), 211 (21), 210 (12), 209 (22), 198 (10), 196 (12), 161 (10), 160 (69), 159 (19), 155 (14), 146 (12), 145 (100), 144 (11), 129 (21), 128 (31), 117 (28), 116 (11), 115 (69), 102 (14), 91 (18), 89 (26), 76 (10), 75 (16).

HRMS (EI, 70 eV) m/z: calc. for **$C_{11}H_{12}Br_2O$**: 317.9255; found: 317.9243.

Synthesis of 5-bromo-2-methoxy-3-(2-methylallyl)pyridine (5ea)

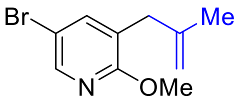

Compound **5ea** was prepared *via* **TP1** using 3,5-dibromo-2-methoxypyridine (**2e**, 133 mg, 0.50 mmol) and dry toluene (1.0 mL). Then, *s*Bu$_2$Mg·2LiOR (**1c**, 0.36 mL, 0.30 mmol) was added at 25 °C. After stirring at 25 °C for 5 min, CuCN·2LiCl (1.00 M in THF, 50 µL, 50 µmol) and 3-bromo-2-methylprop-1-ene (40 µL, 0.40 mmol) were added at 0 °C and the reaction solution was allowed to warm to room temperature overnight. After work-up, the crude product was purified *via* column chromatography (*iso*hexane:ethyl acetate = 96:4, R$_f$ = 0.50) to give the product **5ea** (78 mg, 322 µmol, 81% yield) as a colorless oil.

^{1}H-NMR (400 MHz, CDCl$_3$, ppm): δ = 8.06 (d, *J* = 2.5 Hz, 1H), 7.47 (dt, *J* = 2.6, 0.7 Hz, 1H), 4.95 – 4.79 (m, 1H), 4.67 (dd, *J* = 2.1, 1.0 Hz, 1H), 3.91 (s, 3H), 3.24 (s, 2H), 1.71 (s, 3H).

^{13}C-NMR (101 MHz, CDCl$_3$, ppm): δ = 161.3, 145.1, 142.9, 140.5, 124.7, 113.0, 111.8, 53.9, 37.4, 22.5.

IR (ATR, cm^{-1}) ṽ = 2976, 2950, 1653, 1578, 1569, 1560, 1465, 1414, 1390, 1376, 1308, 1247, 1154, 1138, 1020, 893, 752.

MS (EI, 70 eV, %) m/z = 243 (99), 242 (59), 241 (100), 240 (61), 228 (78), 226 (79), 214 (50), 212 (71), 210 (74), 200 (65), 198 (96), 196 (30), 188 (72), 186 (74), 172 (28), 170 (27), 147 (54), 146 (86), 130 (42), 129 (32), 119 (72), 118 (70), 117 (28), 91 (27).

HRMS (EI, 70 eV) m/z: calc. for **$C_{10}H_{12}BrNO$**: 241.0102; found: 241.0097.

Synthesis of (5-bromo-2-methoxypyridin-3-yl)(4-chlorophenyl)(cyclopropyl)methanol (5eb)

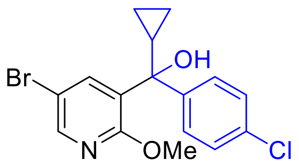

Compound **5eb** was prepared *via* **TP1** using 3,5-dibromo-2-methoxypyridine (**2e**, 133 mg, 0.50 mmol) and dry toluene (1.0 mL). Then, *s*Bu_2Mg·2LiOR (**1c**, 0.36 mL, 0.30 mmol) was added at 25 °C. After stirring at 25 °C for 5 min, (4-chlorophenyl)(cyclopropyl)methanone (72 mg, 0.40 mmol) was added and the reaction solution was allowed to warm to room temperature overnight. After work-up, the crude product was purified *via* column chromatography (*iso*hexane:ethyl acetate = 95:5, R_f = 0.28) to give the product **5eb** (100 mg, 271 µmol, 68% yield) as a colorless oil.

^{1}H-NMR (400 MHz, $CDCl_3$, ppm): δ = 8.16 (q, *J* = 2.4 Hz, 2H), 7.44 – 6.93 (m, 4H), 3.87 (d, *J* = 1.0 Hz, 1H), 3.73 (s, 3H), 1.45 (ddtd, *J* = 8.2, 6.5, 5.5, 1.1 Hz, 1H), 0.66 – 0.50 (m, 3H), 0.46 (ddt, *J* = 7.9, 5.4, 2.9 Hz, 1H).

^{13}C-NMR (101 MHz, $CDCl_3$, ppm): δ = 159.7, 146.4, 144.6, 138.8, 133.0, 131.0, 128.1, 127.2, 112.4, 75.3, 54.0, 20.3, 1.9, 1.6.

IR (ATR, cm^{-1}) ṽ = 3546, 3011, 2952, 1578, 1564, 1489, 1461, 1409, 1381, 1331, 1294, 1242, 1202, 1175, 1154, 1146, 1120, 1105, 1092, 1014, 985, 970, 942, 927, 899, 882, 871, 833, 806, 758, 734, 720, 681.

MS (EI, 70 eV, %) m/z = 343 (10), 341 (42), 339 (33), 216 (98), 214 (100), 139 (21).

HRMS (EI, 70 eV) m/z: calc. for **$C_{16}H_{15}BrClNO_2$**: 366.9975; found: 366.9971.

Synthesis of 4-(5-bromo-2-methoxypyridin-3-yl)benzonitrile (5ec)

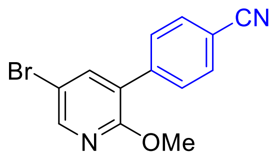

Compound **5ec** was prepared *via* **TP1** using 3,5-dibromo-2-methoxypyridine (**2e**, 133 mg, 0.50 mmol) and dry toluene (1.0 mL). Then, *s*Bu_2Mg·2LiOR (**1c**, 0.36 mL, 0.30 mmol) was added at 25 °C. After stirring at 25 °C for 5 min, $ZnCl_2$ (1.00 M in THF, 0.65 mL, 0.65 mmol), $Pd(OAc)_2$ (5 mg, 4 mol%), SPhos (17 mg, 8 mol%) and 4-iodobenzonitrile (92 mg, 0.40 mmol) were added and the reaction mixture was stirred at room temperature overnight. After work-up, the crude product was purified *via* column chromatography (*iso*hexane:ethyl acetate = 9:1, R_f = 0.74) to give the product **5ec** (61 mg, 211 µmol, 53% yield) as a white solid.

M.p. (°C): 155-157.
^{1}H-NMR (400 MHz, $CDCl_3$, ppm): δ = 8.25 (d, *J* = 2.4 Hz, 1H), 7.75 – 7.70 (m, 3H), 7.68 – 7.62 (m, 2H), 3.96 (s, 3H).
^{13}C-NMR (101 MHz, $CDCl_3$, ppm): δ = 159.6, 147.6, 140.9, 140.2, 132.3, 130.0, 124.4, 118.8, 112.2, 111.9, 54.3.
IR (ATR, cm^{-1}) ṽ = 2955, 2226, 1742, 1608, 1566, 1463, 1415, 1395, 1299, 1245, 1220, 1032, 1017, 1006, 843, 772.
MS (EI, 70 eV, %) m/z = 290 (30), 289 (100), 288 (32), 287 (99), 273 (14), 271 (30), 269 (15), 259 (12), 208 (15), 207 (13), 194 (22), 180 (50), 179 (54), 178 (13), 166 (30), 165 (29), 152 (20), 151 (20), 140 (16), 139 (58), 138 (34), 125 (14), 88 (13), 86 (15).
HRMS (EI, 70 eV) m/z: calc. for **$C_{13}H_9BrN_2O$**: 287.9898; found: 287.9901.

Synthesis of 5-bromo-3-chloro-2-(methylthio)pyridine (5f)

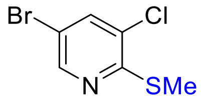

Compound **5f** was prepared *via* **TP1** using 2,5-dibromo-3-chloropyridine (**2f**, 136 mg, 0.50 mmol) and dry toluene (1.0 mL). Then, *s*Bu_2Mg·2LiOR (**1c**, 0.36 mL, 0.30 mmol) was

added at -20 °C. After stirring at -20 °C for 30 min, *S*-methyl methanesulfonothioate (51 mg, 0.40 mmol) was added and the reaction solution was allowed to warm to room temperature overnight. After work-up, the crude product was purified *via* column chromatography (*iso*hexane, R_f = 0.31) to give the product **5f** (57 mg, 239 µmol, 60% yield) as a white solid.

M.p. (°C): 65-67.

^{1}H-NMR (400 MHz, $CDCl_3$, ppm): δ = 8.41 (d, *J* = 2.1 Hz, 1H), 7.68 (d, *J* = 2.0 Hz, 1H), 2.53 (s, 3H).

^{13}C-NMR (101 MHz, $CDCl_3$, ppm): δ = 157.1, 148.0, 137.7, 129.4, 114.8, 13.7.

IR (ATR, cm^{-1}) ṽ = 3037, 2924, 1548, 1415, 1351, 1220, 1210, 1150, 1105, 1036, 900, 894, 812, 718.

MS (EI, 70 eV, %) m/z = 241 (18), 239 (72), 238 (30), 237 (56), 236 (24), 208 (13), 206 (54), 204 (100), 202 (98), 194 (12), 193 (18), 191 (14), 125 (14), 112 (26).

HRMS (EI, 70 eV) m/z: calc. for **$C_6H_5BrClNS$**: 236.9015; found: 236.9010.

Synthesis of (5-bromo-3-fluoropyridin-2-yl)(thiophen-2-yl)methanone (5g)

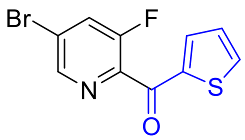

Compound **5g** was prepared *via* **TP1** using 2,5-dibromo-3-fluoropyridine (**2g**, 128 mg, 0.50 mmol) and dry toluene (1.0 mL). Then, *s*Bu_2Mg·2LiOR (**1c**, 0.36 mL, 0.30 mmol) was added at -20 °C. After stirring at -20 °C for 30 min, *N*-methoxy-*N*-methylthiophene-2-carboxamide (69 mg, 0.40 mmol) was added and the reaction solution was allowed to warm to room temperature overnight. After work-up, the crude product was purified *via* column chromatography (*iso*hexane:ethyl acetate = 97:3, R_f = 0.20) to give the product **5g** (75 mg, 262 µmol, 66% yield) as a yellow solid.

M.p. (°C): 134-136.

^{1}H-NMR (400 MHz, $CDCl_3$, ppm): δ = 8.60 (dd, *J* = 1.8, 0.9 Hz, 1H), 7.98 (dd, *J* = 3.9, 1.2 Hz, 1H), 7.88 – 7.70 (m, 2H), 7.17 (dd, *J* = 4.9, 3.9 Hz, 1H).

^{13}C-NMR (101 MHz, $CDCl_3$, ppm): δ = 181.3 (d, *J* = 4.6 Hz), 158.0 (d, *J* = 277.1 Hz), 145.6 (d, *J* = 5.3 Hz), 141.5 (d, *J* = 8.4 Hz), 141.2, 136.7, 136.5, 128.6 (d, *J* = 21.4 Hz), 128.2, 123.7 (d, *J* = 3.4 Hz).

^{19}F-NMR (377 MHz, $CDCl_3$, ppm): δ = -117.6.

IR (ATR, cm^{-1}) ṽ = 3109, 3097, 3068, 2924, 1656, 1634, 1572, 1514, 1442, 1436, 1408, 1396, 1353, 1307, 1234, 1218, 1199, 1134, 1082, 1050, 909, 882, 868, 862, 826, 782, 729, 716, 679, 660.

MS (EI, 70 eV, %) m/z = 259 (62), 257 (60), 111 (100).

HRMS (EI, 70 eV) m/z: calc. for **$C_{10}H_5BrFNOS$**: 284.9259; found: 284.9250.

Synthesis of (5-bromo-2-methoxyphenyl)(2-fluorophenyl)methanone (5aa)

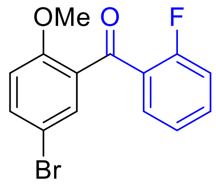

Compound **5aa** was prepared *via* **TP1** using 2,4-dibromoanisole (**2a**, 266 mg, 1.00 mmol) and dry toluene (2.0 mL). Then, *s*Bu_2Mg·2LiOR (**1c**, 0.76 mL, 0.60 mmol) was added at 25 °C. After stirring at 25 °C for 5 min, CuCN·2LiCl (1.00 M in THF, 100 µL, 100 µmol) and 2-fluorobenzoyl chloride (0.36 mL, 3.00 mmol) were added at 0 °C and the reaction solution was allowed to warm to room temperature overnight. After work-up, the crude product was purified *via* column chromatography (*iso*hexane:ethyl acetate = 95:5, R_f = 0.18) to give the product **5aa** (233 mg, 754 µmol, 75% yield) as a white solid.

M.p. (°C): 57-59.

^{1}H-NMR (400 MHz, $CDCl_3$, ppm): δ = 7.71 (td, *J* = 7.5, 1.9 Hz, 1H), 7.64 (d, *J* = 2.6 Hz, 1H), 7.57 (dd, *J* = 8.8, 2.6 Hz, 1H), 7.51 (dddd, *J* = 8.3, 7.2, 5.0, 1.9 Hz, 1H), 7.23 (td, *J* = 7.5, 1.1 Hz, 1H), 7.06 (ddd, *J* = 10.7, 8.3, 1.1 Hz, 1H), 6.84 (d, *J* = 8.8 Hz, 1H), 3.66 (s, 3H).

^{13}C-NMR (101 MHz, $CDCl_3$, ppm): δ = 191.1, 161.3 (d, *J* = 255.5 Hz), 157.5 (d, *J* = 1.7 Hz), 135.7, 134.1 (d, *J* = 8.8 Hz), 132.8 (d, *J* = 0.7 Hz), 131.4 (d, *J* = 1.2 Hz), 131.0 (d, *J* = 2.1 Hz), 127.8 (d, *J* = 11.8 Hz), 124.3 (d, *J* = 3.6 Hz), 116.1 (d, *J* = 22.3 Hz), 113.5, 113.1, 56.0.

^{19}F-NMR (377 MHz, $CDCl_3$, ppm): δ = -112.3.

IR (ATR, cm^{-1}) ṽ = 2939, 1655, 1610, 1591, 1576, 1482, 1454, 1396, 1301, 1271, 1255, 1238, 1215, 1181, 1162, 1152, 1126, 1101, 1023, 954, 832, 813, 790, 758.

MS (EI, 70 eV, %) m/z = 310 (37), 308 (39), 293 (40), 291 (29), 215 (72), 213 (92), 212 (91), 201 (71), 199 (76), 123 (100).

HRMS (EI, 70 eV) m/z: calc. for $\mathbf{C_{14}H_{10}BrFO_2}$: 307.9848; found: 307.9843.

Synthesis of 2-bromo-9*H*-xanthen-9-one (5ab)

Step 1: Synthesis of (5-bromo-2-hydroxyphenyl)(2-fluorophenyl)methanone[11]

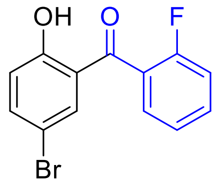

A dry and argon-flushed *Schlenk*-flask, equipped with a magnetic stirring bar and a septum, was charged with (5-bromo-2-methoxyphenyl)(2-fluorophenyl)methanone (**5aa,** 106 mg, 0.34 mmol) and dissolved in dry DCM (1.0 mL). Then, BBr_3 (1.00 M in DCM, 0.68 mL, 0.68 mmol) was added dropwise at -78 °C and the reaction solution was allowed to warm to room temperature overnight. The mixture was quenched with water (10 mL) and extracted with ether (3 x 30 mL). The combined organic extracts were dried over Na_2SO_4, filtered and concentrated *in vacuo*. The crude product was purified *via* column chromatography (*iso*hexane:ethyl acetate = 95:5, R_f = 0.44) to give the product (5-bromo-2-hydroxyphenyl)(2-fluorophenyl)methanone (98 mg, 332 µmol, 98% yield) as a yellow solid.

M.p. (°C): 88-90.

^{1}H-NMR (400 MHz, $CDCl_3$, ppm): δ = 11.89 (s, 1H), 7.63 – 7.54 (m, 2H), 7.51 (t, *J* = 2.7 Hz, 1H), 7.47 (ddd, *J* = 8.5, 6.6, 1.8 Hz, 1H), 7.32 (td, *J* = 7.5, 1.0 Hz, 1H), 7.23 (ddd, *J* = 9.5, 8.4, 1.0 Hz, 1H), 6.98 (d, *J* = 8.9 Hz, 1H).

^{13}C-NMR (101 MHz, $CDCl_3$, ppm): δ = 197.8, 162.1, 159.3 (d, *J* = 252.2 Hz), 139.8, 135.3 (d, *J* = 2.6 Hz), 133.6 (d, *J* = 8.3 Hz), 130.0 (d, *J* = 2.7 Hz), 125.8 (d, *J* = 15.5 Hz), 124.7 (d, *J* = 3.6 Hz), 121.1, 120.6, 116.7 (d, *J* = 21.3 Hz), 110.8.

^{19}F-NMR (377 MHz, $CDCl_3$, ppm): δ = -111.9.

IR (ATR, cm^{-1}) ṽ = 3072, 1629, 1613, 1583, 1485, 1464, 1452, 1400, 1330, 1293, 1268, 1238, 1212, 1159, 1149, 1122, 1100, 1084, 951, 944, 838, 829, 816, 792, 759, 720.

MS (EI, 70 eV, %) m/z = 296 (37), 295 (96), 294 (39), 293 (100), 277 (37), 276 (31), 275 (32), 274 (32), 201 (34), 200 (47), 199 (35), 198 (42), 172 (28), 170 (29), 145 (17), 143 (18), 123 (72).

---

[11] Adapted procedure from: J. F. W. McOmie, D. E. West, *Org. Synth.* **1969**, *49*, 50.

HRMS (EI, 70 eV) m/z: calc. for **$C_{13}H_8BrFO_2$**: 293.9692; found: 293.9693.

Step 2: Synthesis of 2-bromo-9*H*-xanthen-9-one (**5ab**)[12]

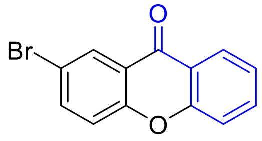

A dry and argon-flushed *Schlenk*-flask, equipped with a magnetic stirring bar and a septum, was charged with (5-bromo-2-hydroxyphenyl)(2-fluorophenyl)methanone (88 mg, 0.30 mmol), K_2CO_3 (83 mg, 0.60 mmol) and dry acetone (3.0 mL). Then, the tube was sealed and stirred at 50 °C for 4 h. The mixture was quenched with water (10 mL) and extracted with ether (3 x 30 mL). The combined organic extracts were dried over Na_2SO_4, filtered and concentrated *in vacuo* to give the product **5ab** (81 mg, 294 µmol, 98% yield) as an off-white solid.

M.p. (°C): 146-148.
^{1}H-NMR (400 MHz, CDCl$_3$, ppm): δ = 8.45 (d, *J* = 2.4 Hz, 1H), 8.33 (dd, *J* = 8.0, 1.8 Hz, 1H), 7.80 (dd, *J* = 8.9, 2.5 Hz, 1H), 7.75 (ddd, *J* = 8.7, 7.1, 1.8 Hz, 1H), 7.50 (dd, *J* = 8.5, 1.1 Hz, 1H), 7.44 – 7.36 (m, 2H).
^{13}C-NMR (101 MHz, CDCl$_3$, ppm): δ = 176.2, 156.2, 155.1, 137.8, 135.4, 129.4, 127.0, 124.4, 123.3, 121.7, 120.2, 118.2, 117.2.
IR (ATR, cm^{-1}) ṽ = 3077, 2925, 1663, 1616, 1606, 1472, 1458, 1439, 1424, 1337, 1315, 1266, 1216, 1170, 1153, 1132, 1108, 882, 843, 824, 756, 718, 680, 672.
MS (EI, 70 eV, %) m/z = 277 (14), 276 (98), 275 (17), 274 (100), 248 (19), 246 (20), 195 (27), 139 (78).
HRMS (EI, 70 eV) m/z: calc. for **$C_{13}H_7BrO_2$**: 273.9629; found: 273.9625.

[12] Adapted procedure from: C. Zhou, R. C. Larock, *J. Org. Chem.* **2006**, *71*, 3551-3558.

Synthesis of (5-bromo-4-methylthiophen-2-yl)(3-methoxyphenyl)methanol (9a)

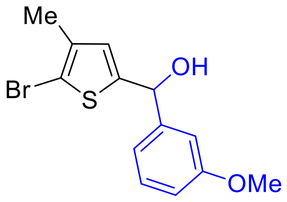

Compound **9a** was prepared *via* **TP1** using 2,5-dibromo-3-methylthiophene (**6a**, 128 mg, 0.50 mmol), dry toluene (1.0 mL) and PMDTA (63 µL, 0.30 mmol). Then, *s*Bu_2Mg·2LiOR (**1c**, 0.36 mL, 0.30 mmol) was added at 25 °C. After stirring at 25 °C for 5 min, 3-methoxybenzaldehyde (55 mg, 0.40 mmol) was added and the reaction solution was allowed to stir at room temperature overnight. After work-up, the crude product was purified *via* column chromatography (*iso*hexane:ethyl acetate = 9:1, R_f = 0.25) to give the product **9a** (100 mg, 319 µmol, 80% yield) as a yellowish oil.

^{1}H-NMR (400 MHz, $CDCl_3$, ppm): δ = 7.36 – 7.19 (m, 1H), 7.05 – 6.94 (m, 2H), 6.85 (ddd, *J* = 8.3, 2.5, 1.1 Hz, 1H), 6.61 – 6.47 (m, 1H), 5.86 (d, *J* = 3.7 Hz, 1H), 3.81 (s, 3H), 2.39 (dd, *J* = 3.9, 0.9 Hz, 1H), 2.10 (s, 3H).

^{13}C-NMR (101 MHz, $CDCl_3$, ppm): δ = 159.9, 147.1, 144.2, 136.9, 129.9, 126.9, 118.7, 113.8, 111.8, 109.5, 72.6, 55.4, 15.4.

IR (ATR, cm^{-1}) ṽ = 2975, 1608, 1600, 1587, 1489, 1464, 1455, 1436, 1316, 1281, 1263, 1256, 1156, 1131, 1094, 1082, 1050, 1020, 765.

MS (EI, 70 eV, %) m/z = 314 (18), 312 (18), 234 (13), 233 (100), 207 (14), 205 (37), 203 (23), 201 (14), 190 (11), 178 (14), 176 (14), 173 (11), 172 (83), 157 (15), 135 (83), 129 (16), 128 (17), 125 (17), 115 (11), 109 (16), 98 (24), 97 (35), 94 (11), 92 (12), 77 (20).

HRMS (EI, 70 eV) m/z: calc. for **$C_{13}H_{13}BrO_2S$**: 311.9820; found: 311.9813.

Synthesis of (3,5-dibromo-4-methylthiophen-2-yl)bis(4-(dimethylamino)phenyl)methanol (9b)

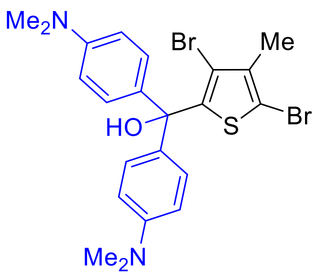

Compound **9b** was prepared *via* **TP1** using 2,4,5-tribromo-3-methylthiophene (**6b**, 167 mg, 0.50 mmol), dry toluene (1.0 mL) and PMDTA (63 µL, 0.30 mmol). Then, $s\mathrm{Bu_2Mg\cdot 2LiOR}$ (**1c**, 0.36 mL, 0.30 mmol) was added at 25 °C. After stirring at 25 °C for 5 min, bis(4-(dimethylamino)phenyl)methanone (107 mg, 0.40 mmol) was added and the reaction solution was allowed to stir at room temperature overnight. After work-up, the crude product was purified *via* column chromatography (*iso*hexane:ethyl acetate = 9:1, R_f = 0.41) to give the product **9b** (128 mg, 244 µmol, 61% yield) as a blue oil.

^{1}H-NMR (400 MHz, $CDCl_3$, ppm): δ = 7.22 – 7.10 (m, 4H), 6.73 – 6.52 (m, 4H), 3.73 (s, 1H), 2.95 (s, 12H), 2.18 (s, 3H).

^{13}C-NMR (101 MHz, $CDCl_3$, ppm): δ = 150.0, 147.4, 137.8, 133.0, 128.7, 111.5, 110.0, 108.3, 79.7, 40.6, 16.2.

IR (ATR, cm^{-1}) ṽ = 2918, 1610, 1585, 1543, 1518, 1481, 1443, 1369, 1321, 1286, 1269, 1228, 1188, 1171, 1129, 1115, 1063, 1022, 945, 819, 754.

MS (EI, 70 eV, %) m/z = 526 (39), 525 (17), 524 (63), 523 (11), 522 (35), 510 (21), 509 (49), 508 (43), 507 (84), 506 (23), 505 (44), 444 (23), 442 (17), 430 (16), 429 (21), 428 (16), 427 (18), 404 (14), 364 (11), 349 (10), 348 (12), 347 (20), 331 (11), 308 (16), 306 (12), 283 (16), 270 (18), 269 (74), 268 (26), 253 (16), 241 (10), 149 (10), 148 (100), 121 (18), 120 (16), 44 (30).

HRMS (EI, 70 eV) m/z: calc. for **$C_{22}H_{24}Br_2N_2OS$**: 521.9976; found: 521.9969.

Synthesis of (5-allyl-2-bromophenyl)trimethylsilane (9c)

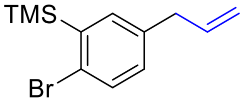

Compound **9c** was prepared *via* **TP1** using (2,5-dibromophenyl)trimethylsilane (**6c**, 154 mg, 0.50 mmol), dry toluene (1.0 mL) and PMDTA (63 µL, 0.30 mmol). Then, *s*Bu_2Mg·2LiOR (**1c**, 0.36 mL, 0.30 mmol) was added at -20 °C. After stirring at -20 °C for 30 min, CuCN·2LiCl (1.00 M in THF, 50 µL, 50 µmol) and allyl bromide (34 µL, 0.40 mmol) were added and the reaction mixture was allowed to warm to room temperature overnight. After work-up, the crude product was purified *via* column chromatography (*iso*hexane, R_f = 0.80) to give the product **9c** (89 mg, 331 µmol, 83% yield) as a colorless oil.

^{1}H-NMR (400 MHz, $CDCl_3$, ppm): δ = 7.45 (d, *J* = 8.1 Hz, 1H), 7.24 (d, *J* = 2.4 Hz, 1H), 7.03 (dd, *J* = 8.1, 2.4 Hz, 1H), 6.03 – 5.84 (m, 1H), 5.17 – 5.02 (m, 2H), 3.34 (dt, *J* = 6.8, 1.5 Hz, 2H), 0.39 (s, 9H).
^{13}C-NMR (101 MHz, $CDCl_3$, ppm): δ = 141.1, 138.3, 137.1, 136.5, 132.8, 131.2, 128.1, 116.3, 39.8, -0.4.
IR (ATR, cm^{-1}) ṽ = 2954, 2899, 1456, 1447, 1436, 1380, 1262, 1250, 1140, 1109, 1016, 992, 914, 871, 839, 814, 761, 690.
MS (EI, 70 eV, %) m/z = 273 (12), 271 (12), 268 (10), 256 (10), 255 (100), 254 (10), 253 (99), 191 (11), 173 (44), 171 (13), 163 (29), 145 (86), 139 (10), 137 (11), 133 (10), 131 (27), 129 (34), 128 (10), 115 (13), 91 (12), 75 (10).
HRMS (EI, 70 eV) m/z: calc. for **$C_{12}H_{17}BrSi$**: 268.0283; found: 268.0277.

Synthesis of 4'-bromo-3'-methyl-1,2,3,4-tetrahydro-1,1'-biphenyl (9d)

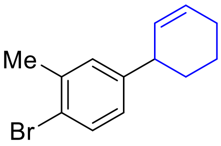

Compound **9d** was prepared *via* **TP1** using 1,4-dibromo-2-methylbenzene (**6d**, 125 mg, 0.50 mmol), dry toluene (1.0 mL) and PMDTA (63 µL, 0.30 mmol). Then, *s*Bu_2Mg·2LiOR (**1c**,

0.36 mL, 0.30 mmol) was added at 25 °C. After stirring at 25 °C for 5 min, CuCN·2LiCl (1.00 M in THF, 50 µL, 50 µmol) and 3-bromocyclohexene (46 µL, 0.40 mmol) were added at 0 °C and the reaction mixture was allowed to warm to room temperature overnight. After work-up, the crude product was purified *via* column chromatography (*iso*hexane, R_f = 0.71) to give the product **9d** (66 mg, 263 µmol, 66% yield) as a colorless oil.

^{1}H-NMR (400 MHz, $CDCl_3$, ppm): δ = 7.44 (d, *J* = 8.2 Hz, 1H), 7.08 (d, *J* = 2.2 Hz, 1H), 6.90 (dd, *J* = 8.2, 2.3 Hz, 1H), 5.90 (dtd, *J* = 9.8, 3.7, 2.3 Hz, 1H), 5.71 – 5.58 (m, 1H), 3.33 (ddt, *J* = 8.3, 5.5, 2.8 Hz, 1H), 2.38 (s, 3H), 2.08 (ddq, *J* = 6.8, 3.6, 2.0, 1.5 Hz, 2H), 2.03 – 1.95 (m, 1H), 1.79 – 1.68 (m, 1H), 1.67 – 1.56 (m, 1H), 1.53 – 1.46 (m, 1H).
^{13}C-NMR (101 MHz, $CDCl_3$, ppm): δ = 146.1, 137.7, 132.2, 130.4, 129.8, 128.8, 127.0, 122.3, 41.4, 32.7, 25.1, 23.1, 21.2.
IR (ATR, cm^{-1}) ṽ = 2929, 2860, 1653, 1477, 1446, 1436, 1027, 892, 878, 814, 756.
MS (EI, 70 eV, %) m/z = 252 (41), 250 (42), 171 (51), 156 (23), 144 (10), 143 (100), 142 (11), 141 (26), 129 (28), 128 (76), 115 (32), 105 (24), 79 (12).
HRMS (EI, 70 eV) m/z: calc. for **$C_{13}H_{15}Br$**: 250.0357; found: 250.0350.

Synthesis of 2-bromo-5-(cyclohex-2-en-1-yl)pyridine (13a)

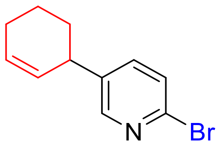

Compound **13a** was prepared *via* **TP1** using 2,5-dibromopyridine (**10a**, 119 mg, 0.50 mmol), dry toluene (1.0 mL) and PMDTA (63 µL, 0.30 mmol). Then, *s*Bu_2Mg·2LiOR (**1c**, 0.36 mL, 0.30 mmol) was added at -20 °C. After stirring at -20 °C for 30 min, CuCN·2LiCl (1.00 M in THF, 50 µL, 50 µmol) and 3-bromocyclohexene (46 µL, 0.40 mmol) were added and the reaction solution was allowed to warm to room temperature overnight. After work-up, the crude product was purified *via* column chromatography (*iso*hexane:diethyl ether = 10:0.05, R_f = 0.15) to give the product **13a** (69 mg, 290 µmol, 72% yield) as a yellowish oil.

^{1}H-NMR (400 MHz, $CDCl_3$, ppm): δ = 8.23 (t, *J* = 1.7 Hz, 1H), 7.40 (d, *J* = 1.6 Hz, 2H), 5.96 (dtd, *J* = 9.9, 3.7, 2.3 Hz, 1H), 5.68 – 5.48 (m, 1H), 3.40 (ddt, *J* = 8.2, 5.5, 2.8 Hz, 1H), 2.09

(dddd, *J* = 8.9, 5.4, 4.2, 2.6 Hz, 2H), 2.06 – 1.94 (m, 1H), 1.77 – 1.67 (m, 1H), 1.67 – 1.57 (m, 1H), 1.50 (dddd, *J* = 13.1, 10.1, 8.1, 3.1 Hz, 1H).

^{13}C-NMR (101 MHz, $CDCl_3$, ppm): δ = 149.9, 141.3, 139.6, 138.2, 130.1, 128.2, 127.9, 38.8, 32.4, 24.9, 20.8.

IR (ATR, cm^{-1}) ṽ = 2927, 2868, 2857, 1670, 1606, 1574, 1560, 1453, 1430, 1377, 1355, 1346, 1260, 1190, 1088, 1022, 830.

MS (EI, 70 eV, %) m/z = 240 (12), 239 (93), 238 (29), 237 (100), 236 (17), 224 (31), 222 (32), 211 (27), 210 (39), 209 (31), 208 (33), 183 (10), 158 (24), 143 (17), 130 (70), 129 (12), 128 (15), 117 (23), 116 (15), 104 (14), 103 (19), 89 (11), 79 (10), 77 (26), 51 (13), 41 (10).

HRMS (EI, 70 eV) m/z: calc. for **$C_{11}H_{12}BrN$**: 237.0153; found: 237.0148.

Synthesis of 2-bromo-5-(butylthio)-3-methylpyridine (13b)

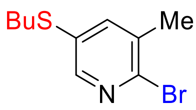

Compound **13b** was prepared *via* **TP1** using 2,5-dibromo-3-methylpyridine (**10b**, 251 mg, 1.00 mmol), dry toluene (2.0 mL) and PMDTA (0.13 mL, 0.60 mmol). Then, *s*Bu_2Mg·2LiOR (**1c**, 0.71 mL, 0.60 mmol) was added at -20 °C. After stirring at -20 °C for 30 min, *S*-butyl benzenesulfonothioate (184 mg, 0.80 mmol) was added and the reaction mixture was allowed to warm to room temperature overnight. After work-up, the crude product was purified *via* column chromatography (*iso*hexane:diethyl ether = 98:2, R_f = 0.20) to give the product **13b** (181 mg, 696 µmol, 87% yield) as a colorless oil.

^{1}H-NMR (400 MHz, $CDCl_3$, ppm): δ = 8.11 (d, *J* = 2.4 Hz, 1H), 7.67 – 7.40 (m, 1H), 3.04 – 2.72 (m, 2H), 2.35 (s, 3H), 1.66 – 1.55 (m, 2H), 1.49 – 1.38 (m, 2H), 0.92 (t, *J* = 7.3 Hz, 3H).

^{13}C-NMR (101 MHz, $CDCl_3$, ppm): δ = 147.2, 142.0, 139.4, 135.2, 133.6, 33.6, 31.2, 22.0, 21.9, 13.7.

IR (ATR, cm^{-1}) ṽ = 2957, 2929, 2872, 1534, 1453, 1436, 1397, 1383, 1376, 1124, 1047, 718, 685.

MS (EI, 70 eV, %) m/z = 261 (11), 259 (10), 232 (34), 230 (35), 228 (13), 226 (16), 219 (99), 218 (17), 217 (100), 216 (18), 214 (15), 212 (15), 205 (83), 204 (25), 203 (82), 202 (24), 201 (14), 199 (14), 186 (32), 184 (32), 173 (10), 172 (13), 171 (10), 170 (13), 161 (20), 159 (19).

HRMS (EI, 70 eV) m/z: calc. for **$C_{10}H_{14}BrNS$**: 259.0030; found: 259.0023.

Synthesis of 2-bromo-5-(cyclohex-2-en-1-yl)-4-methylpyridine (13c)

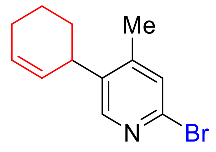

Compound **13c** was prepared *via* **TP1** using 2,5-dibromo-4-methylpyridine (**10c**, 126 mg, 0.50 mmol), dry toluene (1.0 mL) and PMDTA (63 µL, 0.30 mmol). Then, *s*Bu_2Mg·2LiOR (**1c**, 0.36 mL, 0.30 mmol) was added at -20 °C. After stirring at -20 °C for 30 min, CuCN·2LiCl (1.00 M in THF, 50 µL, 50 µmol) and 3-bromocyclohexene (46 µL, 0.40 mmol) were added and the reaction solution was allowed to warm to room temperature overnight. After work-up, the crude product was purified *via* column chromatography (*iso*hexane:diethyl ether = 99:1, R_f = 0.11) to give the product **13c** (99 mg, 393 µmol, 98% yield) as a yellowish oil.

^{1}H-NMR (400 MHz, $CDCl_3$, ppm): δ = 8.10 (s, 1H), 7.24 (s, 1H), 5.96 (dtd, *J* = 10.0, 3.7, 2.4 Hz, 1H), 5.60 (dq, *J* = 9.9, 2.3 Hz, 1H), 3.53 (ddt, *J* = 8.2, 5.6, 2.8 Hz, 1H), 2.31 (s, 3H), 2.13 – 2.05 (m, 2H), 2.00 – 1.90 (m, 1H), 1.76 – 1.65 (m, 1H), 1.64 – 1.55 (m, 1H), 1.45 (dddd, *J* = 12.9, 9.8, 7.7, 3.1 Hz, 1H).

^{13}C-NMR (101 MHz, $CDCl_3$, ppm): δ = 149.7, 148.1, 139.7, 139.2, 129.9, 129.0, 128.2, 35.7, 30.2, 24.9, 20.7, 18.7.

IR (ATR, cm^{-1}) ṽ = 2926, 2857, 2836, 1579, 1545, 1465, 1446, 1349, 1341, 1293, 1154, 1142, 1086, 983, 893, 883, 864, 784, 723.

MS (EI, 70 eV, %) m/z = 253 (72), 252 (13), 251 (74), 250 (14), 238 (36), 225 (32), 224 (96), 223 (30), 222 (100), 210 (20), 209 (11), 208 (20), 187 (12), 185 (12), 172 (36), 157 (22), 156 (14), 144 (84), 143 (17), 142 (16), 131 (16), 130 (16), 128 (16), 115 (23), 77 (11).

HRMS (EI, 70 eV) m/z: calc. for **$C_{12}H_{14}BrN$**: 251.0310; found: 251.0305.

Synthesis of 6-bromo-2-methyl-3-(2-methylallyl)pyridine (13d)

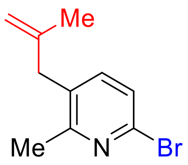

Compound **13d** was prepared *via* **TP1** using 2,5-dibromo-6-methylpyridine (**10d**, 126 mg, 0.50 mmol), dry toluene (1.0 mL) and PMDTA (63 µL, 0.30 mmol). Then, *s*Bu_2Mg·2LiOR (**1c**, 0.36 mL, 0.30 mmol) was added at -20 °C. After stirring at -20 °C for 30 min, CuCN·2LiCl (1.00 M in THF, 50 µL, 50 µmol) and 3-bromo-2-methylprop-1-ene (40 µL, 0.40 mmol) were added and the reaction mixture was allowed to warm to room temperature overnight. After work-up, the crude product was purified *via* column chromatography (*iso*hexane:diethyl ether = 99:1, R_f = 0.11) to give the product **13d** (84 mg, 371 µmol, 93% yield) as a yellowish oil.

^{1}H-NMR (400 MHz, CD_3CN, ppm): δ = 7.36 (d, *J* = 8.0 Hz, 1H), 7.31 (d, *J* = 8.0 Hz, 1H), 4.84 (s, 1H), 4.49 (s, 1H), 3.29 (s, 2H), 2.41 (s, 3H), 1.71 (s, 3H).

^{13}C-NMR (101 MHz, CD_3CN, ppm): δ = 159.8, 144.3, 141.3, 139.0, 133.7, 126.2, 112.5, 40.5, 22.6, 22.0.

IR (ATR, cm^{-1}) ṽ = 2954, 2926, 1651, 1576, 1558, 1435, 1168, 1126, 893, 881, 811.

MS (EI, 70 eV, %) m/z = 226 (99), 224 (100), 212 (52), 211 (11), 210 (54), 209 (11), 187 (35), 185 (35), 145 (22), 144 (16), 131 (19), 130 (15).

HRMS (EI, 70 eV) m/z: calc. for **$C_{10}H_{12}NBr$**: 225.0153; found: 225.0067.

Synthesis of 5-allyl-2-bromo-4-chloro-3-fluoropyridine (13e)

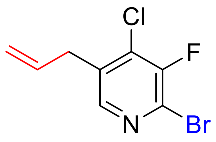

Compound **13e** was prepared *via* **TP1** using 2,5-dibromo-4-chloro-3-fluoropyridine (**10e**, 116 mg, 0.40 mmol), dry toluene (0.8 mL) and PMDTA (52 µL, 0.24 mmol). Then, *s*Bu_2Mg·2LiOR (**1c**, 0.29 mL, 0.24 mmol) was added at -20 °C. After stirring at -20 °C for 30 min, CuCN·2LiCl (1.00 M in THF, 40 µL, 40 µmol) and allyl bromide (28 µL, 0.32 mmol) were

added and the reaction solution was allowed to warm to room temperature overnight. After work-up, the crude product was purified *via* column chromatography (*iso*hexane, R_f = 0.15) to give the product **13e** (58 mg, 232 µmol, 72% yield) as a yellowish oil.

^{1}H-NMR (400 MHz, $CDCl_3$, ppm): δ = 8.06 (s, 1H), 5.91 (ddt, *J* = 16.7, 10.1, 6.4 Hz, 1H), 5.39 – 4.92 (m, 2H), 3.49 (dt, *J* = 6.4, 1.6 Hz, 2H).
^{13}C-NMR (101 MHz, $CDCl_3$, ppm): δ = 152.6 (d, *J* = 260.8 Hz), 145.7 (d, *J* = 6.5 Hz), 135.6 (d, *J* = 2.8 Hz), 133.0, 131.7, 128.0 (d, *J* = 23.8 Hz), 118.4, 34.3 (d, *J* = 1.5 Hz).
^{19}F-NMR (377 MHz, $CDCl_3$, ppm): δ = -113.2.
IR (ATR, cm^{-1}) ṽ = 1640, 1570, 1443, 1400, 1207, 992, 923, 789.
MS (EI, 70 eV, %) m/z = 253 (13), 252 (24), 251 (56), 250 (100), 249 (42), 248 (76), 216 (12), 215 (15), 214 (13), 213 (15), 169 (10), 168 (13), 135 (41), 134 (23), 107 (14).
HRMS (EI, 70 eV) m/z: calc. for **$C_8H_6BrClFN$:** 248.9356; found: 248.9353.

Synthesis of 2-bromo-4-(2-methylallyl)pyridine (13f)

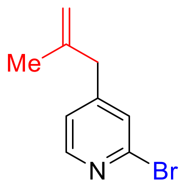

Compound **13f** was prepared *via* **TP1** using 2,4-dibromopyridine (**10f**, 119 mg, 0.50 mmol), dry toluene (1.0 mL) and PMDTA (63 µL, 0.30 mmol). Then, *s*Bu_2Mg·2LiOR (**1c**, 0.36 mL, 0.30 mmol) was added at -20 °C. After stirring at -20 °C for 30 min, CuCN·2LiCl (1.00 M in THF, 50 µL, 50 µmol) and 3-bromo-2-methylprop-1-ene (40 µL, 0.40 mmol) were added and the reaction mixture was allowed to warm to room temperature overnight. After work-up, the crude product was purified *via* column chromatography (*iso*hexane:ethyl acetate = 97:3, R_f = 0.18) to give the product **13f** (54 mg, 255 µmol, 64% yield) as a colorless oil.

^{1}H-NMR (400 MHz, $CDCl_3$, ppm): δ = 8.26 (d, *J* = 5.0 Hz, 1H), 7.33 (s, 1H), 7.09 (d, *J* = 5.0 Hz, 1H), 4.90 (s, 1H), 4.76 (s, 1H), 3.27 (s, 2H), 1.66 (s, 3H).
^{13}C-NMR (101 MHz, $CDCl_3$, ppm): δ = 152.1, 150.0, 142.5, 142.4, 128.5, 123.6, 114.2, 43.6, 22.2.

IR (ATR, cm^{-1}) $\tilde{v}$ = 2974, 2937, 2912, 1650, 1587, 1541, 1463, 1446, 1436, 1378, 1207, 1117, 1079, 987, 897, 874, 850, 806, 734, 706, 693.

MS (EI, 70 eV, %) m/z = 133 (10), 132 (100), 117 (29), 57 (13), 55 (10), 43 (13), 41 (14), 38 (12).

HRMS (EI, 70 eV) m/z: calc. for **$C_9H_{10}BrN$**: 210.9997; found: 210.9983.

Synthesis of 2-bromo-4-(butylthio)quinoline (13g)

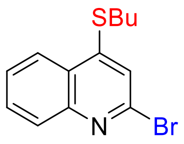

Compound **13g** was prepared *via* **TP1** using 2,4-dibromoquinoline (**10g**, 144 mg, 0.50 mmol), dry toluene (1.0 mL) and PMDTA (63 µL, 0.30 mmol). Then, *s*Bu_2Mg·2LiOR (**1c**, 0.36 mL, 0.30 mmol) was added at -20 °C. After stirring at -20 °C for 30 min, *S*-butyl benzenesulfonothioate (92 mg, 0.40 mmol) was added and the reaction solution was allowed to warm to room temperature overnight. After work-up, the crude product was purified *via* column chromatography (*iso*hexane, R_f = 0.15) to give the product **13g** (68 mg, 230 µmol, 57% yield) as a colorless oil.

^{1}H-NMR (400 MHz, $CDCl_3$, ppm): δ = 8.02 (ddd, *J* = 32.3, 8.5, 1.0 Hz, 2H), 7.71 (ddd, *J* = 8.4, 6.9, 1.4 Hz, 1H), 7.55 (ddd, *J* = 8.3, 6.9, 1.3 Hz, 1H), 7.22 (s, 1H), 3.09 (t, *J* = 7.3 Hz, 2H), 1.89 – 1.75 (m, 2H), 1.56 (dq, *J* = 14.6, 7.4 Hz, 2H), 1.01 (t, *J* = 7.4 Hz, 3H).

^{13}C-NMR (101 MHz, $CDCl_3$, ppm): δ = 151.2, 147.6, 141.6, 130.9, 129.3, 126.8, 125.4, 123.7, 119.1, 31.0, 30.0, 22.3, 13.8.

IR (ATR, cm^{-1}) $\tilde{v}$ = 2965, 2955, 2925, 1560, 1545, 1492, 1464, 1455, 1394, 1382, 1261, 1253, 1147, 1101, 829, 763, 701.

MS (EI, 70 eV, %) m/z = 268 (43), 266 (44), 255 (91), 254 (40), 253 (91), 252 (29), 250 (34), 248 (37), 241 (99), 239 (100), 236 (10), 234 (11), 208 (44), 207 (11), 206 (37), 160 (22), 159 (38), 128 (28), 127 (64), 116 (10).

HRMS (EI, 70 eV) m/z: calc. for **$C_{13}H_{14}BrNS$**: 295.0030; found: 295.0022.

Synthesis of (5-bromopyridin-2-yl)(phenyl)methanol (14a)

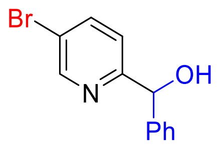

Compound **14a** was prepared *via* **TP1** using 2,5-dibromopyridine (**10a**, 119 mg, 0.50 mmol) and dry toluene (1.0 mL). Then, *s*Bu_2Mg·2LiOR (**1c**, 0.36 mL, 0.30 mmol) was added at -20 °C. After stirring at -20 °C for 30 min, benzaldehyde (42 µL, 0.40 mmol) was added and the reaction solution was allowed to warm to room temperature overnight. After work-up, the crude product was purified *via* column chromatography (*iso*hexane:ethyl acetate = 8:2, R_f = 0.28) to give the product **14a** (78 mg, 295 µmol, 74% yield) as a colorless oil.

^{1}H-NMR (400 MHz, $CDCl_3$, ppm): δ = 8.64 (d, *J* = 2.0 Hz, 1H), 7.78 (dd, *J* = 8.4, 2.3 Hz, 1H), 7.41 – 7.27 (m, 5H), 7.13 (dt, *J* = 8.5, 0.7 Hz, 1H), 5.78 (s, 1H), 4.70 (s, 1H).
^{13}C-NMR (101 MHz, $CDCl_3$, ppm): δ = 159.8, 148.8, 142.5, 140.1, 128.9, 128.3, 127.1, 122.9, 119.5, 74.9.
IR (ATR, cm^{-1}) ṽ = 3313, 3286, 1466, 1453, 1366, 1091, 1055, 1026, 1009, 764, 700.
MS (EI, 70 eV, %) m/z = 265 (55), 264 (25), 263 (56), 262 (22), 188 (22), 186 (25), 166 (15), 160 (16), 159 (33), 158 (32), 157 (34), 156 (17), 154 (10), 107 (18), 105 (29), 91 (16), 79 (62), 78 (40), 77 (100), 76 (22), 52 (11), 51 (54), 50 (24).
HRMS (EI, 70 eV) m/z: calc. for **$C_{12}H_{10}BrNO$:** 262.9946; found: 262.9943.

Synthesis of 2-allyl-5-bromo-3-methylpyridine (14b)

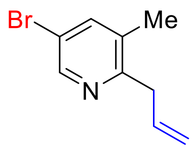

Compound **14b** was prepared *via* **TP1** using 2,5-dibromo-3-methylpyridine (**10b**, 126 mg, 0.50 mmol) and dry toluene (1.0 mL). Then, *s*Bu_2Mg·2LiOR (**1c**, 0.36 mL, 0.30 mmol) was added at -20 °C. After stirring at -20 °C for 30 min, CuCN·2LiCl (1.00 M in THF, 50 µL, 50 µmol) and allyl bromide (34 µL, 0.40 mmol) were added and the reaction solution was allowed to

warm to room temperature overnight. After work-up, the crude product was purified *via* column chromatography (*iso*hexane:ethyl acetate = 98:2, R_f = 0.19) to give the product **14b** (63 mg, 297 µmol, 74% yield) as a colorless oil.

^{1}H-NMR (400 MHz, $CDCl_3$, ppm): δ = 8.43 (d, *J* = 2.3 Hz, 1H), 7.57 (d, *J* = 1.9 Hz, 1H), 5.99 (ddt, *J* = 16.7, 10.1, 6.4 Hz, 1H), 5.19 – 4.88 (m, 2H), 3.52 (dt, *J* = 6.4, 1.6 Hz, 2H), 2.28 (s, 3H).

^{13}C-NMR (101 MHz, $CDCl_3$, ppm): δ = 156.9, 147.8, 140.1, 134.5, 133.6, 118.3, 116.7, 39.9, 18.6.

IR (ATR, cm^{-1}) ṽ = 2978, 2925, 1637, 1558, 1458, 1438, 1420, 1393, 1237, 1152, 1136, 1129, 1110, 995, 908, 890, 882, 728, 667.

MS (EI, 70 eV, %) m/z = 212 (98), 210 (100), 131 (35), 130 (15).

HRMS (EI, 70 eV) m/z: calc. for **$C_9H_{10}BrN$:** 210.9997; found: 210.9990.

Synthesis of (5-bromo-4-methylpyridin-2-yl)(cyclohexyl)methanone (14c)

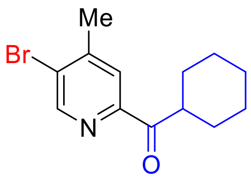

Compound **14c** was prepared *via* **TP1** using 2,5-dibromo-4-methylpyridine (**10c**, 126 mg, 0.50 mmol) and dry toluene (1.0 mL). Then, *s*Bu_2Mg·2LiOR (**1c**, 0.36 mL, 0.30 mmol) was added at -20 °C. After stirring at -20 °C for 30 min, *N*-methoxy-*N*-methylcyclohexanecarboxamide (69 mg, 0.40 mmol) was added and the reaction mixture was allowed to warm to room temperature overnight. After work-up, the crude product was purified *via* column chromatography (*iso*hexane:ethyl acetate = 99:1, R_f = 0.28) to give the product **14c** (59 mg, 209 µmol, 52% yield) as a yellowish oil.

^{1}H-NMR (400 MHz, $CDCl_3$, ppm): δ = 8.67 (s, 1H), 7.87 (s, 1H), 3.79 (ddt, *J* = 11.2, 7.7, 3.3 Hz, 1H), 2.44 (s, 3H), 1.93 – 1.77 (m, 4H), 1.52 – 1.32 (m, 4H), 1.30 – 1.07 (m, 2H).

^{13}C-NMR (101 MHz, $CDCl_3$, ppm): δ = 204.7, 151.6, 150.6, 148.1, 127.6, 124.7, 44.1, 29.0, 26.1, 25.8, 22.5.

IR (ATR, cm^{-1}) ṽ = 2926, 2853, 1692, 1583, 1449, 1385, 1309, 1256, 1240, 1165, 1058, 1046, 1031, 1006, 987, 898, 779.

MS (EI, 70 eV, %) m/z = 283 (17), 281 (19), 255 (16), 253 (17), 240 (14), 238 (14), 226 (14), 224 (15), 200 (22), 198 (22), 187 (41), 186 (11), 185 (44), 173 (99), 172 (22), 171 (100), 170 (23), 92 (15), 91 (10), 90 (11).

HRMS (EI, 70 eV) m/z: calc. for **$C_{13}H_{16}BrNO$**: 281.0415; found: 281.0409.

Synthesis of 1-(5-bromo-6-methylpyridin-2-yl)ethan-1-one (14d)

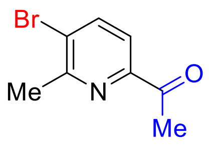

Compound **14d** was prepared *via* **TP1** using 2,5-dibromo-6-methylpyridine (**10d**, 126 mg, 0.50 mmol) and dry toluene (1.0 mL). Then, *s*Bu_2Mg·2LiOR (**1c**, 0.36 mL, 0.30 mmol) was added at -20 °C. After stirring at -20 °C for 30 min, *N*-methoxy-*N*-methylacetamide (41 mg, 0.40 mmol) was added and the reaction mixture was allowed to warm to room temperature overnight. After work-up, the crude product was purified *via* column chromatography (*iso*hexane:ethyl acetate = 95:5, R_f = 0.39) to give the product **14d** (69 mg, 322 µmol, 81% yield) as a white solid.

M.p. (°C): 95-97.

^{1}H-NMR (400 MHz, $CDCl_3$, ppm): δ = 7.90 (d, *J* = 8.2 Hz, 1H), 7.70 (d, *J* = 8.2 Hz, 1H), 2.70 (s, 3H), 2.67 (s, 3H).

^{13}C-NMR (101 MHz, $CDCl_3$, ppm): δ = 199.8, 157.0, 151.7, 140.6, 126.0, 120.5, 25.8, 25.2.

IR (ATR, cm^{-1}) ṽ = 3066, 2919, 1692, 1656, 1567, 1554, 1430, 1392, 1385, 1357, 1297, 1248, 1209, 1129, 1110, 1032, 984, 957, 835, 755, 695, 668.

MS (EI, 70 eV, %) m/z = 215 (50), 213 (53), 190 (11), 188 (12), 187 (96), 185 (100), 173 (71), 171 (72), 170 (52), 145 (15), 143 (15), 92 (40), 91 (23), 90 (19), 65 (12), 63 (12), 43 (12).

HRMS (EI, 70 eV) m/z: calc. for **C_8H_8BrNO**: 212.9789; found: 212.9785.

Synthesis of 2-allyl-5-bromo-4-chloro-3-fluoropyridine (14e)

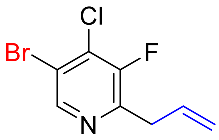

Compound **14e** was prepared *via* **TP1** using 2,5-dibromo-4-chloro-3-fluoropyridine (**10e**, 116 mg, 0.40 mmol) and dry toluene (0.8 mL). Then, sBu$_2$Mg·2LiOR (**1c**, 0.29 mL, 0.24 mmol) was added at -20 °C. After stirring at -20 °C for 30 min, CuCN·2LiCl (1.00 M in THF, 40 µL, 40 µmol) and allyl bromide (28 µL, 0.32 mmol) were added and the reaction solution was allowed to warm to room temperature overnight. After work-up, the crude product was purified *via* column chromatography (*iso*hexane, R_f = 0.15) to give the product **14e** (67 mg, 267 µmol, 84% yield) as a yellowish oil.

^{1}H-NMR (400 MHz, CDCl$_3$, ppm): δ = 8.50 (s, 1H), 6.00 (ddt, J = 16.9, 10.1, 6.7 Hz, 1H), 5.33 – 5.05 (m, 2H), 3.61 (ddt, J = 6.6, 2.8, 1.5 Hz, 2H).

^{13}C-NMR (101 MHz, CDCl$_3$, ppm): δ = 153.9 (d, J = 263.7 Hz), 148.0 (d, J = 16.1 Hz), 147.0 (d, J = 6.6 Hz), 133.1 (d, J = 1.4 Hz), 131.5 (d, J = 17.4 Hz), 119.4 (d, J = 4.3 Hz), 118.0, 36.3 (d, J = 2.3 Hz).

^{19}F-NMR (377 MHz, CDCl$_3$, ppm): δ = -122.1.

IR (ATR, cm^{-1}) $\tilde{\nu}$ = 1641, 1572, 1540, 1440, 1401, 1188, 993, 946, 919, 900, 855, 768, 704.

MS (EI, 70 eV, %) m/z = 253 (13), 252 (24), 251 (56), 250 (100), 249 (42), 248 (76), 216 (12), 215 (15), 214 (13), 213 (15), 169 (10), 168 (13), 135 (41), 134 (23), 107 (14).

HRMS (EI, 70 eV) m/z: calc. for **$C_8H_6BrClFN$**: 248.9356; found: 248.9353.

Synthesis of (4-bromopyridin-2-yl)dicyclopropylmethanol (14f)

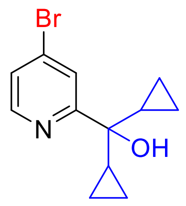

Compound **14f** was prepared *via* **TP1** using 2,4-dibromopyridine (**10f**, 119 mg, 0.50 mmol) and dry toluene (1.0 mL). Then, sBu$_2$Mg·2LiOR (**1c**, 0.36 mL, 0.30 mmol) was added at -20 °C. After stirring at -20 °C for 30 min, dicyclopropyl ketone (44 mg, 0.40 mmol) was added and the reaction mixture was allowed to warm to room temperature overnight. After work-up, the crude product was purified *via* column chromatography (*iso*hexane:ethyl acetate = 95:5, R_f = 0.32) to give the product **14f** (74 mg, 276 µmol, 69% yield) as a colorless oil.

^{1}H-NMR (400 MHz, CDCl$_3$, ppm): δ = 8.27 (d, *J* = 5.3 Hz, 1H), 7.71 (d, *J* = 1.4 Hz, 1H), 7.37 (dd, *J* = 5.4, 1.8 Hz, 1H), 4.70 (s, 1H), 1.13 (tt, *J* = 8.3, 5.3 Hz, 2H), 0.67 (dtd, *J* = 9.6, 5.6, 4.2 Hz, 2H), 0.48 (dddd, *J* = 9.2, 8.3, 5.9, 4.2 Hz, 2H), 0.32 (dtd, *J* = 9.7, 5.6, 4.3 Hz, 2H), 0.26 – 0.16 (m, 2H).

^{13}C-NMR (101 MHz, CDCl$_3$, ppm): δ = 167.3, 147.7, 133.7, 125.6, 123.6, 71.8, 20.1, 1.6, -0.6.

IR (ATR, cm^{-1}) ṽ = 3400, 3008, 1573, 1551, 1459, 1400, 1377, 1343, 1283, 1234, 1201, 1183, 1133, 1104, 1091, 1037, 1024, 1014, 926, 913, 874, 850, 820, 787, 739, 681.

MS (EI, 70 eV, %) m/z = 245 (11), 233 (79), 232 (13), 231 (100), 228 (18), 214 (11), 212 (14), 199 (16), 197 (11), 186 (12), 184 (15), 173 (15), 171 (12), 160 (14), 159 (13), 158 (35), 157 (14), 156 (31), 111 (44), 78 (17), 77 (10), 71 (10), 69 (13), 57 (23), 55 (15), 44 (13), 43 (44), 41 (37).

HRMS (EI, 70 eV) m/z: calc. for **$C_{12}H_{14}BrNO$:** 267.0259; found: 267.0253.

Synthesis of 4-bromo-2-(butylthio)quinoline (14g)

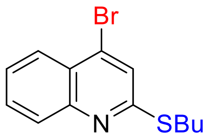

Compound **14g** was prepared *via* **TP1** using 2,4-dibromoquinoline (**10g**, 144 mg, 0.50 mmol) and dry toluene (1.0 mL). Then, $sBu_2Mg\cdot2LiOR$ (**1c**, 0.36 mL, 0.30 mmol) was added at -20 °C. After stirring at -20 °C for 30 min, *S*-butyl benzenesulfonothioate (92 mg, 0.40 mmol) was added and the reaction solution was allowed to warm to room temperature overnight. After work-up, the crude product was purified *via* column chromatography (*iso*hexane, R_f = 0.15) to give the product **14g** (92 mg, 311 µmol, 78% yield) as a colorless oil.

^{1}H-NMR (400 MHz, $CDCl_3$, ppm): δ = 8.12 – 8.02 (m, 1H), 7.91 (d, *J* = 8.4 Hz, 1H), 7.67 (ddd, *J* = 8.4, 6.9, 1.4 Hz, 1H), 7.52 (s, 1H), 7.49 (ddd, *J* = 8.2, 6.9, 1.2 Hz, 1H), 3.40 – 3.16 (m, 2H), 1.76 (tt, *J* = 8.6, 6.8 Hz, 2H), 1.52 (h, *J* = 7.4 Hz, 2H), 0.99 (t, *J* = 7.4 Hz, 3H).

^{13}C-NMR (101 MHz, $CDCl_3$, ppm): δ = 159.9, 148.8, 133.0, 130.6, 128.5, 126.9, 126.3, 125.5, 124.5, 31.5, 29.8, 22.2, 13.9.

IR (ATR, cm^{-1}) ṽ = 2956, 2928, 2871, 1612, 1574, 1542, 1485, 1464, 1455, 1385, 1365, 1270, 1250, 1203, 1144, 1091, 863, 853, 814, 755, 690.

MS (EI, 70 eV, %) m/z = 268 (43), 266 (44), 255 (91), 254 (40), 253 (91), 252 (29), 250 (34), 248 (37), 241 (99), 239 (100), 236 (10), 234 (11), 208 (44), 207 (11), 206 (37), 160 (22), 159 (38), 128 (28), 127 (64), 116 (10).

HRMS (EI, 70 eV) m/z: calc. for **$C_{13}H_{14}BrNS$**: 295.0030; found: 295.0022.

Synthesis of 3'-bromo-4'-methoxy-1,2,3,4-tetrahydro-1,1'-biphenyl (16)

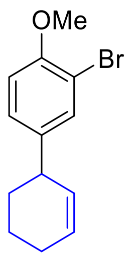

A dry and argon-flushed *Schlenk*-flask, equipped with a magnetic stirring bar and a septum, was charged with 4-bromo-2-iodoanisole (**15**, 313 mg, 1.00 mmol) and dissolved in dry THF (2.0 mL). Then, *i*PrMgCl·LiCl (**1a**, 1.0 mL, 1.20 mmol) was added dropwise at room temperature and the reaction was stirred for 15 min. The completion of the iodine/magnesium-exchange was checked by GC-analysis of reaction aliquots quenched with water, using undecane as internal standard. CuCN·2LiCl (1.00 M in THF, 100 µL, 100 µmol) and 3-bromocyclohexene (0.09 mL, 0.80 mmol) were then added at 0 °C and the reaction solution was allowed to warm to room temperature overnight. The mixture was quenched with a sat. aq. NH_4Cl solution (10 mL), diluted with water (10 mL) and extracted with ethyl acetate (3 x 30 mL). The combined organic extracts were dried over Na_2SO_4, filtered and concentrated *in vacuo*. The crude product was purified *via* column chromatography (*iso*hexane, R_f = 0.20) to give the product **16** (181 mg, 677 µmol, 85% yield) as a colorless oil.

^{1}H-NMR (400 MHz, $CDCl_3$, ppm): δ = 7.39 (d, *J* = 2.2 Hz, 1H), 7.11 (dd, *J* = 8.4, 2.2 Hz, 1H), 6.83 (d, *J* = 8.4 Hz, 1H), 5.89 (dtd, *J* = 9.9, 3.7, 2.3 Hz, 1H), 5.74 – 5.54 (m, 1H), 3.87 (s, 3H), 3.33 (ddp, *J* = 8.2, 5.5, 2.8 Hz, 1H), 2.08 (dddd, *J* = 9.0, 7.7, 4.3, 2.1 Hz, 2H), 2.02 – 1.92 (m, 1H), 1.76 – 1.67 (m, 1H), 1.67 – 1.42 (m, 2H).

^{13}C-NMR (101 MHz, $CDCl_3$, ppm): δ = 154.2, 140.5, 132.6, 129.8, 128.9, 127.8, 111.9, 111.5, 56.4, 40.8, 32.8, 25.1, 21.1.

IR (ATR, cm^{-1}) ṽ = 2921, 2854, 1589, 1480, 1451, 1438, 1201, 1023, 767.

MS (EI, 70 eV, %) m/z = 268 (64), 266 (67), 188 (12), 187 (84), 172 (28), 160 (12), 159 (100), 158 (21), 146 (11), 145 (22), 144 (73), 141 (10), 131 (16), 129 (15), 128 (36), 121 (41), 116 (15), 115 (36).

HRMS (EI, 70 eV) m/z: calc. for **$C_{13}H_{15}BrO$**: 266.0306; found: 266.0301.

Synthesis of 5'-iodo-2'-methoxy-1,2,3,4-tetrahydro-1,1'-biphenyl (17)

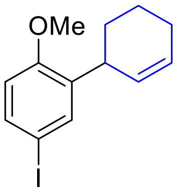

Compound **17** was prepared *via* **TP1** using 2-bromo-4-iodoanisole (**15**, 125 mg, 0.40 mmol) and dry toluene (8.0 mL). Then, *n*Bu_2Mg·2LiOR (**1d**, 0.32 mL, 0.28 mmol) was added at -10 °C. After stirring at -10 °C for 30 min, CuCN·2LiCl (1.00 M in THF, 40 µL, 40 µmol) as well as 3-bromocyclohexene (37 µL, 0.32 mmol) were added at -10 °C and the reaction solution was allowed to warm to room temperature overnight. After work-up, the crude product was purified *via* column chromatography (*iso*hexane, R_f = 0.45) to give the product **17** (65 mg, 207 µmol, 65% yield) as a colorless oil.

^{1}H-NMR (400 MHz, $CDCl_3$, ppm): δ = 7.46 (dd, *J* = 8.5, 2.3 Hz, 1H), 7.42 (d, *J* = 2.3 Hz, 1H), 6.62 (d, *J* = 8.5 Hz, 1H), 5.93 (dtd, *J* = 9.9, 3.7, 2.3 Hz, 1H), 5.74 – 5.48 (m, 1H), 3.80 (s, 3H), 3.76 (ddt, *J* = 8.4, 5.6, 2.8 Hz, 1H), 2.07 (dtt, *J* = 9.4, 3.7, 2.2 Hz, 2H), 1.97 (dddd, *J* = 12.8, 7.3, 5.6, 3.1 Hz, 1H), 1.71 – 1.57 (m, 2H), 1.45 (dddd, *J* = 12.7, 9.4, 7.4, 3.5 Hz, 1H).
^{13}C-NMR (101 MHz, $CDCl_3$, ppm): δ = 156.9, 137.5, 137.3, 135.8, 129.5, 129.3, 112.7, 83.2, 55.6, 34.2, 30.1, 25.2, 20.9.
IR (ATR, cm^{-1}) ṽ = 2934, 2831, 1639, 1592, 1485, 1468, 1447, 1431, 1406, 1326, 1304, 1279, 1241, 1174, 1138, 1124, 1038, 991, 912, 869, 801.
MS (EI, 70 eV, %) m/z = 315 (13), 314 (100), 299 (12), 281 (21), 260 (27), 258 (12), 225 (25), 207 (61), 191 (12), 187 (25), 172 (20), 159 (32), 158 (11), 157 (12), 153 (11), 144 (75), 131 (17), 129 (14), 128 (36), 127 (16), 121 (19), 118 (14), 116 (12), 115 (56), 91 (12), 89 (12).
HRMS (EI, 70 eV) m/z: calc. for **$C_{13}H_{15}IO$**: 314.0168; found: 314.0162.

Synthesis of 1-bromo-5-iodo-2-methoxy-4-methylbenzene (SM1)[13]

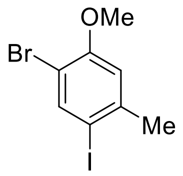

Under air, 1-bromo-2-methoxy-4-methylbenzene (3.00 g, 14.9 mmol), (diacetoxy)iodobenzene (2.64 g, 8.21 mmol) and finely crushed iodine (2.08 g, 8.21 mmol) were suspended in a mixture of acetic acid (30 mL) and acetic anhydride (15 mL). Then, H_2SO_4 (96% aq., 0.762 mL, 14.9 mmol) was added dropwise to start the reaction (exothermic addition), and then more slowly to avoid going over 40 °C. After coming back to room temperature, the mixture was diluted with DCM and the phases were separated. The organic phase was washed with water and then stirred vigorously with a 1.00 M NaOH solution in an Erlenmeyer-*flask* in order to quench the remaining AcOH/Ac_2O. Only then a 0.10 M $Na_2S_2O_3$ solution was added to quench the remaining iodine. The organic phase was dried and the solvent removed under reduced pressure. Then, the iodobenzene by-product was removed under vacuum. Recrystallization from MeOH (reflux to -18 °C) afforded the product **SM1** (3.90 g, 11.9 mmol, 80% yield) as white crystals.

M.p. (°C): 108-110.
^{1}H-NMR (400 MHz, $CDCl_3$, ppm): δ = 7.89 (s, 1H), 6.79 (s, 1H), 3.87 (s, 3H), 2.39 (s, 3H).
^{13}C-NMR (101 MHz, $CDCl_3$, ppm): δ = 156.1, 141.9 (d, *J* = 18.7 Hz), 113.4, 109.4, 89.5, 56.4, 28.2.
IR (ATR, cm^{-1}) ṽ = 2934, 1577, 1557, 1484, 1471, 1457, 1438, 1357, 1282, 1248, 1189, 1172, 1049, 878, 842.
MS (EI, 70 eV, %) m/z = 328 (99), 326 (100), 313 (30), 311 (31), 283 (10), 204 (17), 158 (12), 156 (11), 77 (15).
HRMS (EI, 70 eV) m/z: calc. for **C_8H_8BrIO**: 325.8803; found: 325.8799.

[13] Procedure from: Q. Dherbassy, J.-P. Djukic, J. Wencel-Delord, F. Colobert, *Angew. Chem. Int. Ed.* **2018**, *57*, 4668-4672; *Angew. Chem.* **2018**, *130*, 4758-4762.

Synthesis of 2-bromo-5-isopropylphenol (SM2)[14]

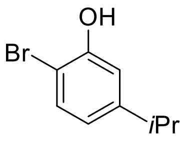

A dry and argon-flushed *Schlenk*-flask, equipped with a magnetic stirring bar and a septum, was charged with 3-isopropylphenol (2.74 mL, 20.0 mmol) and DCM (35 mL) and was cooled down to 0 °C. Br_2 (1.05 mL, 20.4 mmol, in 17 mL DCM) was slowly added at 0 °C and the reaction mixture was allowed to warm to room temperature overnight. The mixture was quenched with a 0.10 M $Na_2S_2O_3$ solution and extracted with DCM (3 x 30 mL). The combined organic extracts were dried over Na_2SO_4, filtered and concentrated *in vacuo*. The crude product was purified *via* column chromatography (*iso*hexane:ethyl acetate = 99:1, R_f = 0.15) to give the product **SM2** (1.70 g, 7.90 mmol, 40% yield) as a yellow oil.

^{1}H-NMR (400 MHz, $CDCl_3$, ppm): δ = 7.35 (d, *J* = 8.2 Hz, 1H), 6.90 (d, *J* = 2.1 Hz, 1H), 6.69 (dd, *J* = 8.3, 2.1 Hz, 1H), 5.44 (s, 1H), 2.84 (p, *J* = 6.9 Hz, 1H), 1.22 (d, *J* = 6.9 Hz, 6H).
^{13}C-NMR (101 MHz, $CDCl_3$, ppm): δ = 152.1, 150.9, 131.7, 120.3, 114.2, 107.2, 33.9, 23.9.
IR (ATR, cm^{-1}) ṽ = 3508, 2962, 1595, 1573, 1482, 1461, 1439, 1420, 1346, 1306, 1289, 1254, 1202, 1178, 1142, 1024, 941, 870, 806.
MS (EI, 70 eV, %) m/z = 216 (39), 214 (39), 201 (90), 199 (91), 120 (100), 91 (17).
HRMS (EI, 70 eV) m/z: calc. for **$C_9H_{11}BrO$**: 213.9993; found: 213.9987.

Synthesis of 1-bromo-2-methoxy-4-isopropylbenzene (SM3)

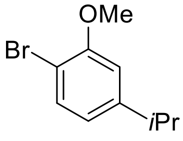

A dry and argon-flushed *Schlenk*-flask, equipped with a magnetic stirring bar and a septum, was charged with 2-bromo-5-isopropylphenol (**SM2**, 1.70 g, 7.90 mmol) and DMF (20 mL) and

[14] Adapted procedure from: L. Shu, P. Wang, W. Liu, C. Gu, *Org. Process Res. Dev.* **2012**, *16*, 1866-1869.

was cooled down to 0 °C. NaH (60%, 382 mg, 9.50 mmol) was slowly added at 0 °C and the reaction solution was stirred for 30 min. Methyl iodide (0.76 mL, 11.9 mmol) was then added at 0 °C and the reaction was allowed to warm to room temperature overnight. The mixture was quenched with a sat. aq. NH_4Cl solution (10 mL), diluted with water (10 mL) and extracted with ethyl acetate (3 x 30 mL). The combined organic extracts were dried over Na_2SO_4, filtered and concentrated *in vacuo*. The crude product was purified *via* column chromatography (*iso*hexane, R_f = 0.33) to give the product **SM3** (1.52 g, 6.63 mmol, 84% yield) as a yellow oil.

^{1}H-NMR (400 MHz, $CDCl_3$, ppm): δ = 7.43 (d, *J* = 8.1 Hz, 1H), 6.76 (d, *J* = 1.9 Hz, 1H), 6.72 (dd, *J* = 8.1, 2.0 Hz, 1H), 3.90 (s, 3H), 2.88 (p, *J* = 6.9 Hz, 1H), 1.24 (d, *J* = 6.9 Hz, 6H).
^{13}C-NMR (101 MHz, $CDCl_3$, ppm): δ = 155.8, 150.1, 133.1, 119.9, 110.6, 108.6, 56.2, 34.3, 24.1.
IR (ATR, cm^{-1}) $\tilde{v}$ = 2961, 2938, 1590, 1577, 1482, 1464, 1410, 1286, 1259, 1197, 1059, 1045, 1025, 852, 812.
MS (EI, 70 eV, %) m/z = 230 (33), 228 (34), 215 (79), 213 (80), 149 (17), 134 (100), 119 (19), 91 (18).
HRMS (EI, 70 eV) m/z: calc. for **$C_{10}H_{13}BrO$**: 228.0150; found: 228.0143.

Synthesis of 1-bromo-5-iodo-4-isopropyl-2-methoxybenzene (SM4)[13]

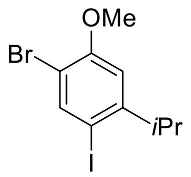

Under air, 1-bromo-2-methoxy-4-isopropylbenzene (**SM3**, 1.51 g, 6.60 mmol), (diacetoxy)iodobenzene (1.20 g, 3.73 mmol) and finely crushed iodine (936 mg, 3.73 mmol) were suspended in a mixture of acetic acid (13 mL) and acetic anhydride (6 mL). Then, H_2SO_4 (96% aq., 0.330 mL, 6.71 mmol) was added dropwise to start the reaction (exothermic addition), and then more slowly to avoid going over 40 °C. After coming back to room temperature, the mixture was diluted with DCM and the phases were separated. The organic phase was washed with water and then stirred vigorously with a 1.00 M NaOH solution in an Erlenmeyer-*flask* in order to quench the remaining AcOH/Ac_2O. Only then a 0.10 M $Na_2S_2O_3$ solution was added to quench the remaining iodine. The organic phase was dried and the

solvent removed under reduced pressure. Then, the iodobenzene by-product was removed under vacuum. Recrystallization from MeOH (reflux to -18 °C) afforded the product **SM4** (1.06 g, 2.99 mmol, 45% yield) as yellow crystals.

M.p. (°C): 46-48.
^{1}H-NMR (400 MHz, $CDCl_3$, ppm): δ = 7.92 (s, 1H), 6.77 (s, 1H), 3.89 (s, 3H), 3.13 (p, *J* = 6.8 Hz, 1H), 1.22 (d, *J* = 6.8 Hz, 6H).
^{13}C-NMR (101 MHz, $CDCl_3$, ppm): δ = 156.6, 151.2, 142.2, 109.9, 109.5, 89.3, 56.3, 38.2, 23.2.
IR (ATR, cm^{-1}) ṽ = 2962, 1576, 1469, 1440, 1386, 1361, 1338, 1244, 1083, 1047.
MS (EI, 70 eV, %) m/z = 356 (48), 354 (49), 341 (40), 339 (41), 245 (11), 215 (10), 214 (96), 213 (10), 212 (100), 171 (17), 169 (17), 148 (12), 147 (10), 133 (18), 127 (29), 118 (11), 117 (15), 115 (21), 105 (23), 103 (22), 102 (12), 91 (12), 90 (19), 89 (36), 77 (21), 63 (10).
HRMS (EI, 70 eV) m/z: calc. for **$C_{10}H_{12}BrIO$**: 353.9116; found: 353.9110.

Synthesis of 3'-allyl-4'-methoxy-1,2,3,4-tetrahydro-1,1'-biphenyl (A)

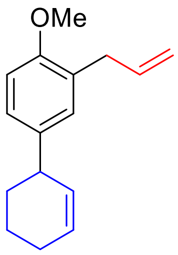

Compound **A** was prepared *via* **TP1** using 3'-bromo-4'-methoxy-1,2,3,4-tetrahydro-1,1'-biphenyl (**16**, 50 mg, 187 µmol) and dry toluene (0.4 mL). Then, *s*Bu_2Mg·2LiOR (**1d**, 0.13 mL, 0.11 mmol) was added at 25 °C. After stirring at 25 °C for 10 min, CuCN·2LiCl (1.00 M in THF, 19 µL, 19 µmol) and allyl bromide (13 µL, 150 µmol) were added at 0 °C and the reaction mixture was allowed to warm to room temperature overnight. After work-up, the crude product was purified *via* column chromatography (*iso*hexane, R_f = 0.15) to give the product **A** (26 mg, 114 µmol, 76% yield) as a colorless oil.

^{1}H-NMR (400 MHz, $CDCl_3$, ppm): δ = 7.05 (dd, *J* = 8.3, 2.3 Hz, 1H), 7.00 (d, *J* = 2.3 Hz, 1H), 6.81 (d, *J* = 8.3 Hz, 1H), 6.02 (ddt, *J* = 16.8, 10.0, 6.6 Hz, 1H), 5.92 – 5.81 (m, 1H), 5.77 – 5.60

(m, 1H), 5.12 – 5.00 (m, 2H), 3.82 (s, 3H), 3.39 (dd, *J* = 6.6, 1.6 Hz, 2H), 3.35 (ddt, *J* = 8.2, 5.5, 2.8 Hz, 1H), 2.16 – 2.05 (m, 2H), 2.00 (dddd, *J* = 15.1, 7.8, 3.9, 2.0 Hz, 1H), 1.75 (dtq, *J* = 11.7, 4.6, 2.2, 1.6 Hz, 1H), 1.70 – 1.47 (m, 2H).

^{13}C-NMR (101 MHz, $CDCl_3$, ppm): δ = 155.7, 138.7, 137.3, 130.8, 129.4, 128.4, 128.2, 126.3, 115.4, 110.3, 55.6, 41.2, 34.5, 32.9, 25.3, 21.5.

IR (ATR, cm^{-1}) ṽ = 2930, 2835, 1638, 1608, 1499, 1464, 1443, 1421, 1295, 1248, 1182, 1130, 1034, 996, 911, 811.

MS (EI, 70 eV, %) m/z = 228 (44), 188 (14), 187 (100), 172 (10), 159 (48), 145 (14), 144 (39), 141 (13), 129 (11), 128 (17), 121 (32), 115 (17), 79 (10).

HRMS (EI, 70 eV) m/z: calc. for **$C_{16}H_{20}O$**: 228.1514; found: 228.1508.

Synthesis of 5'-allyl-2'-methoxy-1,2,3,4-tetrahydro-1,1'-biphenyl (B)

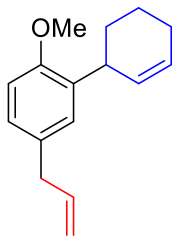

Compound **B** was prepared *via* **TP1** using 5'-iodo-2'-methoxy-1,2,3,4-tetrahydro-1,1'-biphenyl (**17**, 30 mg, 97 µmol), dry toluene (0.3 mL) and PMDTA (10 µL, 45 µmol). Then, *s*Bu_2Mg·2LiOR (**1c**, 0.06 mL, 58 µmol) was added at 25 °C. After stirring at 25 °C for 10 min, CuCN·2LiCl (1.00 M in THF, 10 µL, 10 µmol) and allyl bromide (7 µL, 78 µmol) were added at 0 °C and the reaction was allowed to warm to room temperature overnight. After work-up, the crude product was purified *via* column chromatography (*iso*hexane, R_f = 0.20) to give the product **B** (16 mg, 70.1 µmol, 90% yield) as a colorless oil.

^{1}H-NMR (400 MHz, $CDCl_3$, ppm): δ = 7.00 (dq, *J* = 5.2, 2.3 Hz, 2H), 6.87 – 6.72 (m, 1H), 6.08 – 5.83 (m, 2H), 5.66 (dq, *J* = 10.2, 2.4 Hz, 1H), 5.13 – 4.89 (m, 2H), 3.82 (s, 4H), 3.32 (dt, *J* = 6.7, 1.5 Hz, 2H), 2.17 – 2.03 (m, 2H), 2.02 – 1.93 (m, 1H), 1.78 – 1.58 (m, 2H), 1.55 – 1.40 (m, 1H).

^{13}C-NMR (101 MHz, $CDCl_3$, ppm): δ = 155.4, 138.2, 134.6, 131.8, 130.5, 128.9, 128.5, 126.8, 115.4, 110.4, 55.6, 39.7, 34.4, 30.4, 25.3, 21.3.

IR (ATR, cm^{-1}) ṽ = 2930, 2858, 2835, 2359, 1684, 1654, 1497, 1464, 1458, 1446, 1437, 1244, 1117, 1033, 810.

MS (EI, 70 eV, %) m/z = 229 (17), 228 (100), 213 (14), 187 (70), 185 (14), 174 (20), 172 (28), 171 (31), 159 (87), 158 (16), 157 (16), 155 (14), 153 (23), 152 (19), 147 (27), 145 (17), 144 (76), 141 (37), 131 (30), 129 (32), 128 (58), 121 (30), 115 (75), 91 (36), 79 (16), 77 (19).
HRMS (EI, 70 eV) m/z: calc. for **$C_{16}H_{20}O$**: 228.1514; found: 228.1508.

Synthesis of dicyclohexyl(5-iodo-2-methoxy-4-methylphenyl)methanol (C)

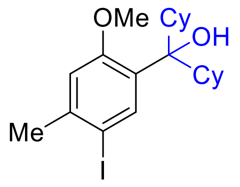

Compound **C** was prepared *via* **TP1** using 1-bromo-5-iodo-4-methyl-2-methoxybenzene (**SM1**, 164 mg, 0.50 mmol) and dry toluene (10 mL). Then, *n*Bu_2Mg·2LiOR (**1d**, 0.38 mL, 0.30 mmol) was added at -10 °C. After stirring at -10 °C for 30 min, dicyclohexyl ketone (78 mg, 0.40 mmol) was added and the reaction solution was allowed to warm to room temperature overnight. After work-up, the crude product was purified *via* column chromatography (*iso*hexane:ethyl acetate = 95:5, R_f = 0.22) to give the product **C** (140 mg, 316 µmol, 79%) as a colorless oil.

^{1}H-NMR (400 MHz, DMSO-d$_6$, ppm): δ = 7.50 (s, 1H), 6.55 (s, 1H), 3.82 (s, 1H), 2.14 (p, *J* = 1.9 Hz, 6H), 1.95 (s, 2H), 1.77 (t, *J* = 11.3 Hz, 2H), 1.28 (ddd, *J* = 50.8, 27.4, 12.5 Hz, 7H), 1.05 – 0.90 (m, 2H), 0.89 – 0.66 (m, 4H), 0.66 – 0.47 (m, 3H), 0.46 – 0.26 (m, 2H).
^{13}C-NMR (101 MHz, DMSO-d$_6$, ppm): δ = 156.7, 139.5, 132.2, 113.8, 90.0, 80.1, 55.3, 43.2, 27.9, 27.0, 26.8, 26.4, 26.3.
IR (ATR, cm^{-1}) ṽ = 3481, 3437, 2251, 2123, 1053, 1024, 1005, 821, 758.
MS (EI, 70 eV, %) m/z = 360 (15), 359 (100), 214 (36), 83 (10).
HRMS (EI, 70 eV) m/z: calc. for **$C_{21}H_{31}IO_2$**: 442.1369; found: 442.1363.

Synthesis of (5-iodo-4-isopropyl-2-methoxyphenyl)(4-(trifluoromethyl)phenyl)methanone (D)

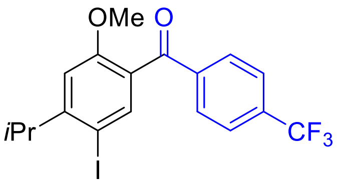

Compound **D** was prepared *via* **TP1** using 1-bromo-5-iodo-4-isopropyl-2-methoxybenzene (**SM4**, 178 mg, 0.50 mmol) and dry toluene (10 mL). Then, nBu$_2$Mg·2LiOR (**1d**, 0.38 mL, 0.30 mmol) was added at -10 °C. After stirring at -10 °C for 30 min, *N*-methoxy-*N*-methyl-4-(trifluoromethyl)benzamide (93 mg, 0.40 mmol) was added and the reaction solution was allowed to warm to room temperature overnight. After work-up, the crude product was purified *via* column chromatography (*iso*hexane:ethyl acetate = 95:5, R_f = 0.27) to give the product **D** (147 mg, 328 µmol, 82% yield) as a yellowish oil.

^{1}H-NMR (400 MHz, CDCl$_3$, ppm): δ = 7.88 (dt, *J* = 7.9, 0.8 Hz, 2H), 7.82 (s, 1H), 7.75 – 7.66 (m, 2H), 6.87 (s, 1H), 3.70 (s, 3H), 3.22 (h, *J* = 6.8 Hz, 1H), 1.28 (d, *J* = 6.8 Hz, 6H).

^{13}C-NMR (101 MHz, CDCl$_3$, ppm): δ = 193.7, 158.4, 155.6, 140.7, 140.2, 134.2 (q, *J* = 32.5 Hz), 130.0, 127.8, 125.4 (q, *J* = 3.7 Hz), 123.8 (q, *J* = 272.7 Hz), 109.5, 89.6, 55.7, 38.7, 23.0.

^{19}F-NMR (377 MHz, CDCl$_3$, ppm): δ = -63.0.

IR (ATR, cm^{-1}) $\tilde{v}$ = 2964, 1667, 1594, 1463, 1410, 1390, 1373, 1340, 1324, 1311, 1277, 1252, 1233, 1168, 1129, 1108, 1066, 1034, 1017, 955, 857, 778.

MS (EI, 70 eV, %) m/z = 449 (18), 448 (97), 431 (40), 430 (11), 379 (21), 306 (12), 304 (58), 303 (100), 290 (10), 289 (40), 245 (12), 176 (10), 173 (88), 165 (12), 161 (63), 147 (15), 145 (68).

HRMS (EI, 70 eV) m/z: calc. for **$C_{18}H_{16}F_3IO_2$**: 448.0147; found: 448.0144.

X-Ray Crystallographic Studies

A crystal of $[Ar_2(OR)MgLi]_2$ (Ar = *o*-OMe-C_6H_4, R = 2-ethylhexyl) (**16**) immersed in parabar oil was mounted at ambient conditions and transferred into the stream of nitrogen (173 K). All measurements were made on a *Rigaku Synergy S* area-detector diffractometer[15] using mirror optics monochromated Cu *K*α radiation (λ = 1.54184 Å).[16] The unit cell constants and an orientation matrix for data collection were obtained from a least-squares refinement of the setting angles of reflections in the range 7.616° < 2θ < 154.218°. A total of 3740 frames were collected using ω scans, with 0.25 second exposure time (0.95 s for high-angle reflections), a rotation angle of 0.5° per frame, a crystal-detector distance of 65.0 mm, at T = 110(2) K.

Data reduction was performed using the *CrysAlisPro*[15] program. The intensities were corrected for Lorentz and polarization effects, and an absorption correction based on the multi-scan method using SCALE3 ABSPACK in *CrysAlisPro*[15] was applied. Data collection and refinement parameters are given in *Table S4.*

The structure was solved by direct methods using *SHELXT*,[17] which revealed the positions of all non-hydrogen atoms of the title compound. All non-hydrogen atoms were refined anisotropically. H-atoms were assigned in geometrically calculated positions and refined using a riding model where each H-atom was assigned a fixed isotropic displacement parameter with a value equal to 1.2Ueq of its parent atom (1.5Ueq for methyl groups).

Refinement of the structure was carried out on F^2 using full-matrix least-squares procedures, which minimized the function $\Sigma w(F_o^2 - F_c^2)^2$. The weighting scheme was based on counting statistics and included a factor to downweight the intense reflections. All calculations were performed using the *SHELXL-2014/7*[18] program in OLEX2.[19]

The X-ray crystal structure determination service unit of the Department of Chemistry and Biochemistry of the University of Bern is acknowledged for measuring, solving, refining and summarizing the structures of compound **16**. The Synergy diffractometer was partially funded by the Swiss National Science Foundation (SNF) within the R'Equip programme (project number 206021_177033).

[15] Oxford Diffraction (**2018**). *CrysAlisPro* (Version 1.171.40.37a). Oxford Diffraction Ltd., Yarnton, Oxfordshire, UK.
[16] P. Macchi, H. B. Bürgi, A. S. Chimpri, J. Hauser, Z. Gal, *J. Appl. Cryst* **2011**, *44*, 763-771.
[17] G. M. Sheldrick, *Acta Cryst*. **2015**, *A71*, 3-8.
[18] G. M. Sheldrick, *Acta Cryst*. **2015**, *C71*, 3-8.
[19] O. V. Dolomanov, L. J. Bourhis, R. J. Gildea, J. A. K. Howard, H. Puschmann, *J. Appl. Cryst.* **2009**, *42,* 339-341.

Table S4. Table of selected crystallograhic parameters of $[Ar_2(OR)MgLi]_2$ (**16**).

	$[Ar_2(OR)MgLi]_2$ (Ar = *o*-OMe-C_6H_4, R = 2-ethylhexyl) (**16**)
**CCDC Number**	2027201
**Empirical formula**	$C_{44}H_{64}Li_2Mg_2O_6$
**Mol. Mass**	751.45
**Crystal system**	Monoclinic
**a/Å**	23.3524(4)
**b/Å**	10.7931(2)
**c/Å**	17.4848(3)
**α/°**	90
**β/°**	96.207(2)
**γ/°**	90
**V/Å^3**	4381.12(13)
**Z**	4
**λ/Å**	0.71073
**Measured reflections**	26142
**Unique reflections**	4530
***R*int**	0.0387
**Observed rflns [I > 2σ(I)]**	25366
**Goof**	1.0590
***R* [on *F*, obs rflns only]**	0.0508
**ω*R* [on F^2, all data]**	0.14490
**Largest diff. peak/hole e/Å^{-3}**	0.3500/-0.6000

NMR Studies

Synthesis of $[Ar_2(OR)MgLi]_2$ (16) (Ar = *o*-OMe-C_6H_4, R = 2-ethylhexyl)

In an argon-flushed *Schlenk*-flask, **1c** was prepared from 1.00 mmol of *n*Bu_2Mg *via* Method A. Once reconstituted in 5 mL toluene to achieve a pale yellow solution, 2.00 mmol of 2-bromoanisole (0.25 mL) was added. The resulting colourless solution was stirred at room temperature for 30 min and became slightly turbid. All volatiles were then removed under vacuum to give a white, waxy solid. This was then suspended in 2 mL hexane and solubilised with 2 mL of dry toluene with gentle heating applied. Slow cooling to room temperature resulted in a crop of colourless crystals – dimeric compound **16** $[Ar_2(OR)MgLi]_2$. Yield: 165 mg, 22%. Note, this compoud crystallises with a molecule of toluene, however, this is not present in the final spectra or elemental anlysis results. Anal. calcd. for $C_{44}H_{62}Li_2Mg_2O_6$: C, 70.51; H, 8.34. Found: C, 70.56; H, 8.42.

^{1}H-NMR (300.1 MHz, D_8-Tol, ppm): δ = 8.06 (d, 4H, C-H_{ortho}), 7.20 (t, 4H, C-H_{meta}), 7.12 (t, 4H, C-H_{para} + D_8-Tol), 6.67 (d, 4H, C-H_{meta}), 3.80 (m, 4H, OCH_2, OR), 3.31 (s, 12H, O*Me*, Ar), 1.52 (m, 2H, C-*H*, OR), 1.34-0.91 (m, 16H, CH_2 (Et), (CH_2)$_3$, OR), 0.73 (t, 6H, CH_3, Et, OR), 0.65 (t, 6H, CH_3, OR).

^{7}Li-NMR (156 MHz, D_8-Tol, ppm): δ = 1.41 $[Ar_2(OR)Mg\mathit{Li}]_2$.

^{13}C{^{1}H}-NMR (75.5 MHz, D_8-Tol, ppm): δ = 166.3 (C_q-OMe), 150.9 (C_q-Mg), 143.2 (C_{Ar}-H), 127.4 (C_{Ar}-H), 123.3 (C_{Ar}-H), 123.3 (C_{Ar}-H), 109.4 (C_{Ar}-H), 66.6 (O*C*H_2, OR), 55.9 (O*Me*, Ar), 44.3 (*C*-H, OR), 31.1 (*C*H_2, Et, OR), 29.4 (*C*H_2, OR), 24.0 (*C*H_2, OR), 23.4 (*C*H_2, OR), 14.2 (*C*H_3, Et, OR), 11.0 (*C*H_3, OR).

^{1}H-DOSY NMR spectroscopy revealed co-diffusion of the aryl and alkoxy-assigned peaks, suggesting that the solid-state structure of $[Ar_2(OR)MgLi]$ is retained in solution and are part of the same molecular entity. A mean diffusion coefficient of $D = 5.051 \times 10^{-10}$ m^2/s.

NMR spectroscopic analysis of the filtrate of compound **16** revealed the presence of residual compound **16** and free LiOR visible by both ^{1}H and ^{13}C{^{1}H} NMR spectra – see **Figure S6** and **Figure S5**.

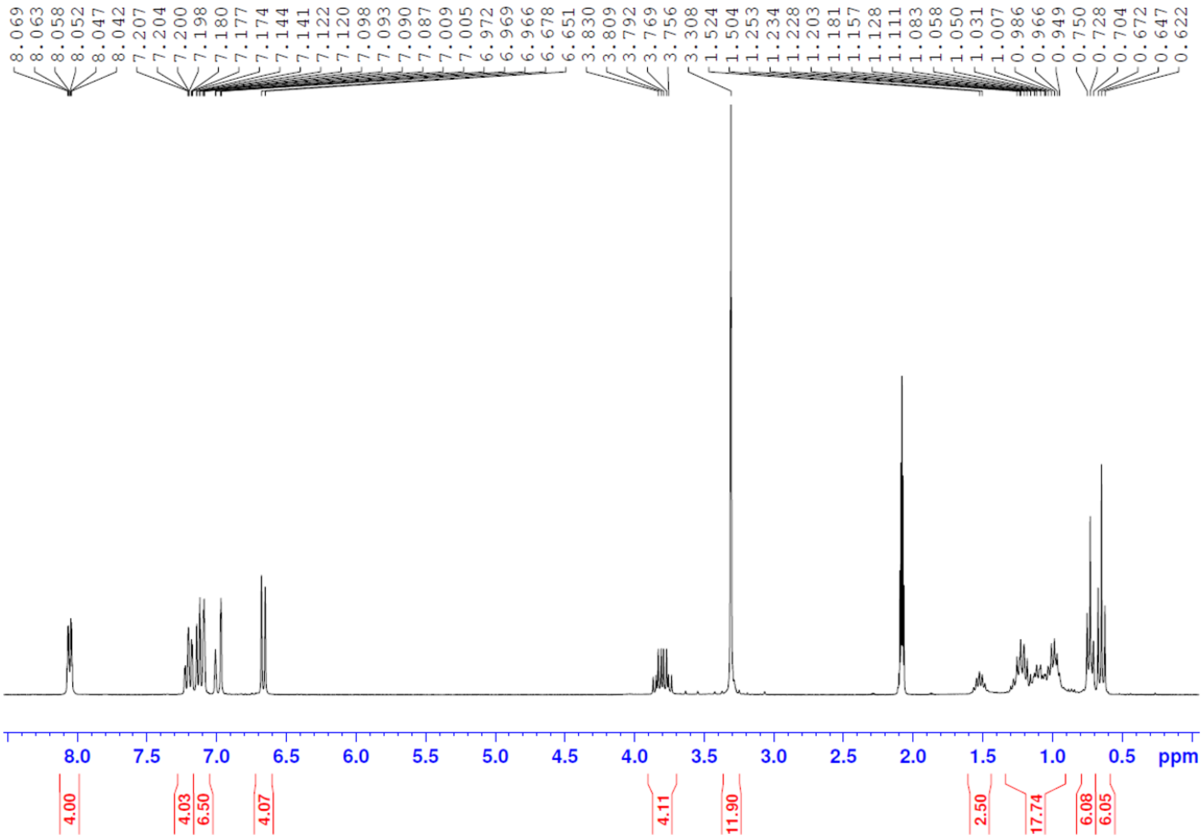

Figure S1. ^{1}H-NMR spectrum of compound **16** in D_8-Tol:

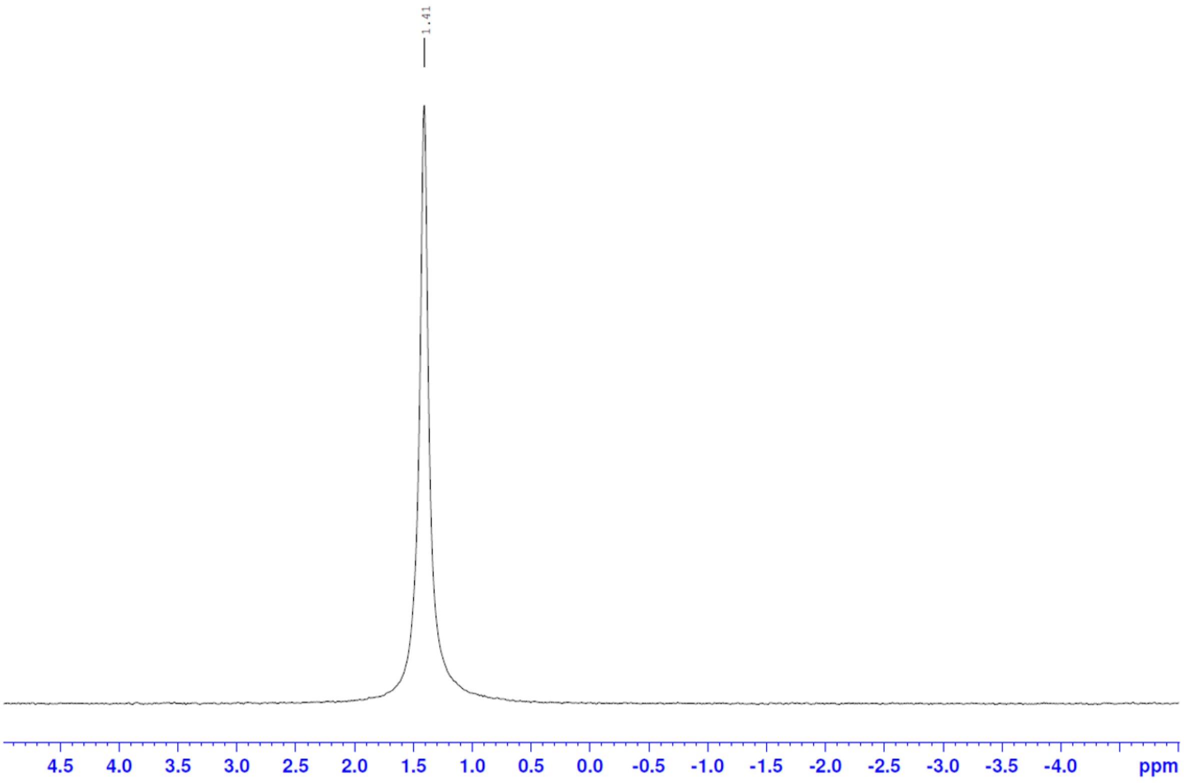

Figure S2. ^{7}Li-NMR spectrum of compound **16** in D_8-Tol.

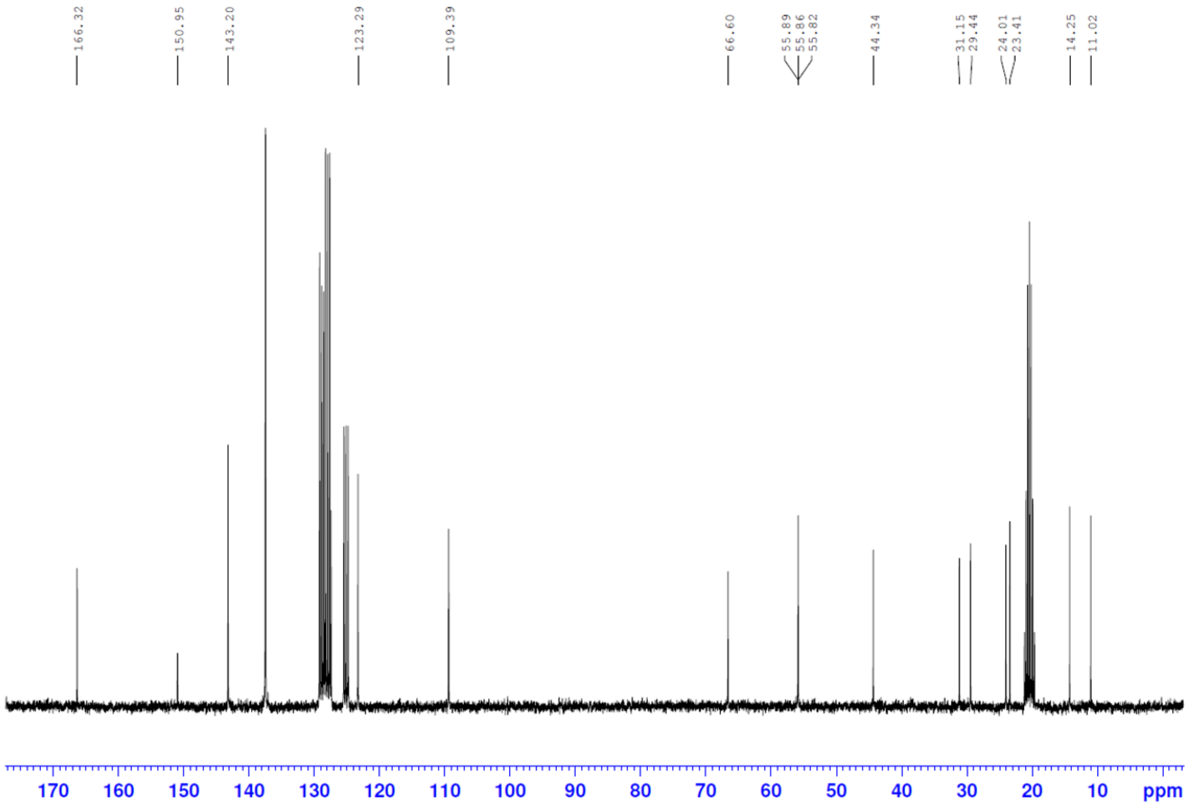

Figure S3. $^{13}C\{^{1}H\}$-NMR spectrum of compound **16** in D_8-Tol.

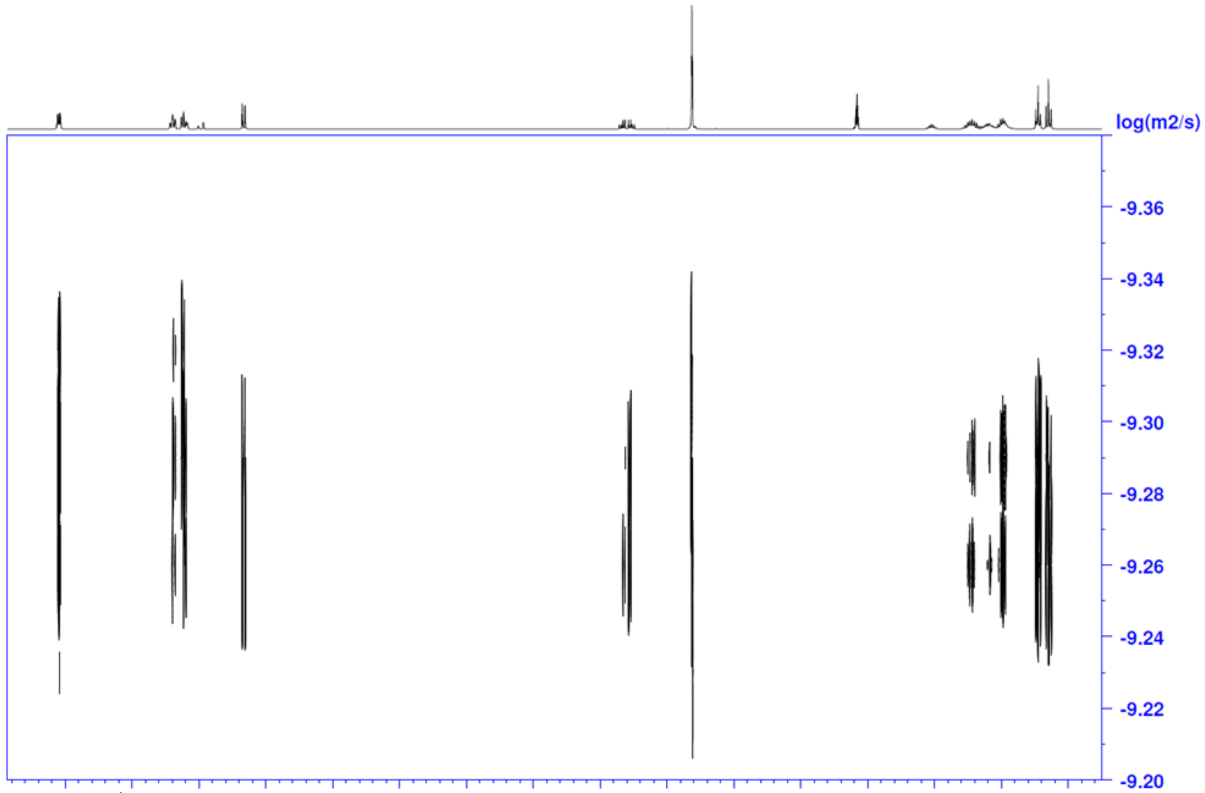

Figure S4. ^{1}H-DOSY NMR spectrum of compound **16** in D_8-Tol.

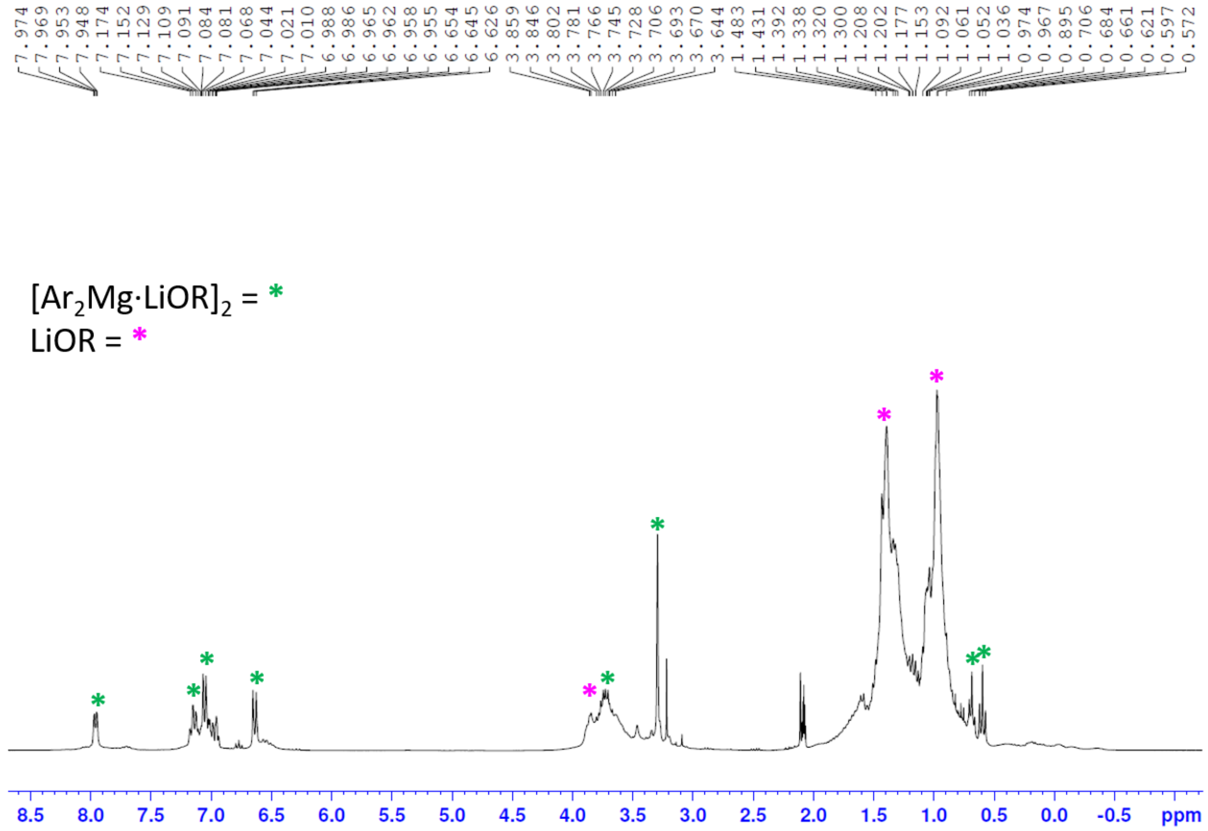

Figure S6. ^{1}H-NMR spectrum of filtrate of compound **16** in D8-Tol.

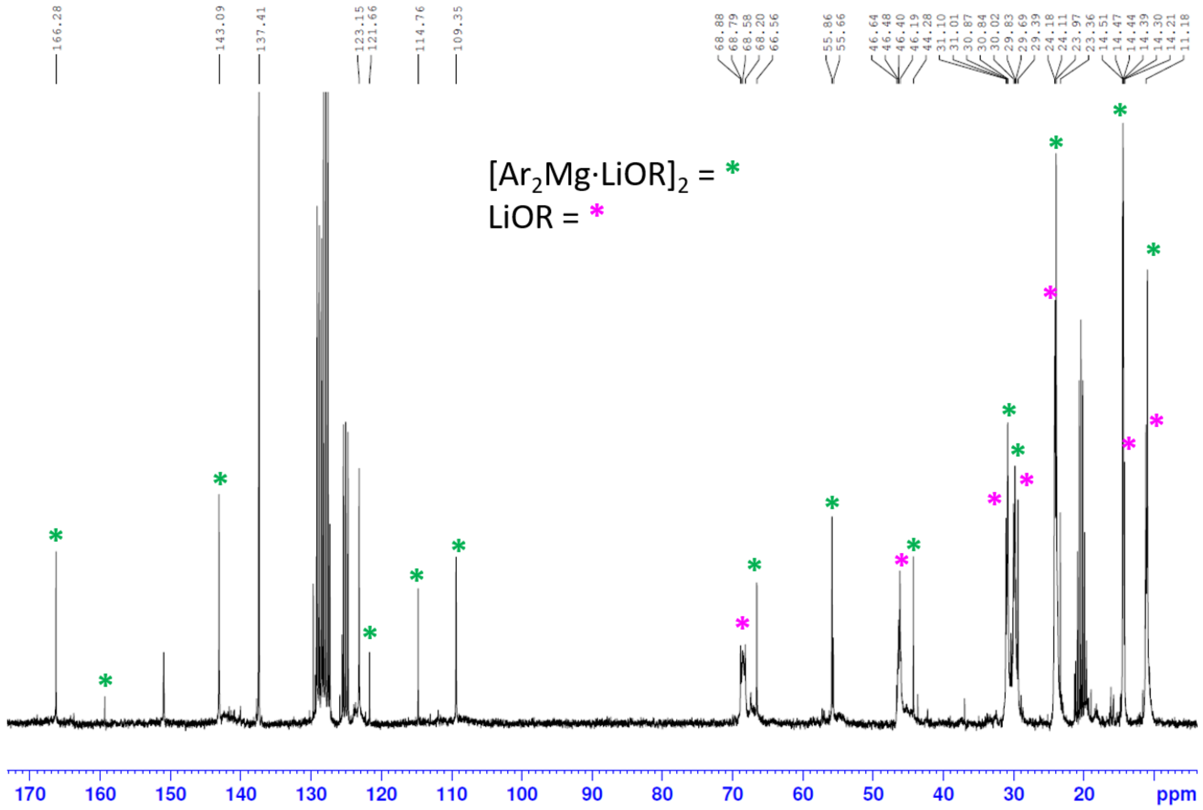

Figure S5. ^{13}C{^{1}H}-NMR spectrum of filtrate of compound **16** in D8-Tol.

NMR Monitoring of Br/Mg-exchange of 2-bromoanisole

In an argon-flushed *Schenk*-flask, **1c** was prepared from 1.00 mmol of *n*Bu_2Mg *via* Method A – a 1.00 M solution was made in D_8-Tol and stored at -30 °C inside the glovebox. A 0.1 mL aliquot of this solution was then diluted to 0.5 mL in a J. Young's NMR tube to give a 0.20 M solution. To this, 0.20 mmol (0.25 mL) of 2-bromoansiole was then added at room temperature and the reaction monitored by NMR spectroscopy to give quantitative conversion to compound **16** [Ar_2(OR)MgLi] after 30 min at room temperature with concomitant formation of *s*BuBr. Analysis of the $^{13}C\{^1H\}$ NMR spectrum after completion of exchange showed the presence of one equivalent of uncoordinated LiOR.

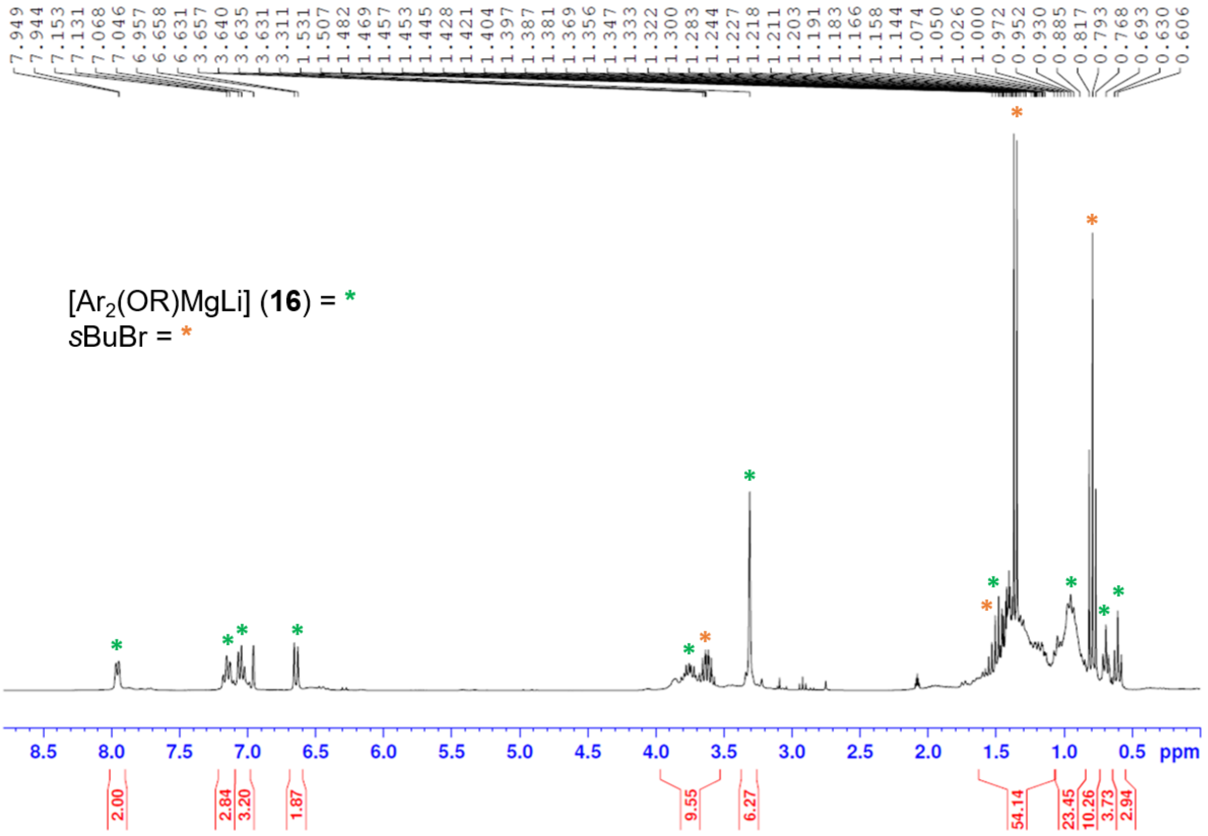

Figure S7. ^1H-NMR spectrum (D_8-Tol) of *in situ* Br/Mg-exchange of 2-bromoanisole showing formation of compound **16** and *s*BuBr.

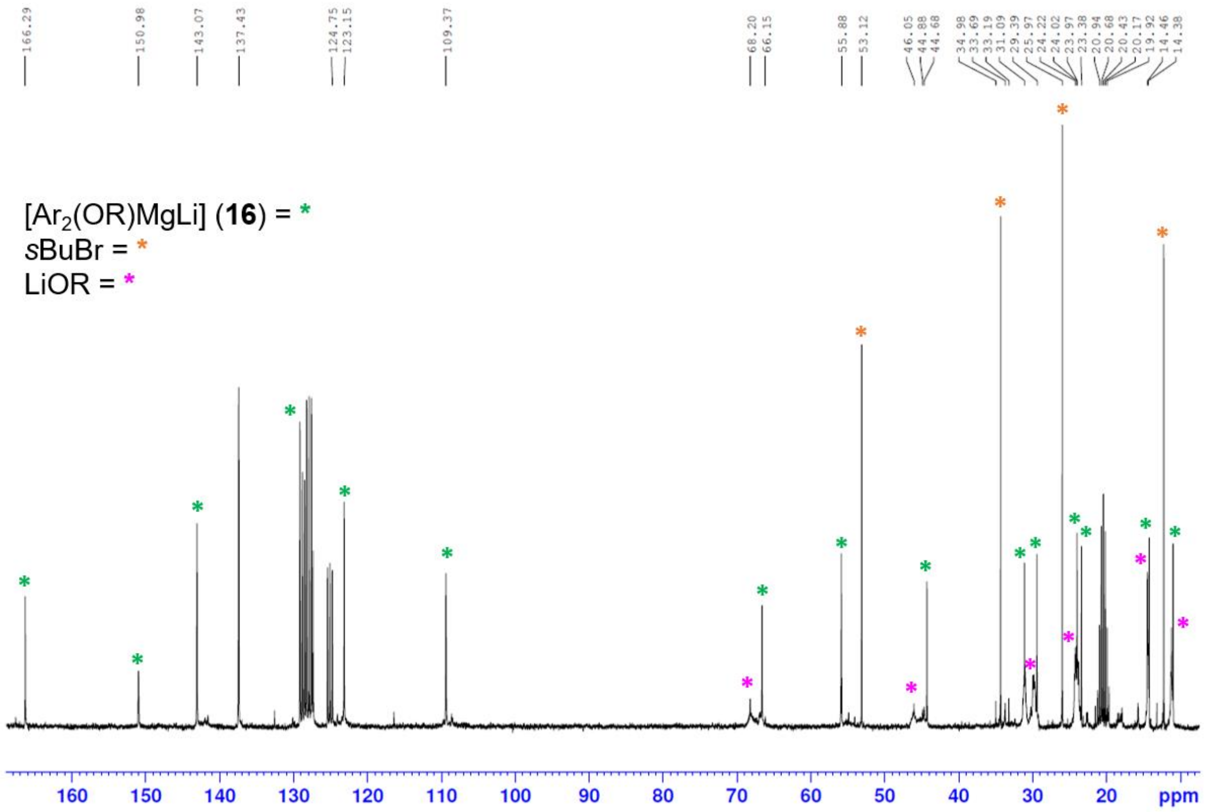

Figure S8. $^{13}C\{^{1}H\}$-NMR spectrum (D_8-Tol) of *in situ* Br/Mg-exchange of 2-bromoanisole showing formation of compound **16**, *s*BuBr and uncomplexed LiOR.

NMR Monitoring of Br/Mg-exchange of 2,5-dibromopyridine (10a)

In an argon-flushed *Schlenk*-flask, **1c** was prepared from 1.00 mmol of *n*Bu_2Mg *via* Method A – a 1.00 M solution was made in D_8-Tol and stored at -30 °C inside the glovebox. A 0.1 mL aliquot of this solution was then added to a J. Young's NMR tube and cooled to -20 °C. To this, 0.20 mmol (48 mg) of 2,5-dibromopyridine (**10a**) (pre-dissolved in 0.4 mL of D_8-Tol) was then added at -20 °C and the reaction was held at this temperature for 30 min. Complete NMR spectroscopic characterization was then conducted at -20 °C due to the temperature sensitive nature of the product. Analysis of the resultant spectra showed selective C(2)-Br/Mg-exchange to generate **11a** with a new C-Mg bond at δ 203.6 ppm.

^{1}H-NMR (300.1 MHz, D_8-Tol, ppm): δ = 8.67 (d, 2H, C_6-*H*), 7.61 (d, 2H, C_4-*H*), 7.28 (d, 2H, C_3-*H*), 3.82 (br. m, 4H, OC*H*$_2$, OR), 3.56 (m, 2H, C*H*, *s*BuBr), 1.47 (m, C*H*$_2$, *s*BuBr), 1.33 (d, C*H*$_3$, *s*BuBr), 0.79 (t, C*H*$_3$, *s*BuBr), 1.71-0.61 (br. m C*H*$_2$ + C*H*$_3$ of OR – poorly defined as product is thermally unstable).

^{7}Li-NMR (156 MHz, D_8-Tol, ppm): δ = 2.39, 2.29, 1.03 [Ar_2Mg·*Li*OR]$_2$.

^{13}C{^{1}H}-NMR (75.5 MHz, D_8-Tol, ppm): δ = 203.6 (C_q-Mg), 149.4 (*C*$_6$-H), 137.2 (*C*$_4$-H), 129.1 (*C*$_3$-H), 118.4 (*C*$_5$-Br), 67.8 (O*C*H$_2$, LiOR), 65.1 (O*C*H$_2$, OR, **11a**), 53.5 (*C*H$_2$, *s*BuBr), 45.9 (*C*H, LiOR), 44.0 (*C*H, OR, **11a**), 34.1 (*C*H, *s*BuBr), 31.0 (*C*H$_2$, OR, **11a** + LiOR), 29.2 (*C*H$_2$, OR, **11a** + LiOR), 26.0 (*C*H$_3$, *s*BuBr), 24.0 (*C*H$_2$, OR, **11a** + LiOR), 23.4 (*C*H$_2$, OR, **11a** + LiOR), 14.6 (*C*H$_3$, OR, LiOR), 14.3 (*C*H$_3$, OR, **11a**), 12.4 (*C*H$_3$, *s*BuBr), 11.0 (*C*H$_3$, OR, **11a** + LiOR).

^{1}H-DOSY NMR spectroscopy revealed co-diffusion of the aryl and alkoxy-assigned peaks, suggesting that in toluene solution they are part of the same molecular entity. A mean diffusion coefficient of $D = 4.349 \times 10^{-10}$ m^2/s.

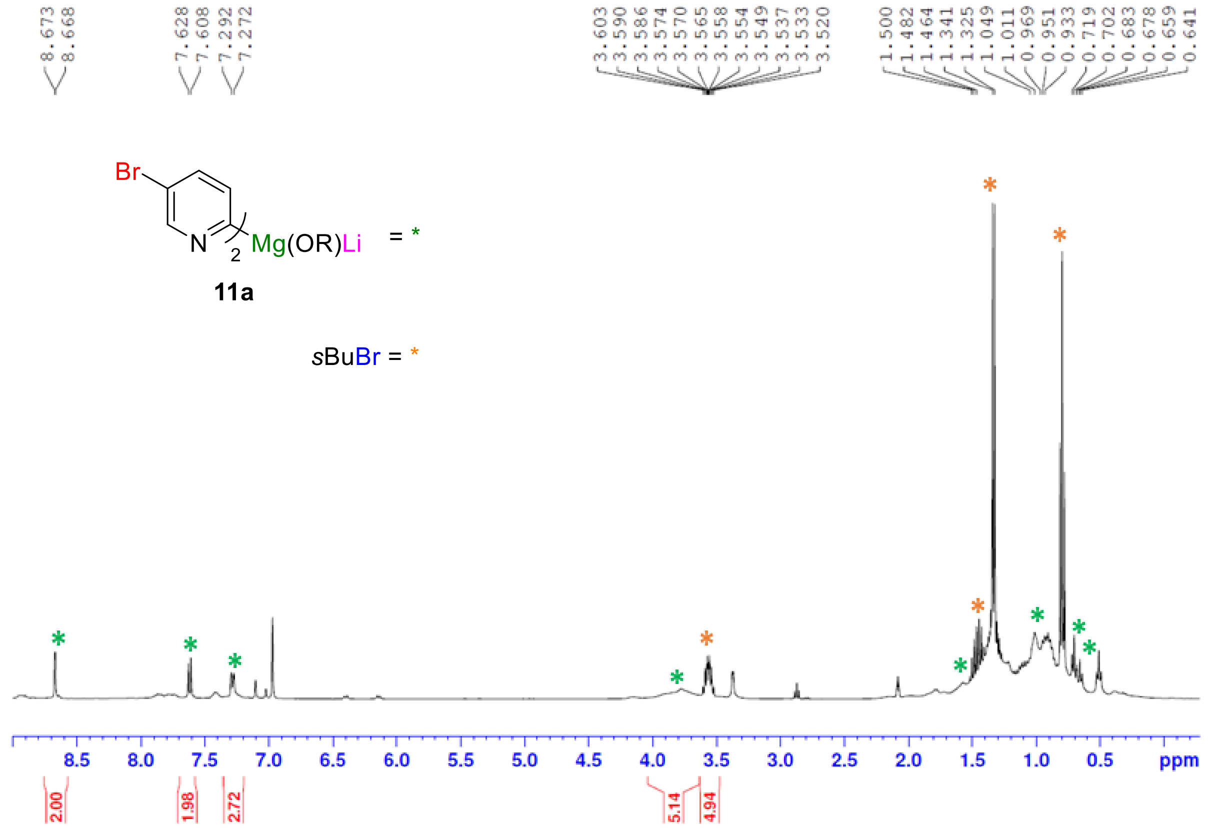

Figure S9. ^{1}H-NMR spectrum (D_8-Tol) of *in situ* Br/Mg-exchange of 2,5-dibromopyridine (**10a**) to give selective C(2)-Br exchange resulting in the formation of **11a**.

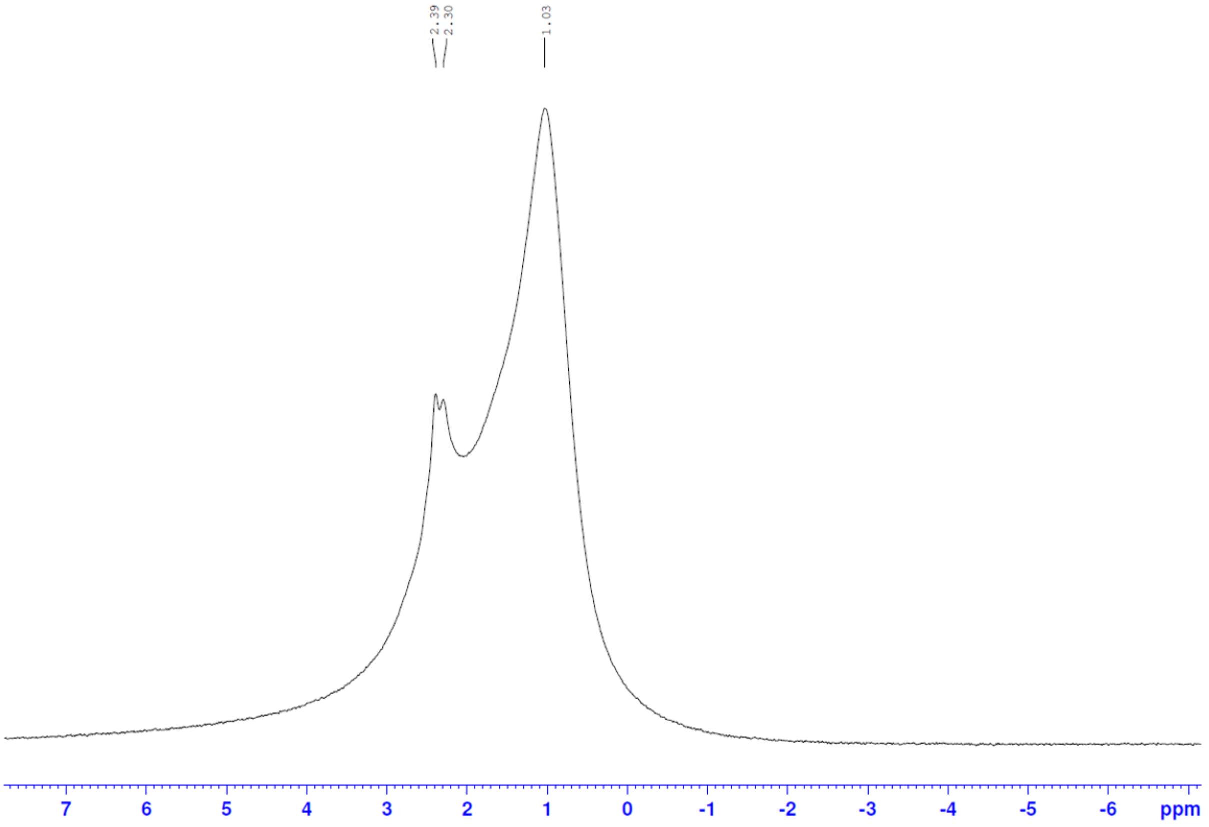

Figure S10. ^{7}Li-NMR spectrum (D_8-Tol) of *in situ* Br/Mg-exchange of 2,5-dibromopyridine (**10a**) to give selective C(2)-Br exchange resulting in the formation of **11a**.

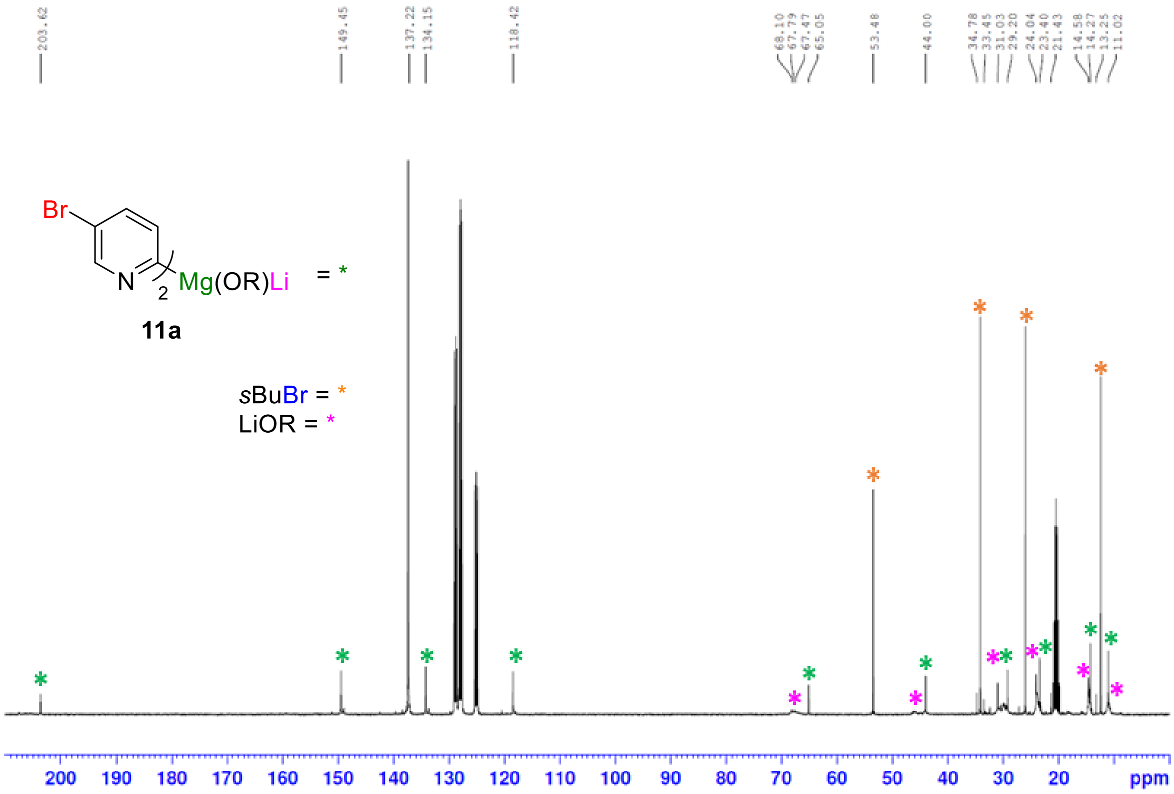

Figure S11. $^{13}C\{^{1}H\}$-NMR spectrum (D_8-Tol) of *in situ* Br/Mg-exchange of 2,5-dibromopyridine (**10a**) to give selective C(2)-Br exchange resulting in the formation of **11a** with release of LiOR.

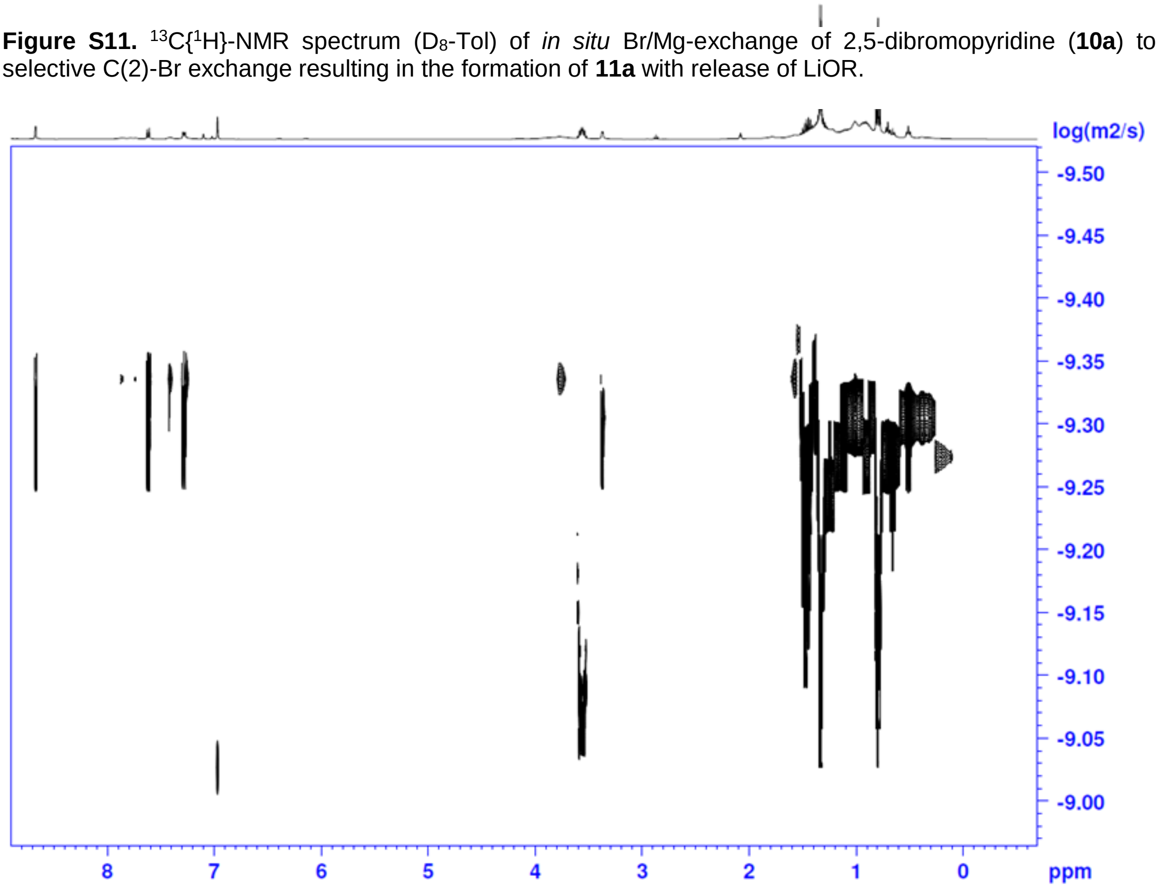

Figure S12. ^{1}H-DOSY NMR spectrum (D_8-Tol) of *in situ* Br/Mg-exchange of 2,5-dibromopyridine (**10a**) to give selective C(2)-Br exchange. Final aryl and alkoxide signals co-diffuse with a mean diffusion coefficient of D = 4.394x10^{-10} m^{2}/s.

Synthesis of LiOR (R = 2-ethylhexyl)

In an argon-filled *Schlenk*-tube, 1.00 mmol of *n*BuLi (0.63 mL, 1.60 M) was added to 5 mL of dry hexane and cooled to 0 °C. To this, 0.16 mL of ROH was added and the mixture was then allowed to stir at room temperature for 1 h. Removal of all volatiles under reduced pressure resulted in a colourless oil – LiOR.

^{1}H-NMR (300.1 MHz, D$_8$-Tol, ppm): δ = 3.96-3.68 (br. m, 2H, OC*H*$_2$), 1.70 (br. m, 1H, C*H*$_2$ x1, Et),‡ 1.46 (br. s, 8 H, C*H*$_2$ x1 (Et) + C*H* + (C*H*$_2$)$_3$, OR), 1.09 (br. t, 3H, C*H*$_3$, OR), 1.01 (br. t, 3H, C*H*$_3$, OR).

^{7}Li-NMR (156 MHz, D$_8$-Tol, ppm): δ = 0.86 (*Li*OR).

^{13}C{^{1}H}-NMR (75.5 MHz, D$_8$-Tol, ppm): δ = 68.2 (O*C*H$_2$, OR), 46.6 (OC*H*$_2$C(*H*), OR), 31.1 (*C*H$_2$, OR), 30.2 (*C*H$_2$, OR), 24.2 (*C*H$_2$, Et, OR), 24.0 (*C*H$_2$, OR), 14.5 (*C*H$_3$, Et, OR), 11.4 (*C*H$_3$, OR).

‡Confirmed by [^{1}H,^{1}H]-COSY NMR spectrum

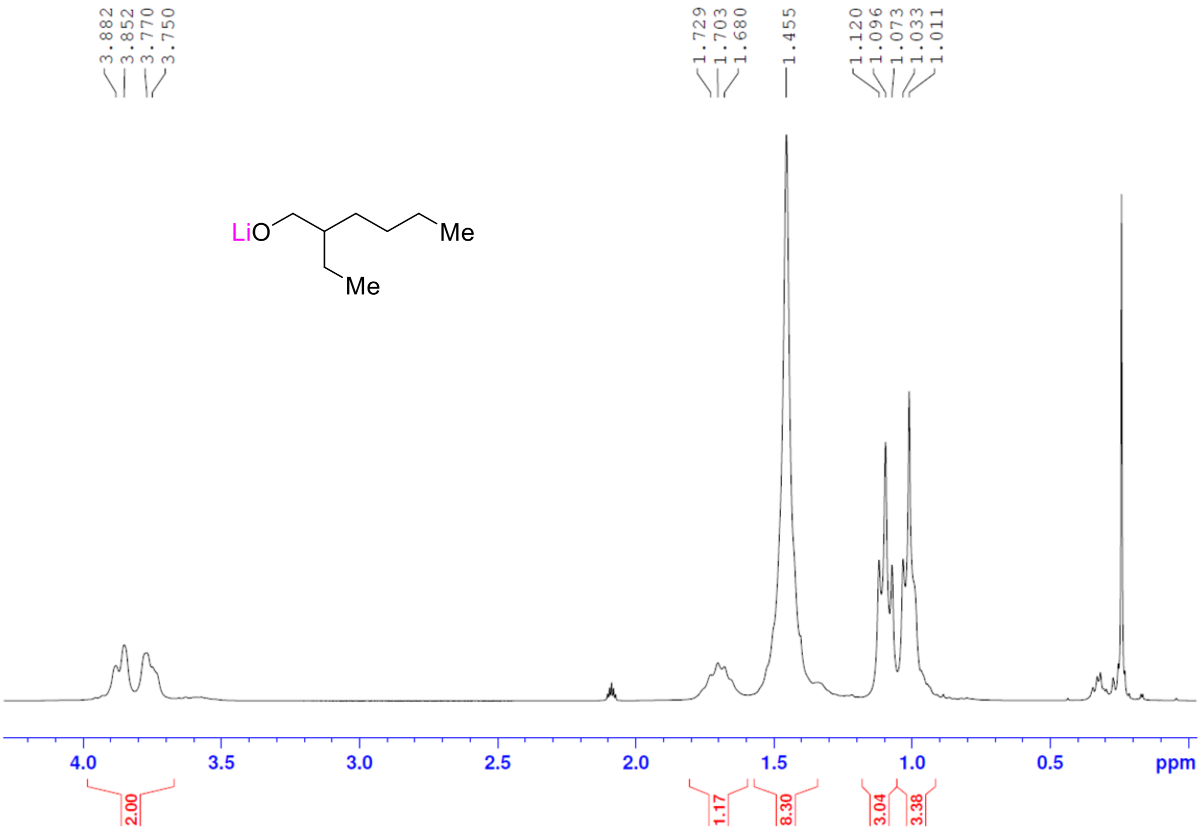

Figure S9. ^{1}H-NMR spectrum of LiOR in D$_8$-Tol.

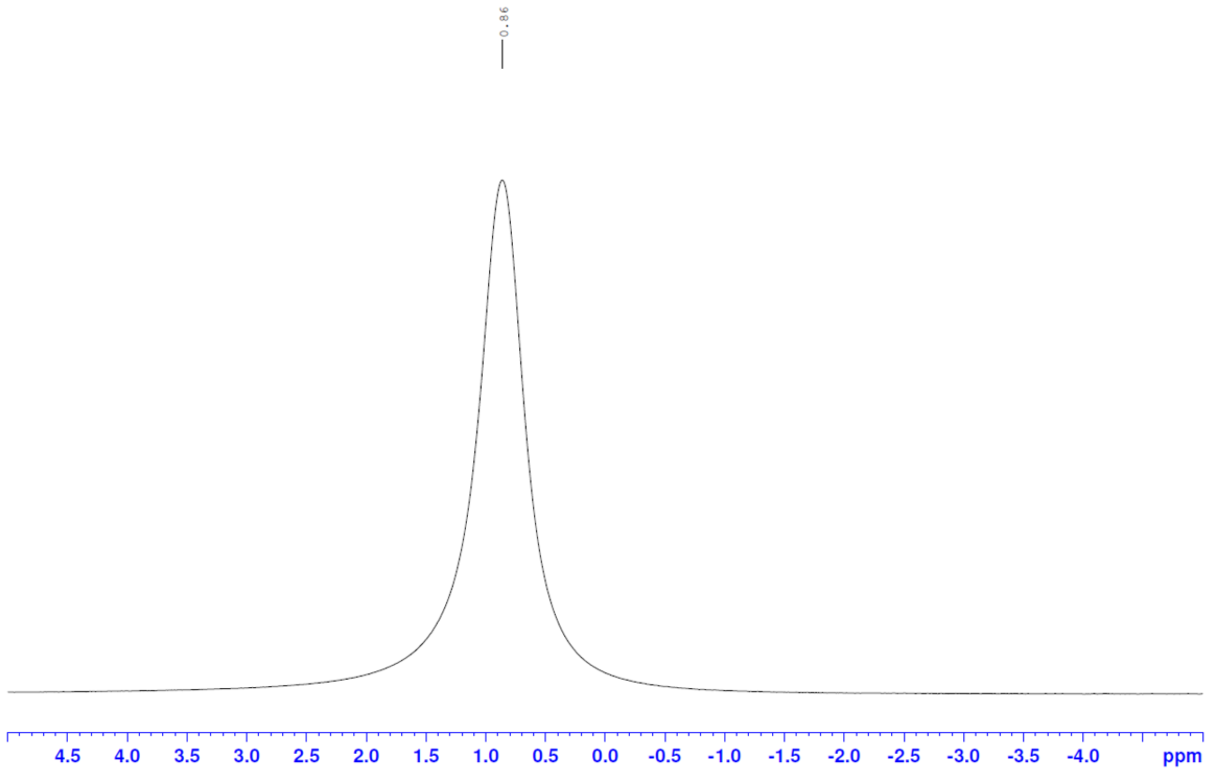

Figure S10. ^{7}Li-NMR spectrum of LiOR in D_8-Tol.

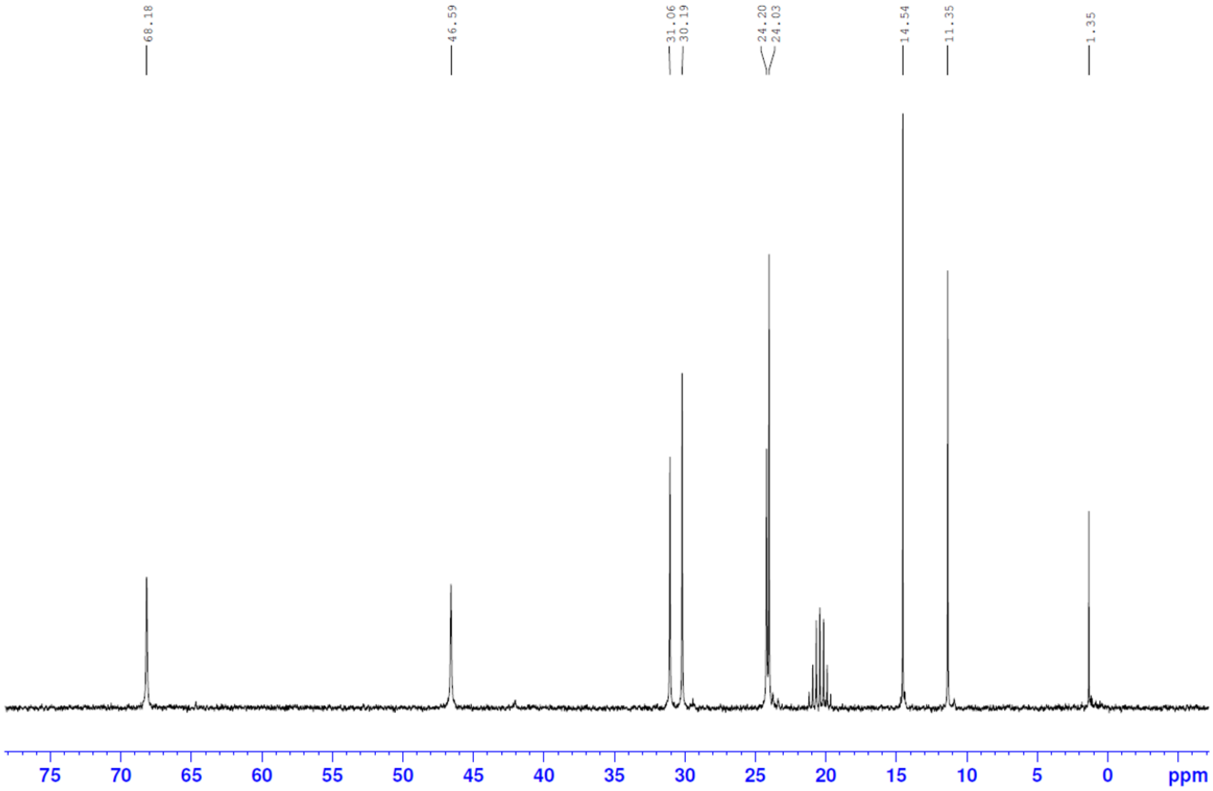

Figure S11. $^{13}C\{^{1}H\}$-NMR spectrum of LiOR in D_8-Tol.

Control Experiments

Synthesis of *s*Bu_2Mg

To an argon-flushed 750 mL *Schlenk*-flask, 100 mL of dry Et_2O was added followed by 40.0 mmol of *s*BuMgCl (20 mL, 2.00 M in Et_2O) and cooled to 0 °C in an ice bath. Taking advantage of the *Schlenk*-equilibrium, 20.0 mmol of 1,4-dioxane (1.7 mL) was then added dropwise, resulting in a thick, white suspension which was stirred at 0 °C overnight. Filtration of this suspension through a plug of cellite and glasswool resulted in a colourless solution of *s*Bu_2Mg in Et_2O. All Et_2O was then removed under reduced pressure to give a pale yellow oil which was then re-dissolved in 20 mL of dry hexane. The dialkylmagnesium reagent was standardised in THF via iodometric titration[3] – typical concentration 0.41 M.

Note: This reagent is highly temperature sensitive and requires continuous storage at -30 °C or below. It must be used within 3 or 4 days of synthesis to ensure purity. Et_2O present within spectra as it cannot be completely removed under vacuum.

^{1}H-NMR (300.1 MHz, D_8-Tol, ppm): δ = 3.50 (s, residual 1,4-dioxane), 3.30 (q, residual Et_2O), 1.87 (quint., 4H, C*H*$_2$, *s*Bu), 1.51 (d, 6H, C*H*$_3$, *s*Bu), 1.20 (t, 6H, C*H*$_3$, *s*Bu), 0.82 (t, residual Et_2O), 0.05 (sext., 2H, C*H*, *s*Bu).
^{13}C{^{1}H}-NMR (75.5 MHz, D_8-Tol, ppm): δ = 65.1 (residual Et_2O), 31.1 (*C*H, *s*Bu), 20.2 (*C*H$_3$, *s*Bu), 19.8 (*C*H$_2$, *s*Bu), 17.1 (*C*H$_3$, *s*Bu), 13.7 (residual Et_2O).

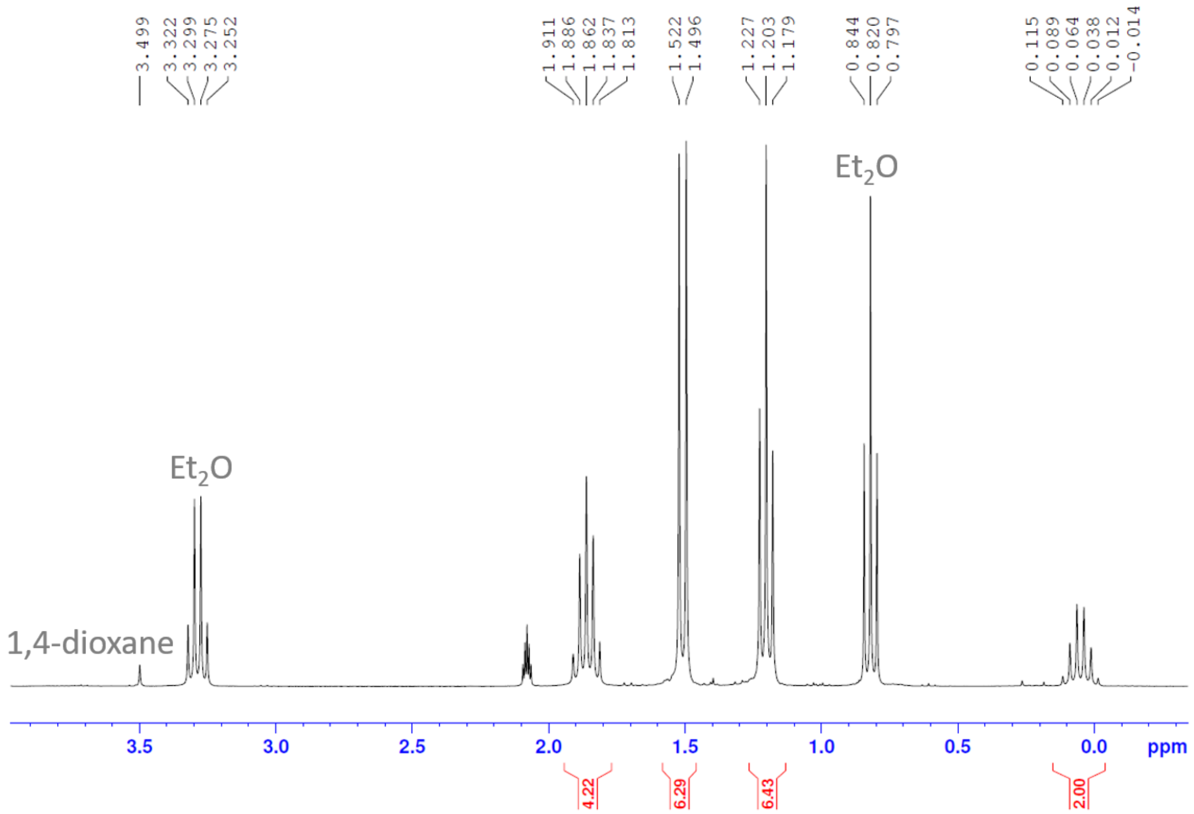

Figure S13. ^{1}H-NMR spectrum of *s*Bu_2Mg in D_8-Tol.

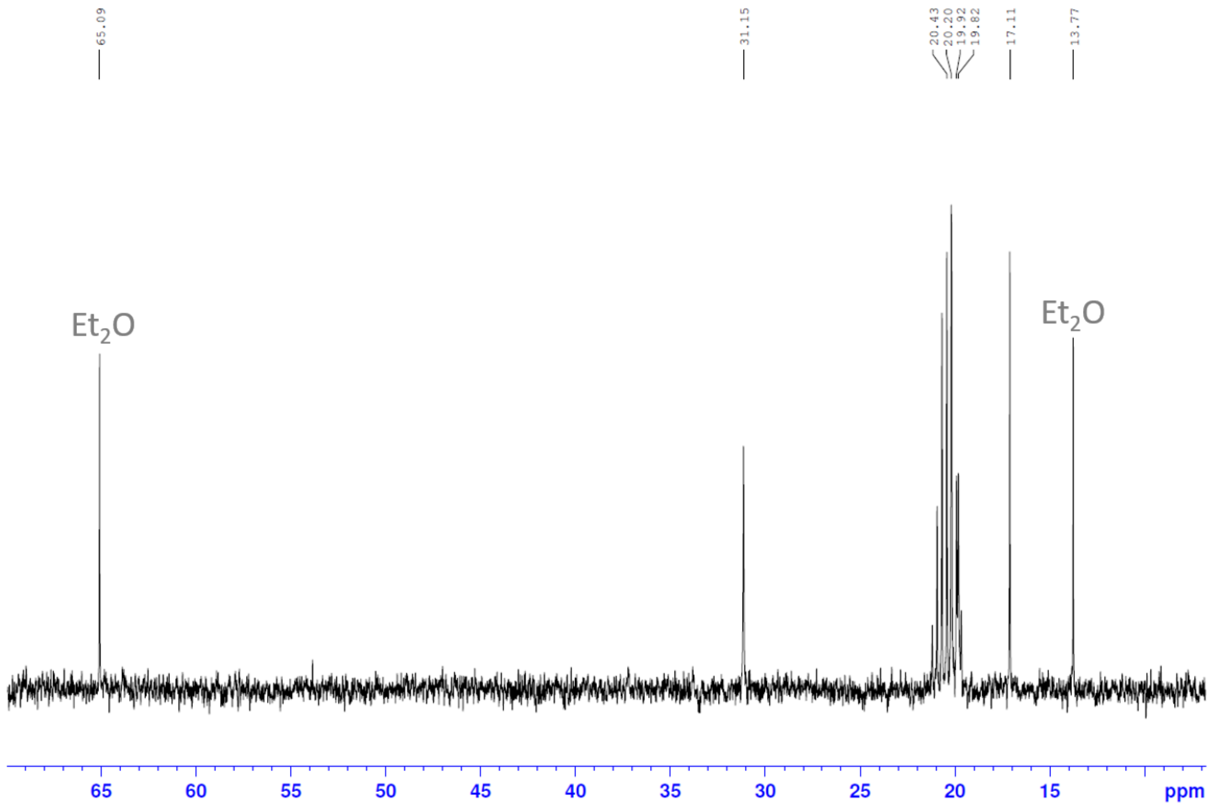

Figure S12. $^{13}C\{^{1}H\}$-NMR spectrum of *s*Bu_2Mg in D_8-Tol.

Synthesis of *s*BuMg(OR) (R = 2-ethylhexyl)

In an argon-prepared *Schlenk*-flask, 1.00 mmol of *s*Bu$_2$Mg (2.44 mL, 0.41 M in hexane) was diluted to 5 mL with dry hexane. The colourless solution was cooled to 0 °C followed by addition of one molar equivalent of 2-ethylhexanol (0.16 mL). The mixture was then warmed to room temperature and stirred for 1 h. Removal of all volatiles under reduced pressure produced a colourless oil which, by multinuclear NMR spectroscopy, proved to be heteroleptic *s*BuMg(OR).

^{1}H-NMR (300.1 MHz, D$_8$-Tol, ppm): δ = 3.87 (m, 2H, OC*H*$_2$), 1.95 (qn, 2H, C*H*$_2$, *s*Bu), 1.73 (m, 1H, C*H*, OR), 1.63 (d, 3H, C*H*$_3$, *s*Bu), 1.43-1.15 (m, 11H, C*H*$_2$ x 4, OR + C*H*$_3$, *s*Bu), 0.34 (sext., 1H, *s*Bu)

^{13}C{^{1}H}-NMR (75.5 MHz, D$_8$-Tol, ppm): δ = 68.3 (O*C*H$_2$, OR), 43.5 (*C*H, OR), 32.5 (*C*H$_2$, *s*Bu), 31.7 (*C*H$_2$, OR), 29.3 (*C*H$_2$, OR), 24.1 (*C*H$_2$, Et, OR), 23.7 (*C*H$_2$, OR), 21.4 (*C*H, *s*Bu), 18.02 (*C*H$_3$, *s*Bu), 14.3 (*C*H$_3$, Et, OR), 10.9 (*C*H$_3$, OR)

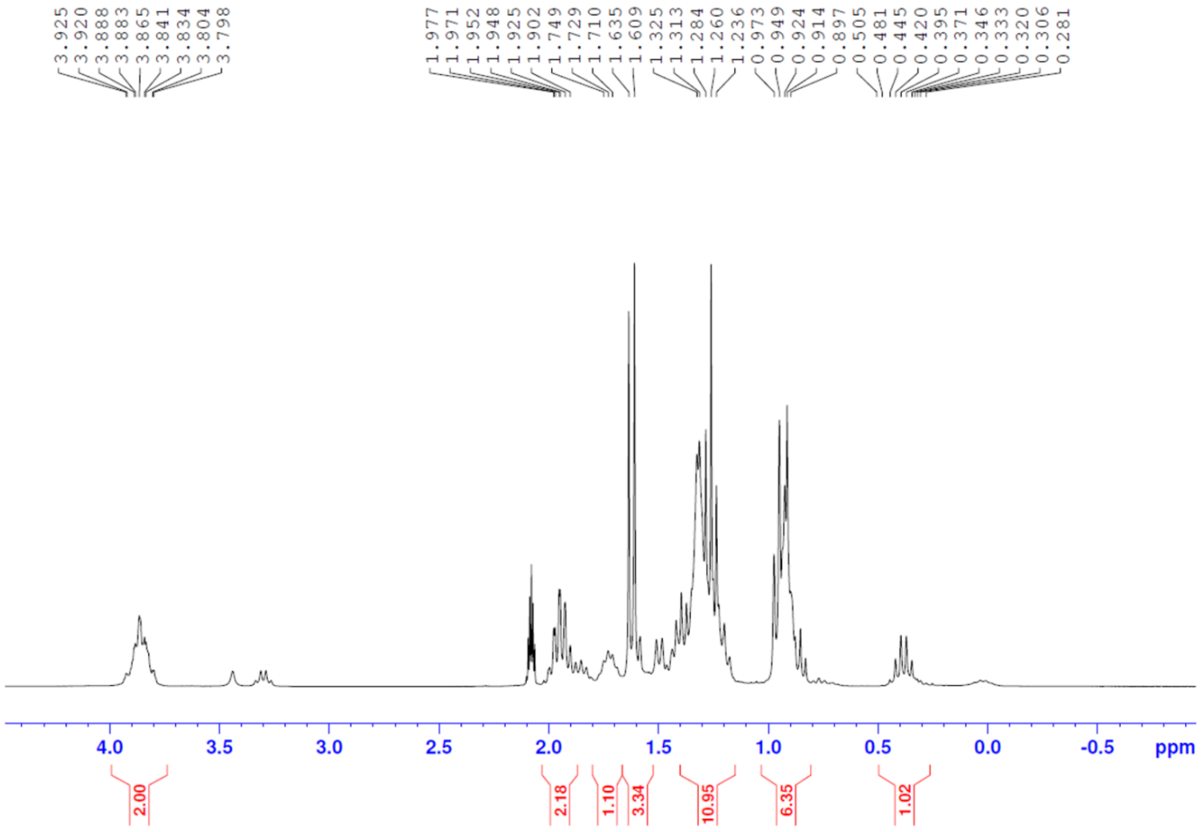

Figure S14. ^{1}H-NMR spectrum of *s*BuMgOR in D$_8$-Tol.

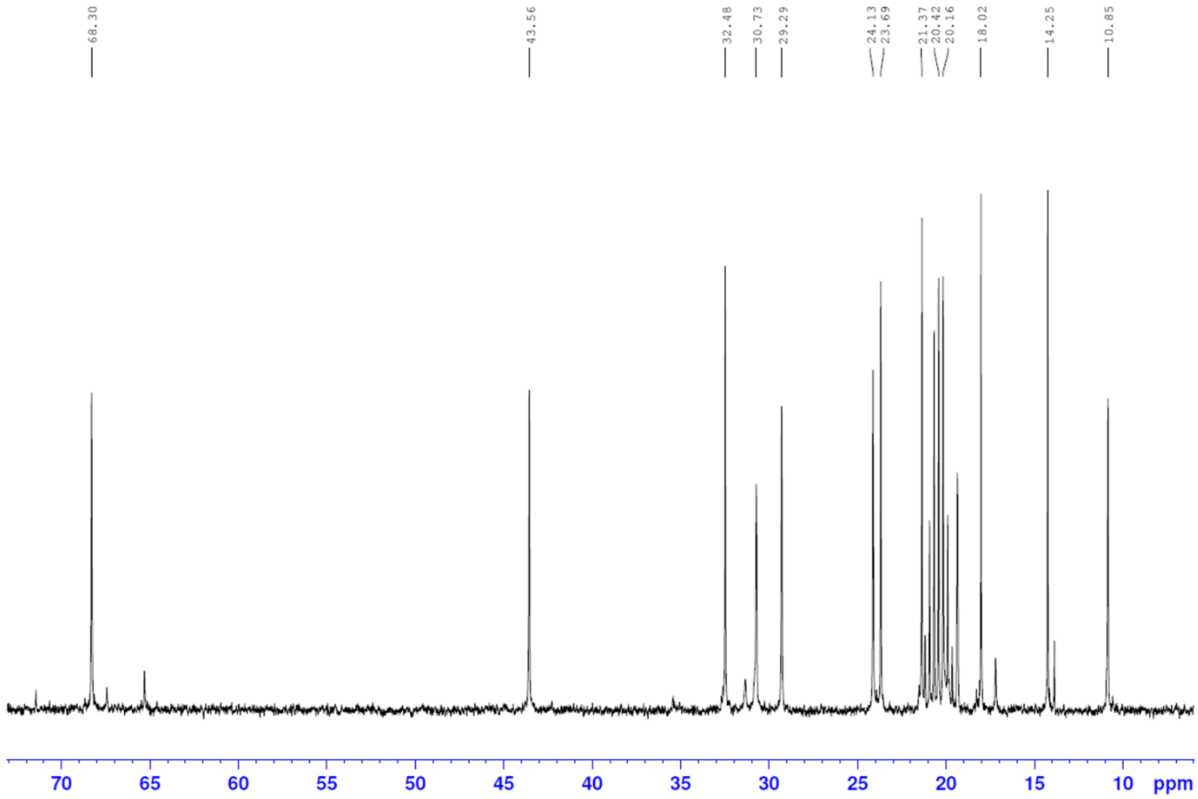

Figure S15. $^{13}C\{^{1}H\}$-NMR spectrum of *s*BuMgOR in D_8-Tol.

Control Br/Mg-Exchange Reactions of 2-bromoanisole (15)

sBu_2Mg

In an argon-flushed *Schlenk*-flask, 1.00 mmol of *s*Bu_2Mg (2.44 mL, 0.41 M in hexane) was added and then all solvent removed to give a colourless oil. The oil was reconstituted in 3.33 mL of toluene resulting in a colourless solution. To this, 1.67 mmoles of 2-bromoanisole (0.6:1 ratio of *s*Bu_2Mg:2-bromoanisole) 1.00 M solution in toluene (containing 20 mol% C_6Me_6) was added. The mixture was stirred at room temperature for 30 min before being quenched with 15 mL of sat. aq. NH_4Cl and extracted with 3 x 20 mL EtOAc. The organic layers were combined, washed with 15 mL brine and dried over Na_2SO_4. A 50 μL aliquot was diluted with 1 mL of EtOAc for analysis by GC. Yields were determined against anisole calibration curve.

*s*BuMgOR

In an argon-flushed *Schlenk*-flask, 1.00 mmol of *s*BuMg(OR) was added and then all solvent prepared as described and then all solvent removed under reduced pressure to give a colourless oil. The oil was reconstituted in 4.17 mL of toluene resulting in a colourless solution. To this, 0.83 mmoles of 2-bromoanisole (0.6:1 ratio of *s*BuMgOR:2-bromoanisole) 1.00 M solution in toluene (containing 20 mol% C_6Me_6) was added. The mixture was stirred at room temperature for 30 min before being quenched with 15 mL of sat. aq. NH_4Cl and extracted with 3 x 20 mL EtOAc. The organic layers were combined, washed with 15 mL brine and dried over Na_2SO_4. A 50 μL aliquot was diluted with 1 mL of EtOAc for analysis by GC. Yields were determined against anisole calibration curve.

Table S5. Table of GC yield of anisole from control reactions of *s*Bu_2Mg and *s*BuMg(OR) with 2-bromoanisole.

Exchange Reagent	Yield of Anisole [%]
sBu_2Mg	5
*s*BuMg(OR)	6

NMR Spectra of Compounds 2, 5-6, 9-10, 13-14, 16-17, SM1-4 and A-D

^{1}H and ^{13}C NMR spectra of compound **2b**

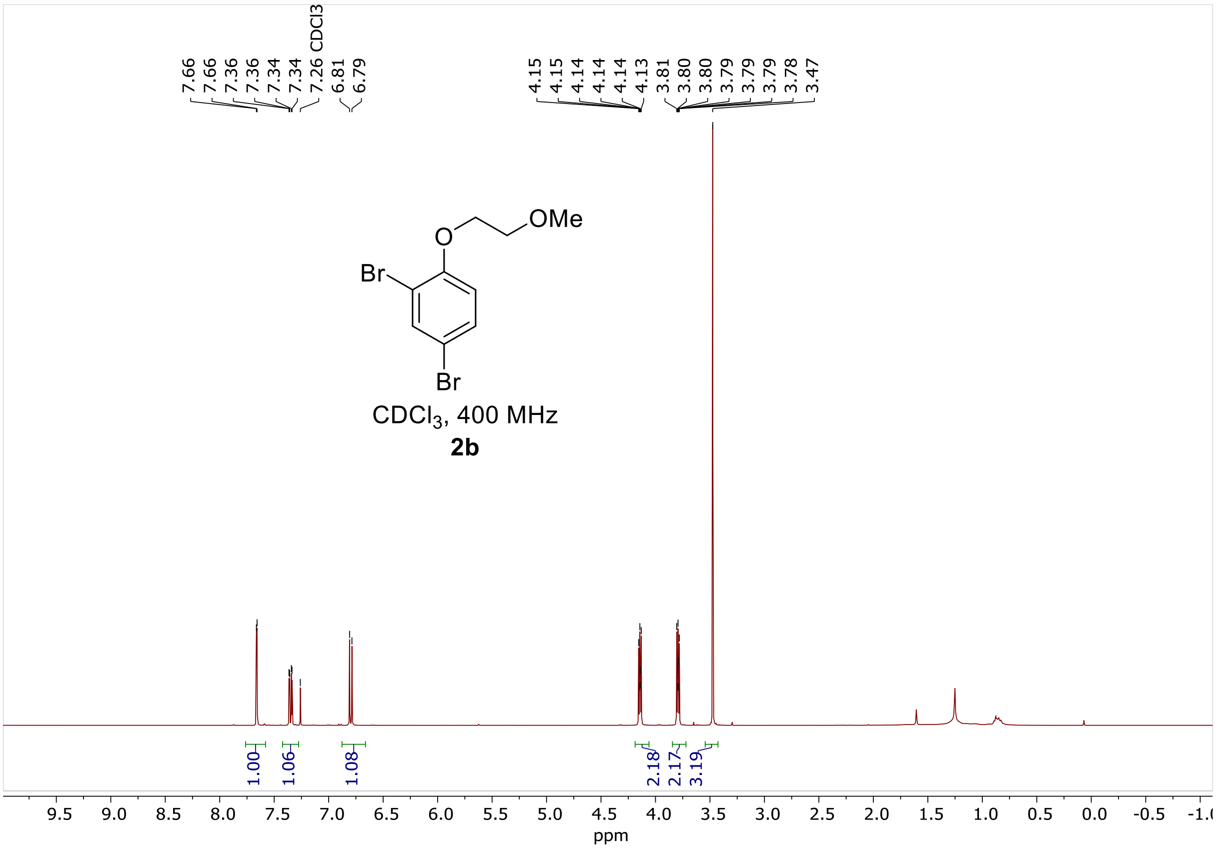

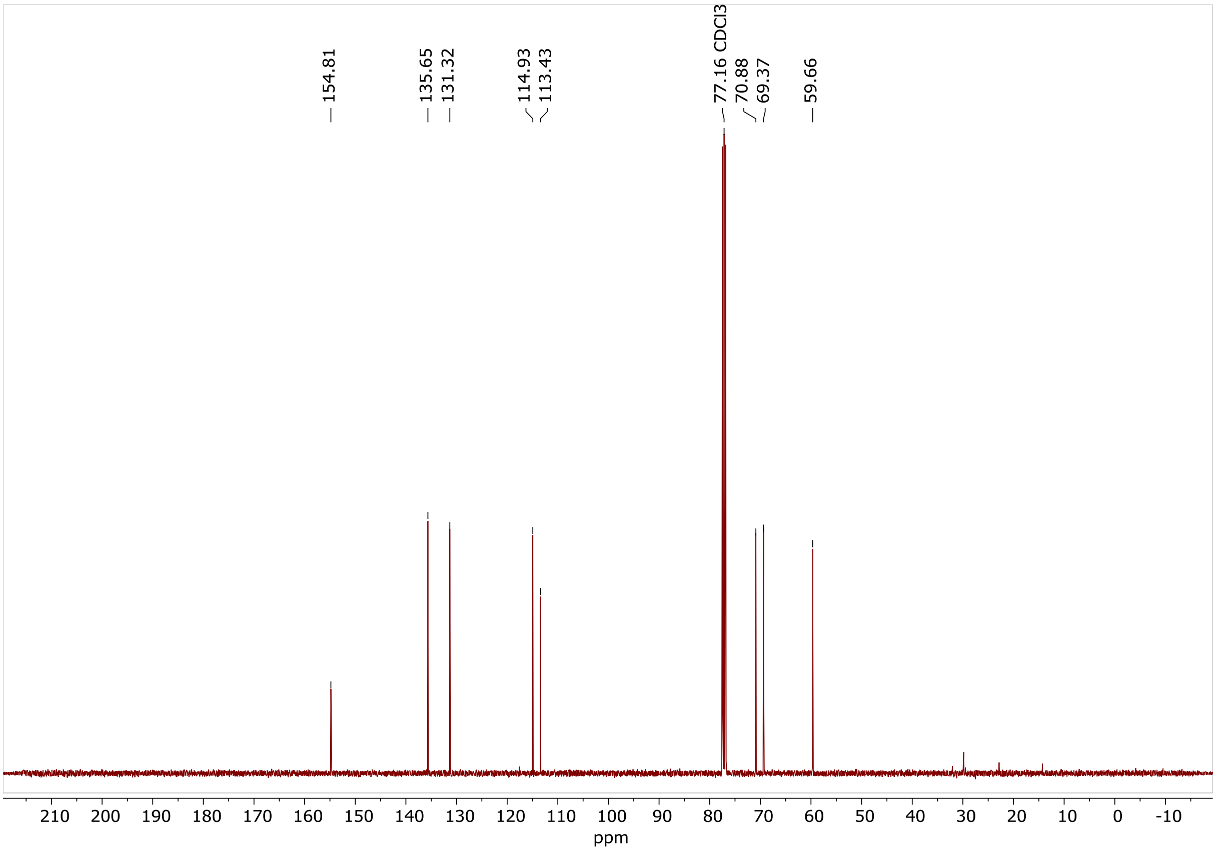

^{1}H and ^{13}C NMR spectra of compound **2c**

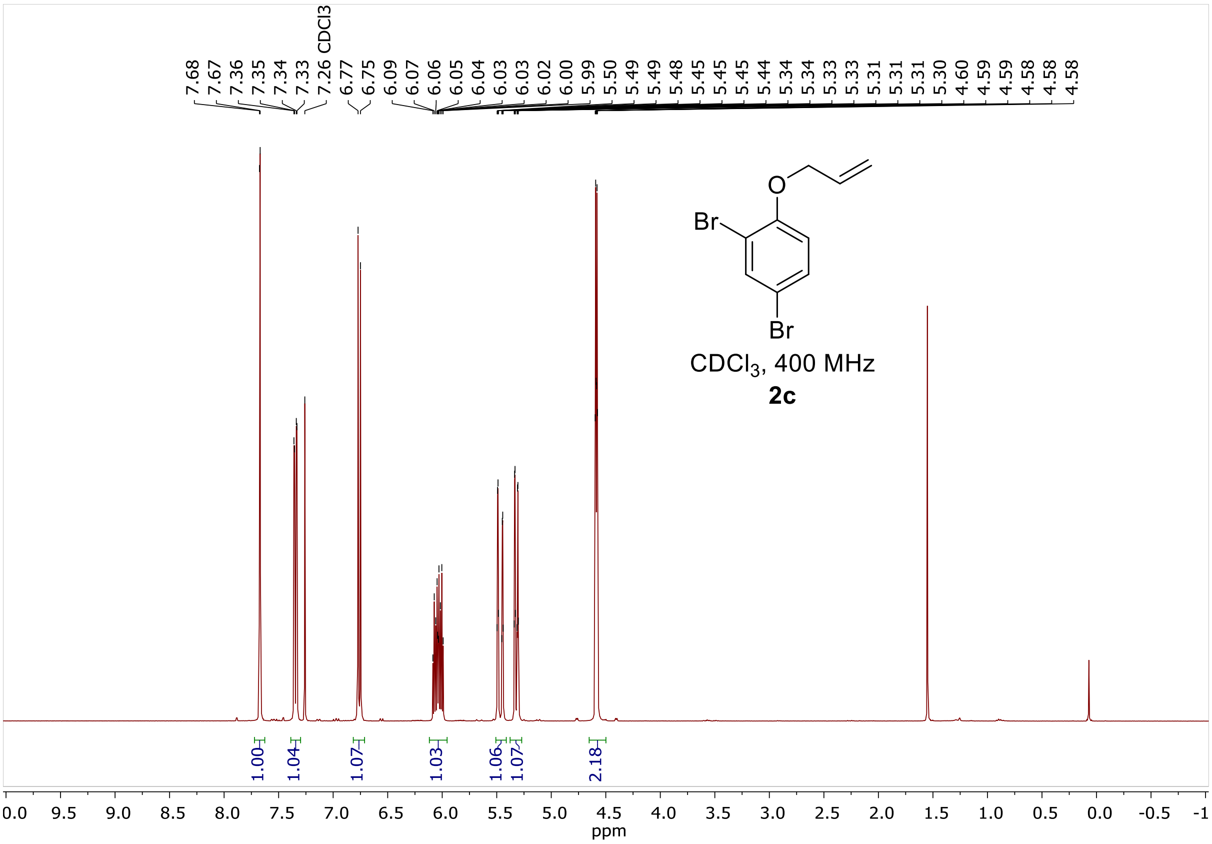

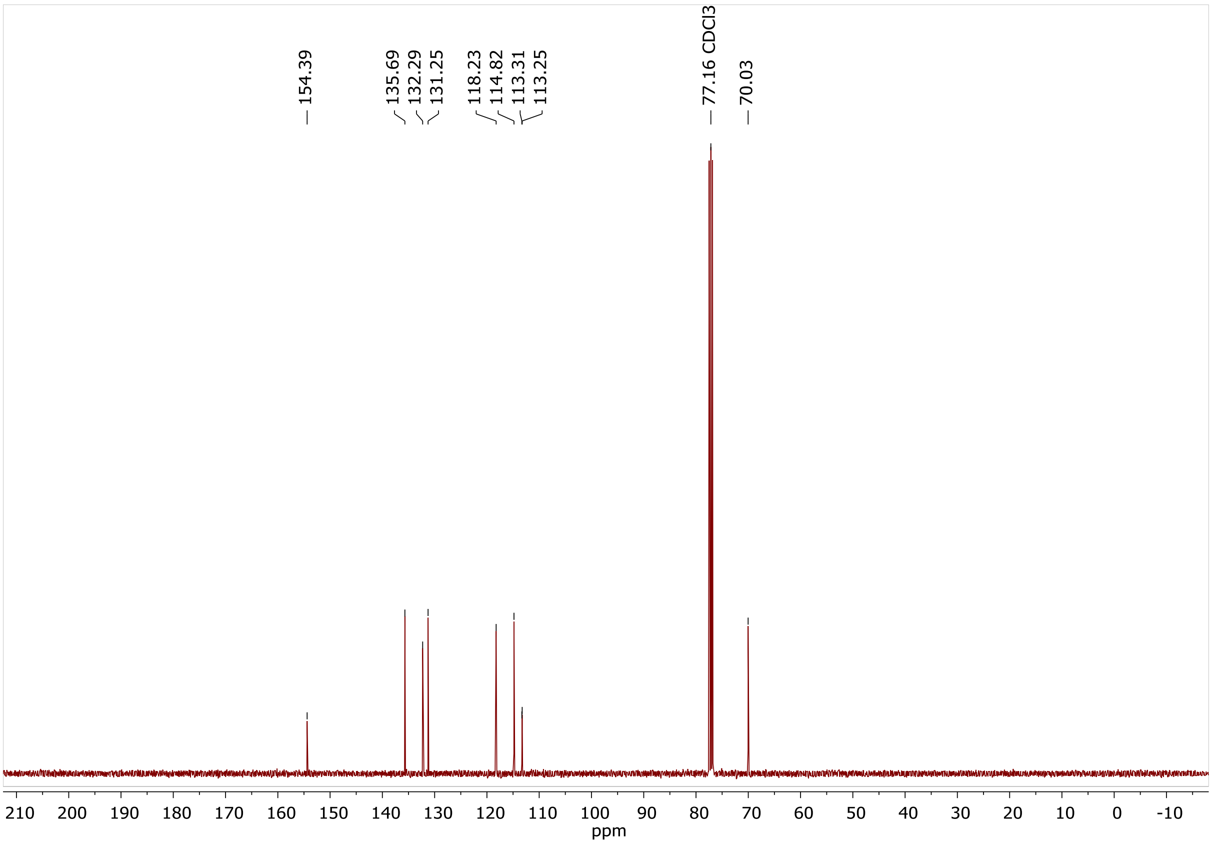

^1H and ^{13}C NMR spectra of compound **6c**

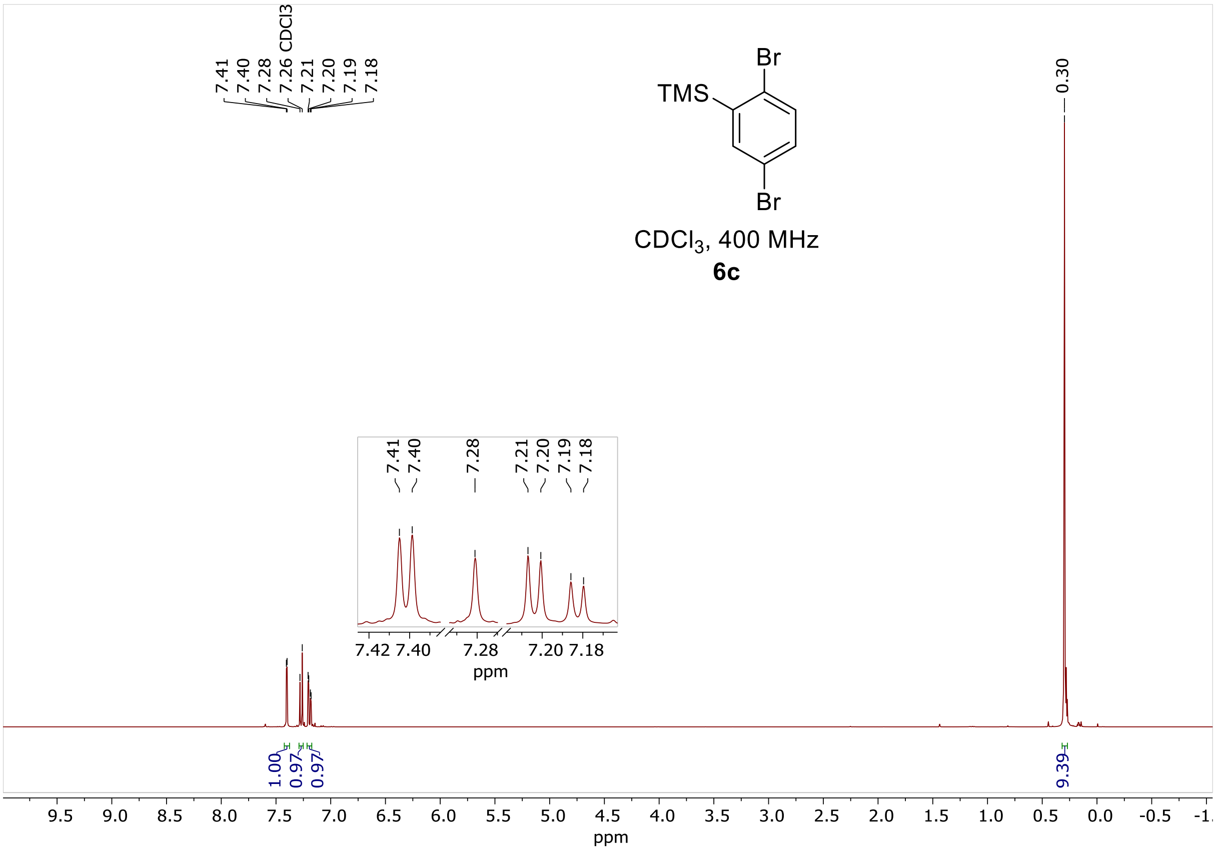

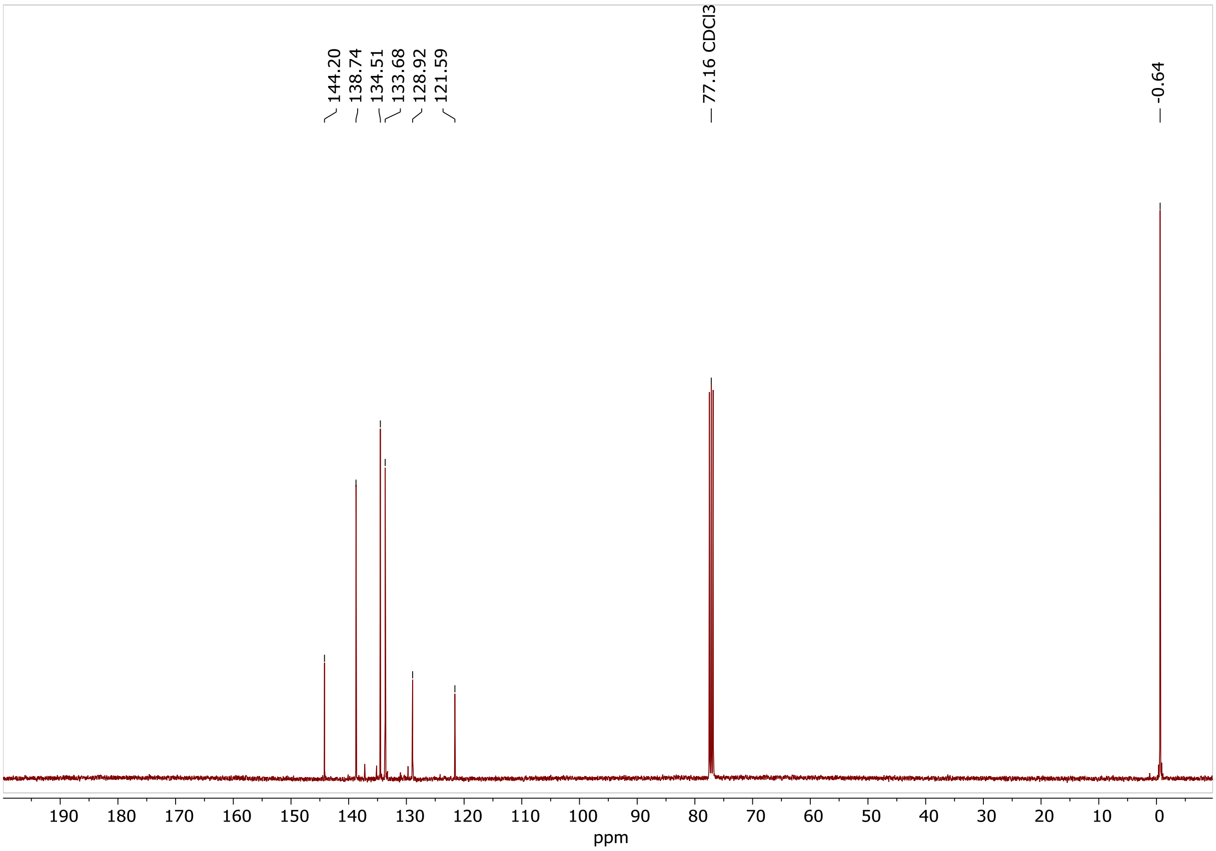

^{1}H, ^{13}C and ^{19}F NMR spectra of compound **10e**

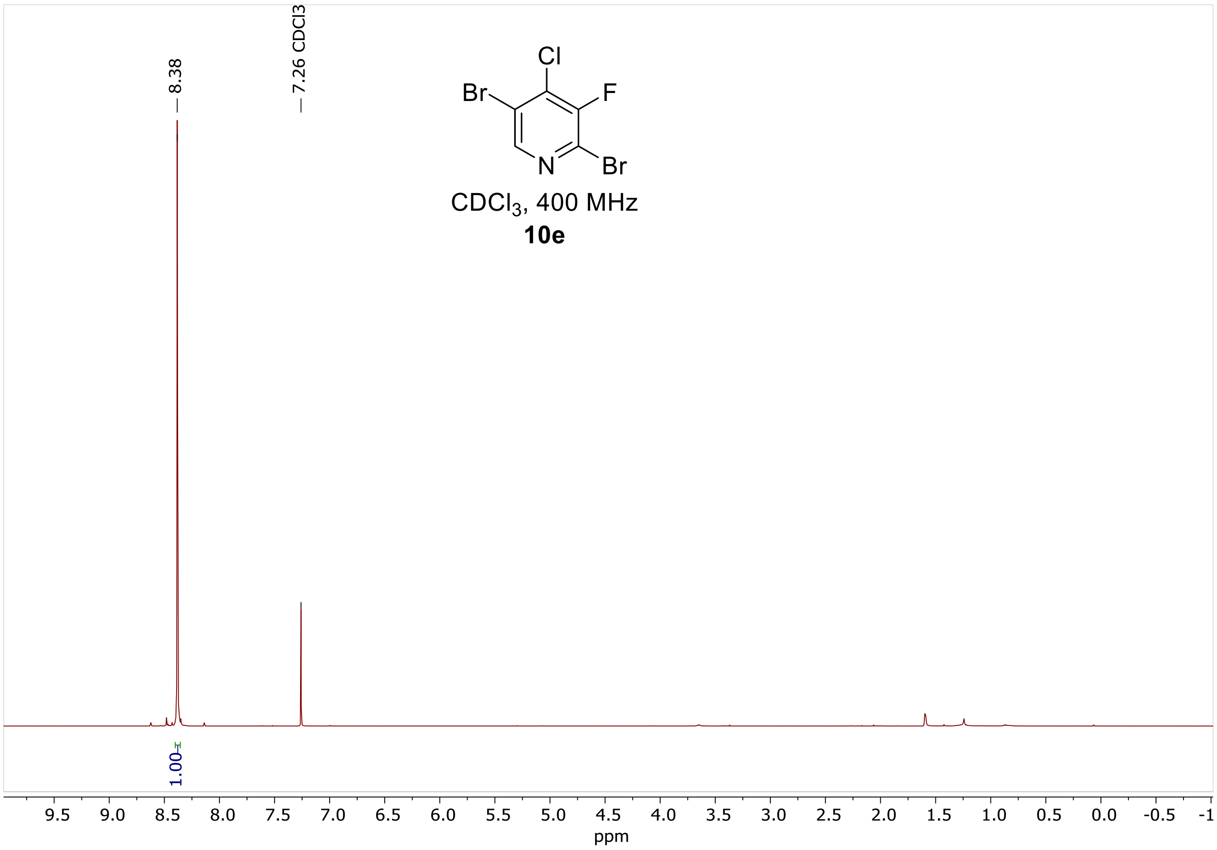

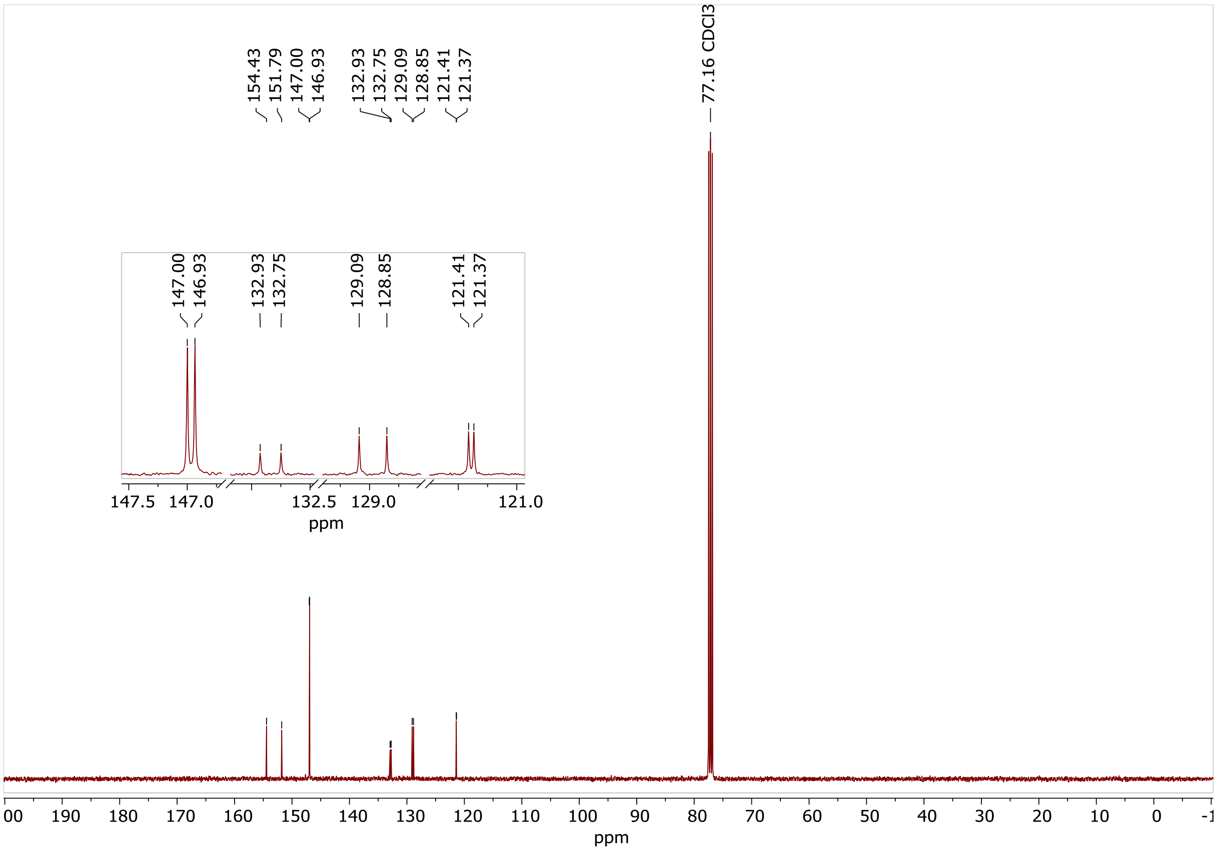

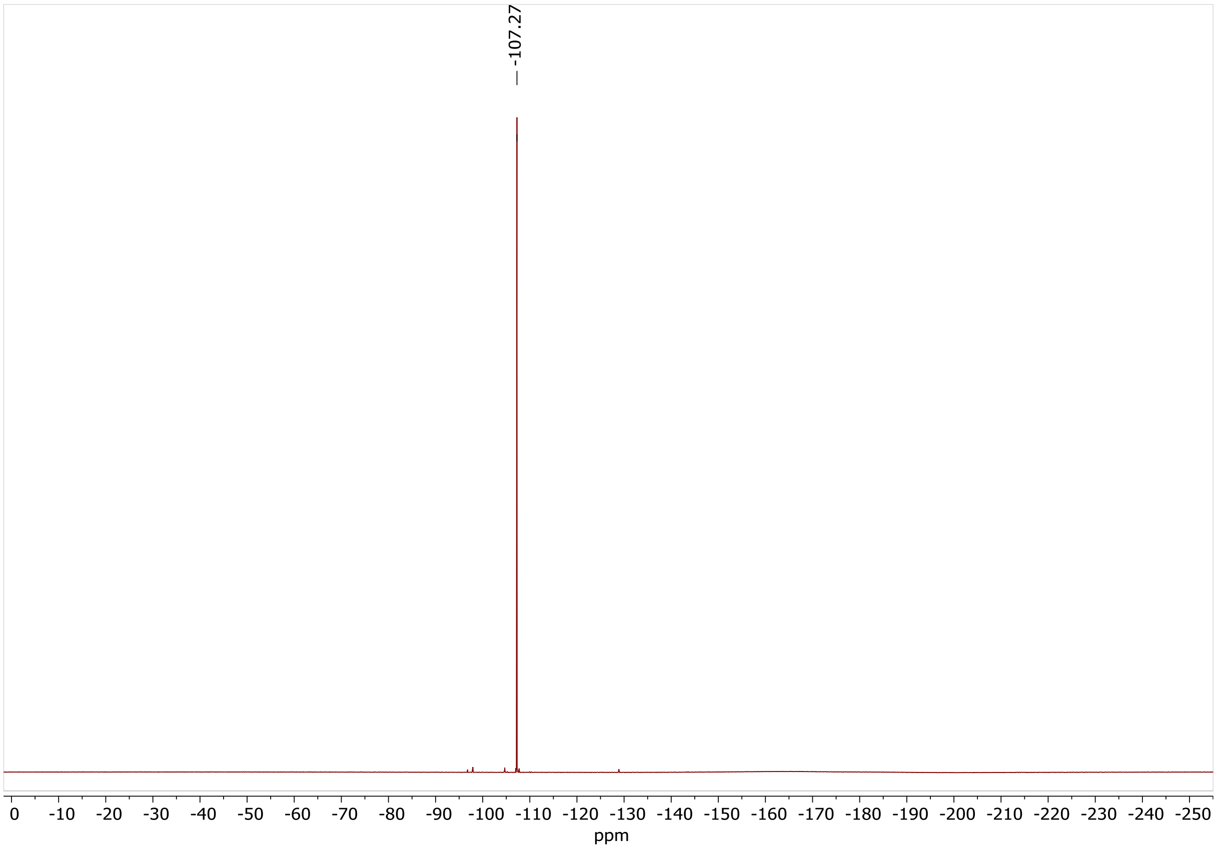

^{1}H and ^{13}C NMR spectra of *S*-butyl benzenesulfonothioate

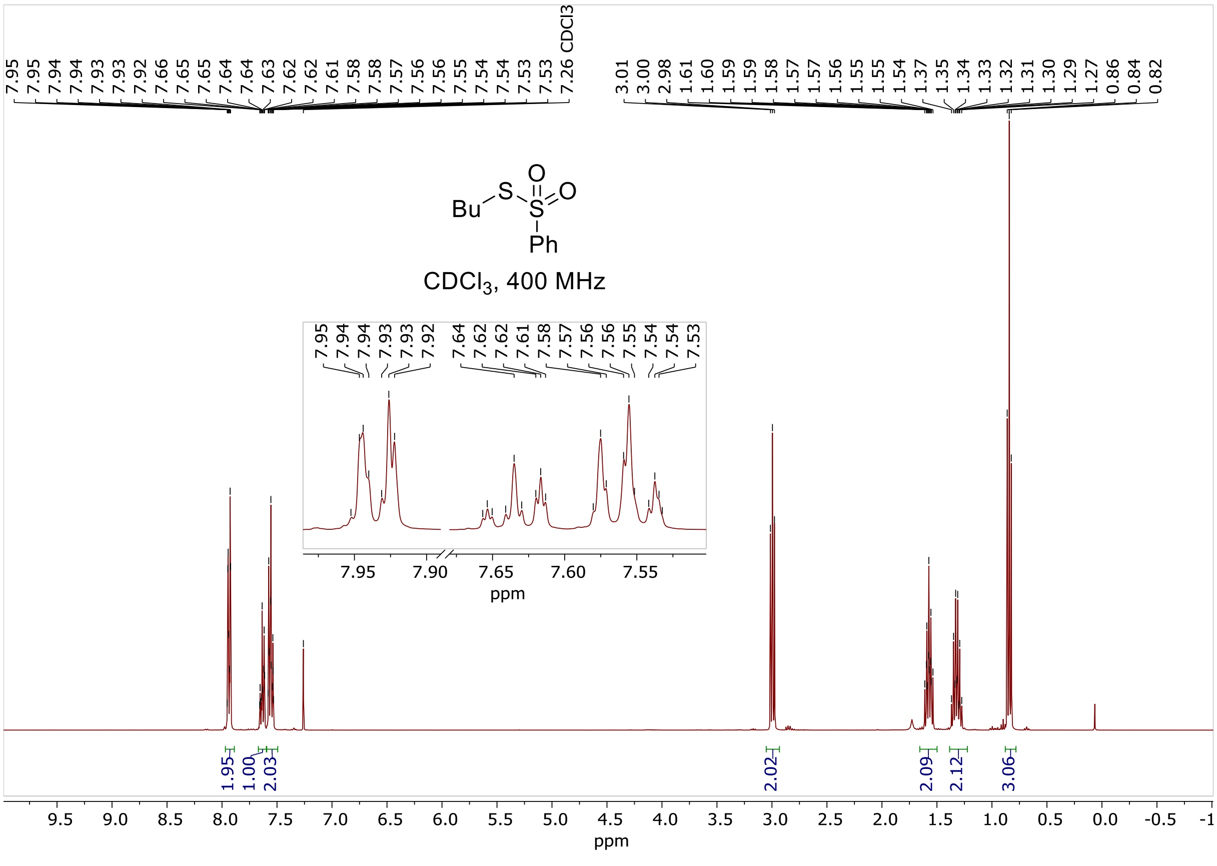

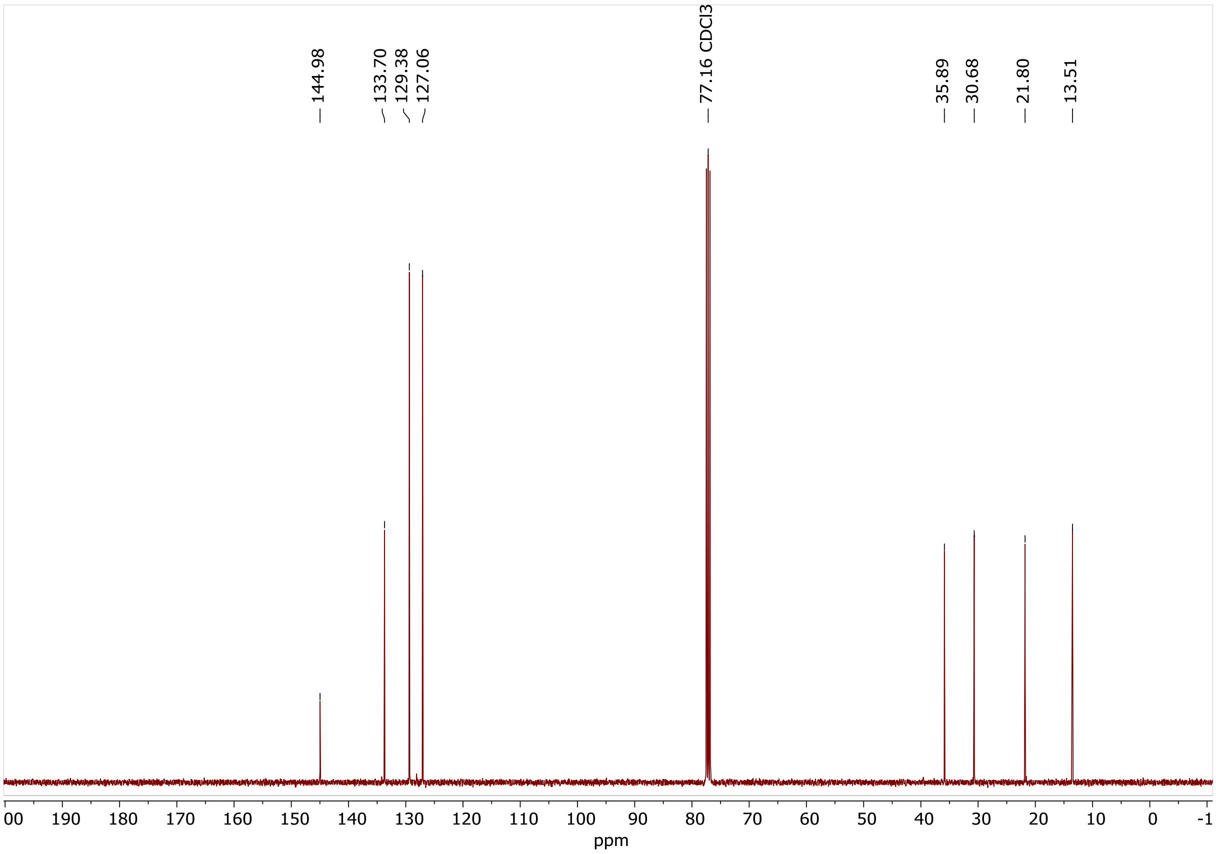

^{1}H, ^{13}C and HMBC NMR spectra of compound **5a**

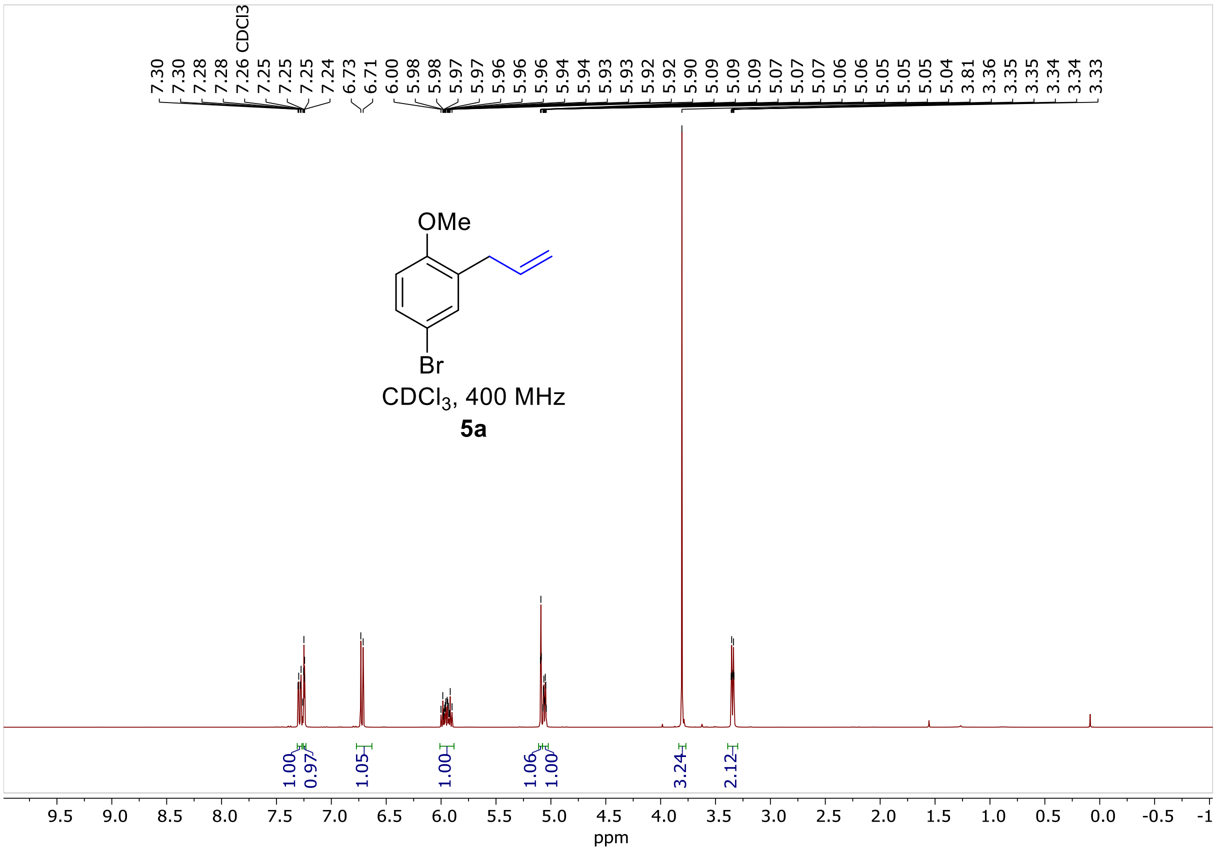

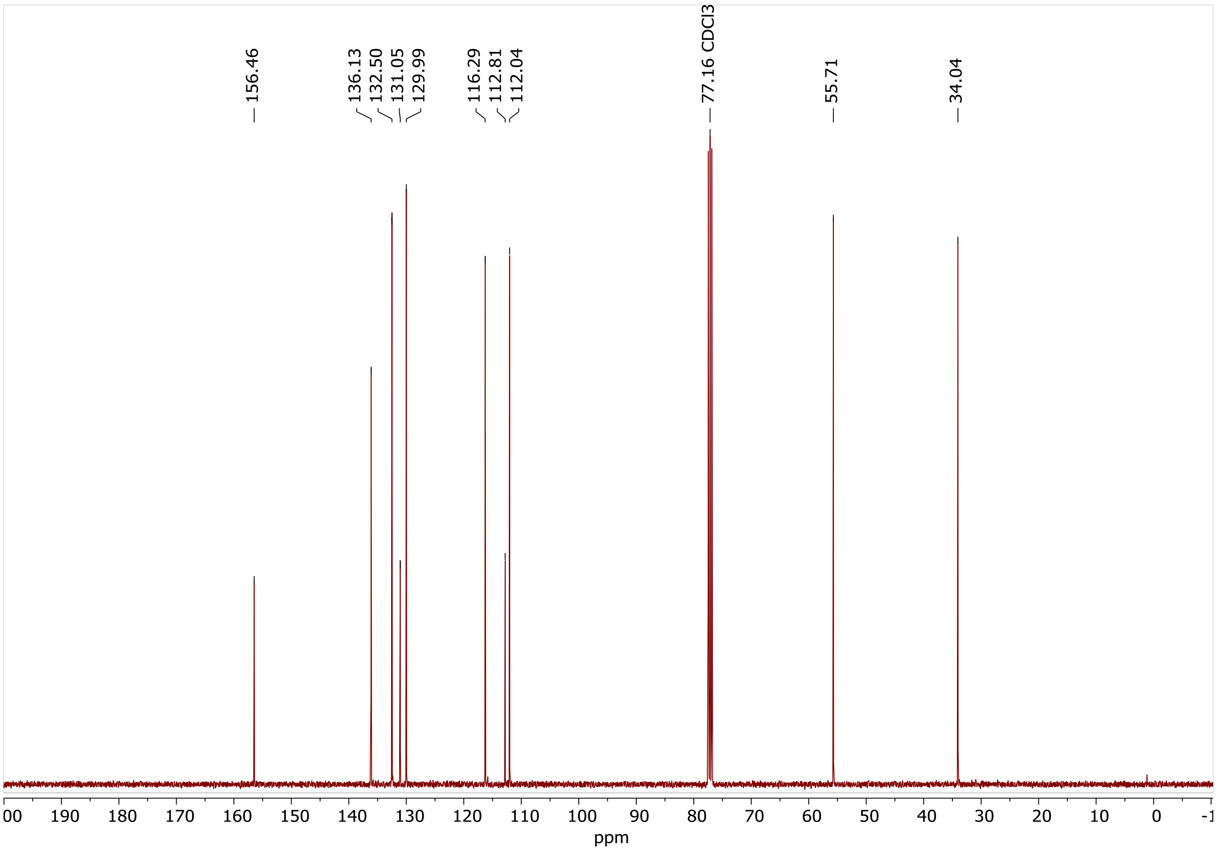
156.46
136.13
132.50
131.05
129.99
116.29
112.81
112.04
77.16 CDCl3
55.71
34.04
ppm

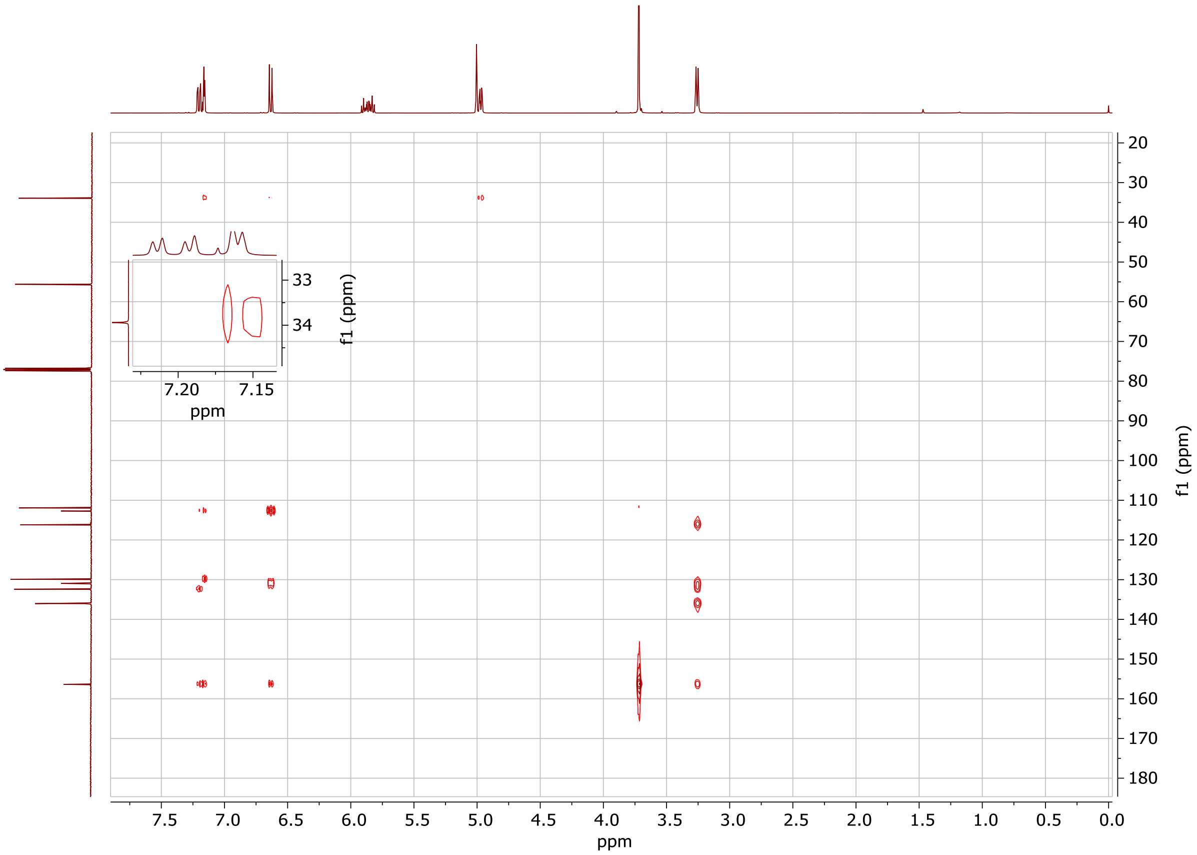
7.20
7.15
ppm
33
34
f1 (ppm)
ppm
f1 (ppm)

^{1}H, ^{13}C and NOESY NMR spectra of compound **5b**

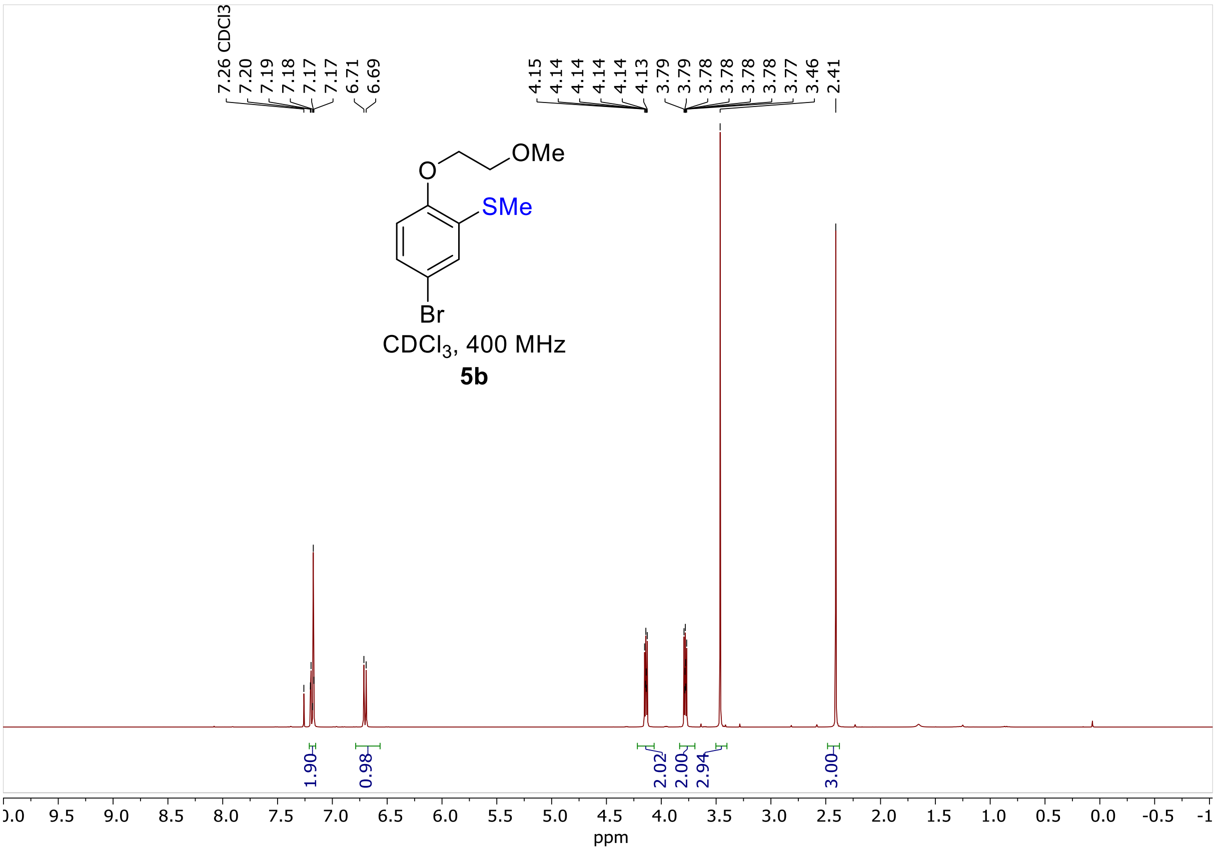

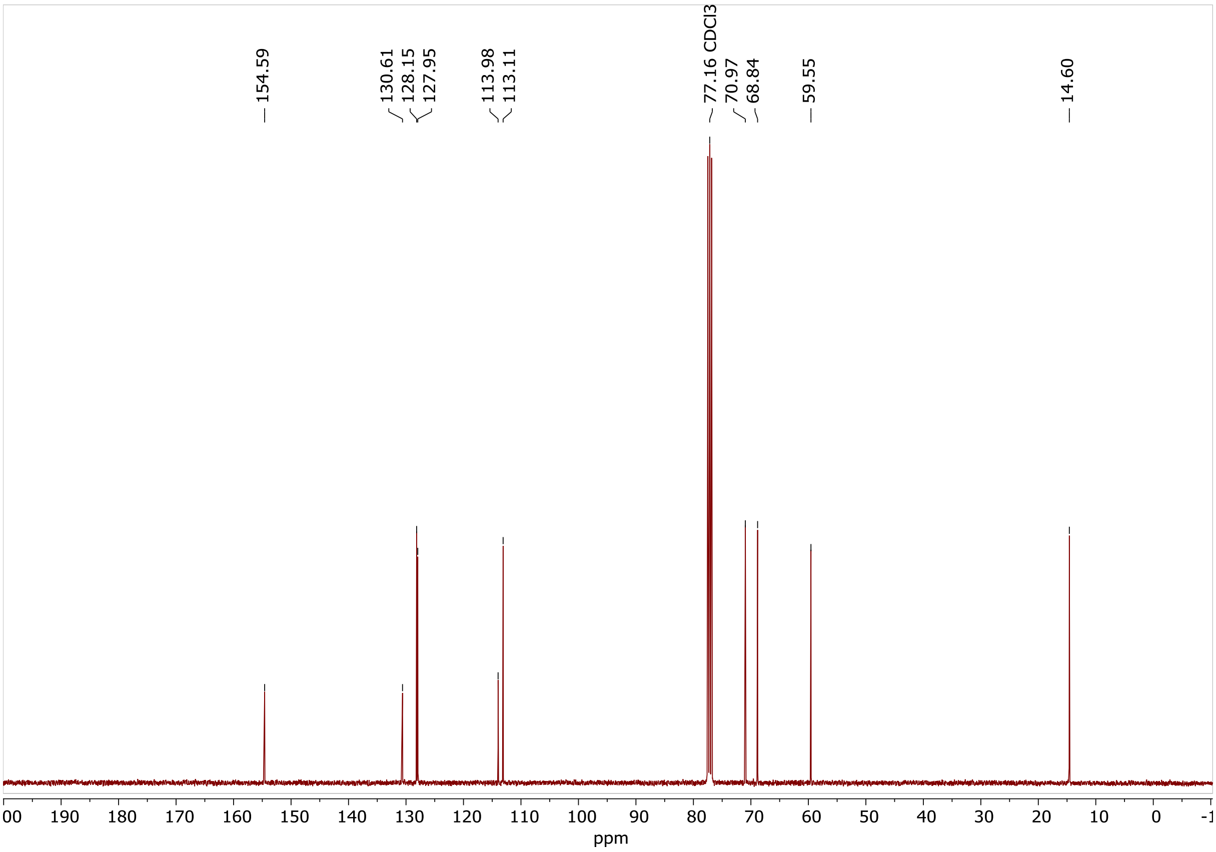

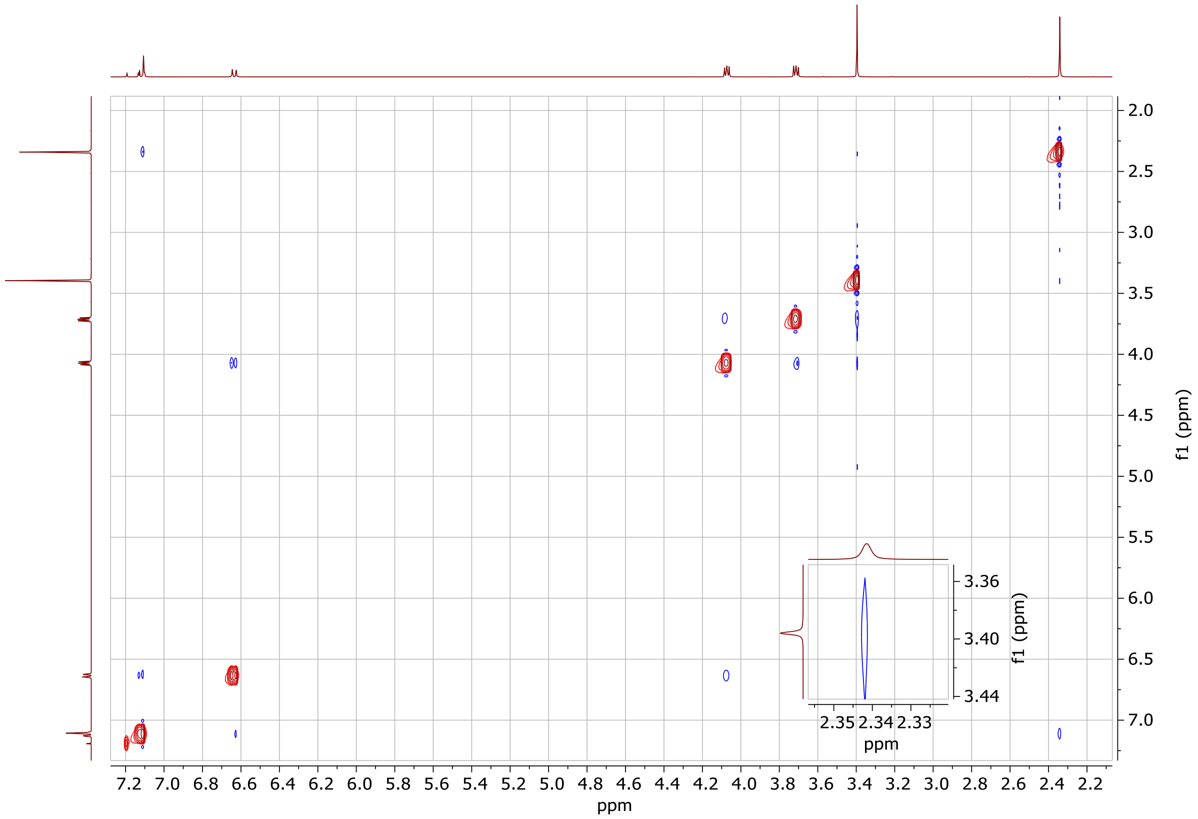

^{1}H, ^{13}C and HMBC NMR spectra of compound **5c**

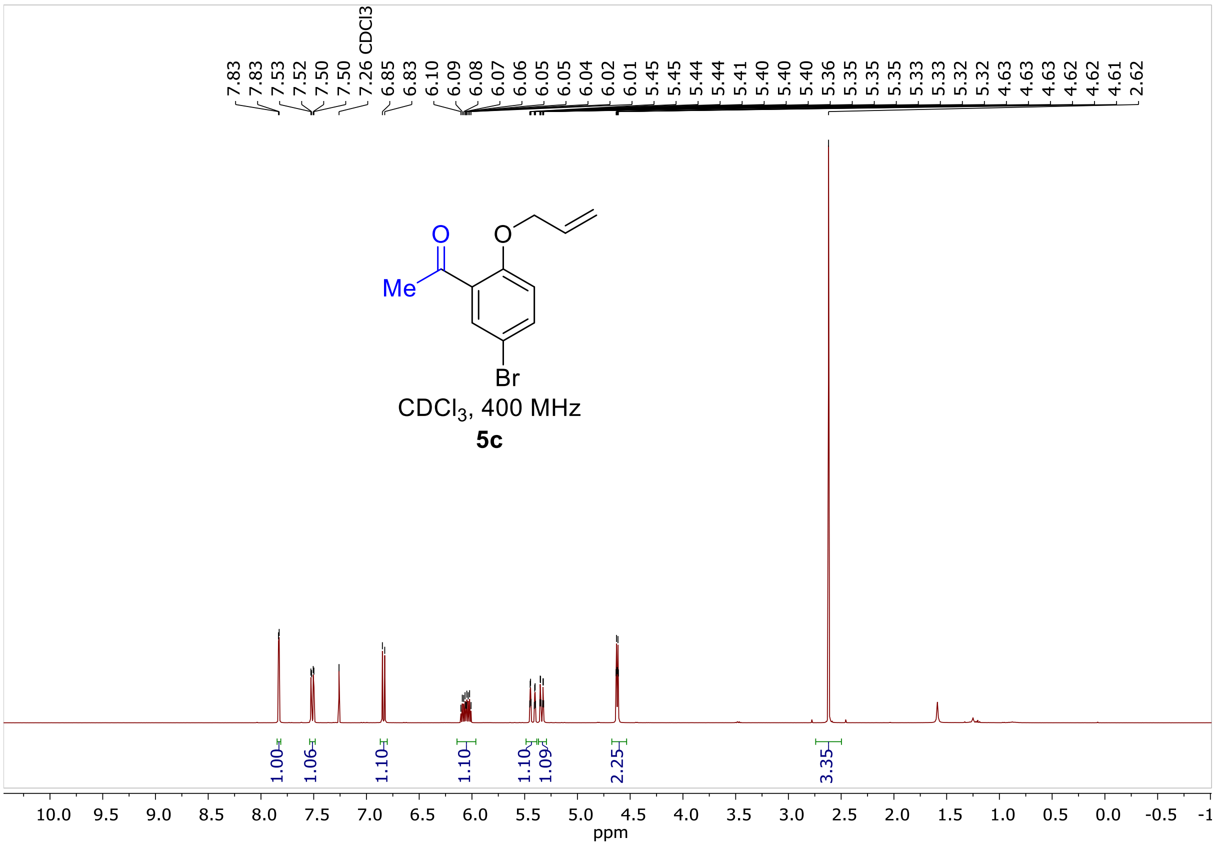

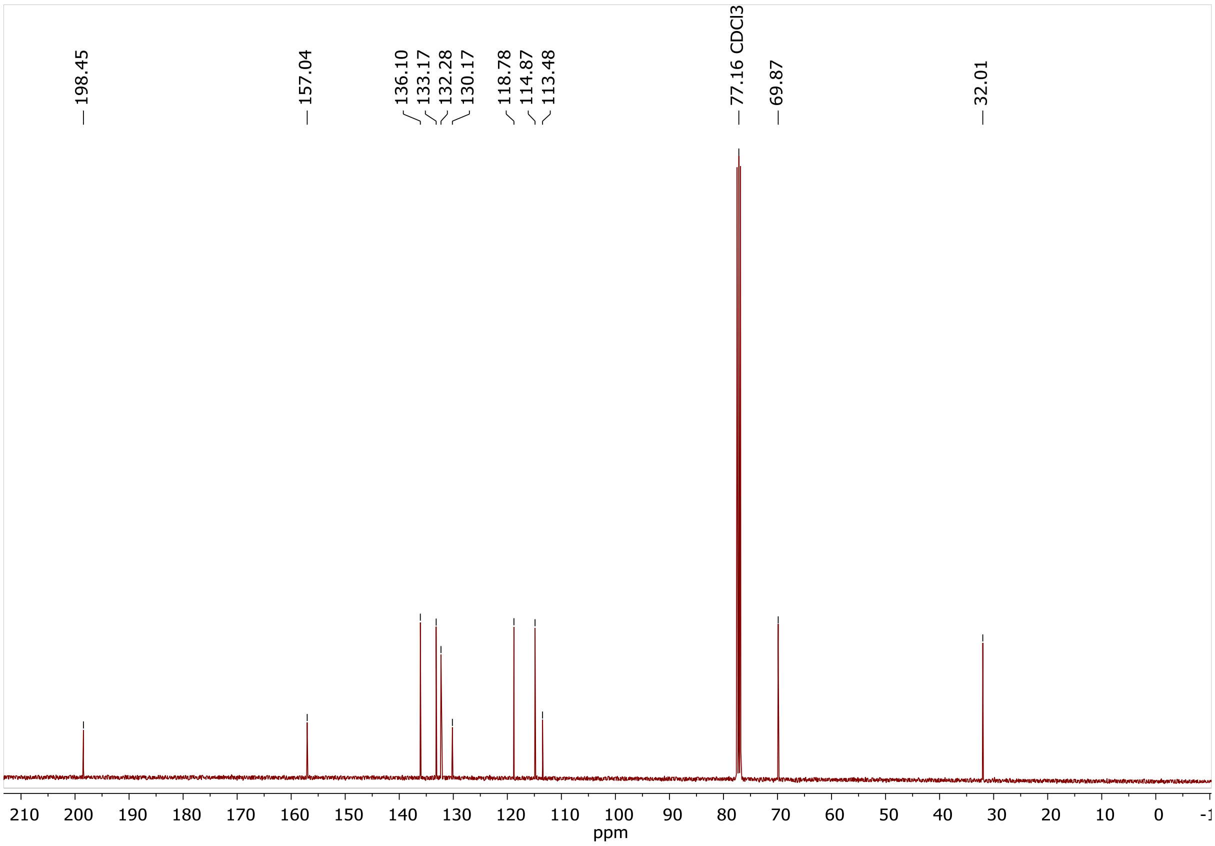
198.45
157.04
136.10
133.17
132.28
130.17
118.78
114.87
113.48
77.16 CDCl3
69.87
32.01
210 200 190 180 170 160 150 140 130 120 110 100 90 80 70 60 50 40 30 20 10 0
ppm

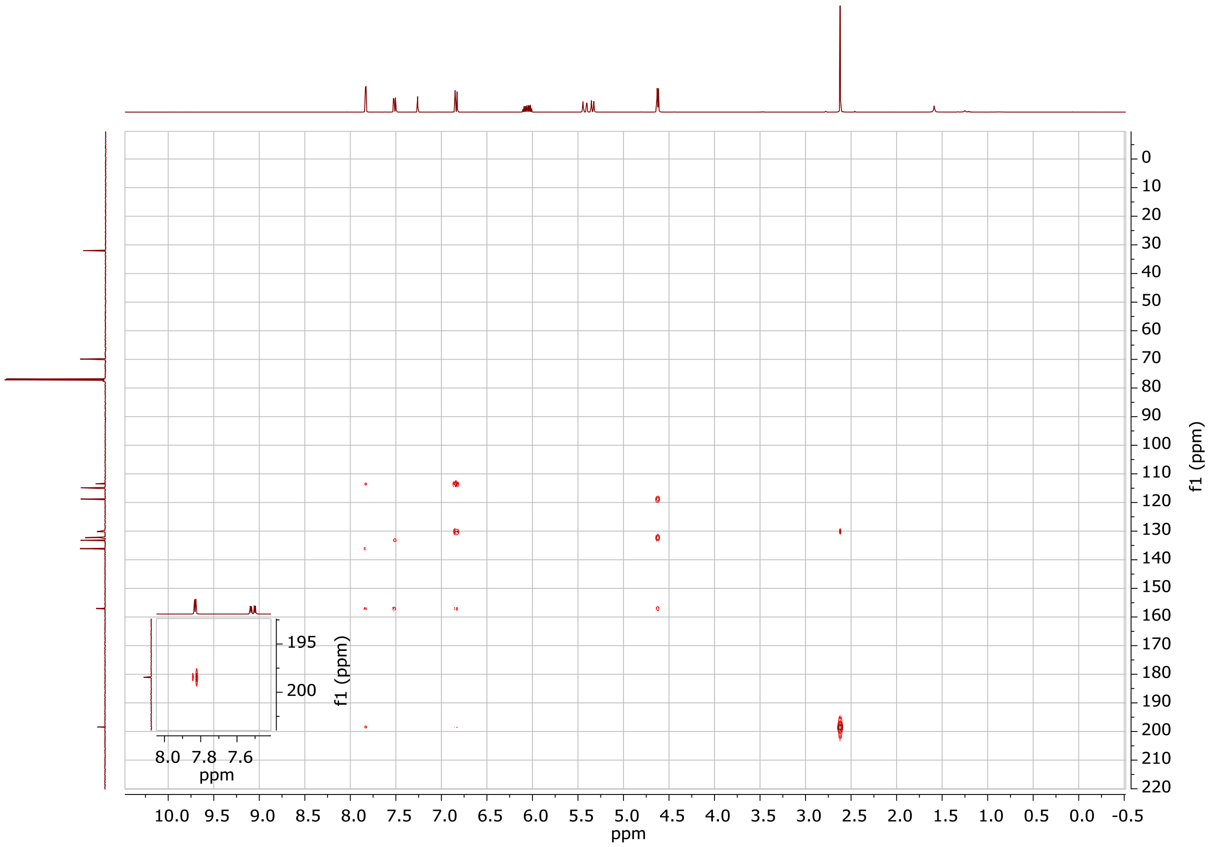
195
200
f1 (ppm)
8.0 7.8 7.6
ppm
0 10 20 30 40 50 60 70 80 90 100 110 120 130 140 150 160 170 180 190 200 210 220
f1 (ppm)
10.0 9.5 9.0 8.5 8.0 7.5 7.0 6.5 6.0 5.5 5.0 4.5 4.0 3.5 3.0 2.5 2.0 1.5 1.0 0.5 0.0 -0.5
ppm

^{1}H and ^{13}C NMR spectra of compound **5d**

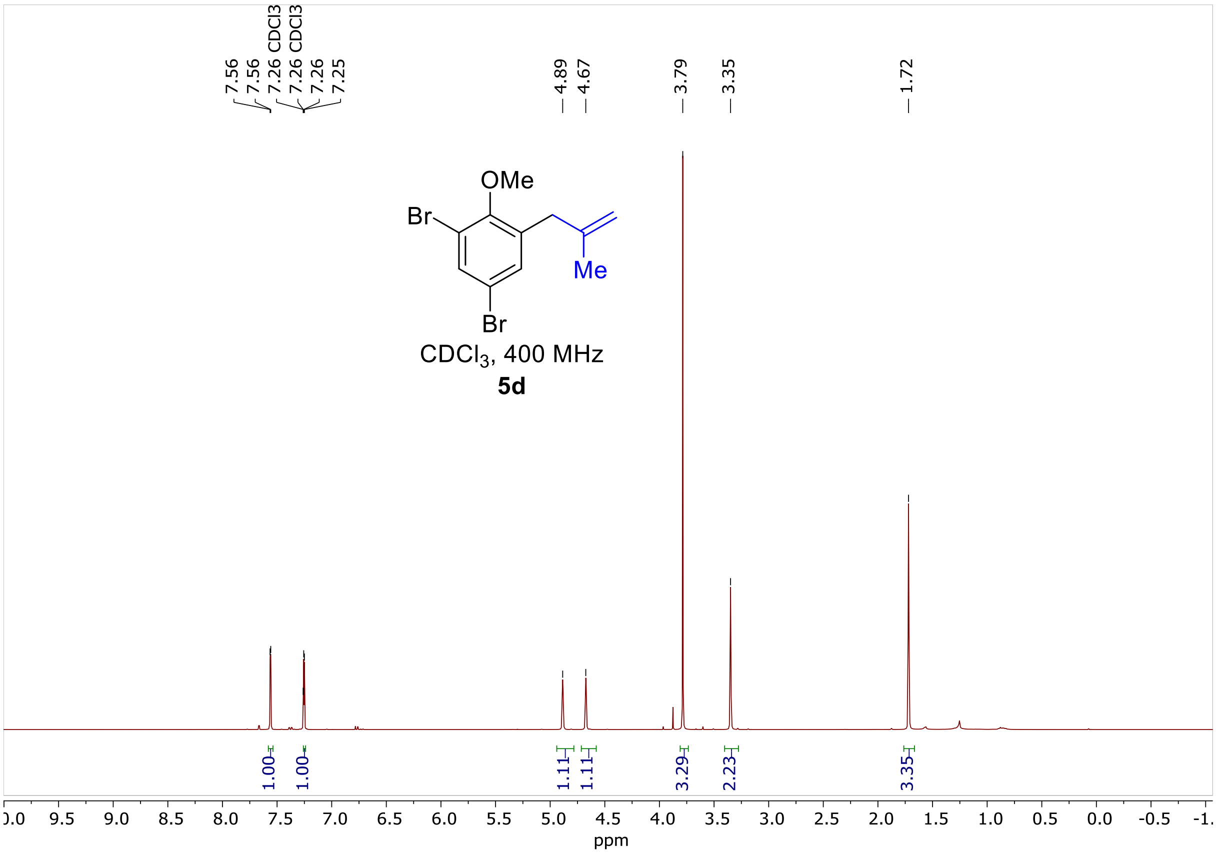

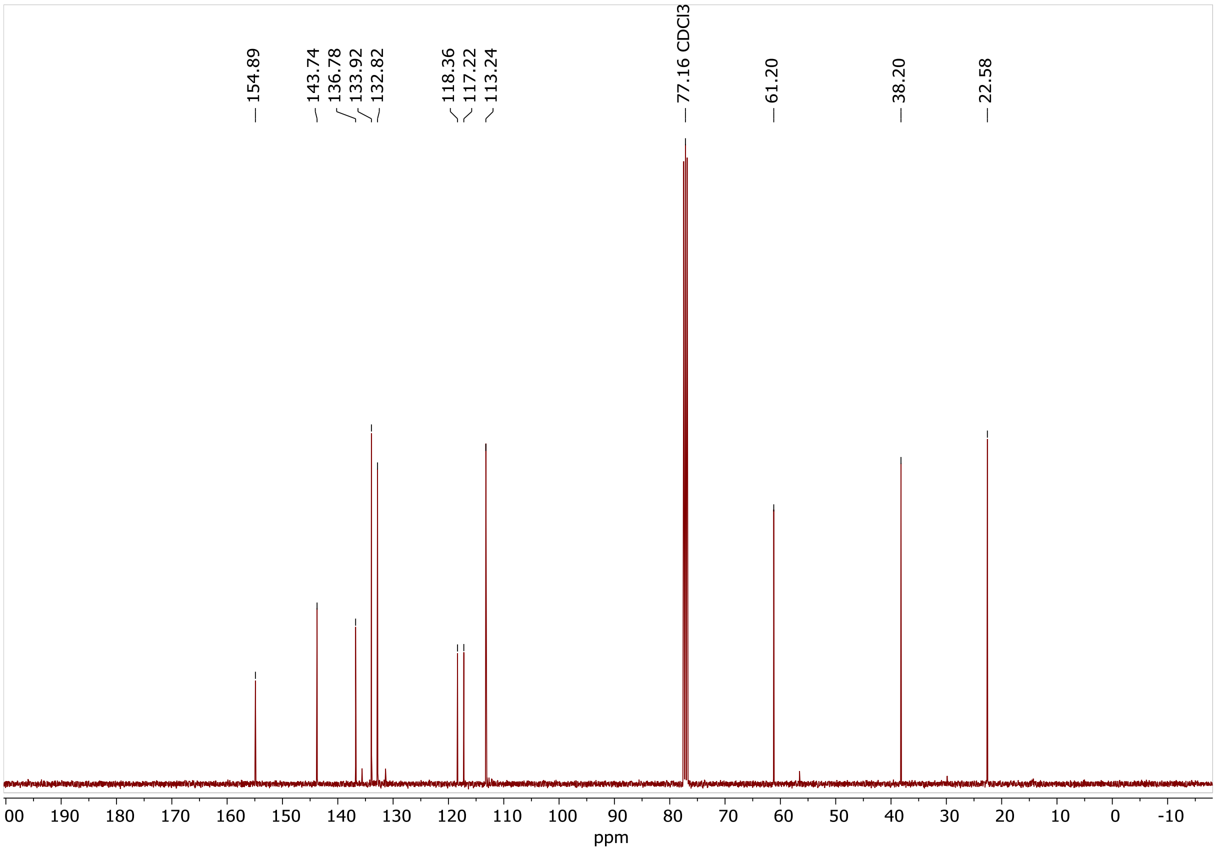

^{1}H, ^{13}C and NOESY NMR spectra of compound **5ea**

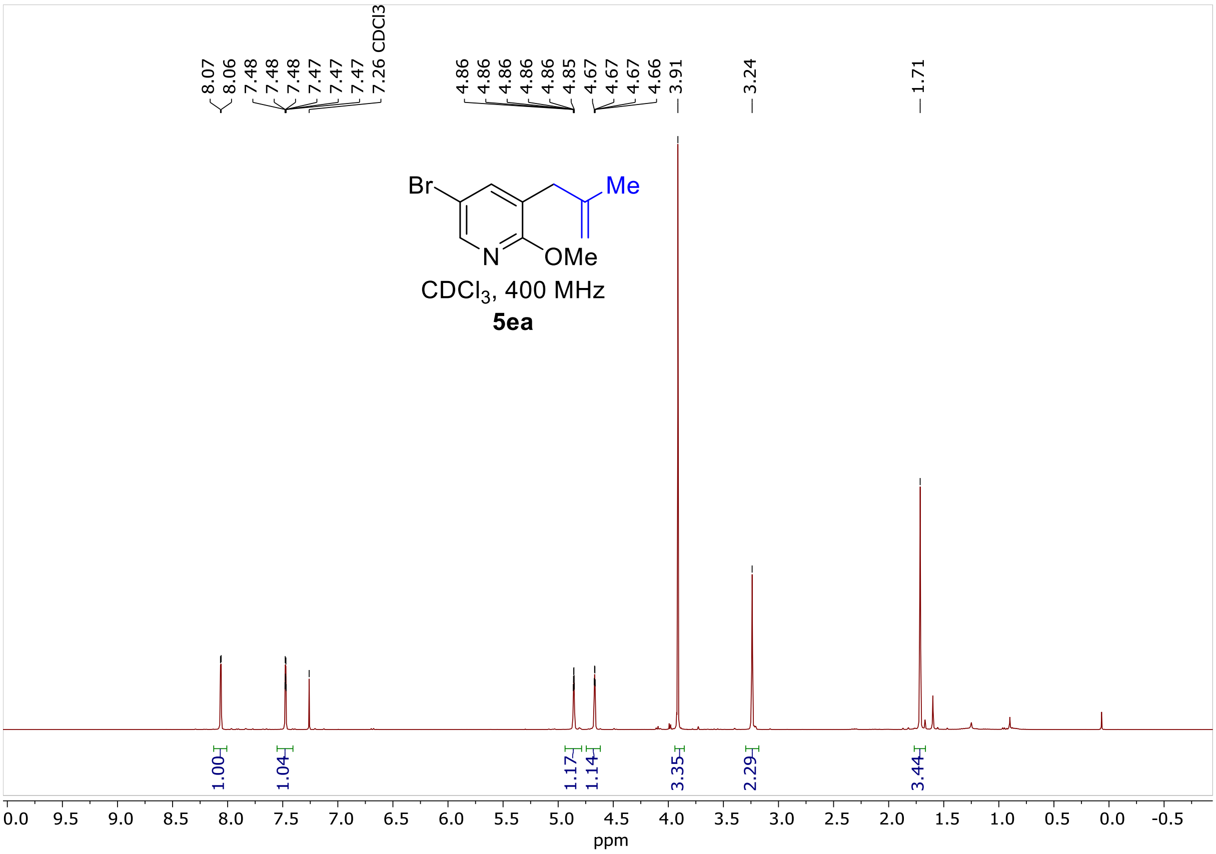

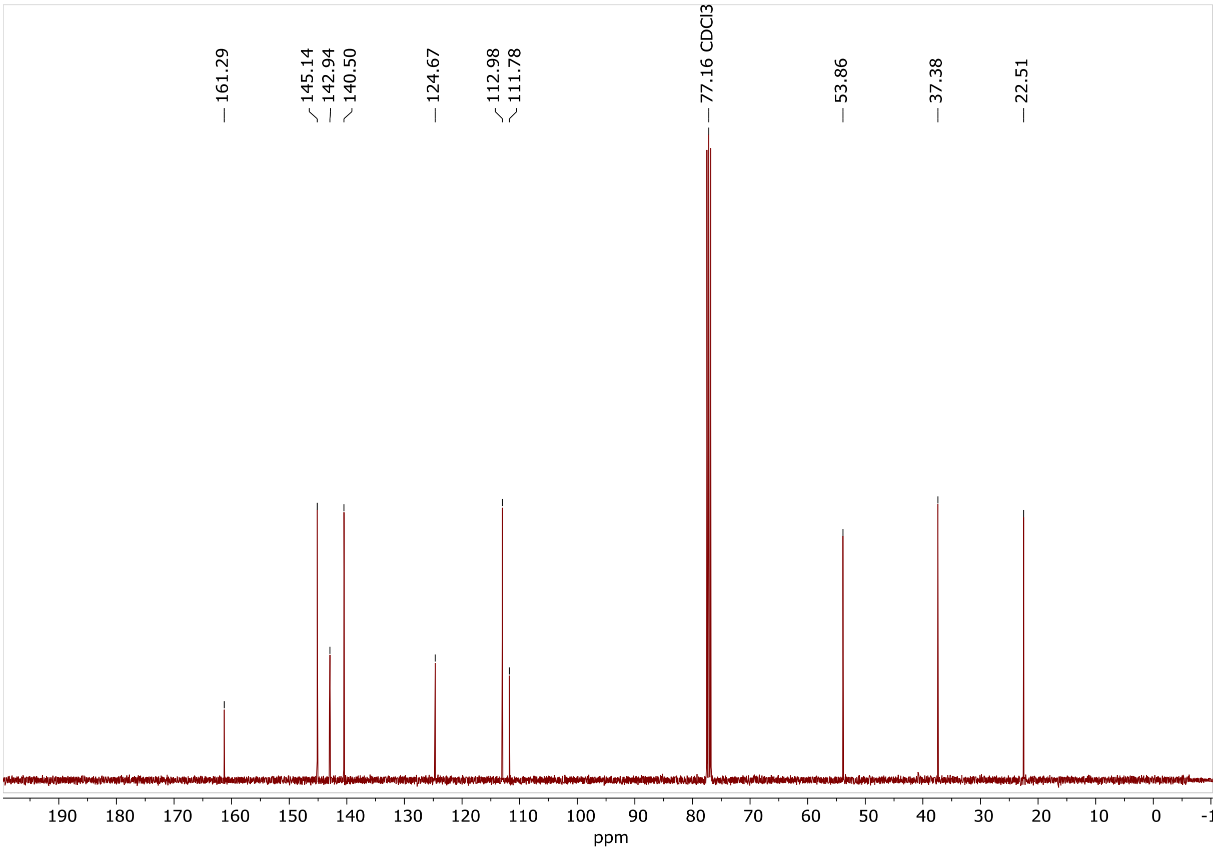

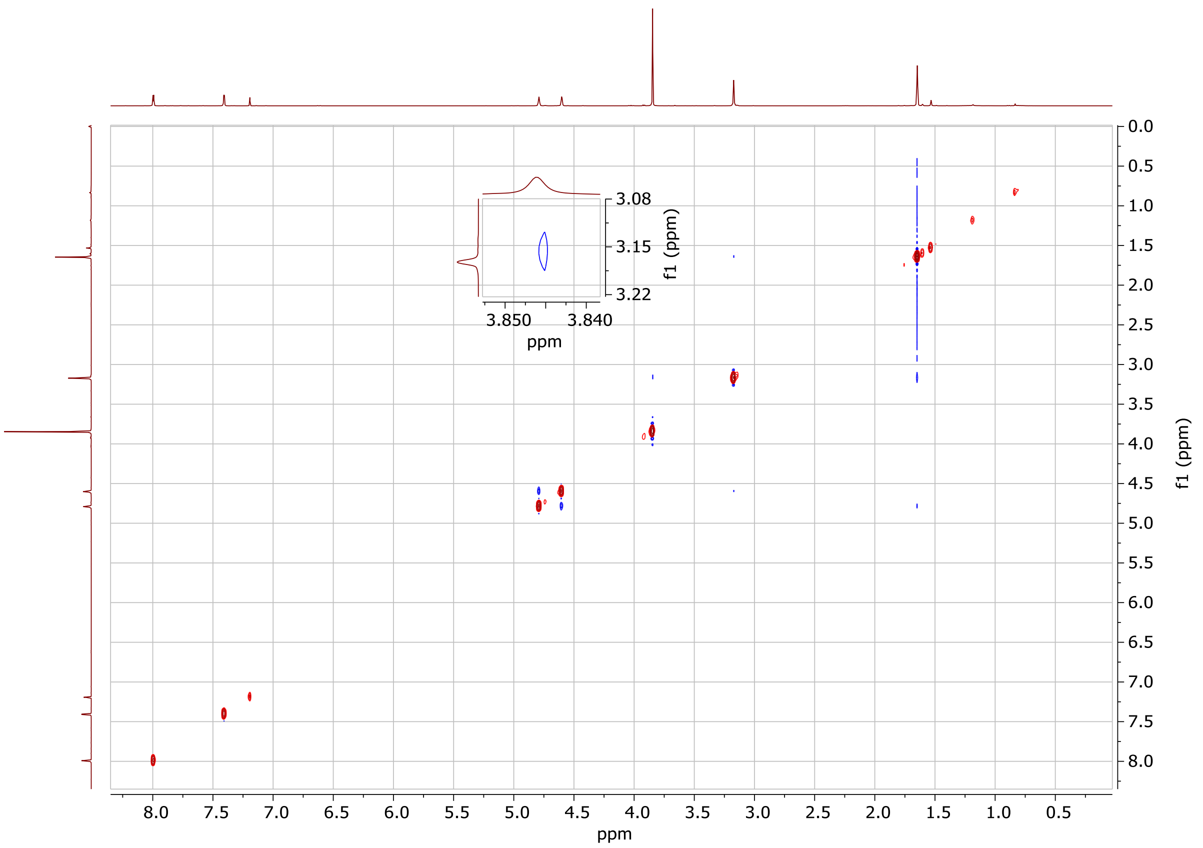

^{1}H, ^{13}C and HMBC NMR spectra of compound **5eb**

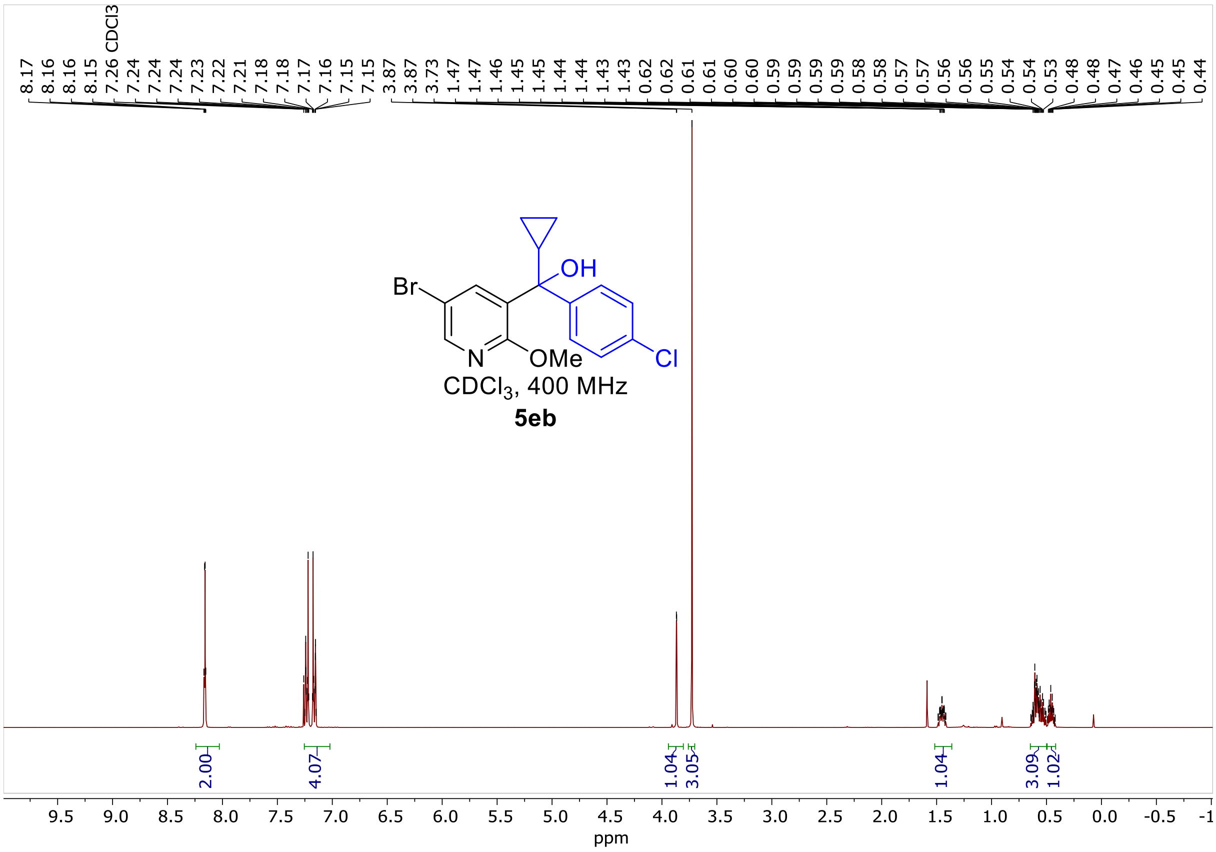

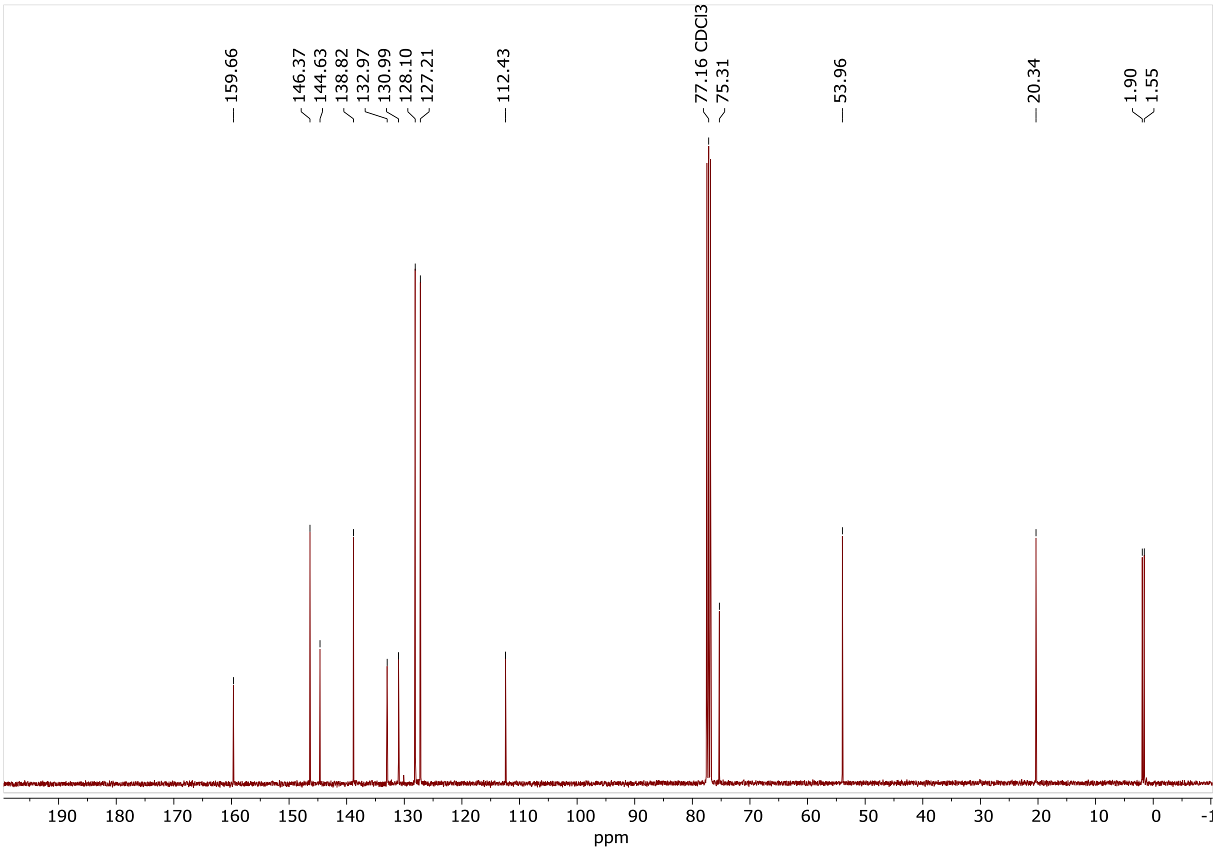
159.66
146.37
144.63
138.82
132.97
130.99
128.10
127.21
112.43
77.16 CDCl3
75.31
53.96
20.34
1.90
1.55
ppm

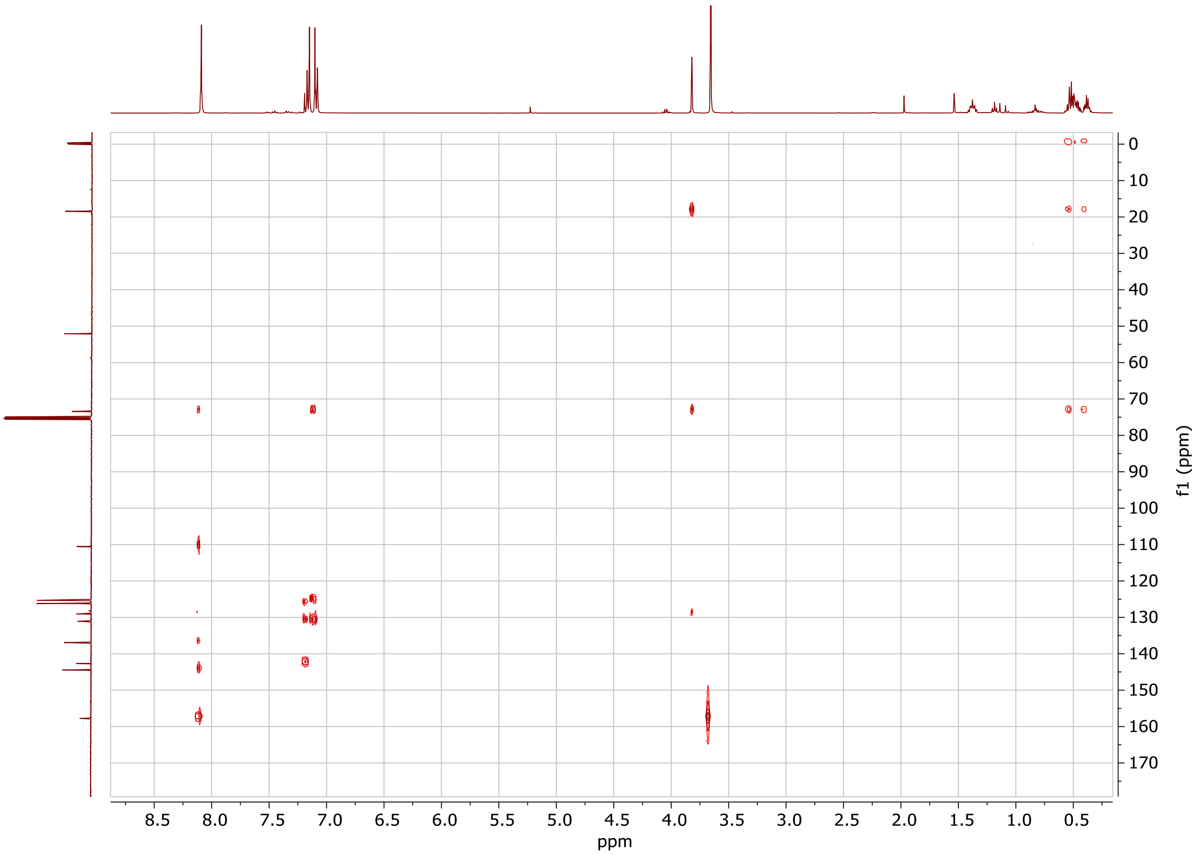
ppm
f1 (ppm)

^{1}H, ^{13}C and HMBC NMR spectra of compound **5ec**

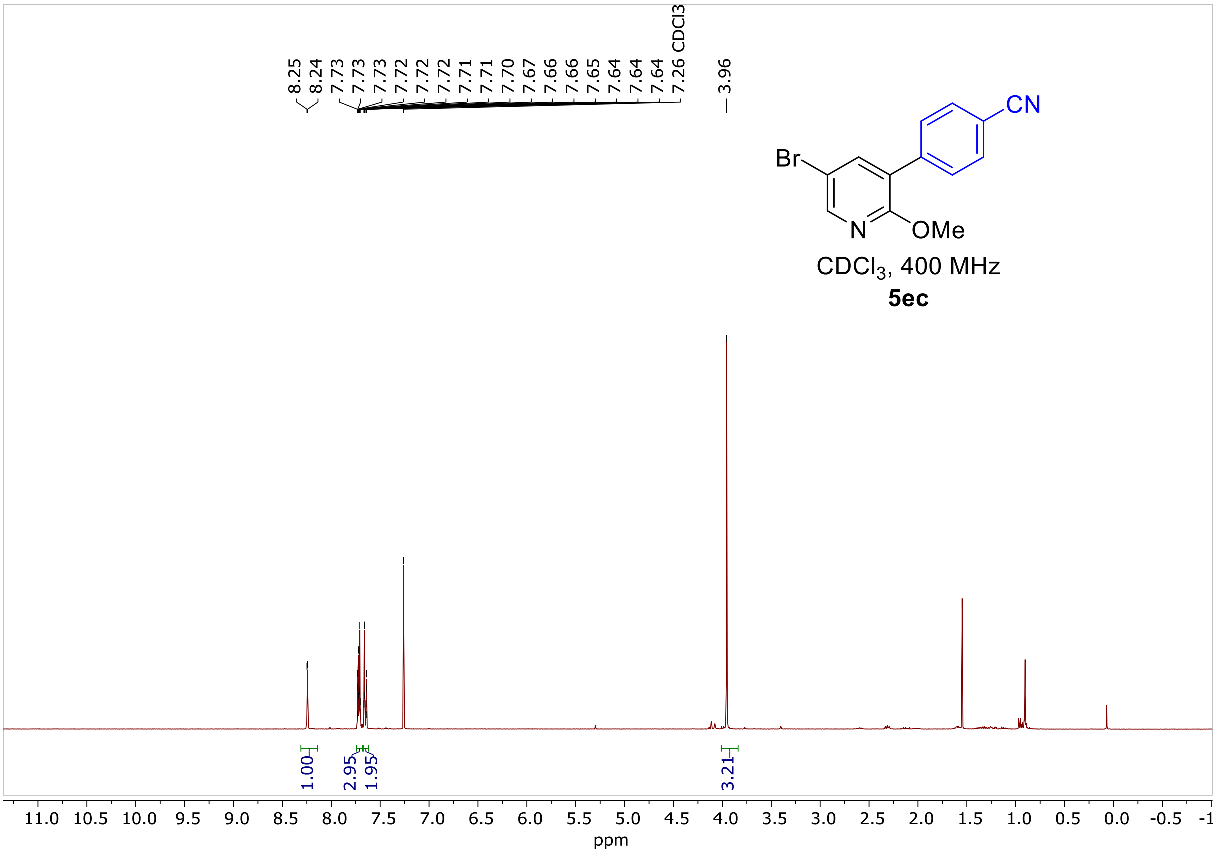

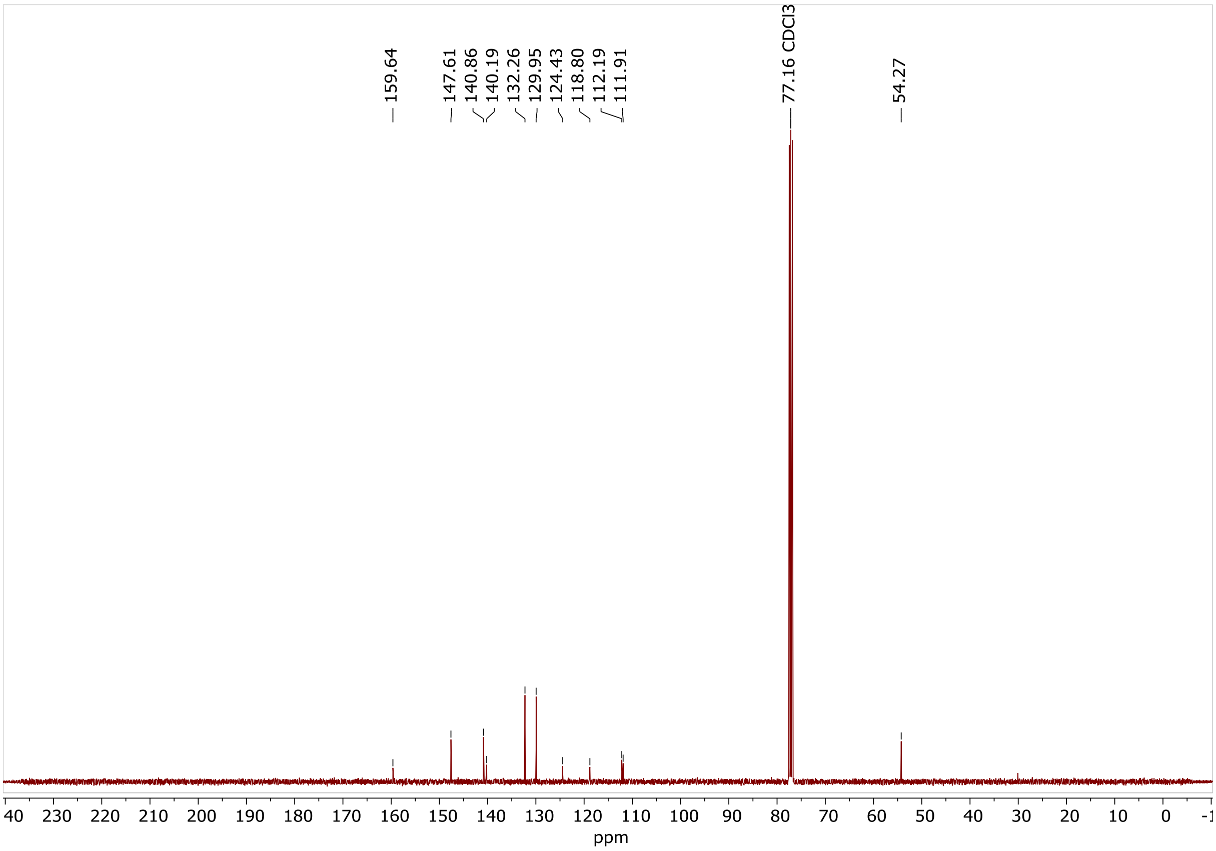

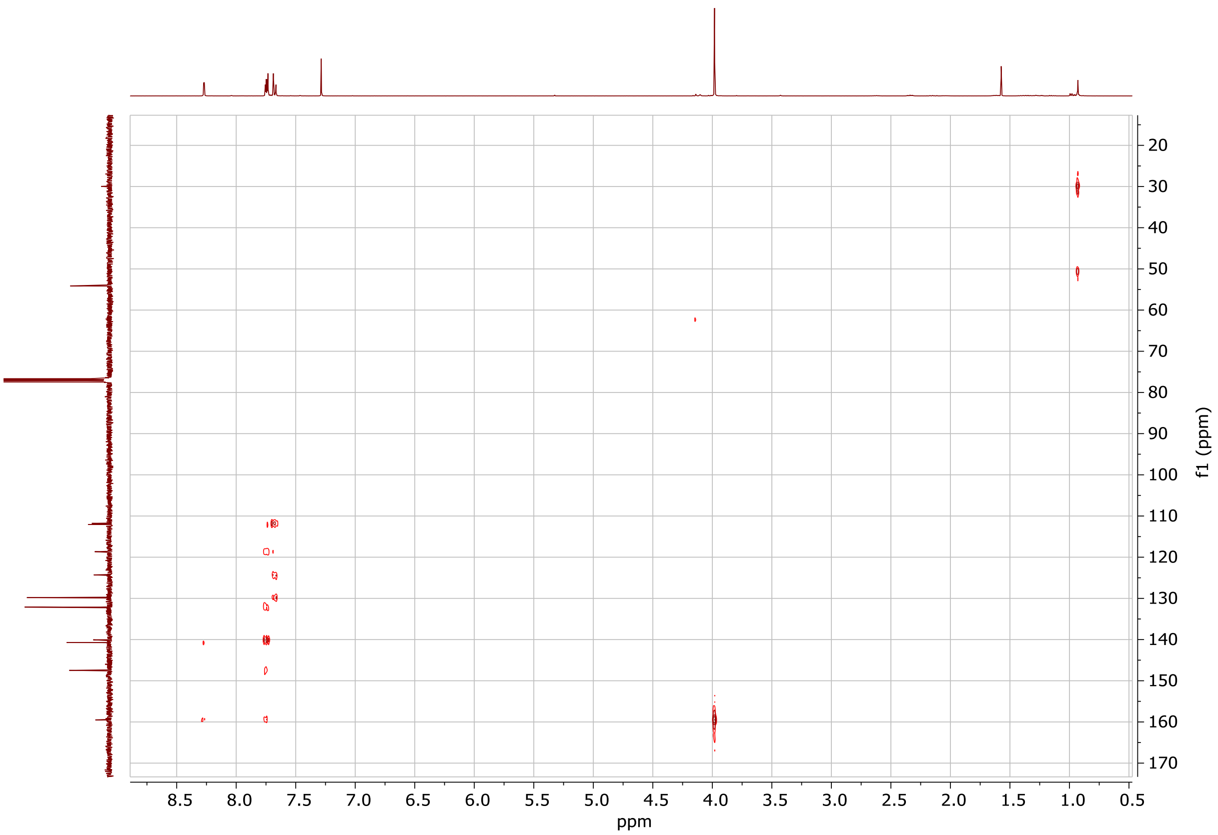

^1H, ^{13}C and HMBC NMR spectra of compound **5f**

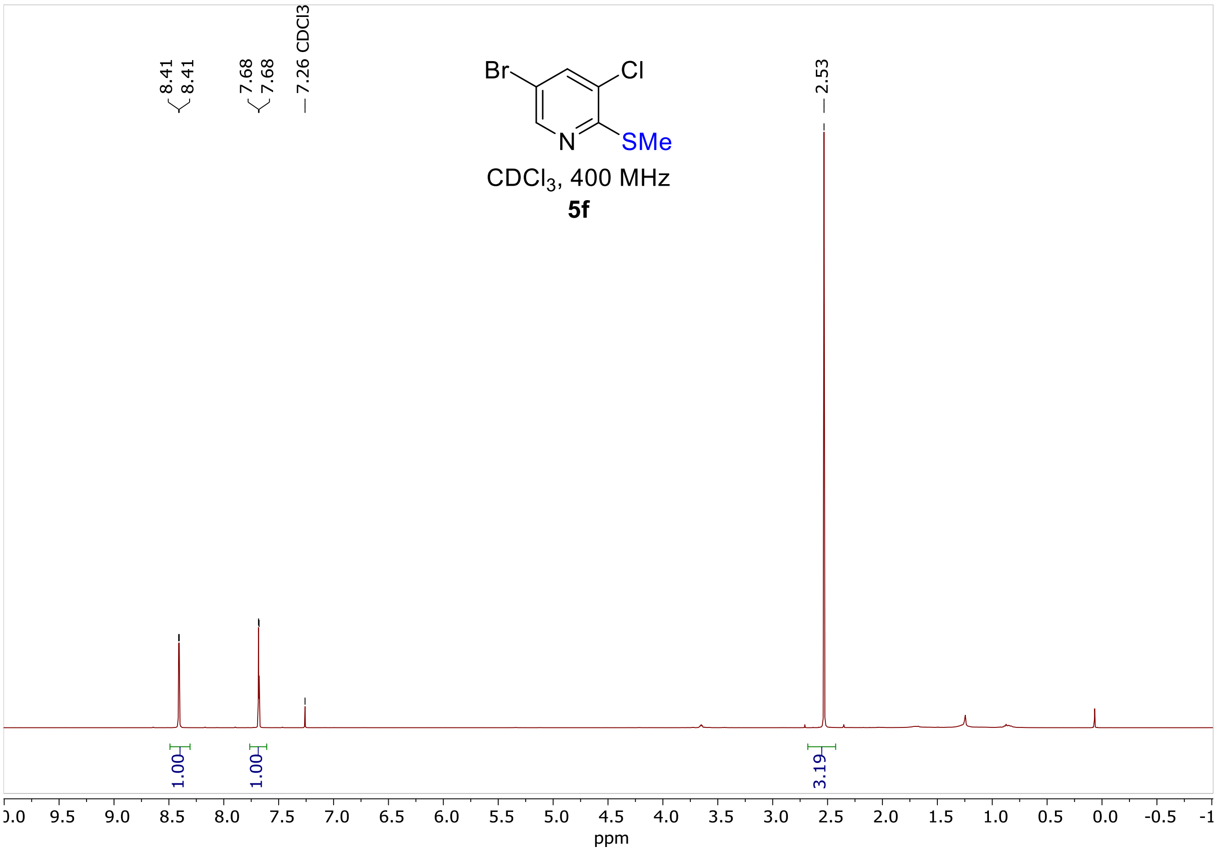

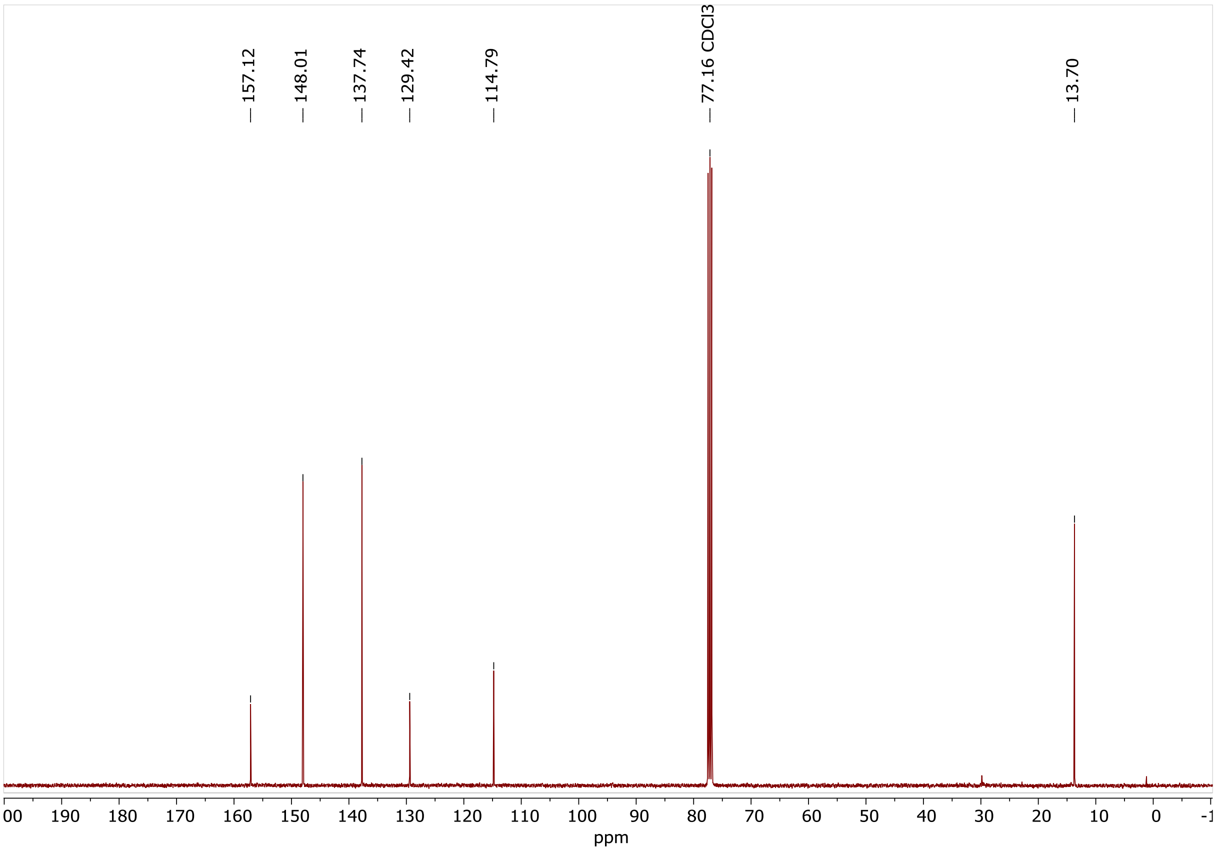
157.12
148.01
137.74
129.42
114.79
77.16 CDCl3
13.70
ppm

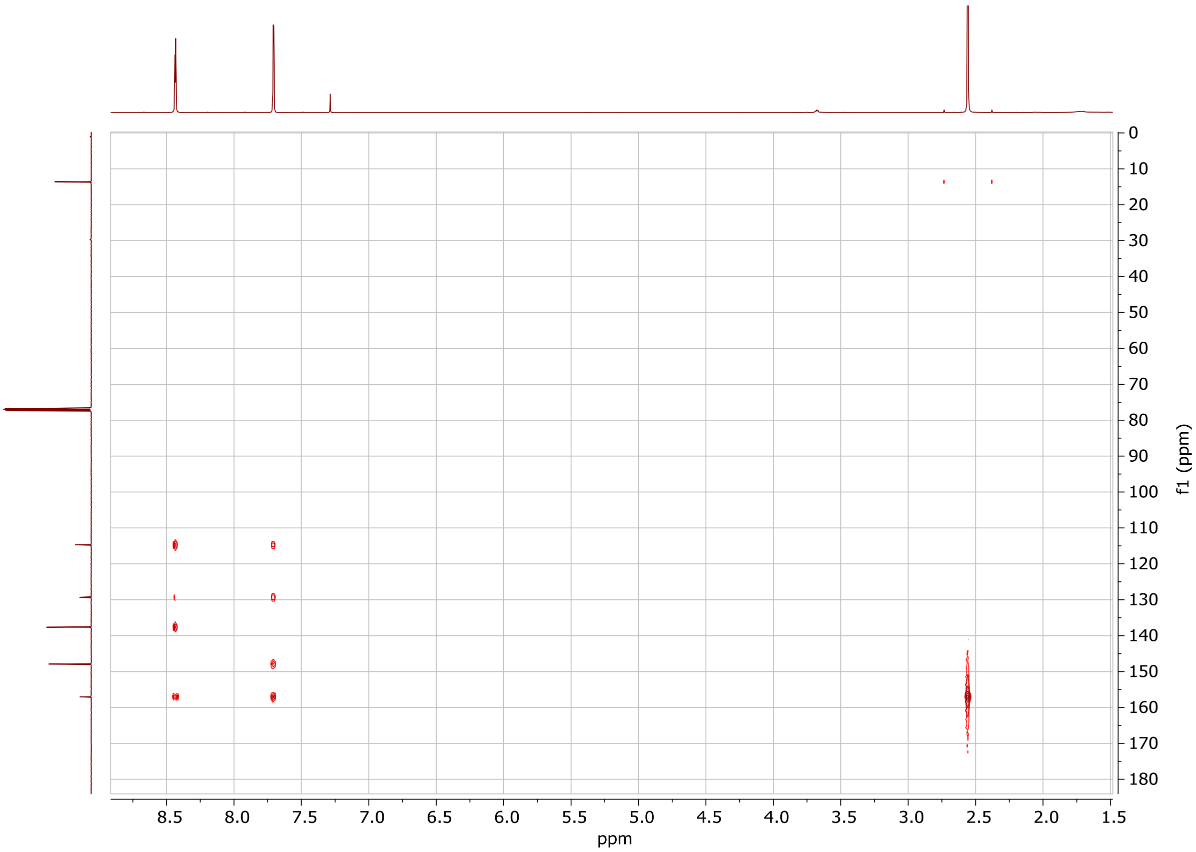
ppm
f1 (ppm)

^{1}H, ^{13}C, ^{19}F and HMBC NMR spectra of compound **5g**

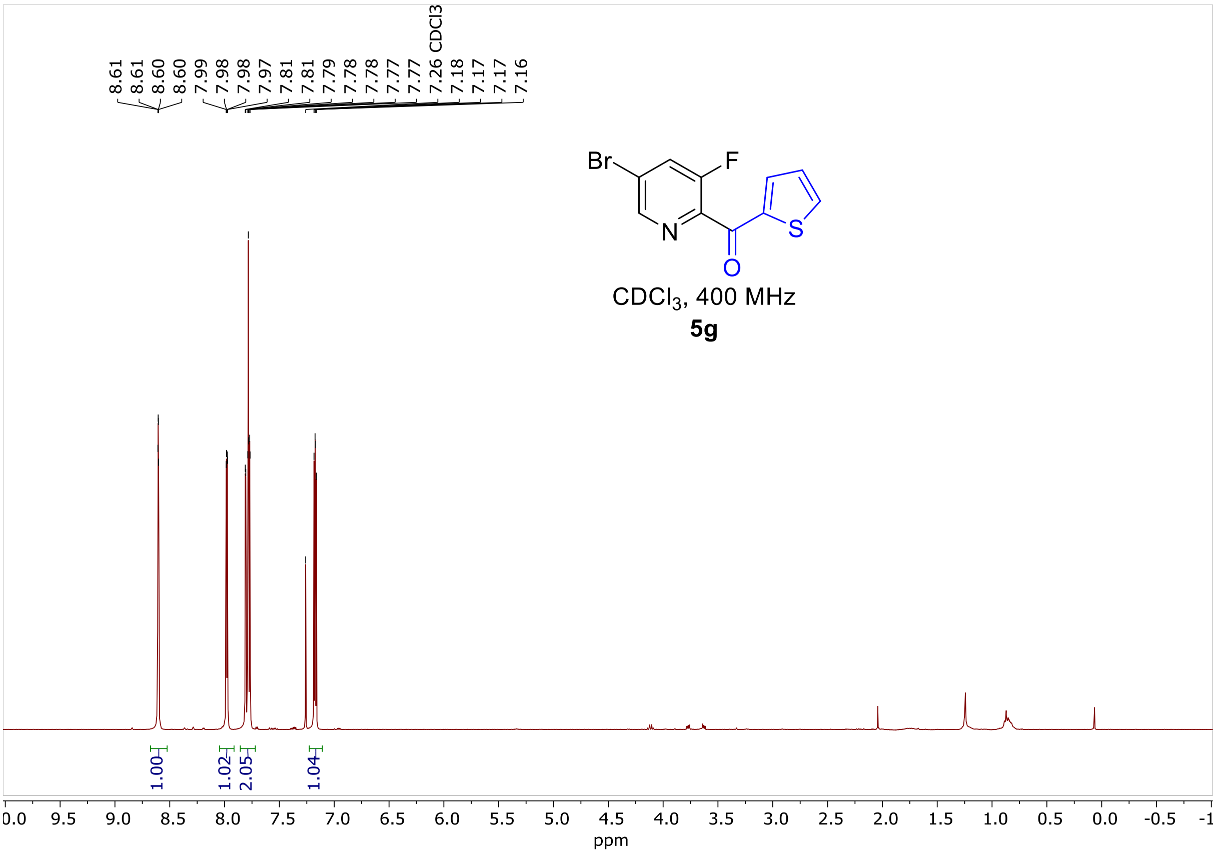

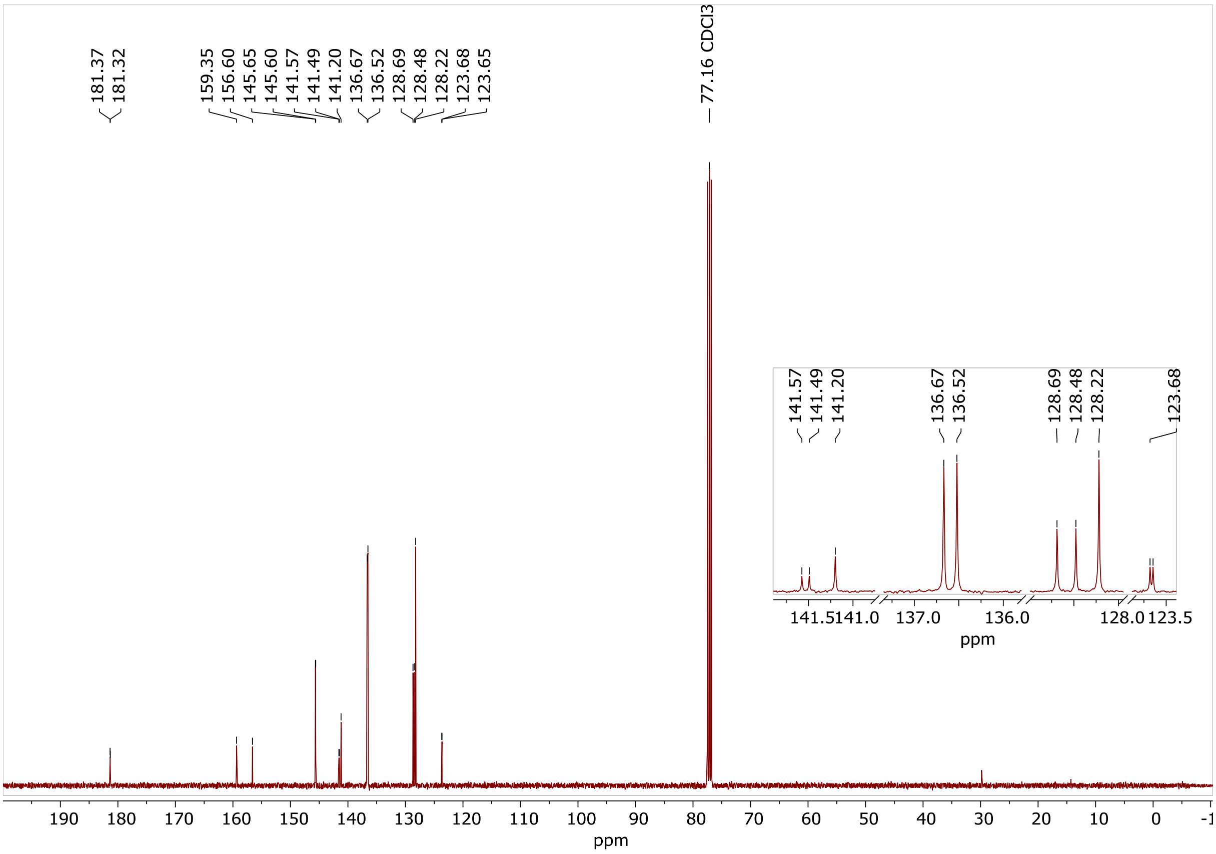

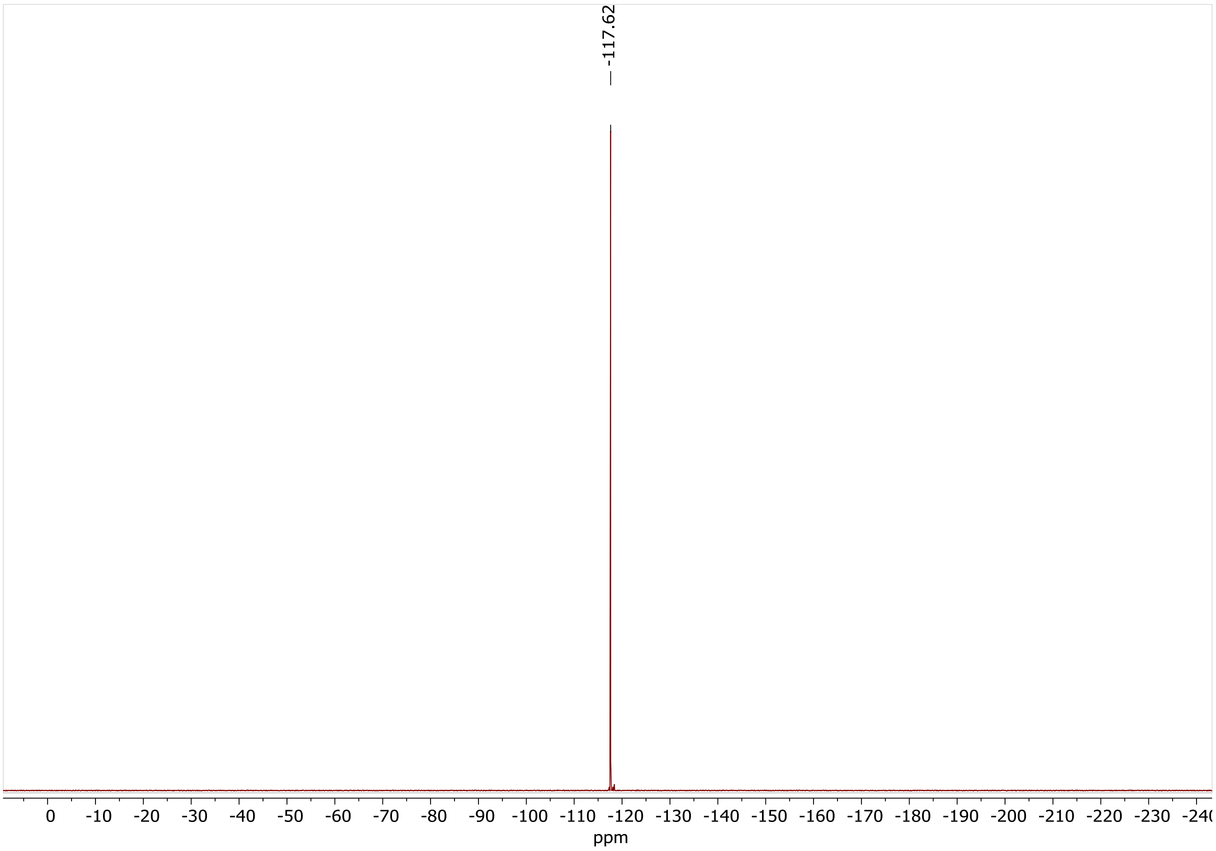
-117.62
0
-10
-20
-30
-40
-50
-60
-70
-80
-90
-100
-110
-120
-130
-140
-150
-160
-170
-180
-190
-200
-210
-220
-230
ppm

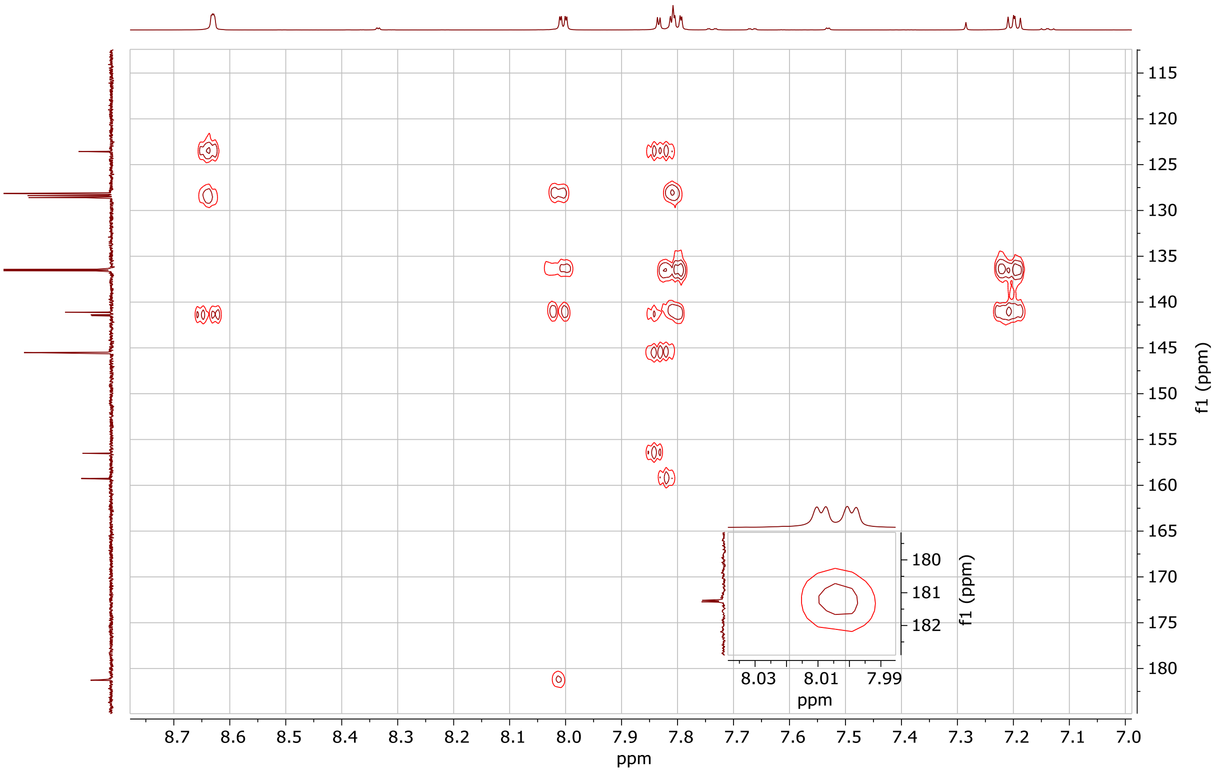
8.7
8.6
8.5
8.4
8.3
8.2
8.1
8.0
7.9
7.8
7.7
7.6
7.5
7.4
7.3
7.2
7.1
7.0
ppm
115
120
125
130
135
140
145
150
155
160
165
170
175
180
f1 (ppm)
8.03
8.01
7.99
ppm
180
181
182
f1 (ppm)

^{1}H, ^{13}C, ^{19}F and HMBC NMR spectra of compound **5aa**

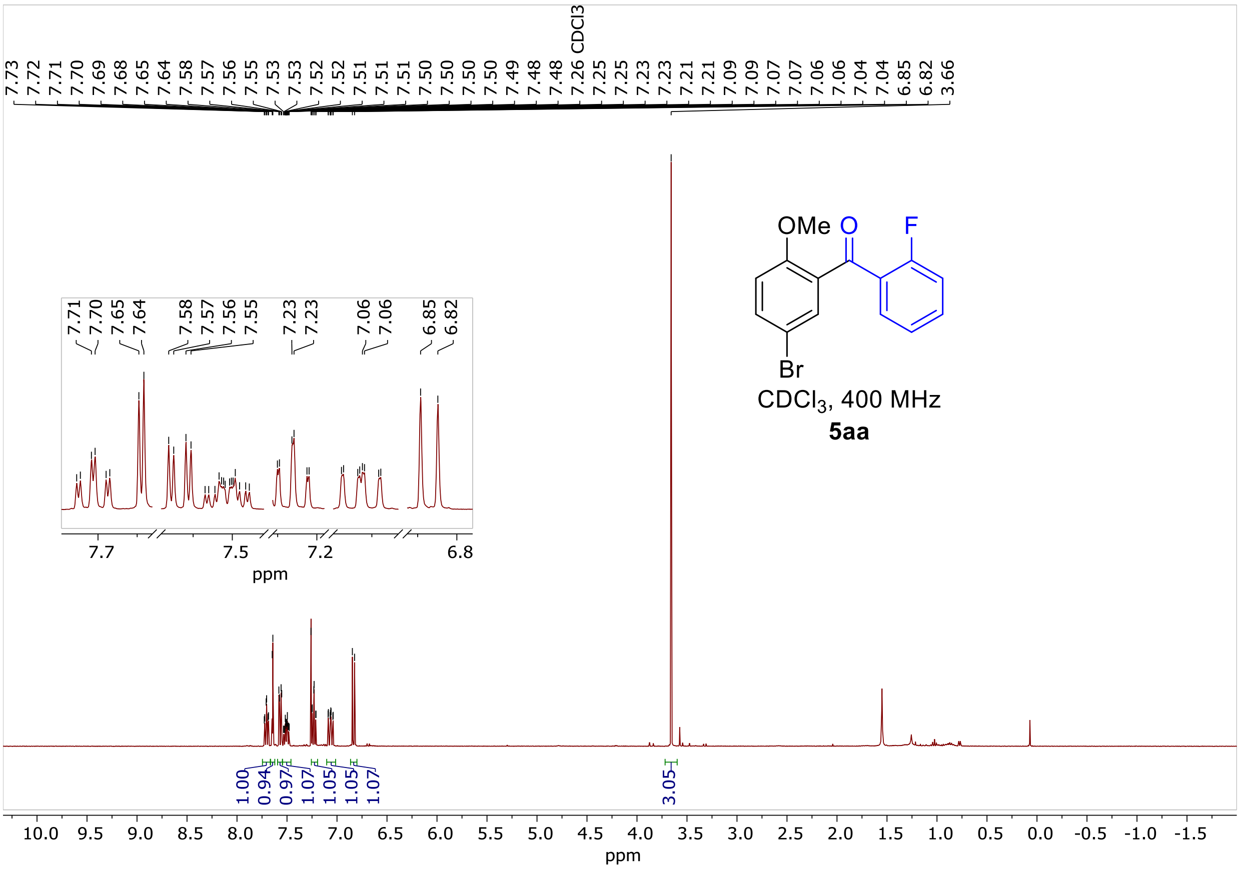

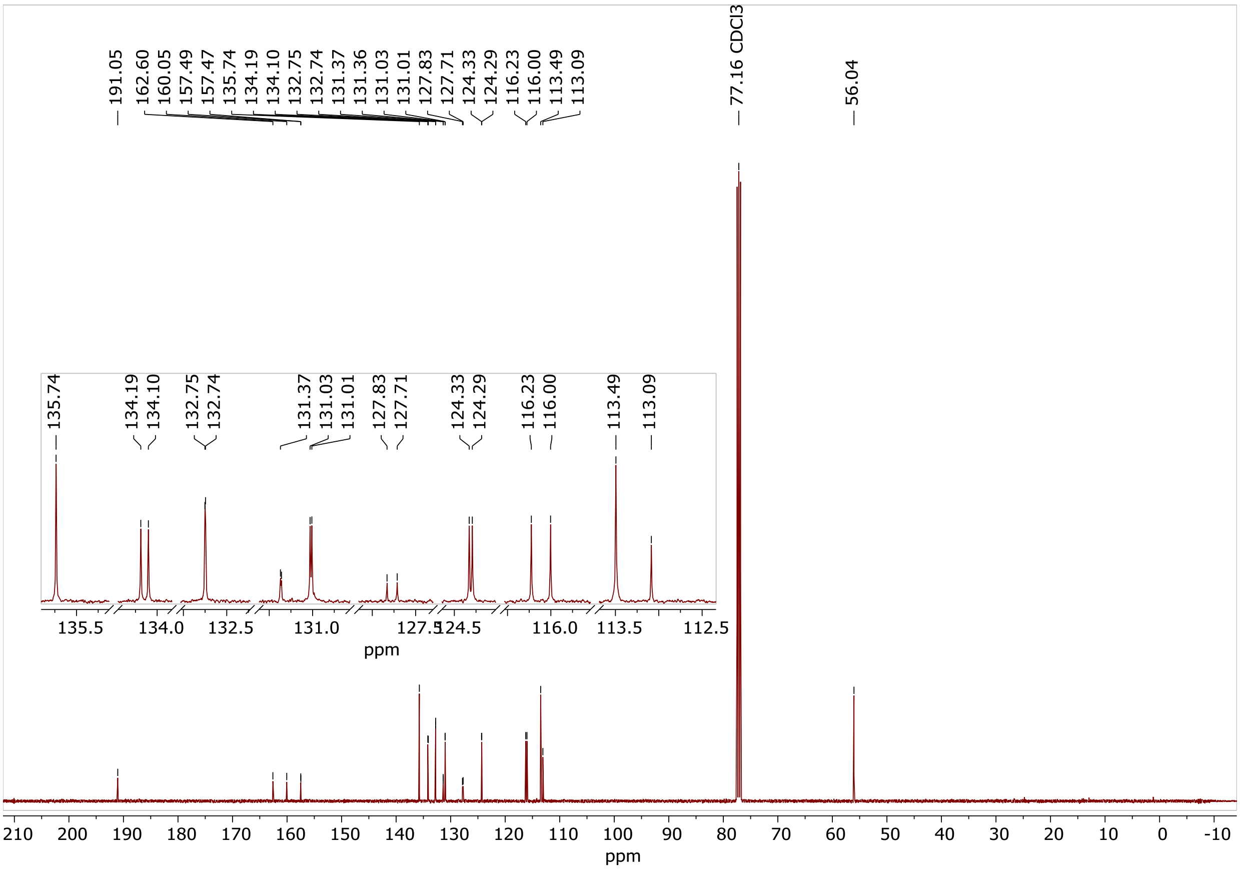

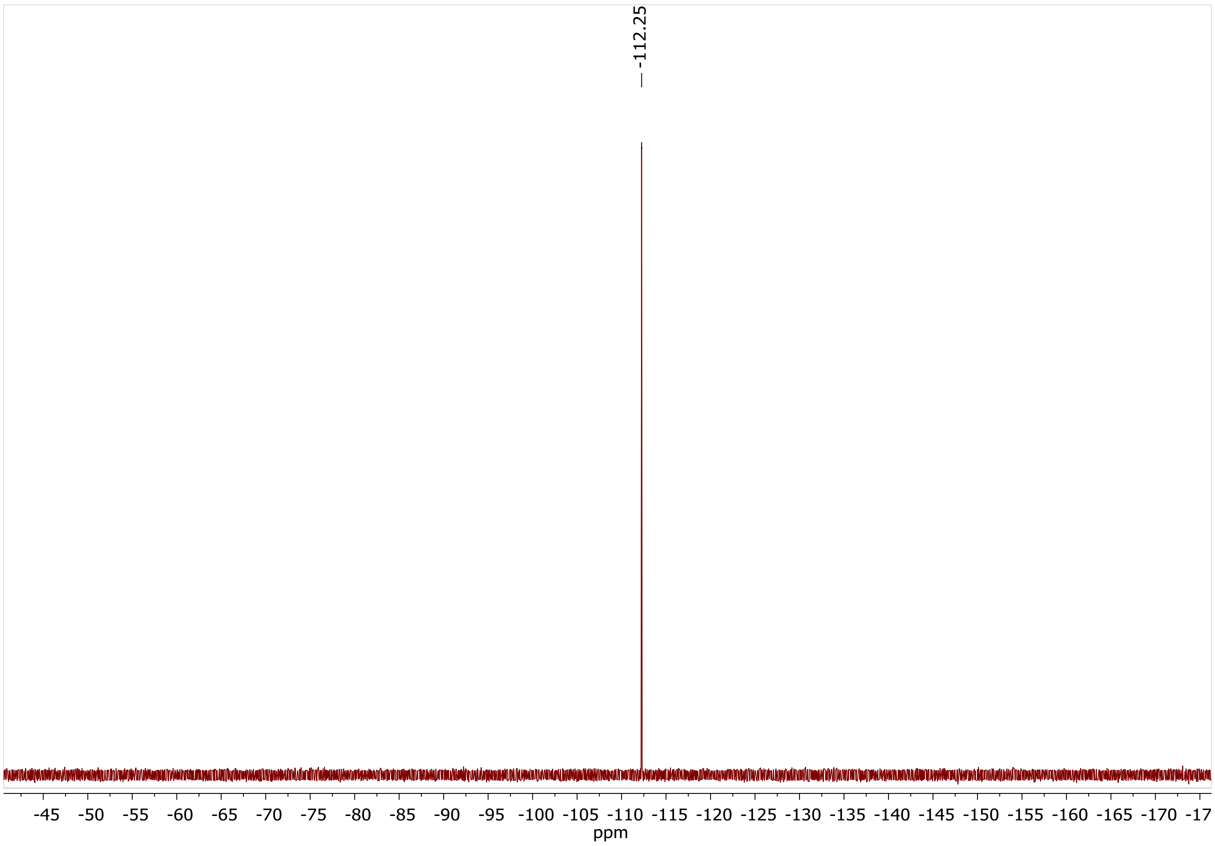
-112.25
-45 -50 -55 -60 -65 -70 -75 -80 -85 -90 -95 -100 -105 -110 -115 -120 -125 -130 -135 -140 -145 -150 -155 -160 -165 -170 -17
ppm

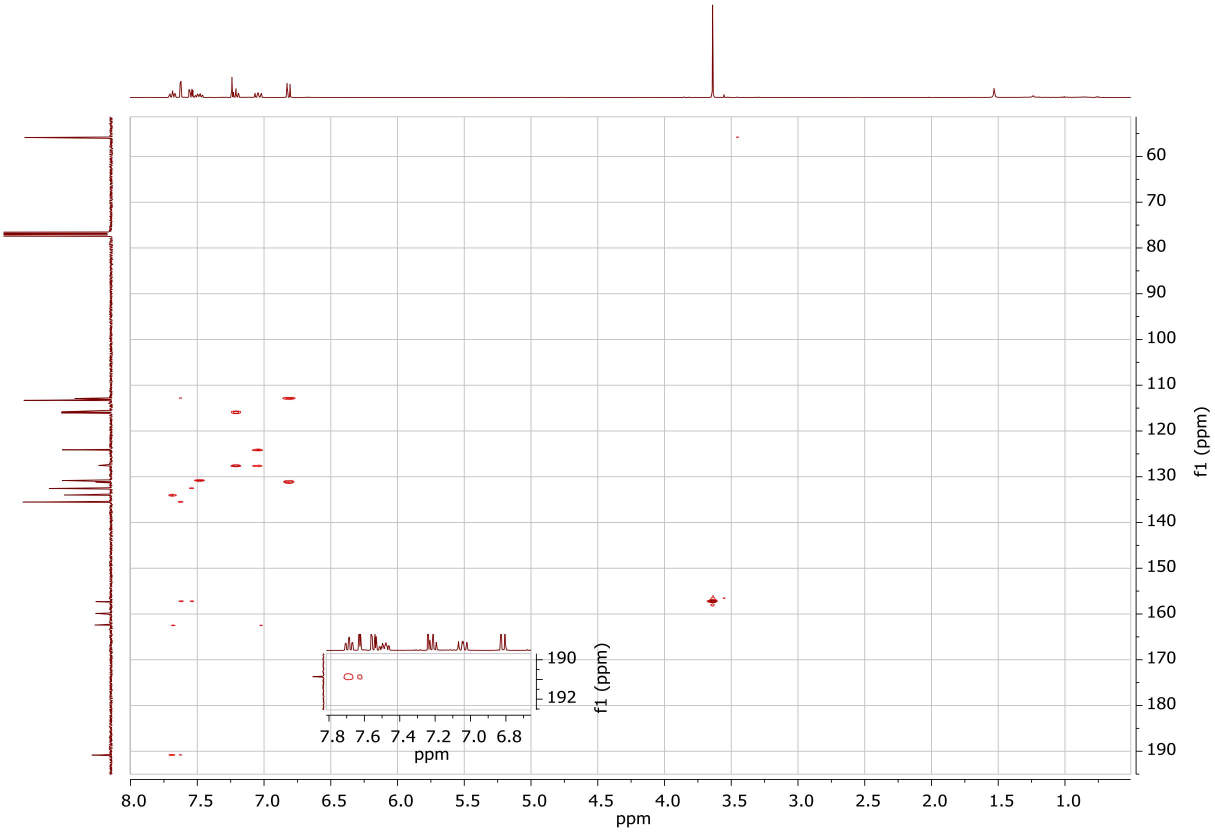
f1 (ppm)
ppm

^{1}H, ^{13}C and ^{19}F NMR spectra of (5-bromo-2-hydroxyphenyl)(2-fluorophenyl)methanone

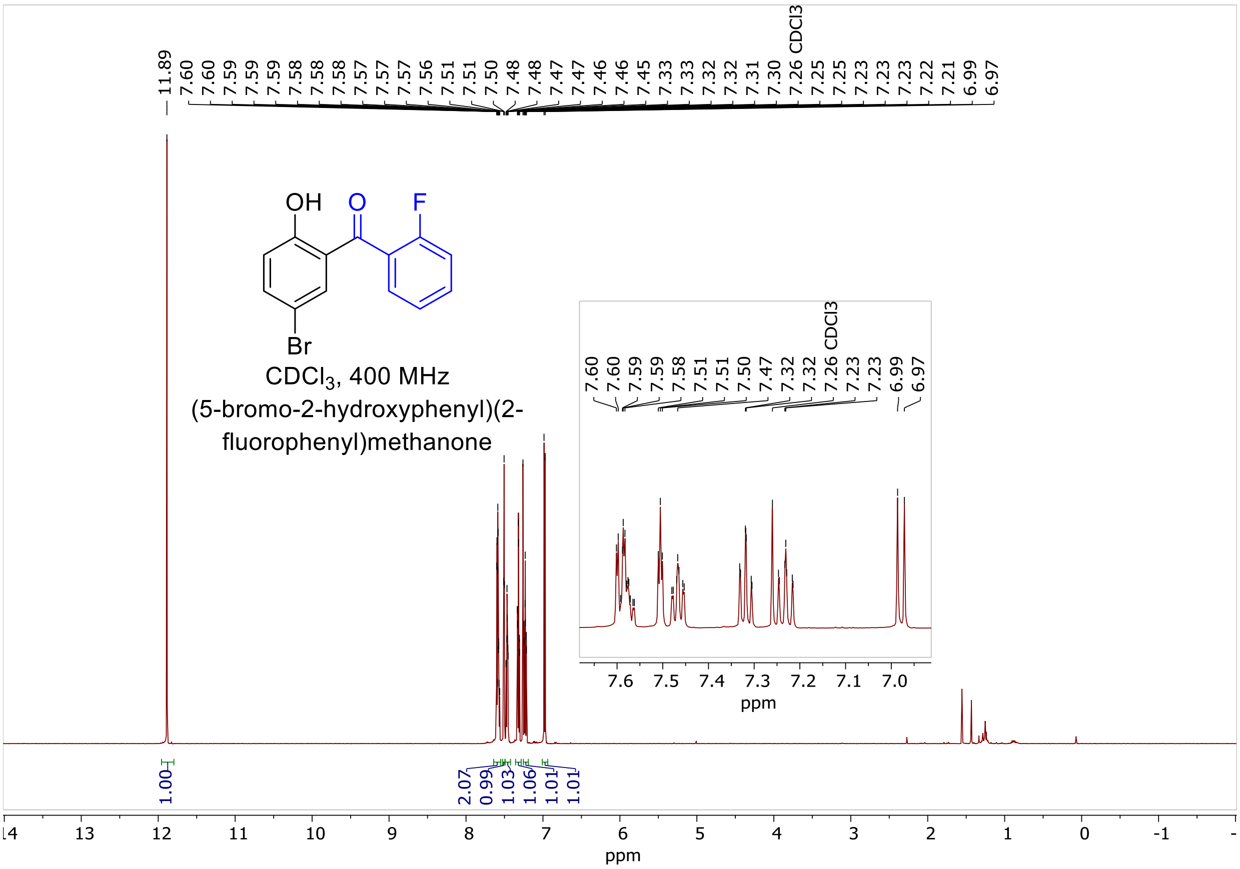

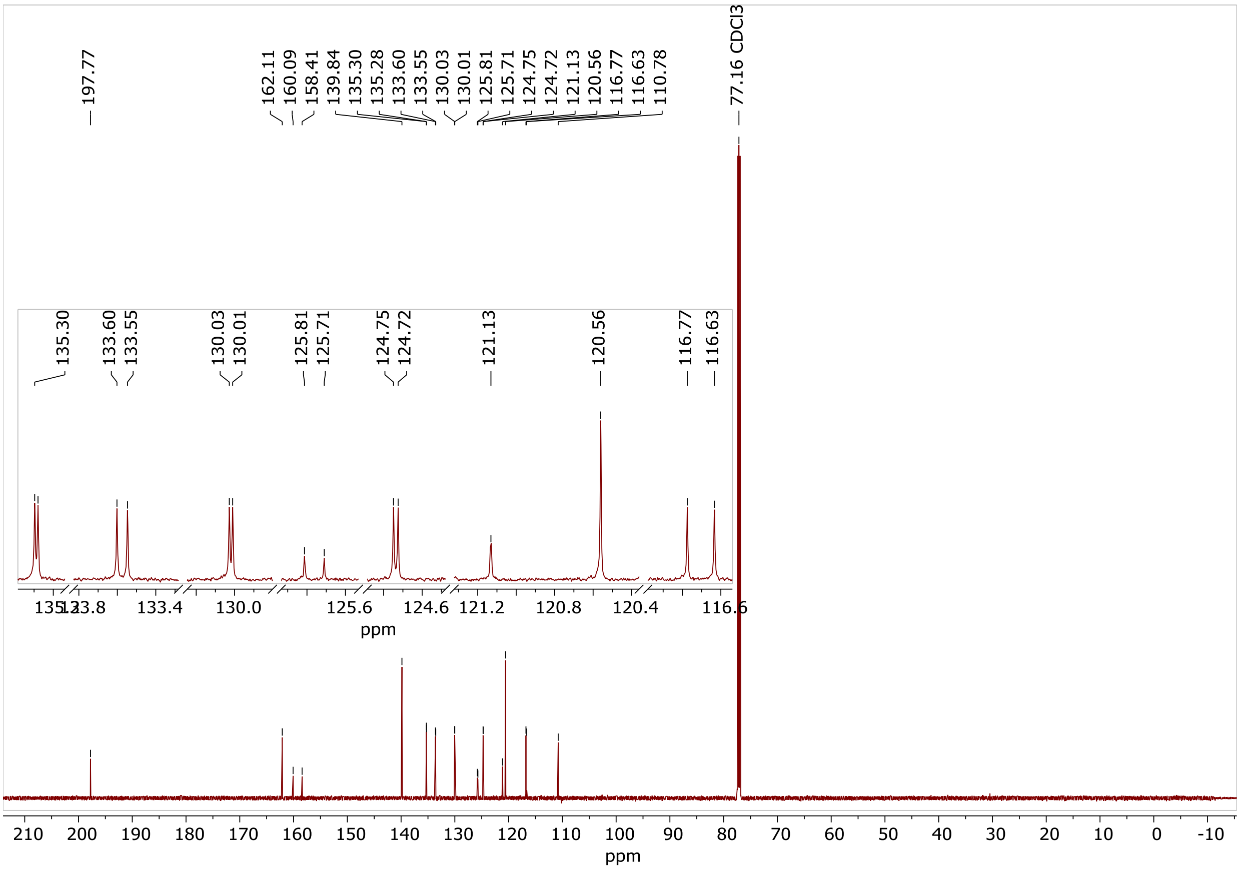

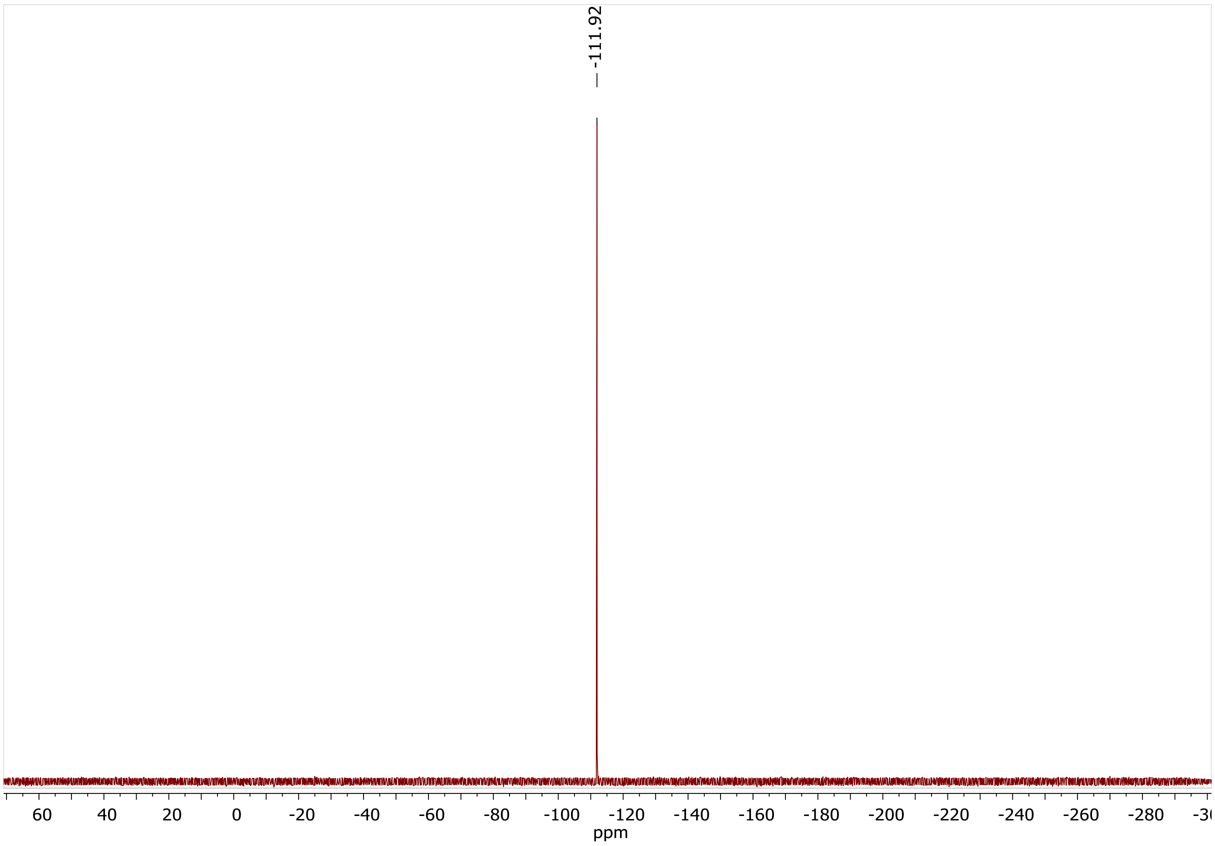

^{1}H and ^{13}C NMR spectra of compound **5ab**

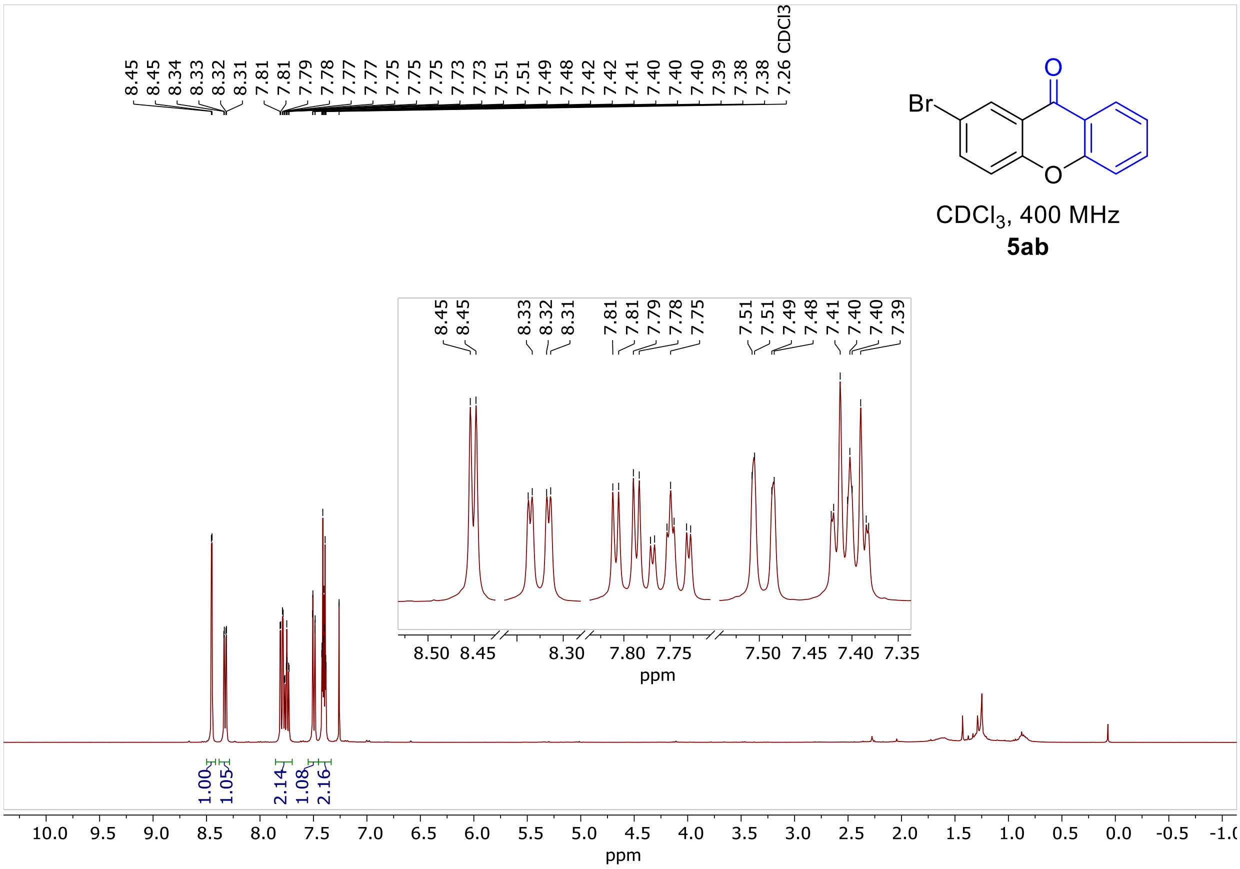

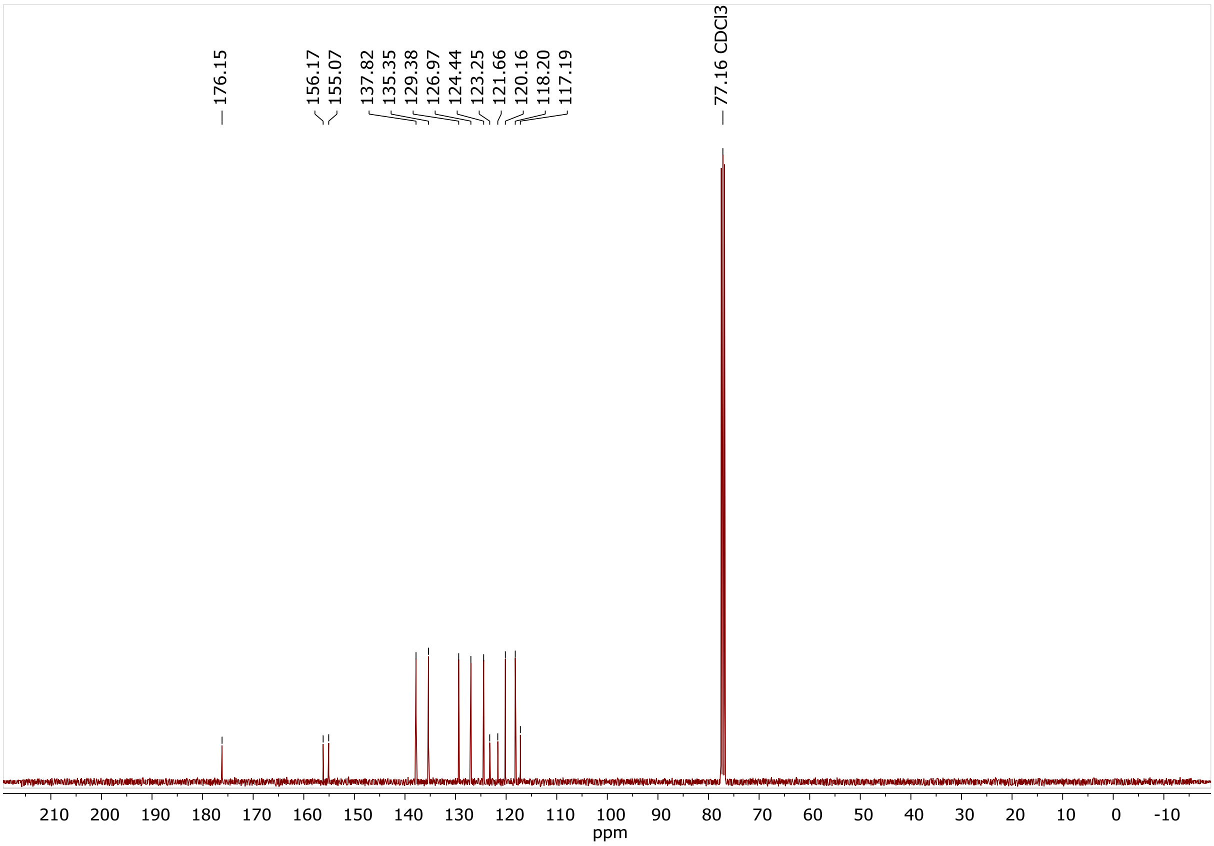

^{1}H, ^{13}C and HMBC NMR spectra of compound **9a**

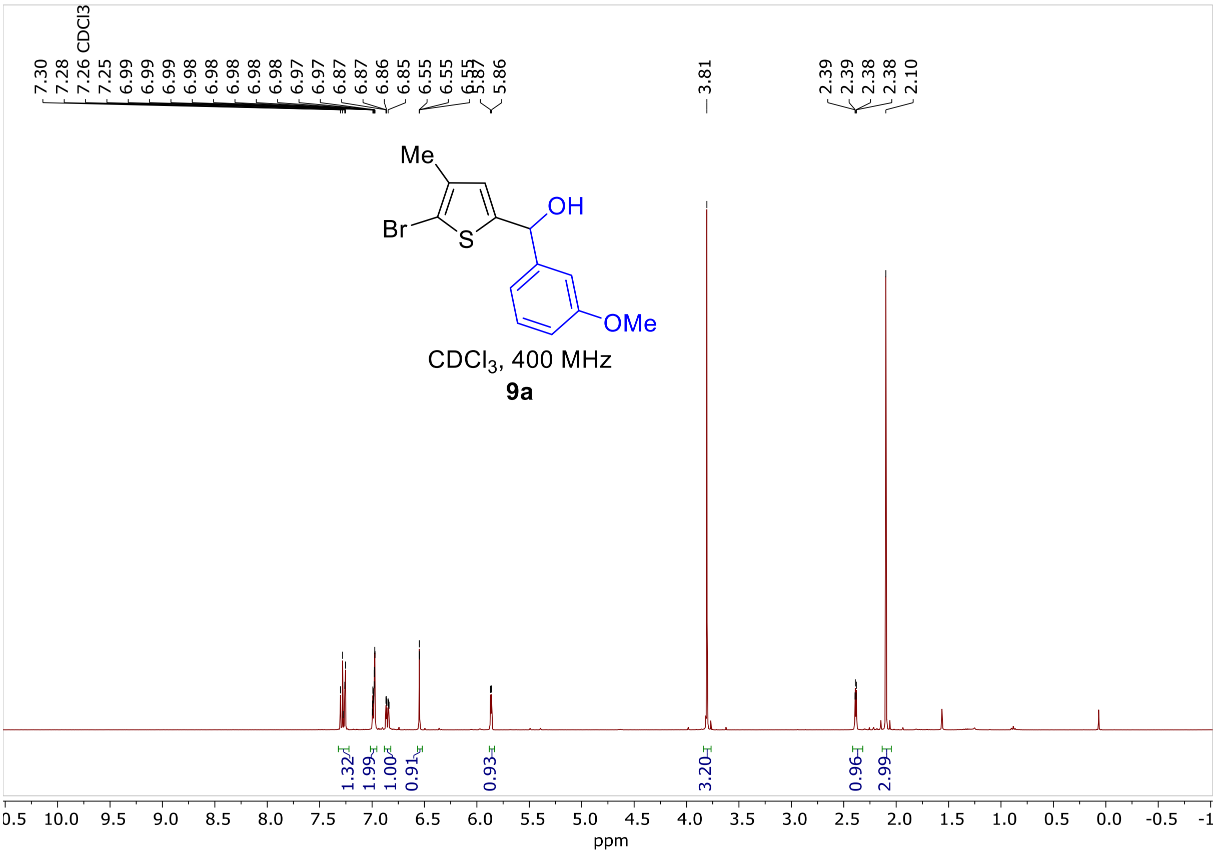

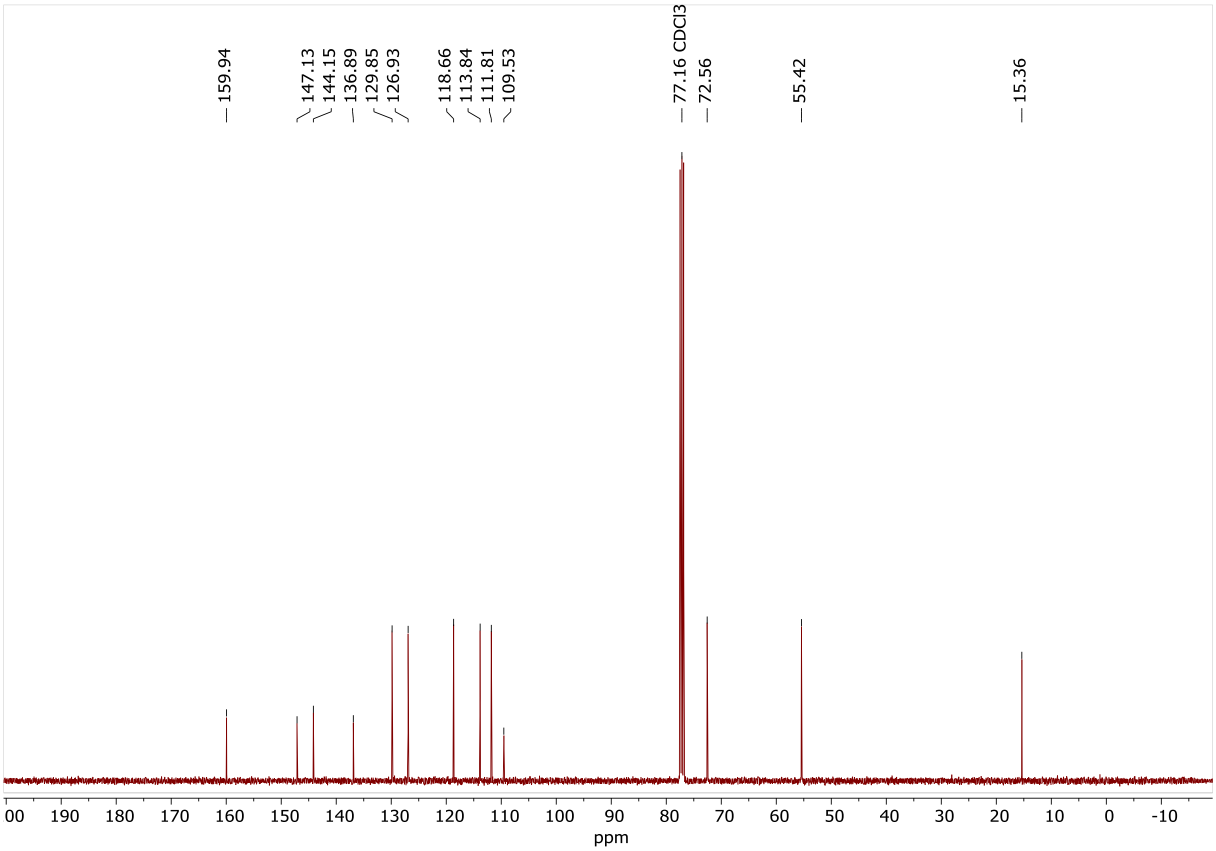
159.94
147.13
144.15
136.89
129.85
126.93
118.66
113.84
111.81
109.53
77.16 CDCl3
72.56
55.42
15.36
ppm

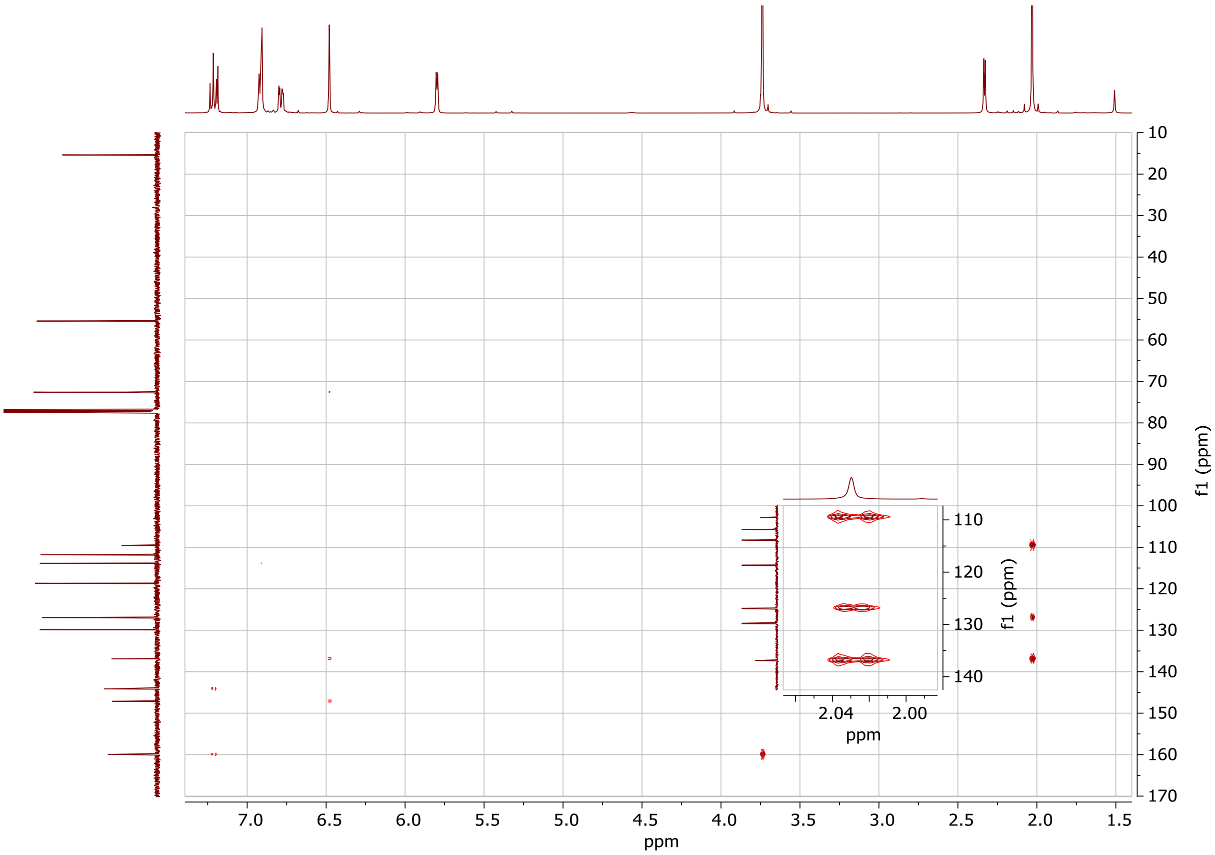
ppm
f1 (ppm)

^1H, ^{13}C and HMBC NMR spectra of compound **9b**

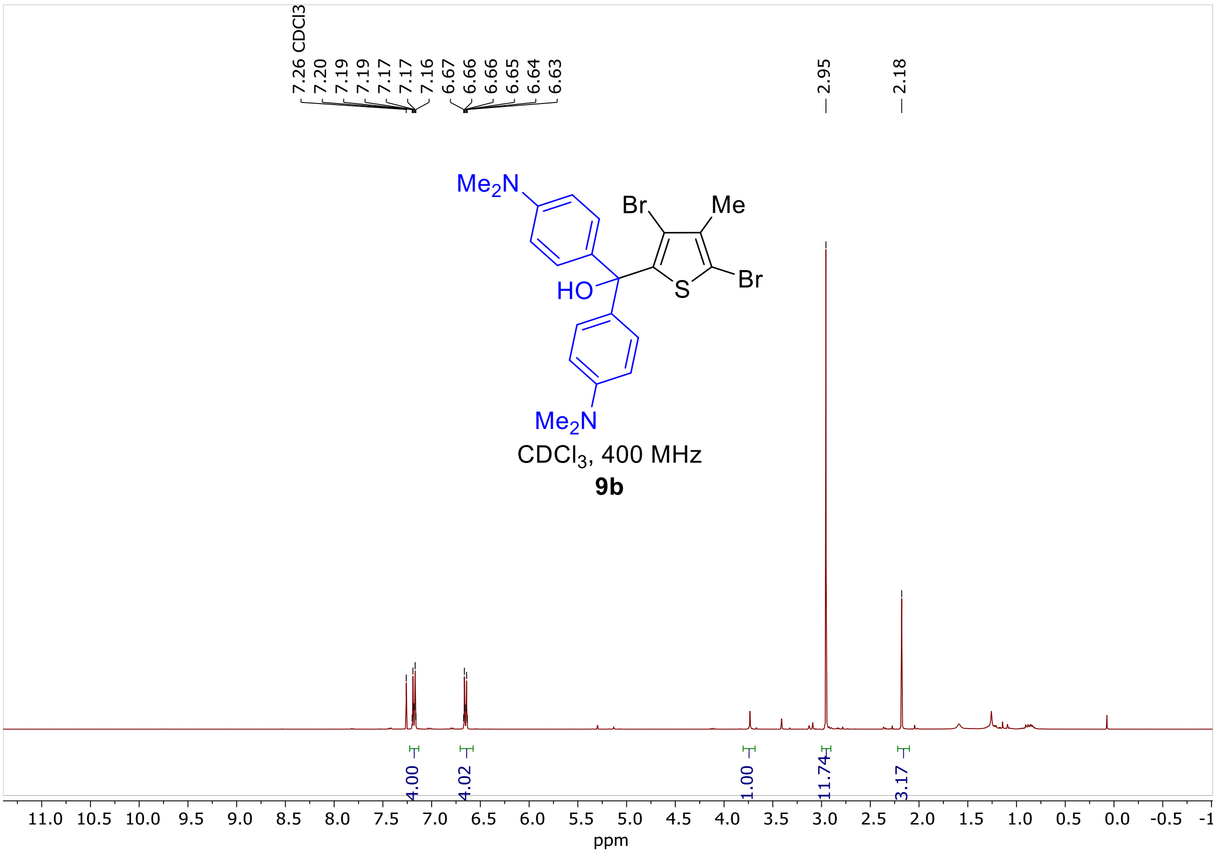

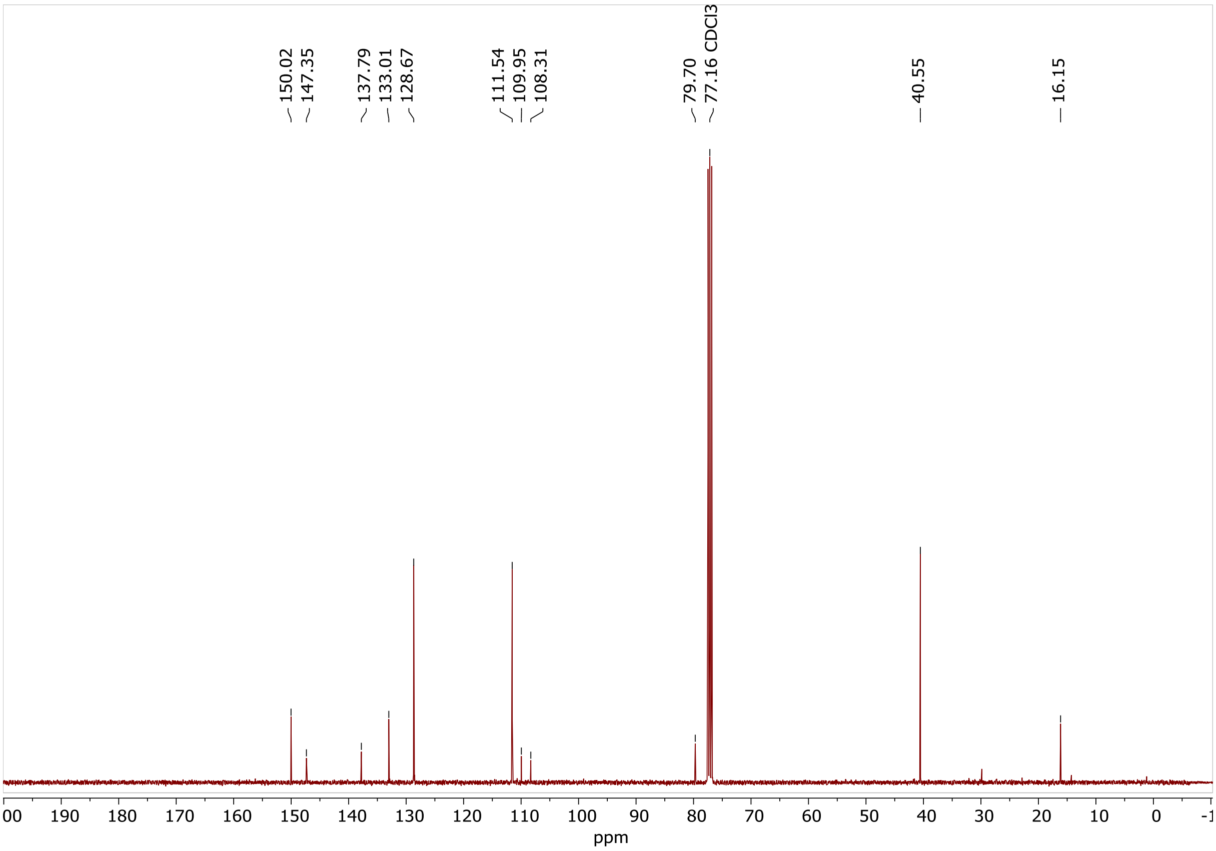

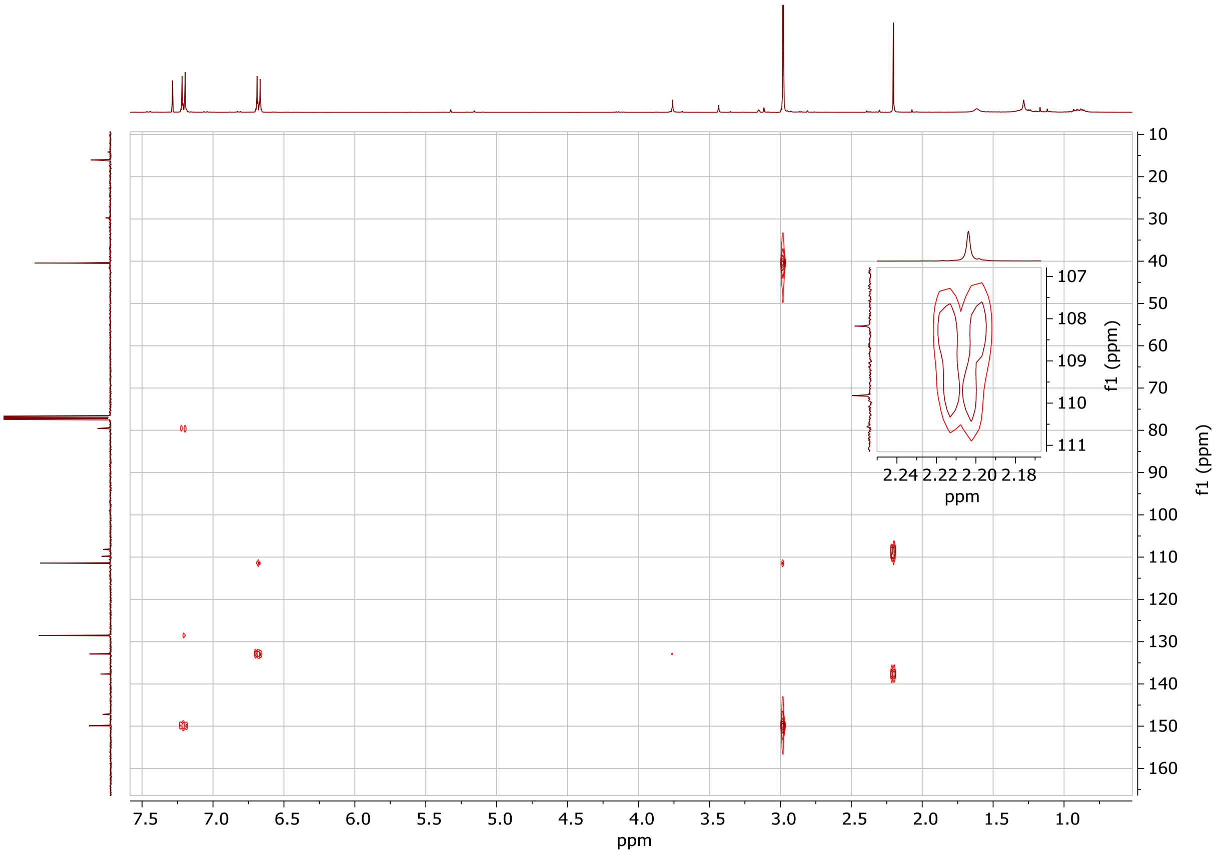

^{1}H, ^{13}C and HMBC NMR spectra of compound **9c**

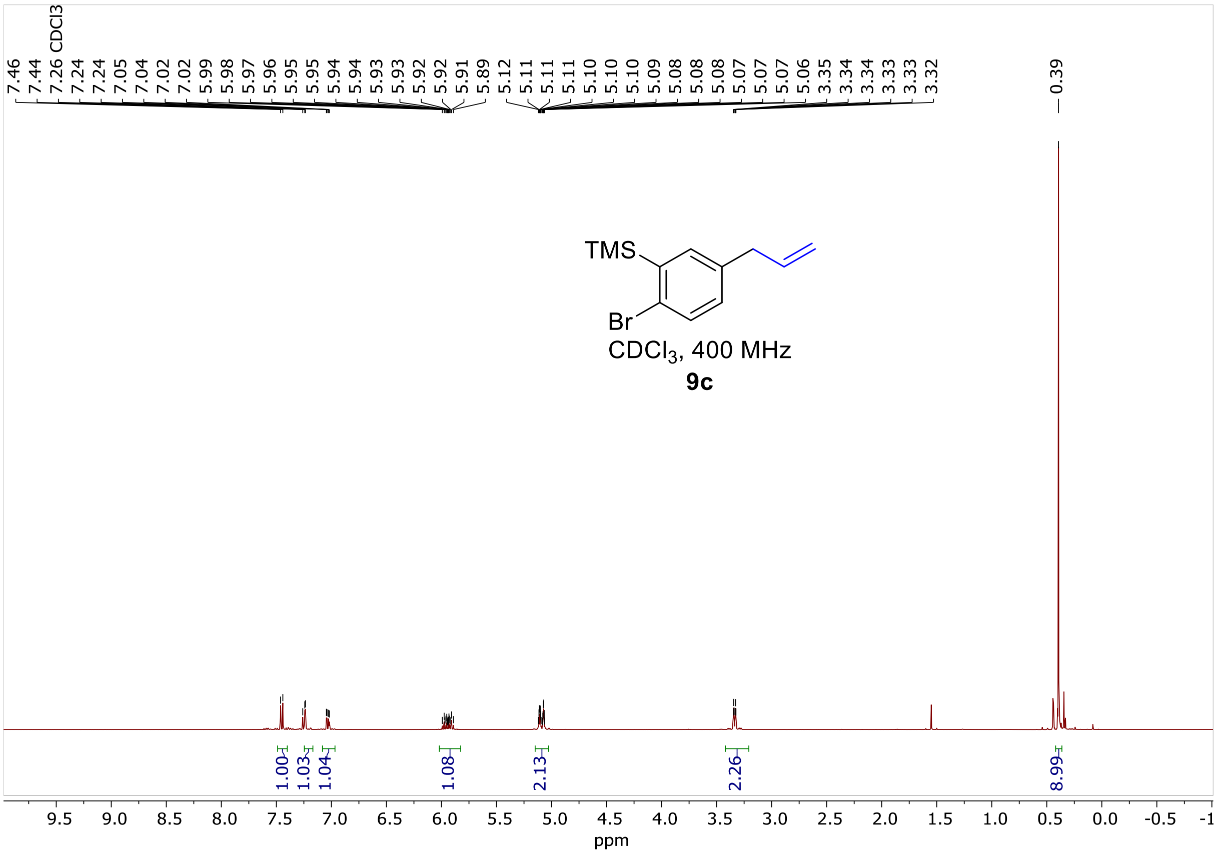

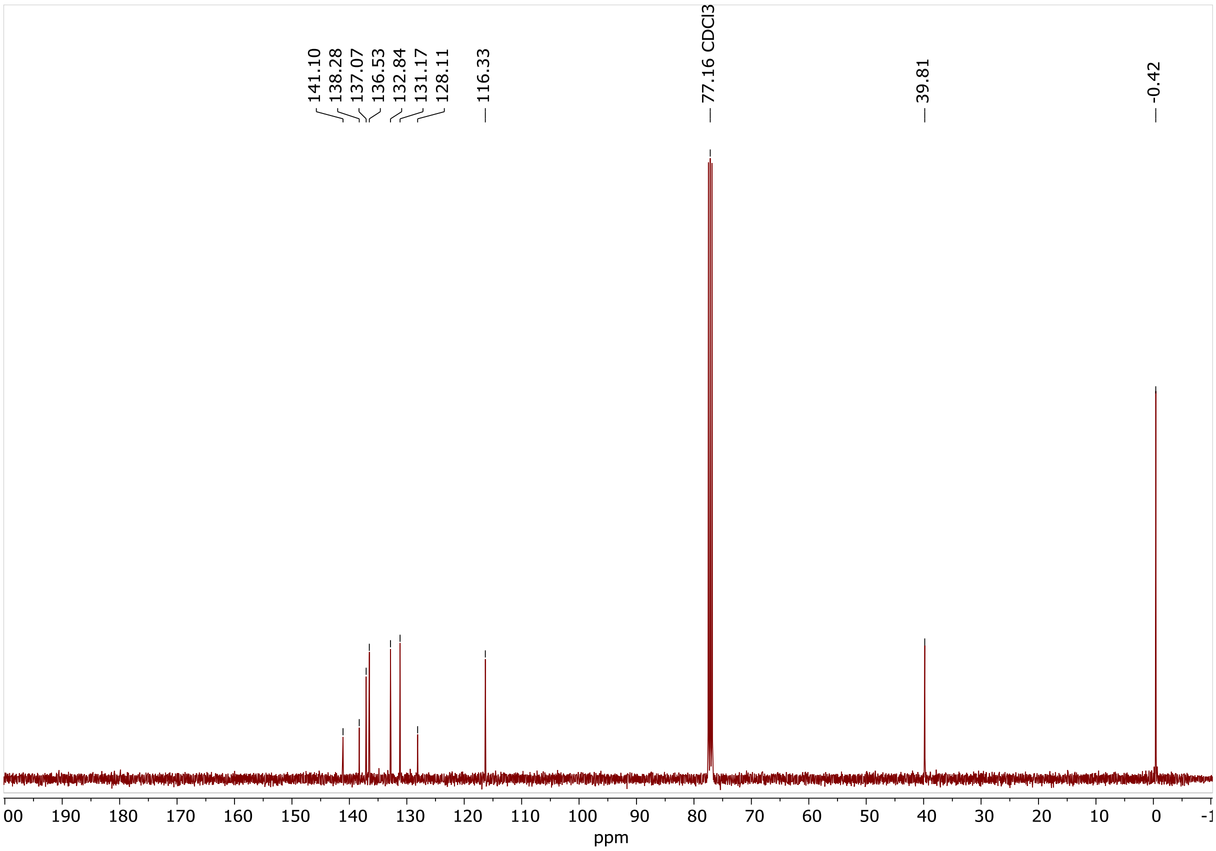
141.10
138.28
137.07
136.53
132.84
131.17
128.11
116.33
77.16 CDCl3
39.81
-0.42
ppm

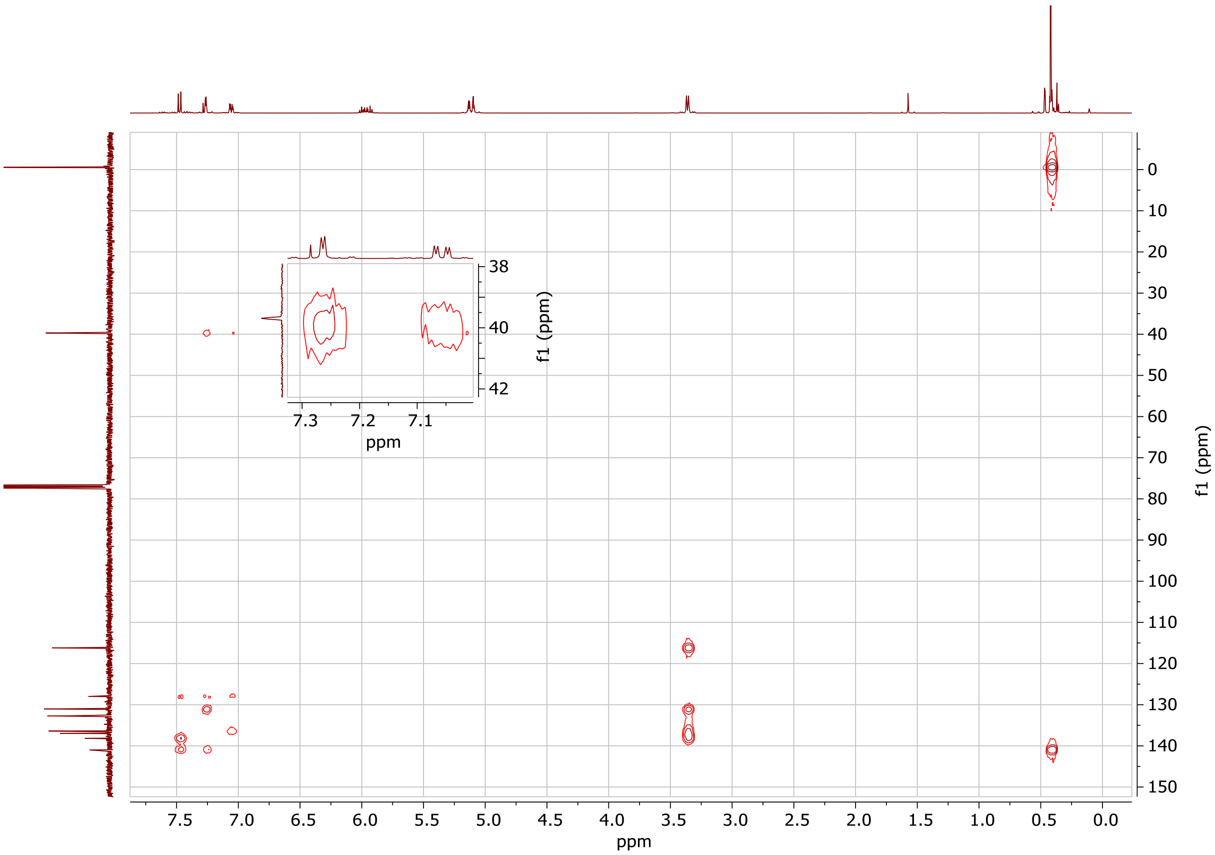
ppm
f1 (ppm)
ppm
f1 (ppm)

^{1}H, ^{13}C and HMBC NMR spectra of compound **9d**

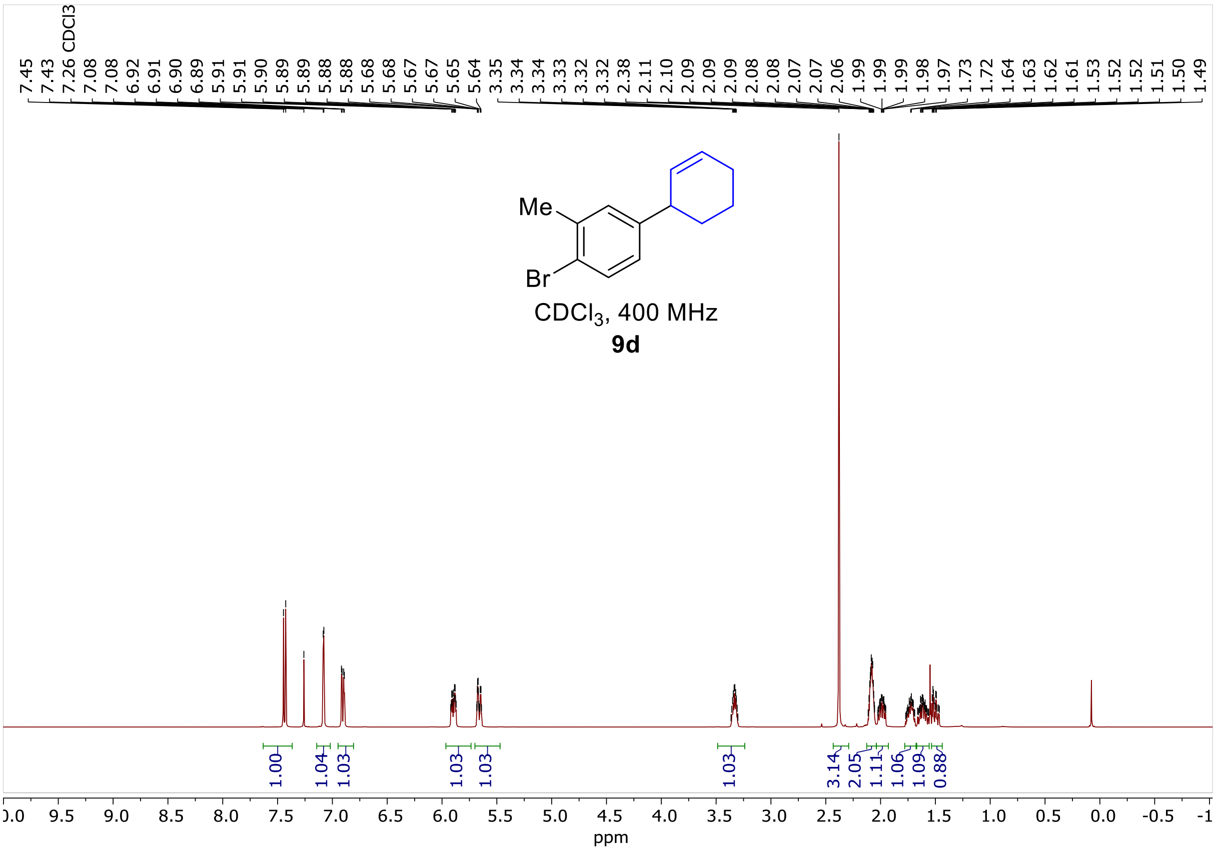

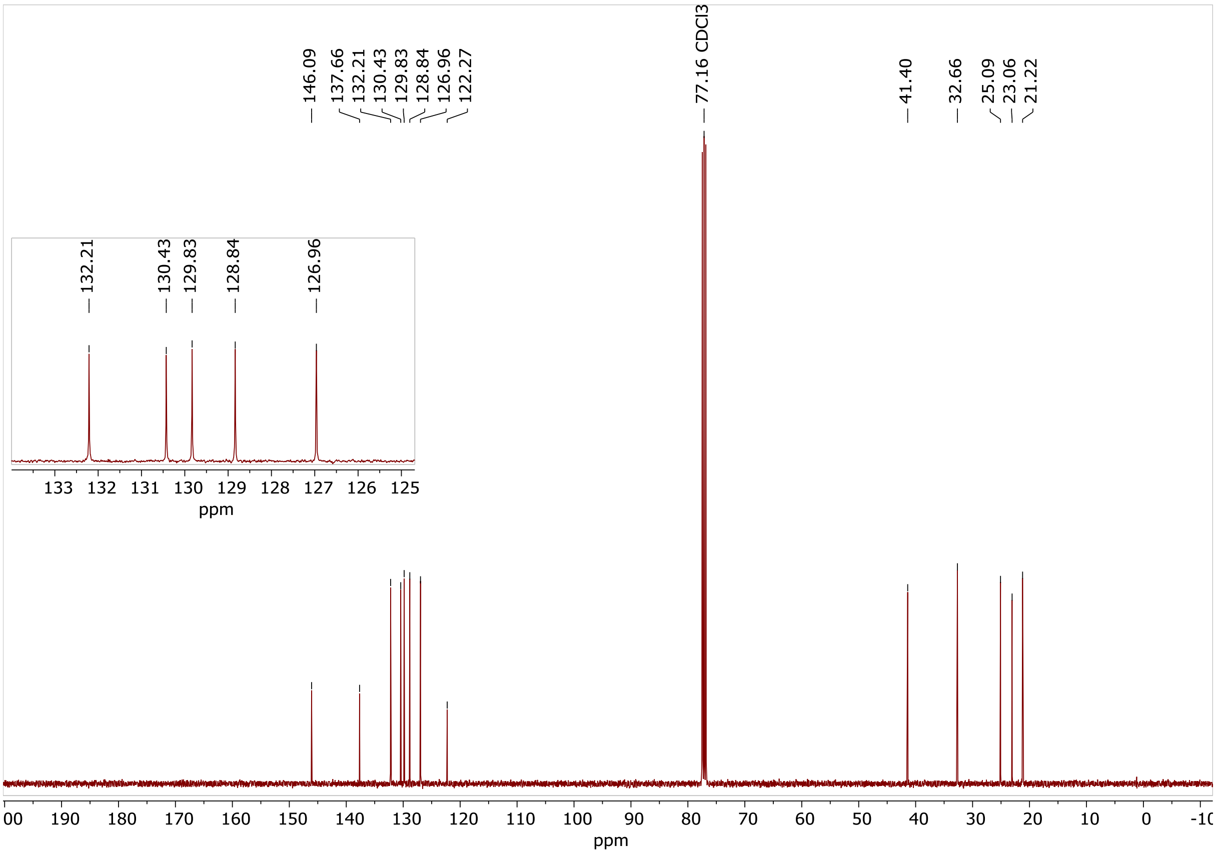

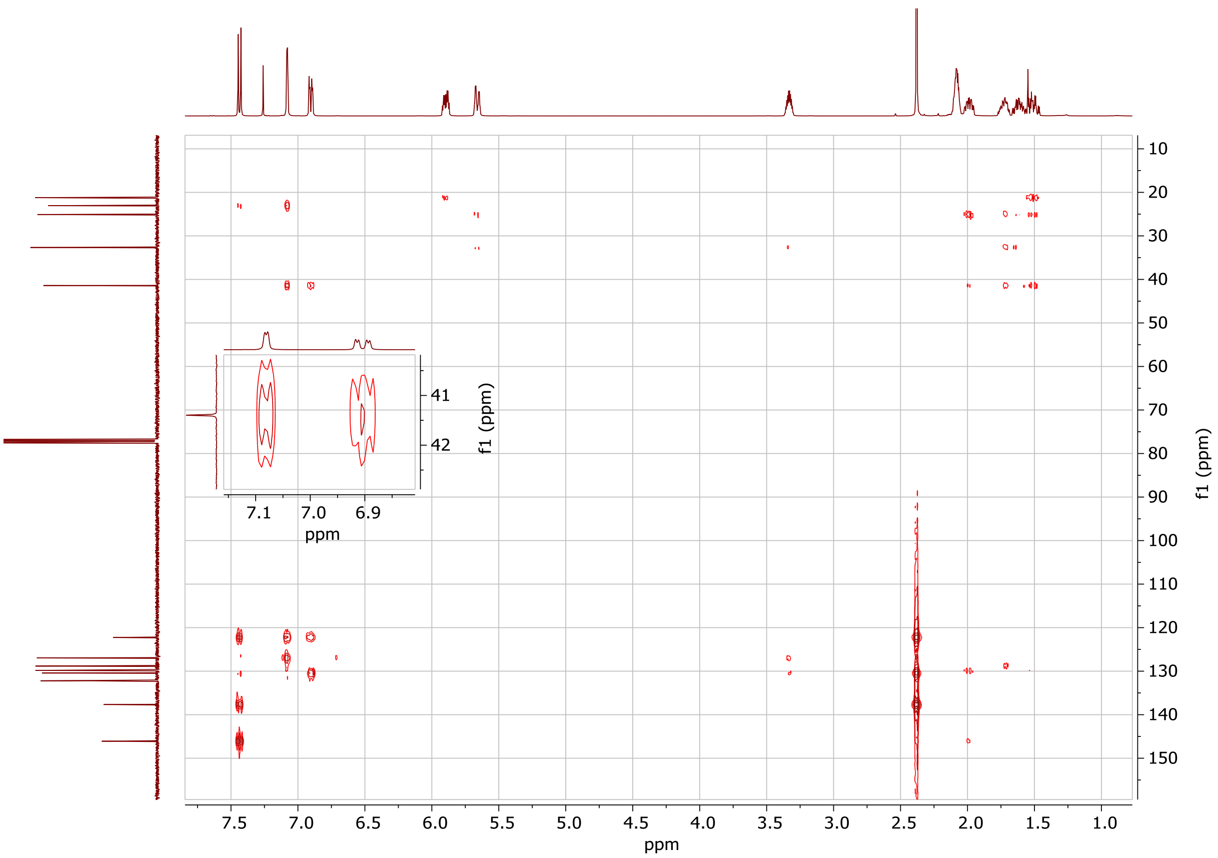

^{1}H, ^{13}C and HMBC NMR spectra of compound **13a**

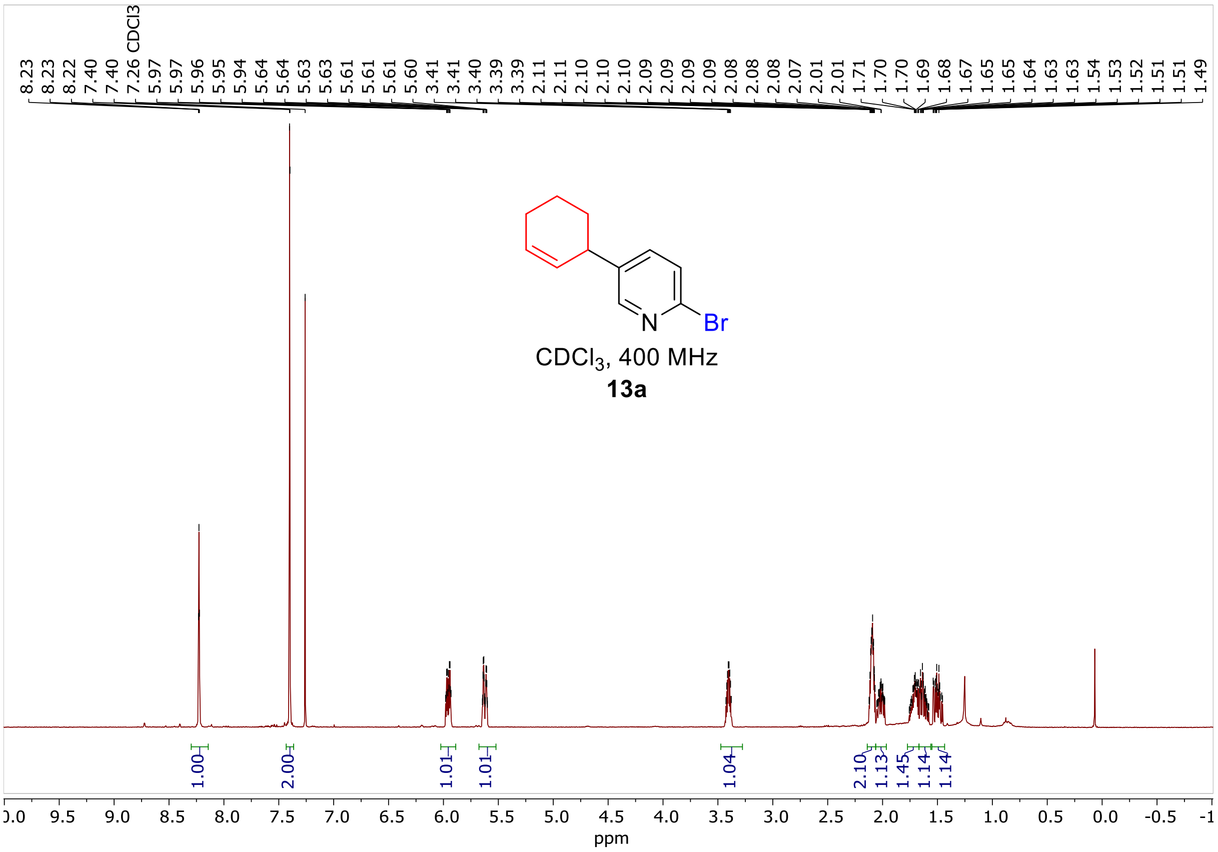

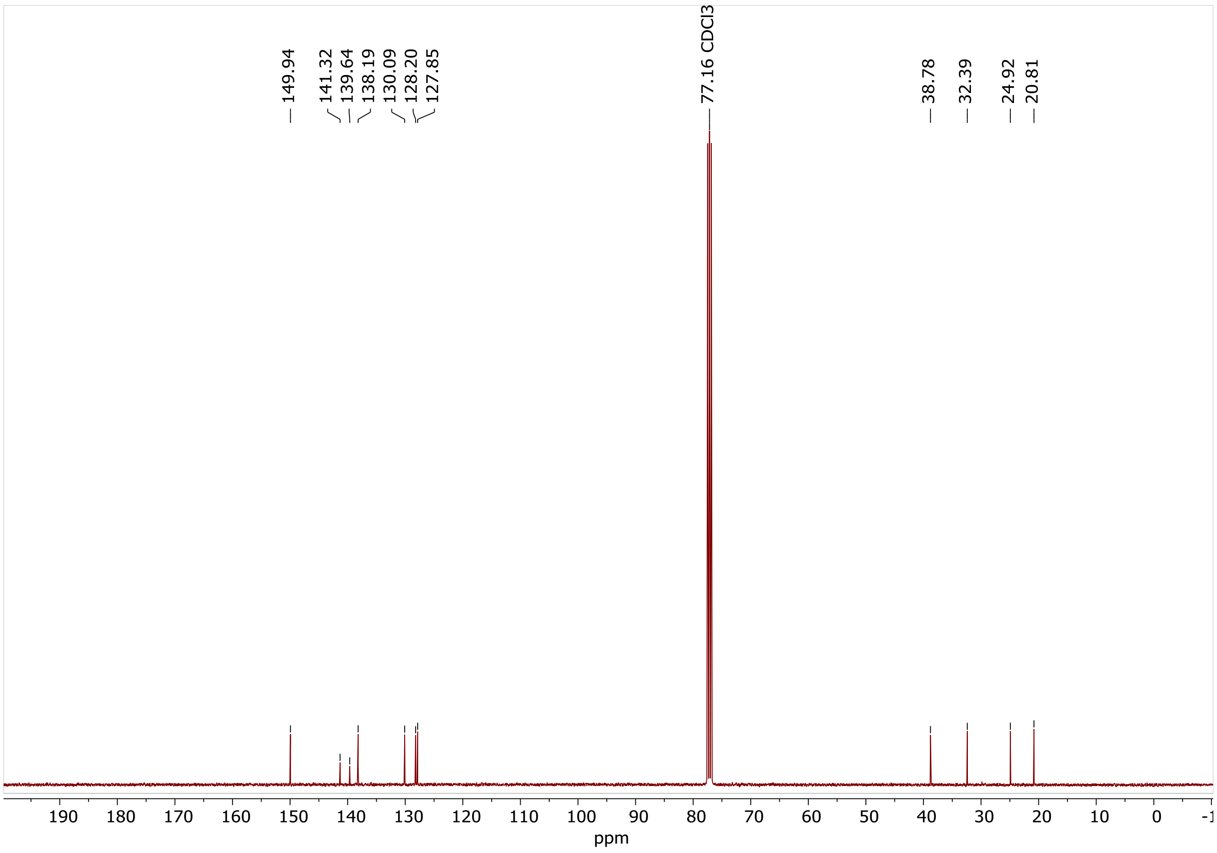
149.94
141.32
139.64
138.19
130.09
128.20
127.85
77.16 CDCl3
38.78
32.39
24.92
20.81
190 180 170 160 150 140 130 120 110 100 90 80 70 60 50 40 30 20 10 0
ppm

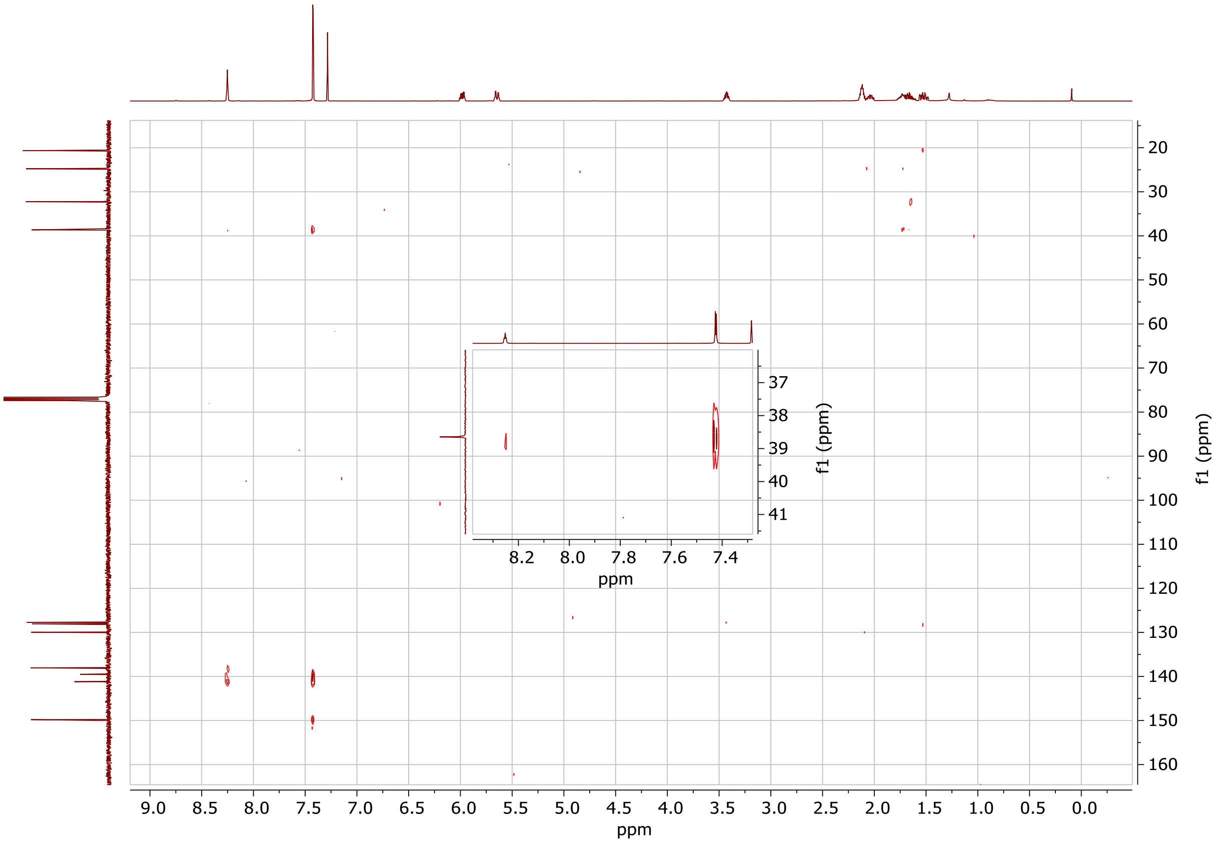
9.0 8.5 8.0 7.5 7.0 6.5 6.0 5.5 5.0 4.5 4.0 3.5 3.0 2.5 2.0 1.5 1.0 0.5 0.0
ppm
f1 (ppm)
20 30 40 50 60 70 80 90 100 110 120 130 140 150 160
8.2 8.0 7.8 7.6 7.4
37 38 39 40 41

^{1}H, ^{13}C and HMBC NMR spectra of compound **13b**

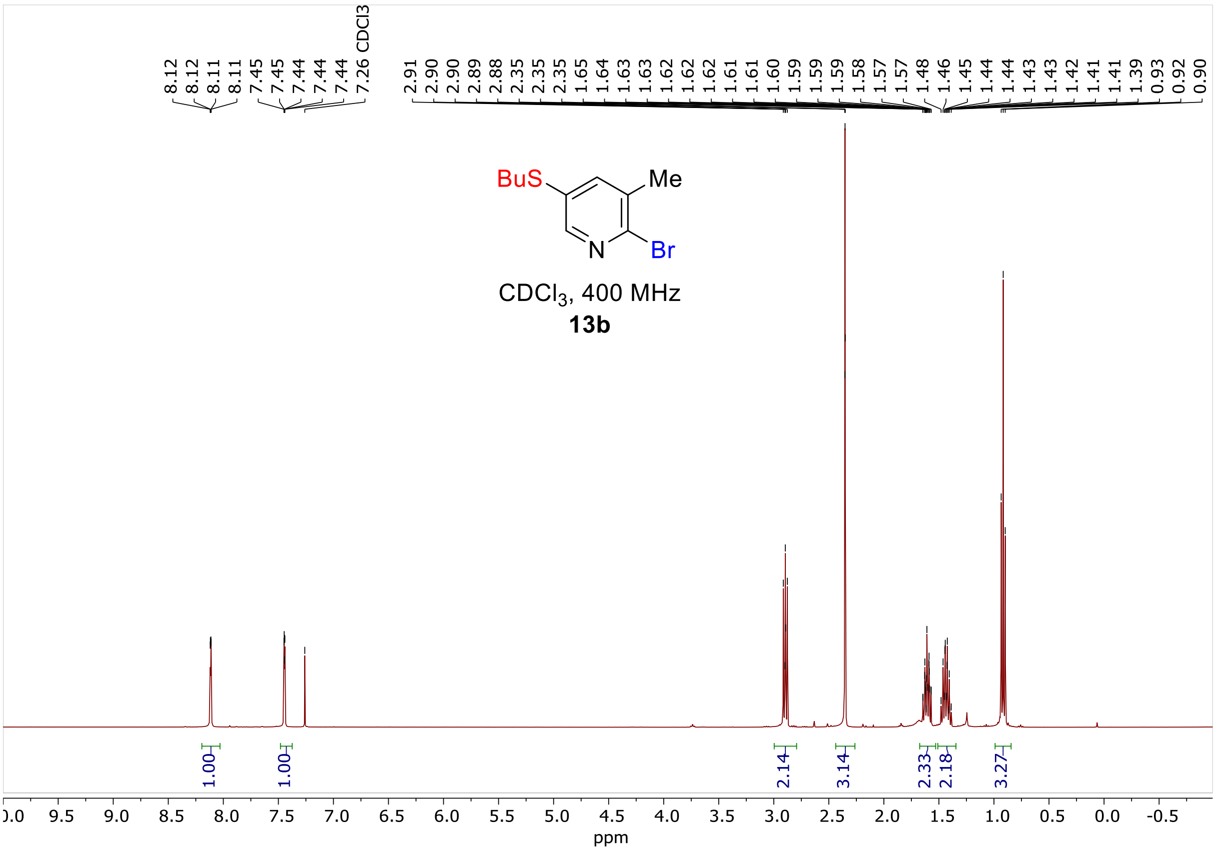

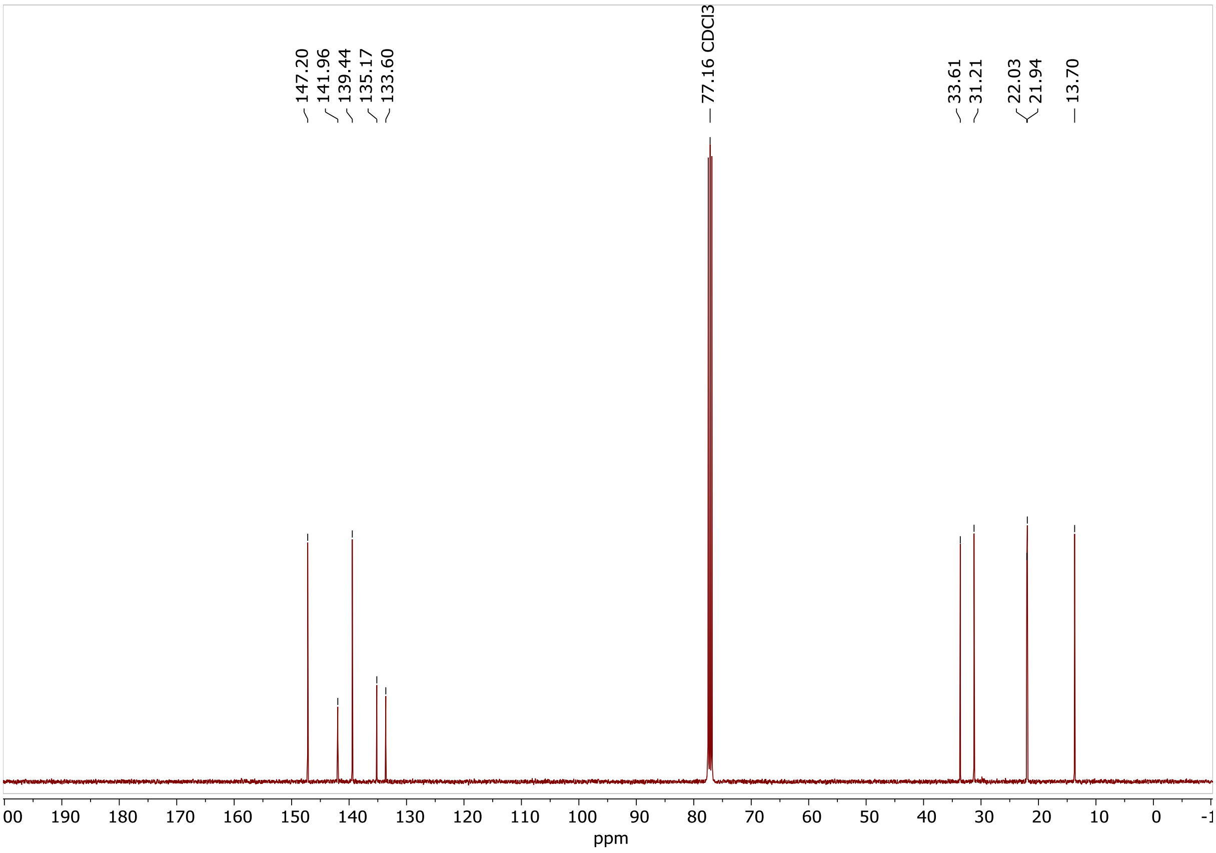

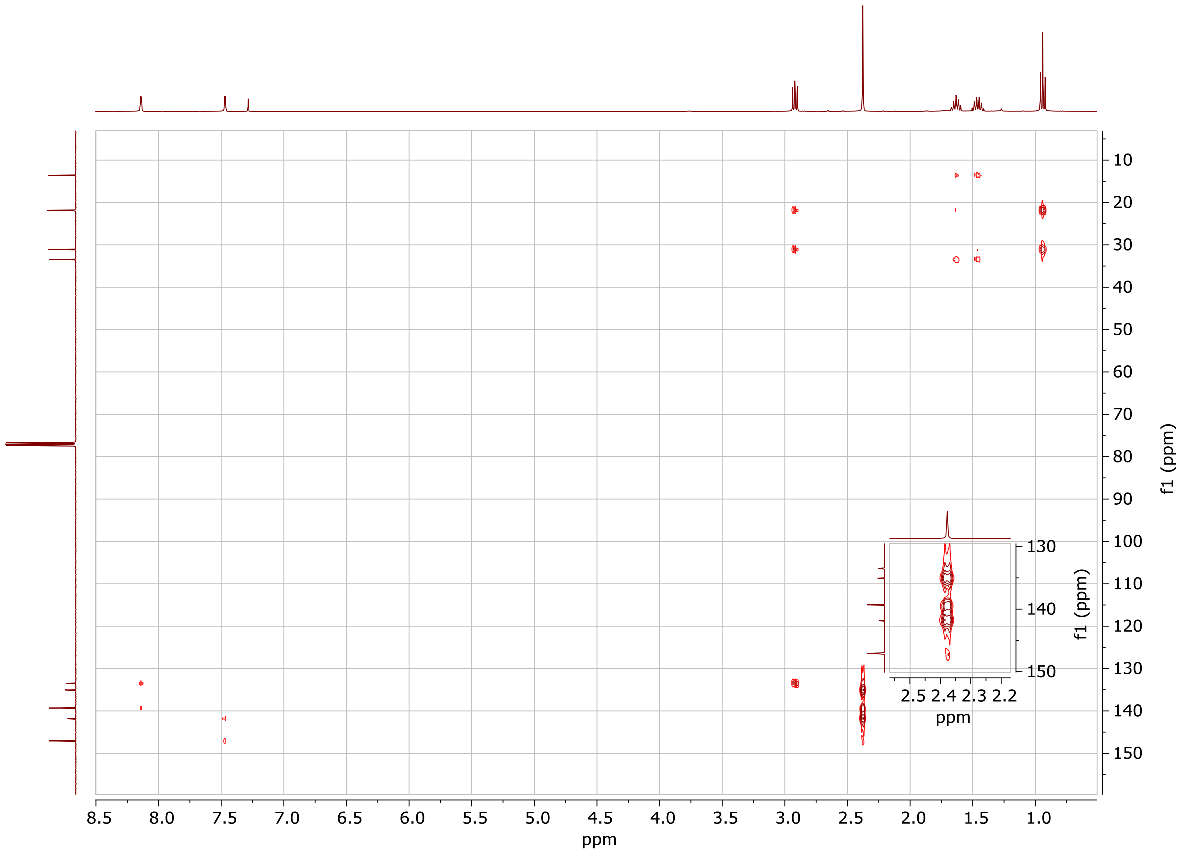

^{1}H, ^{13}C and HMBC NMR spectra of compound **13c**

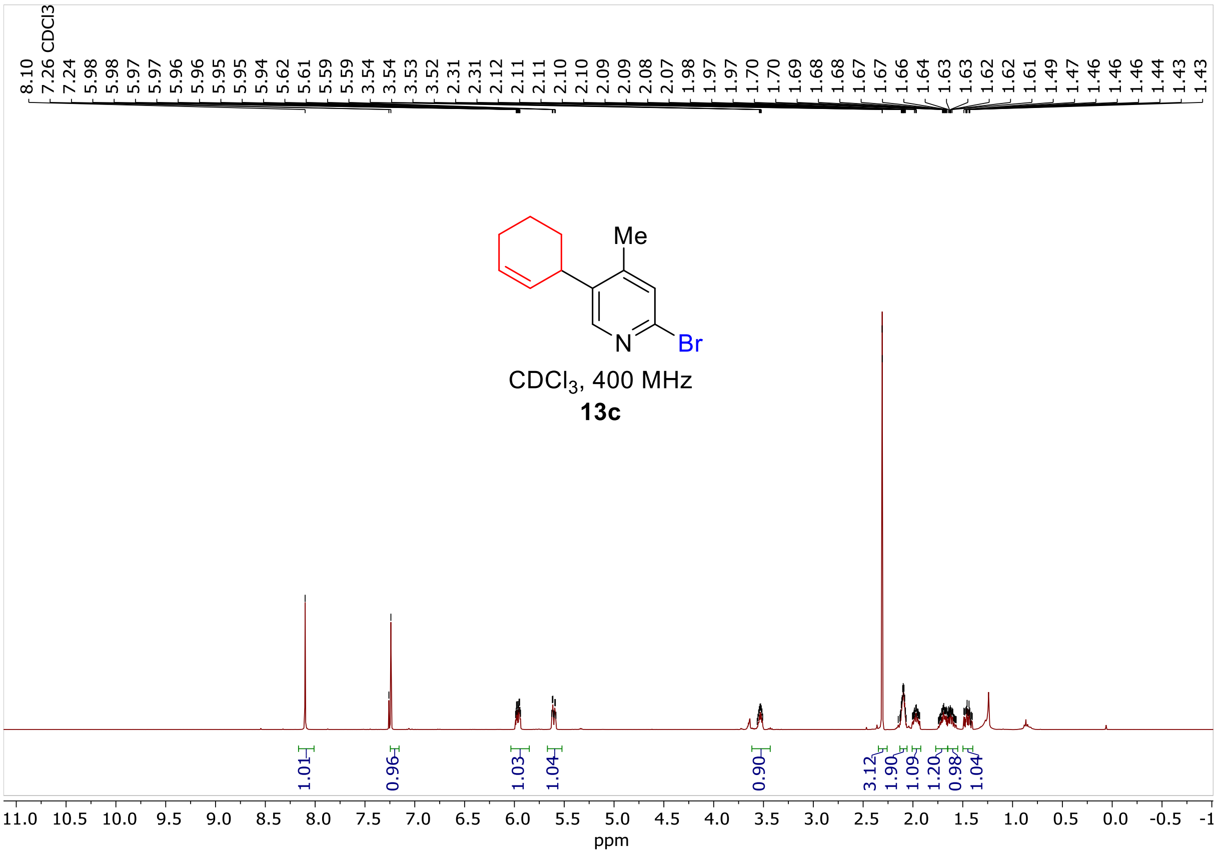

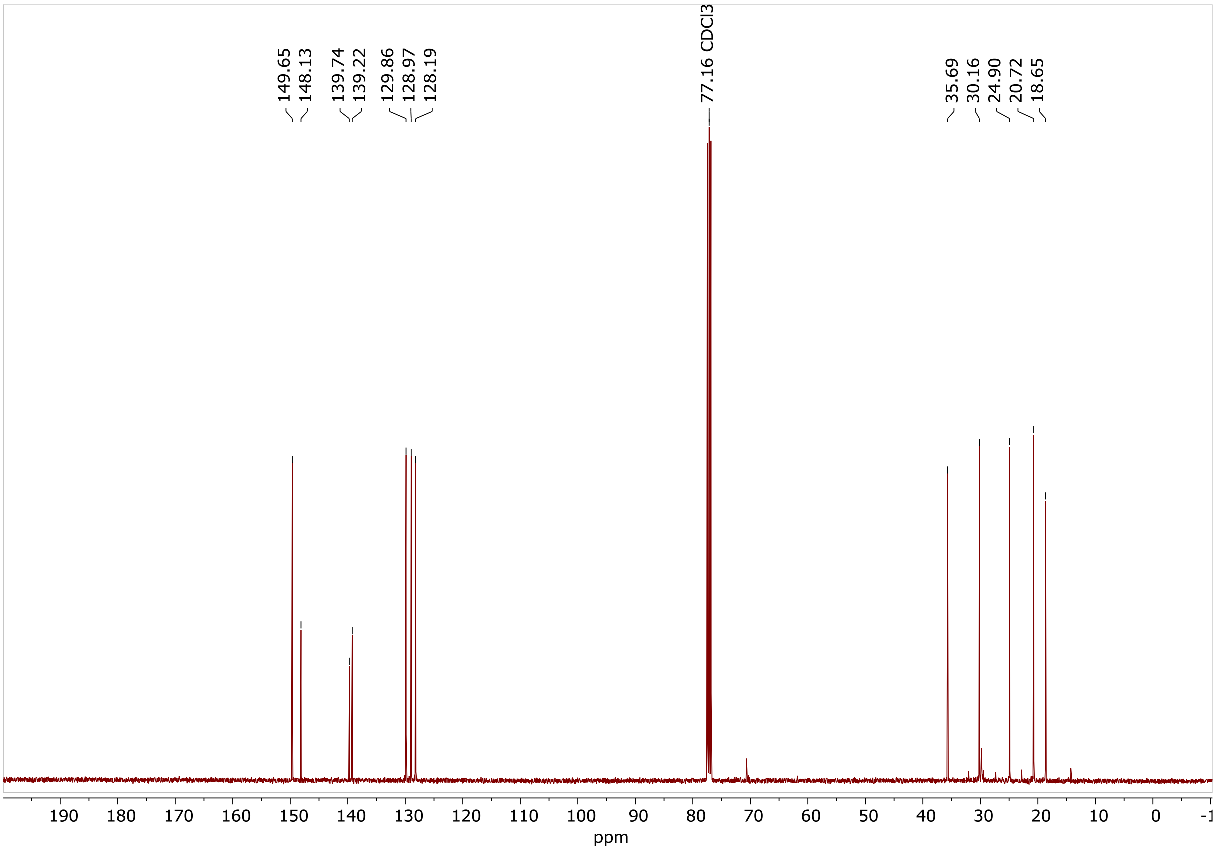
149.65
148.13
139.74
139.22
129.86
128.97
128.19
77.16 CDCl3
35.69
30.16
24.90
20.72
18.65
ppm

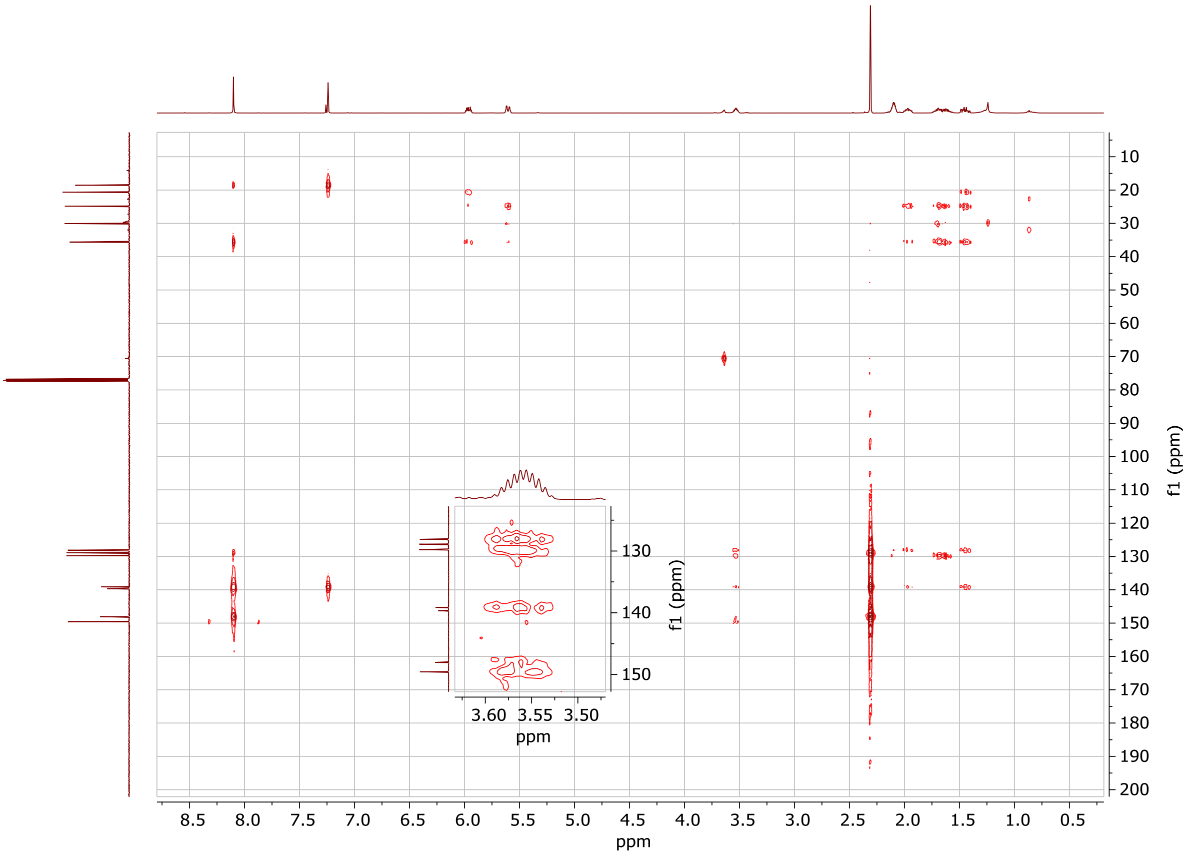
f1 (ppm)
ppm

^{1}H, ^{13}C and HMBC NMR spectra of compound **13d**

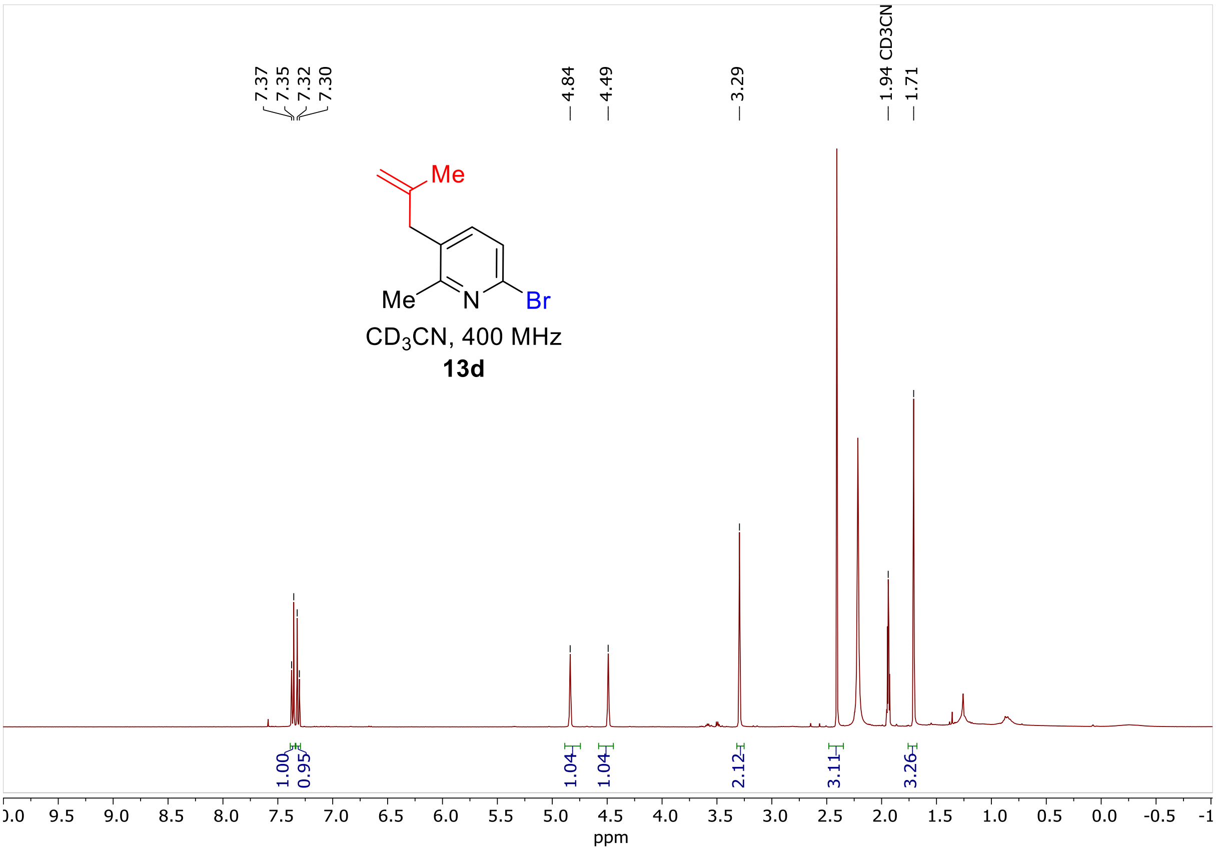

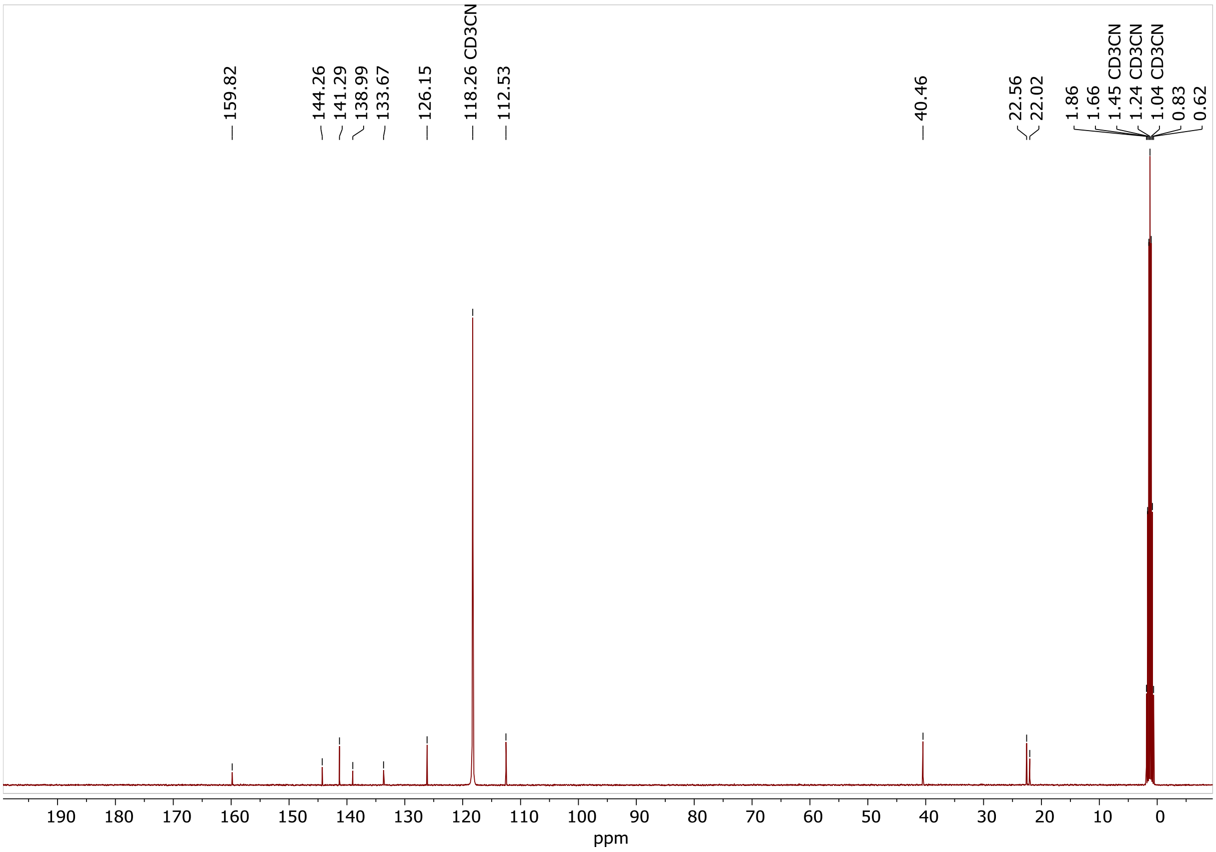

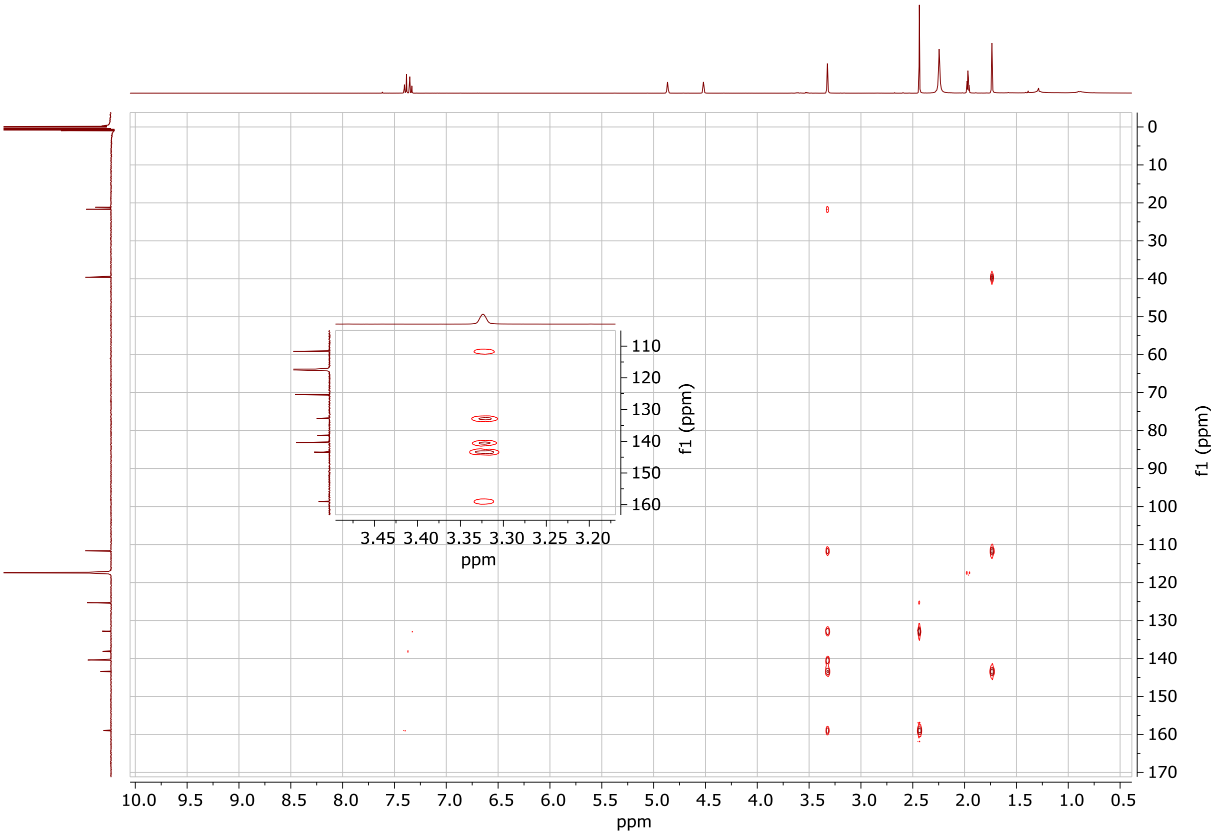

^{1}H, ^{13}C, ^{19}F and HMBC NMR spectra of compound **13e**

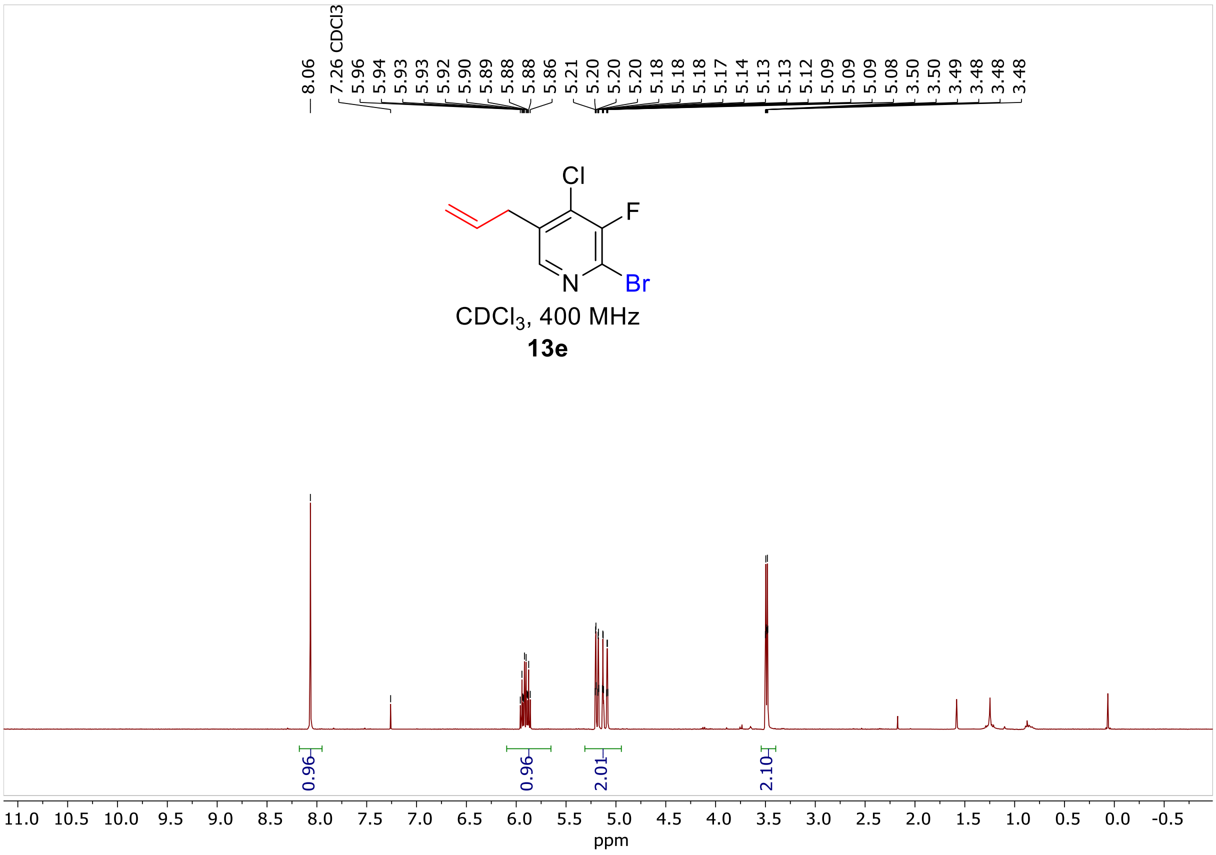

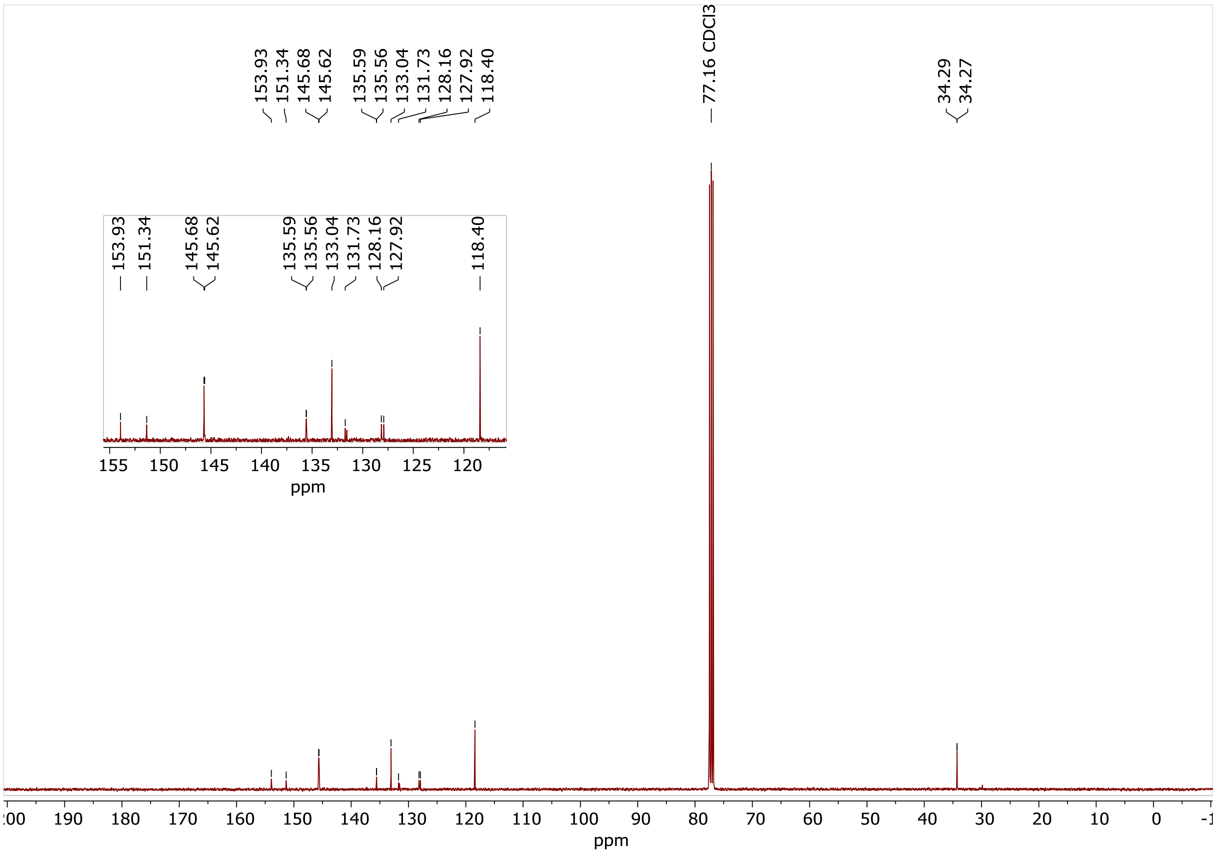
153.93
151.34
145.68
145.62
135.59
135.56
133.04
131.73
128.16
127.92
118.40
77.16 CDCl3
34.29
34.27
155 150 145 140 135 130 125 120
ppm
190 180 170 160 150 140 130 120 110 100 90 80 70 60 50 40 30 20 10 0
ppm

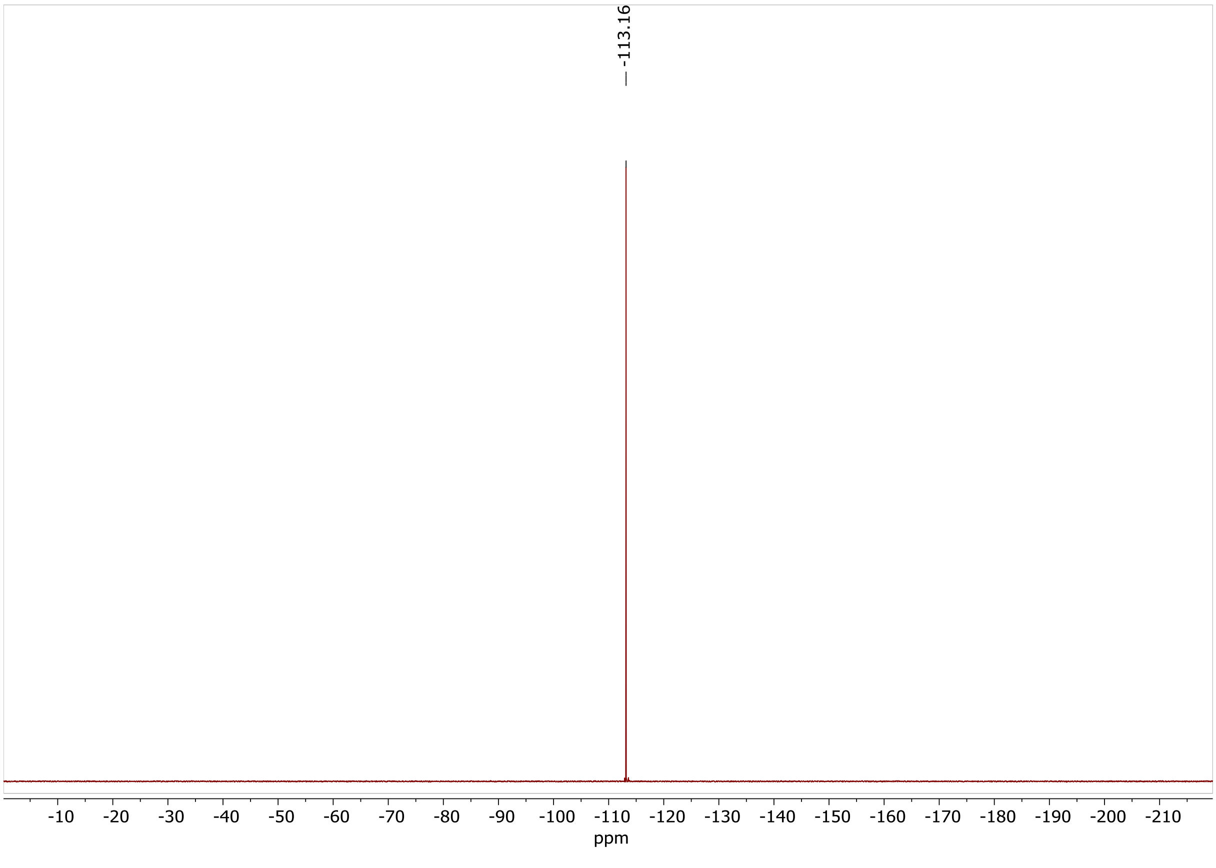
-113.16
-10 -20 -30 -40 -50 -60 -70 -80 -90 -100 -110 -120 -130 -140 -150 -160 -170 -180 -190 -200 -210
ppm

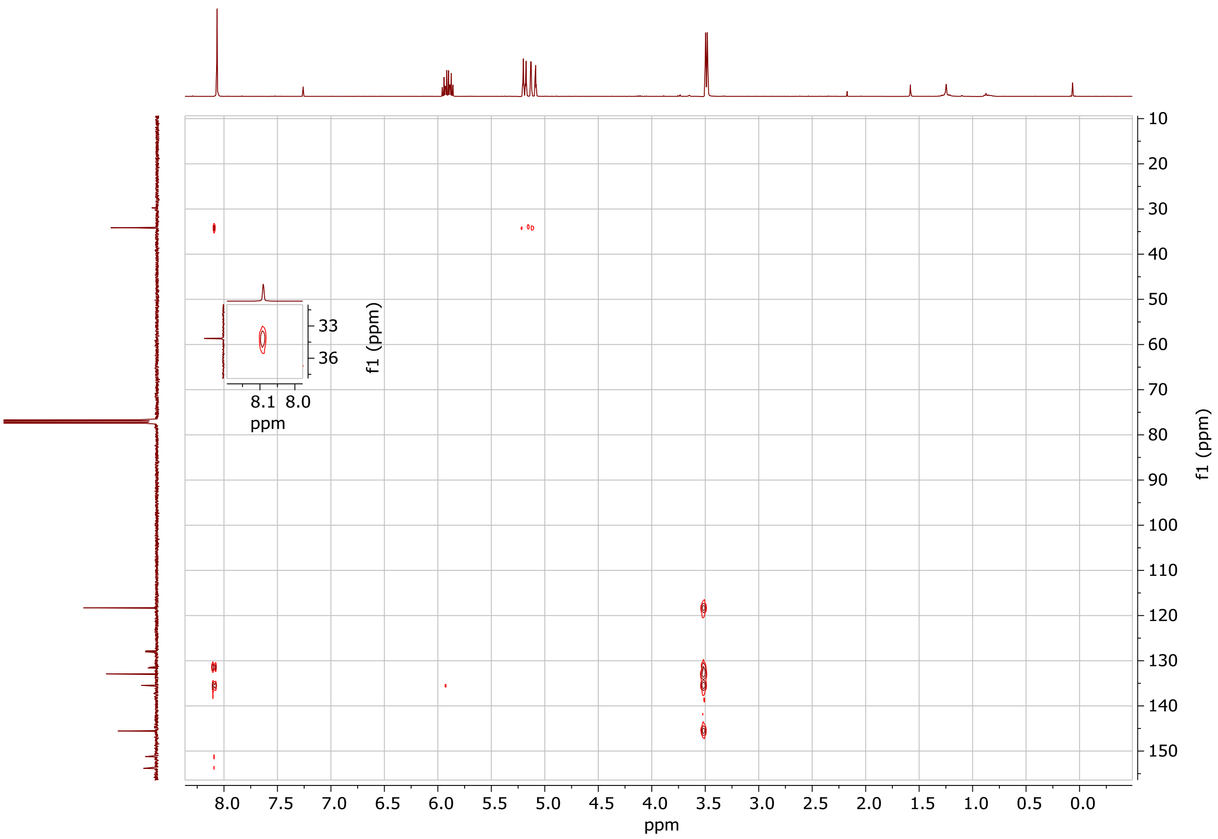

^{1}H, ^{13}C and HMBC NMR spectra of compound **13f**

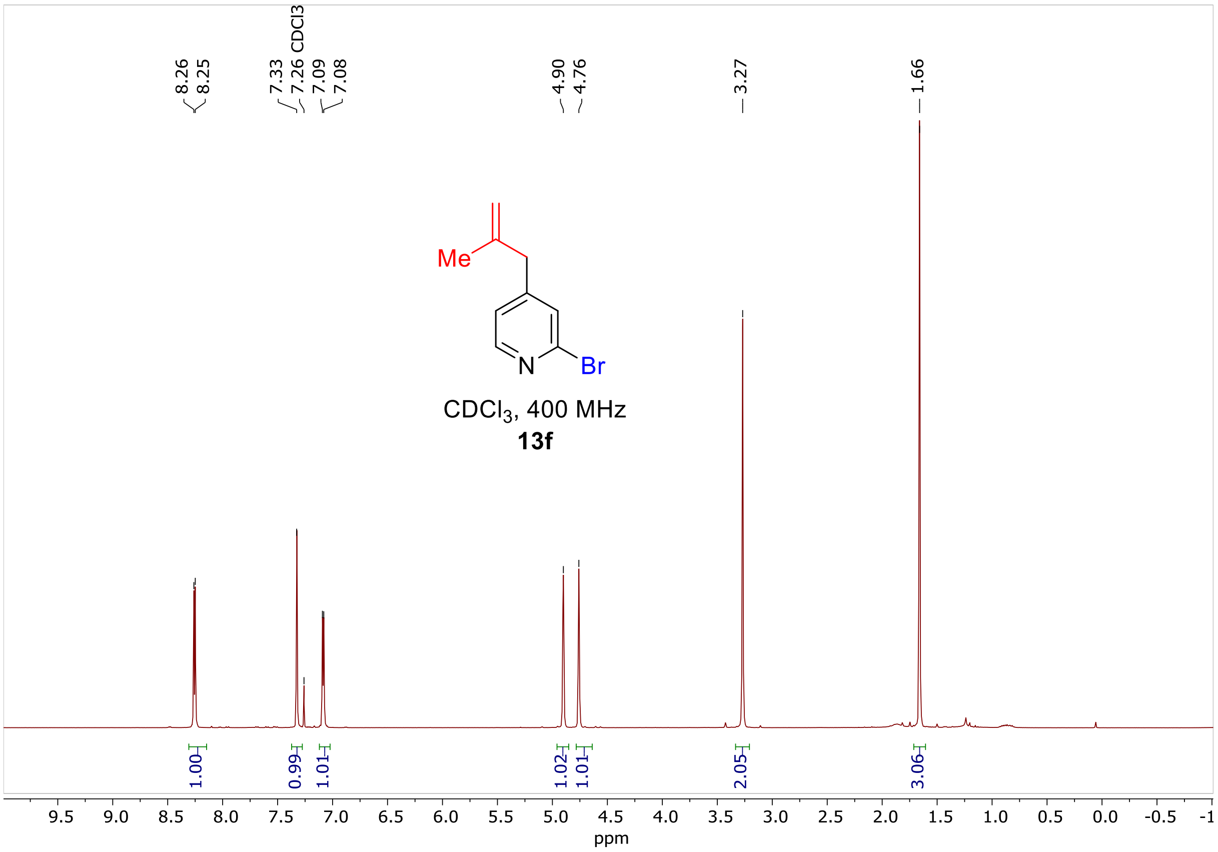

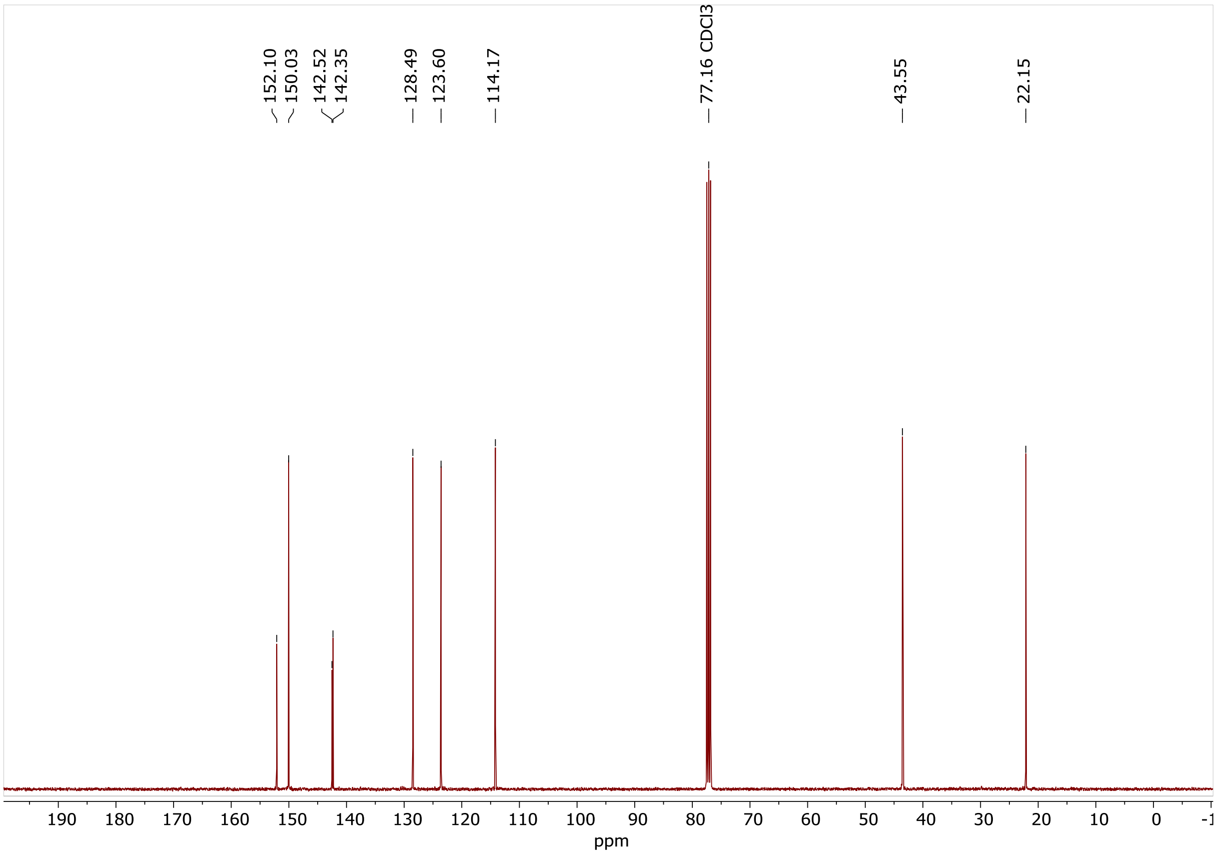
152.10
150.03
142.52
142.35
128.49
123.60
114.17
77.16 CDCl3
43.55
22.15
ppm

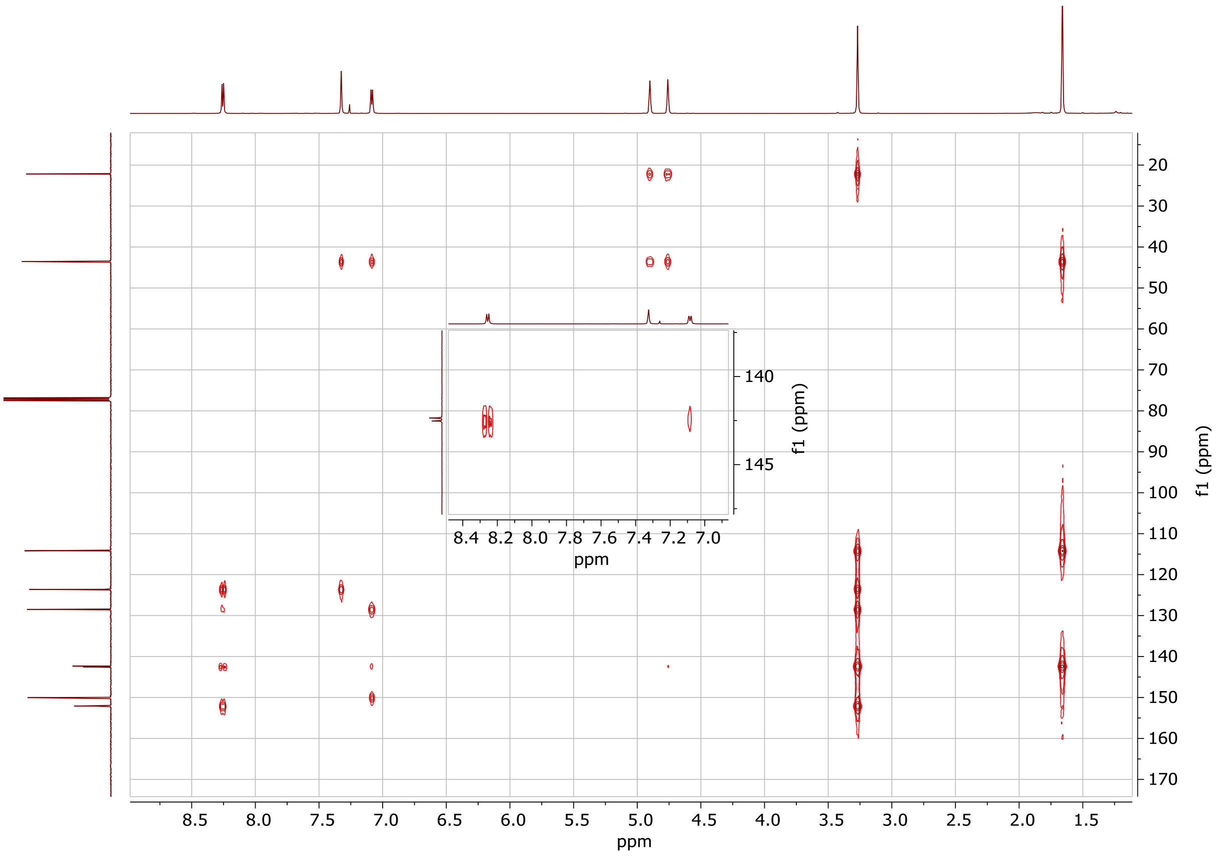
ppm
f1 (ppm)

^{1}H, ^{13}C and HMBC NMR spectra of compound **13g**

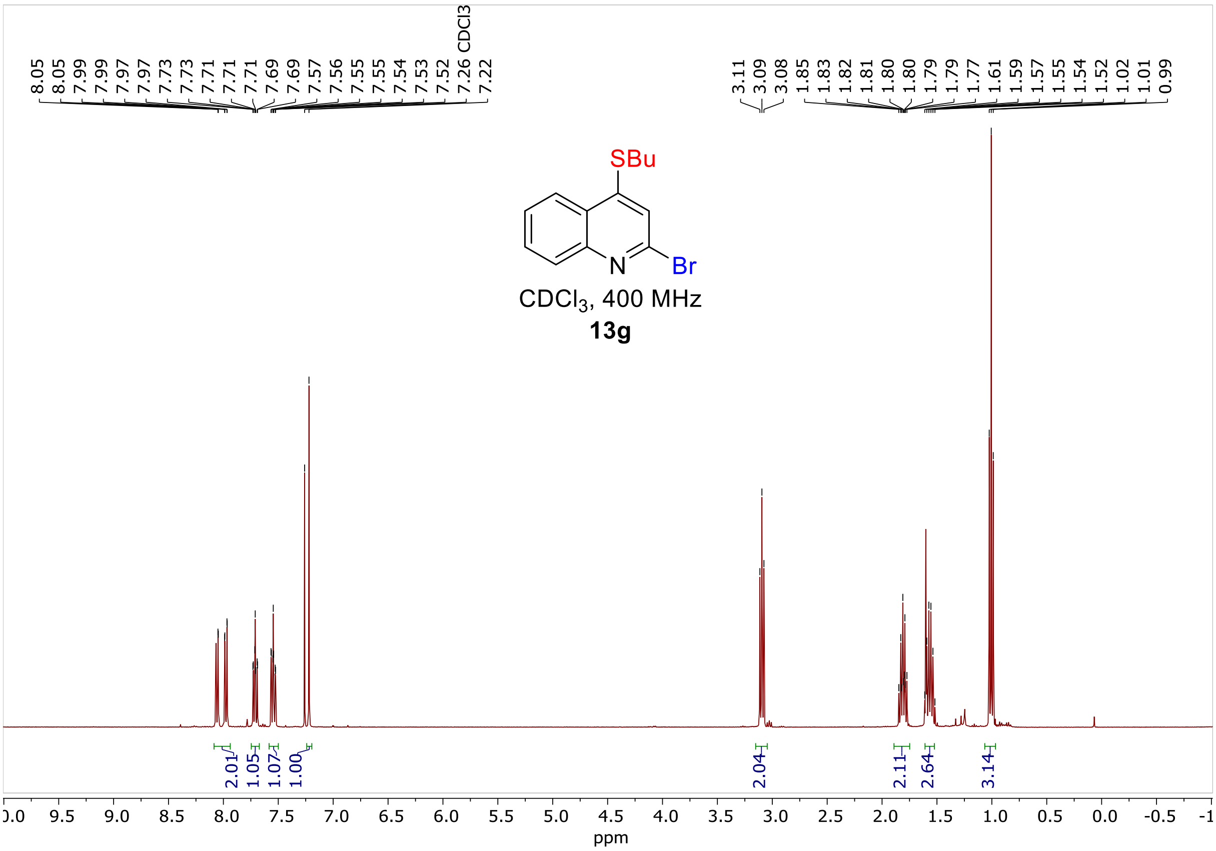

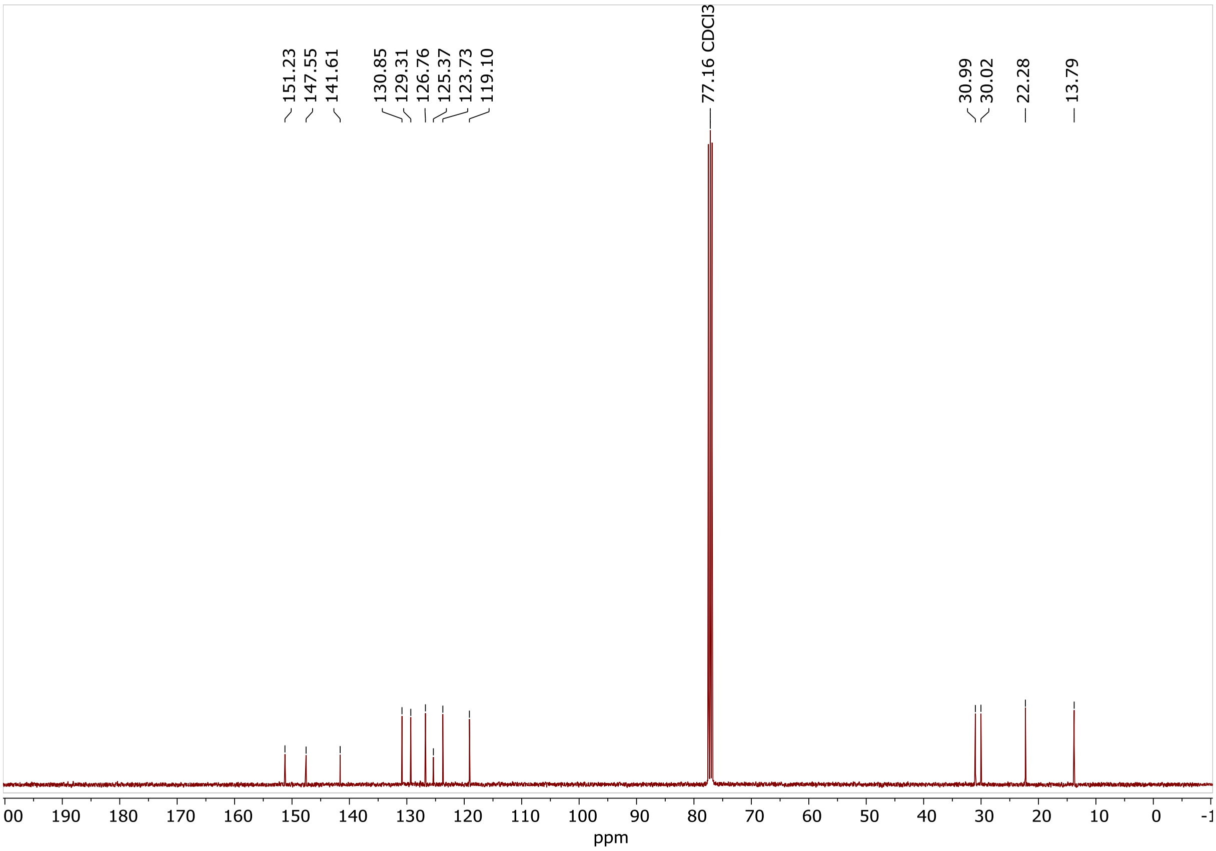

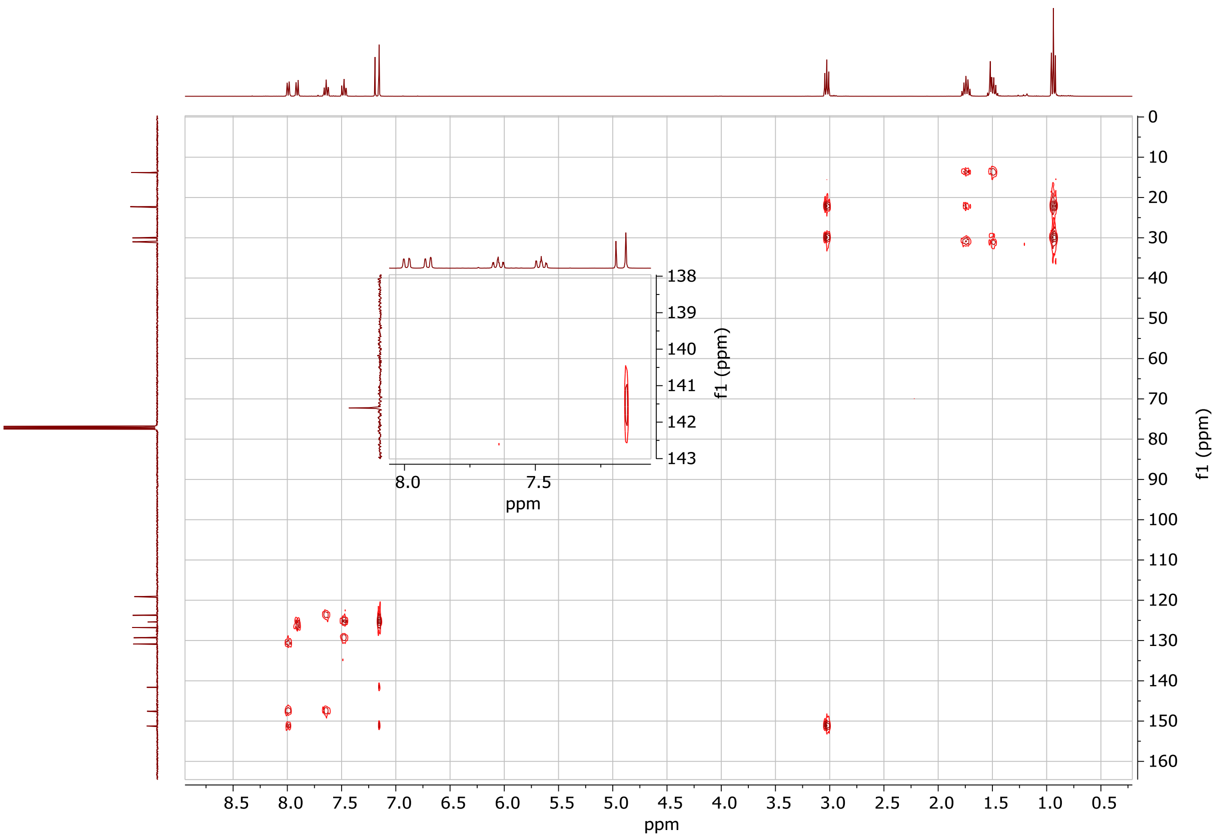

^{1}H, ^{13}C and HMBC NMR spectra of compound **14a**

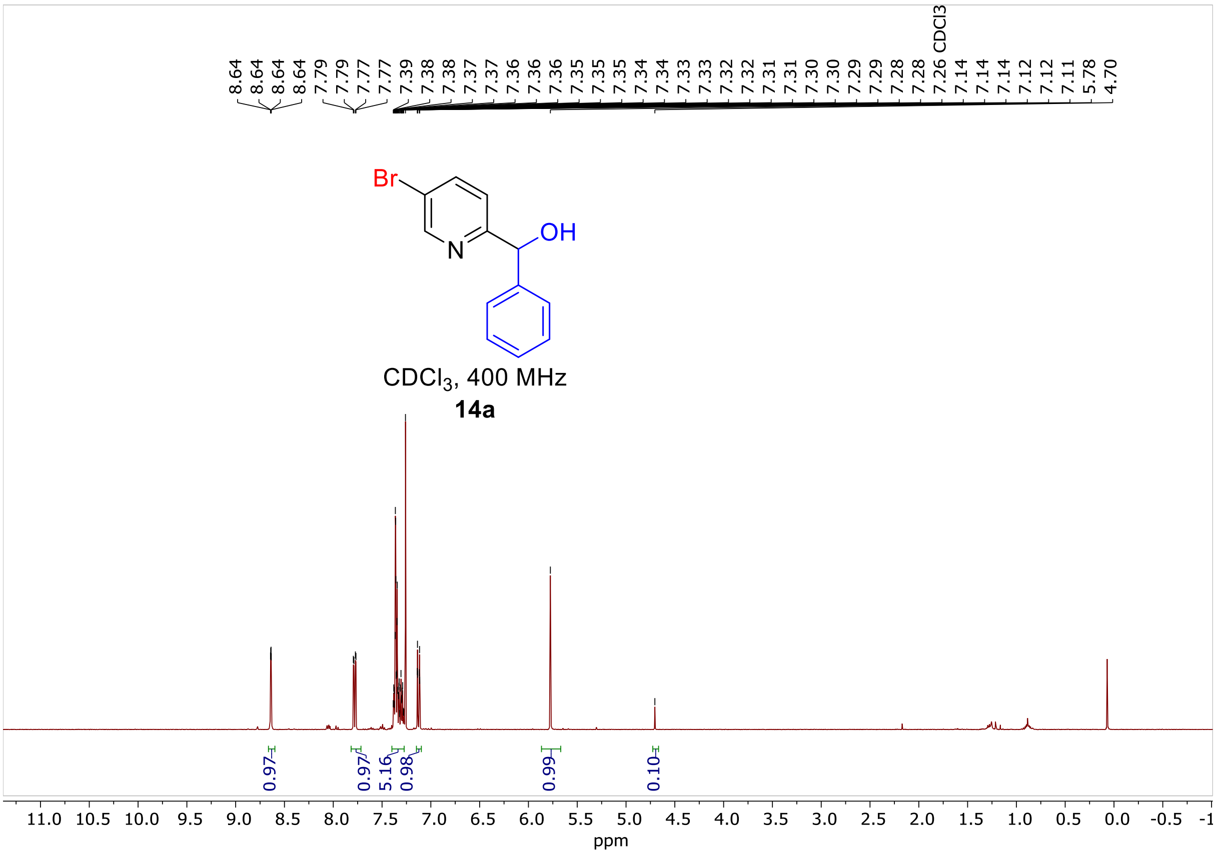

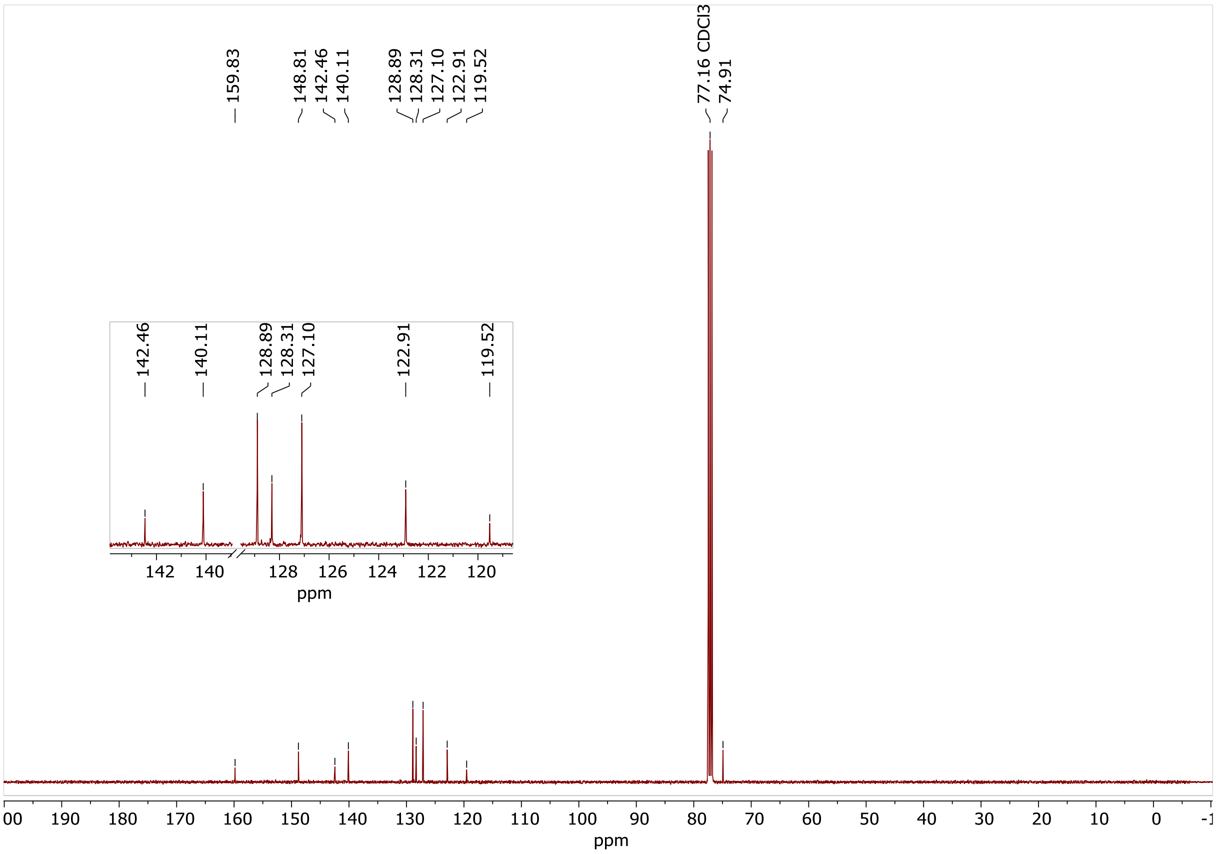
159.83
148.81
142.46
140.11
128.89
128.31
127.10
122.91
119.52
77.16 CDCl3
74.91
ppm

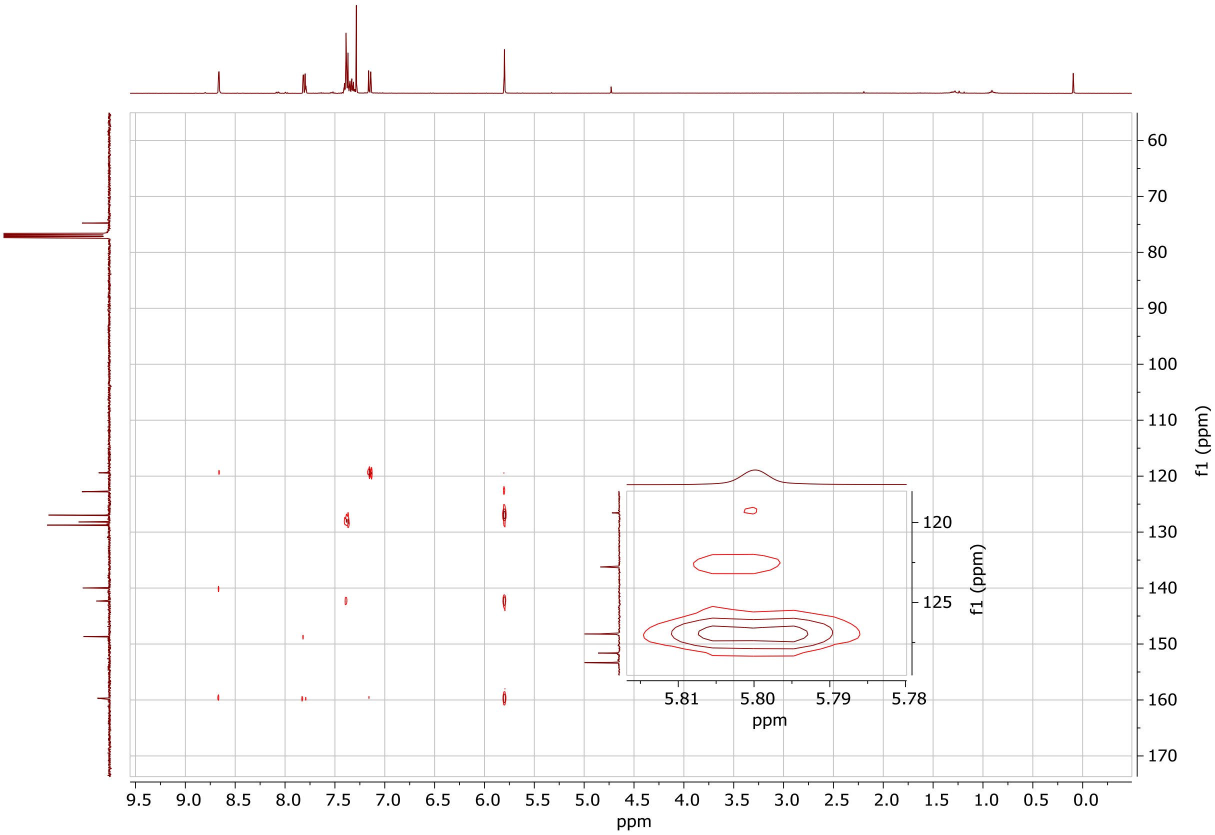
ppm
f1 (ppm)

^{1}H, ^{13}C and NOESY NMR spectra of compound **14b**

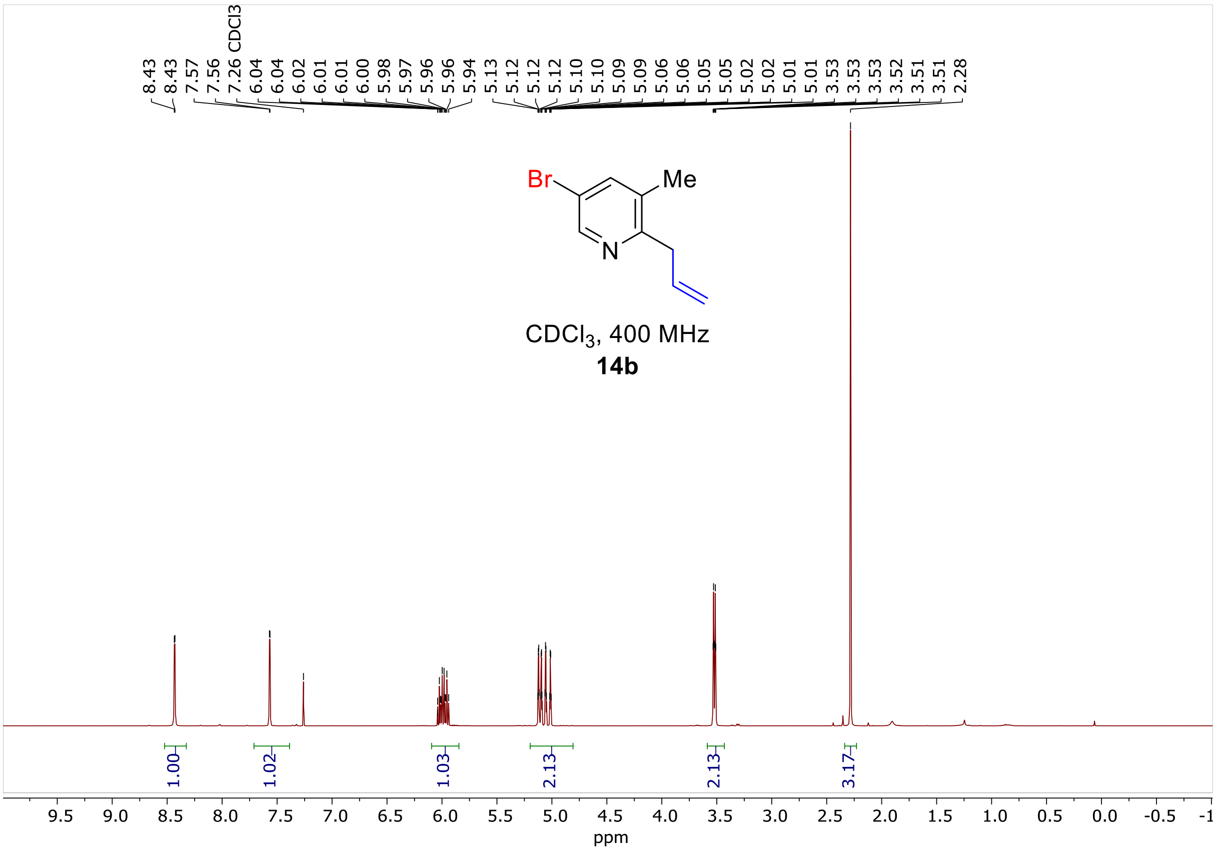

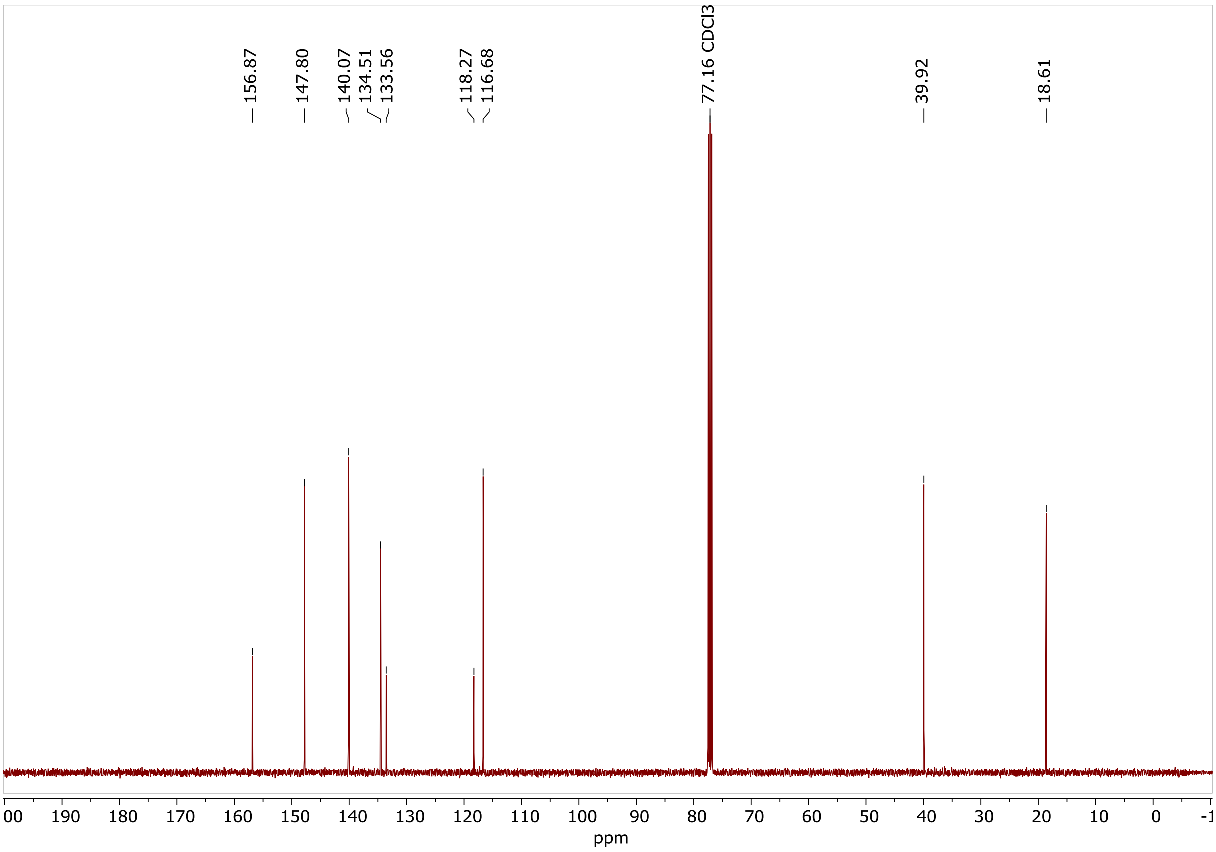

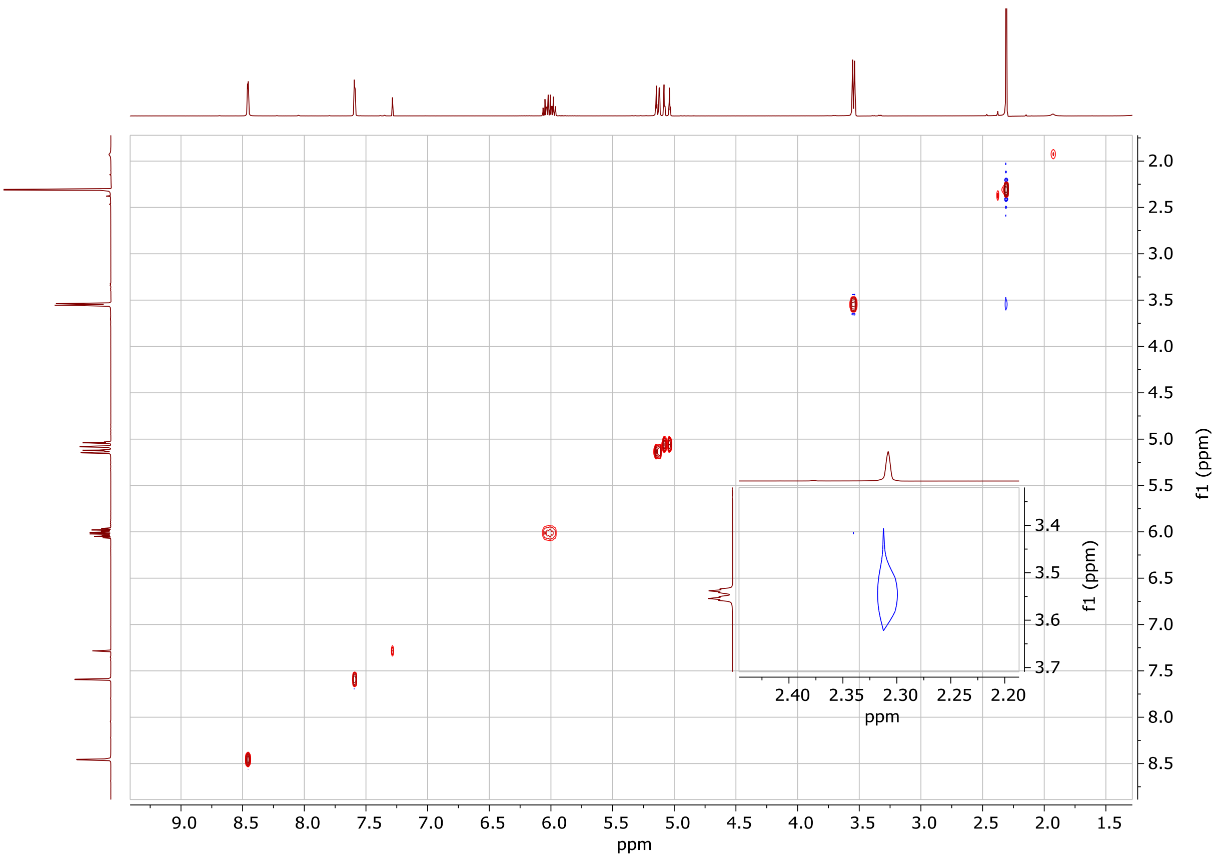

^{1}H, ^{13}C and HMBC NMR spectra of compound **14c**

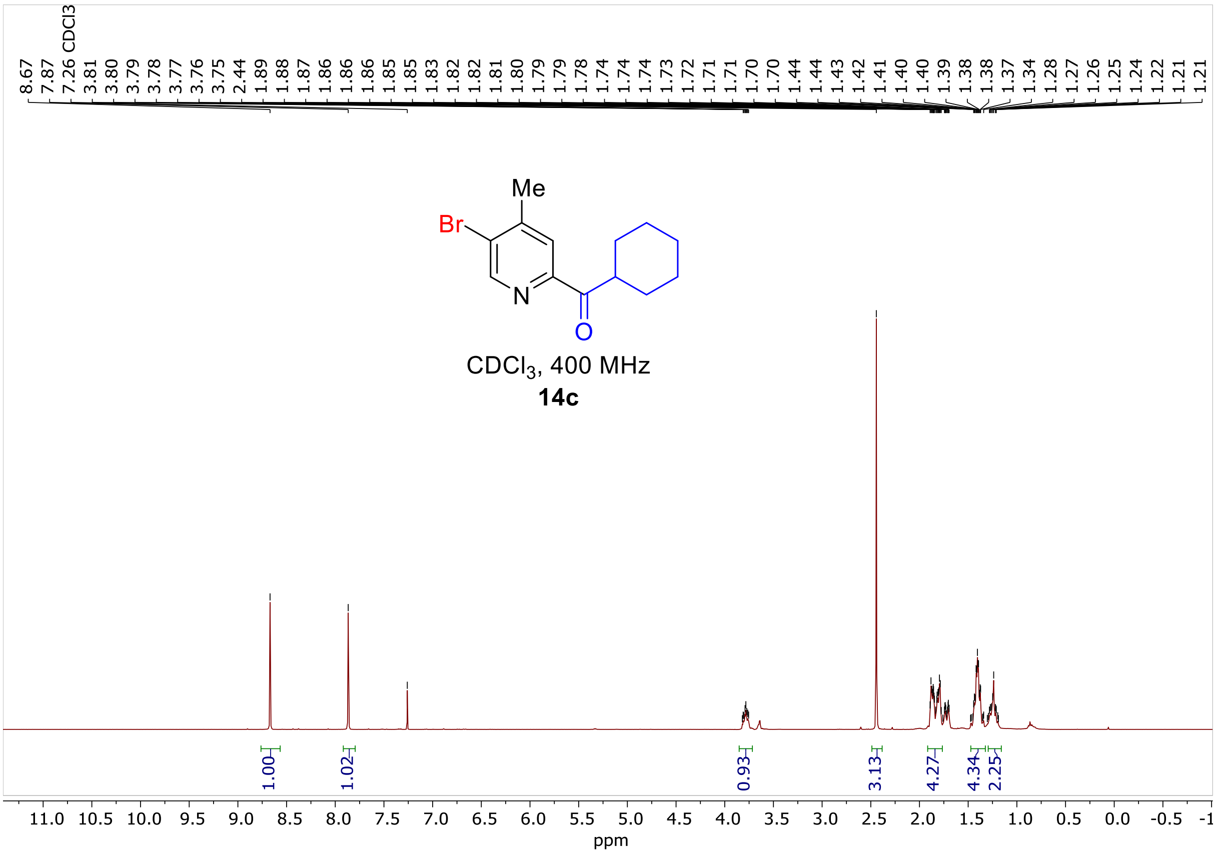

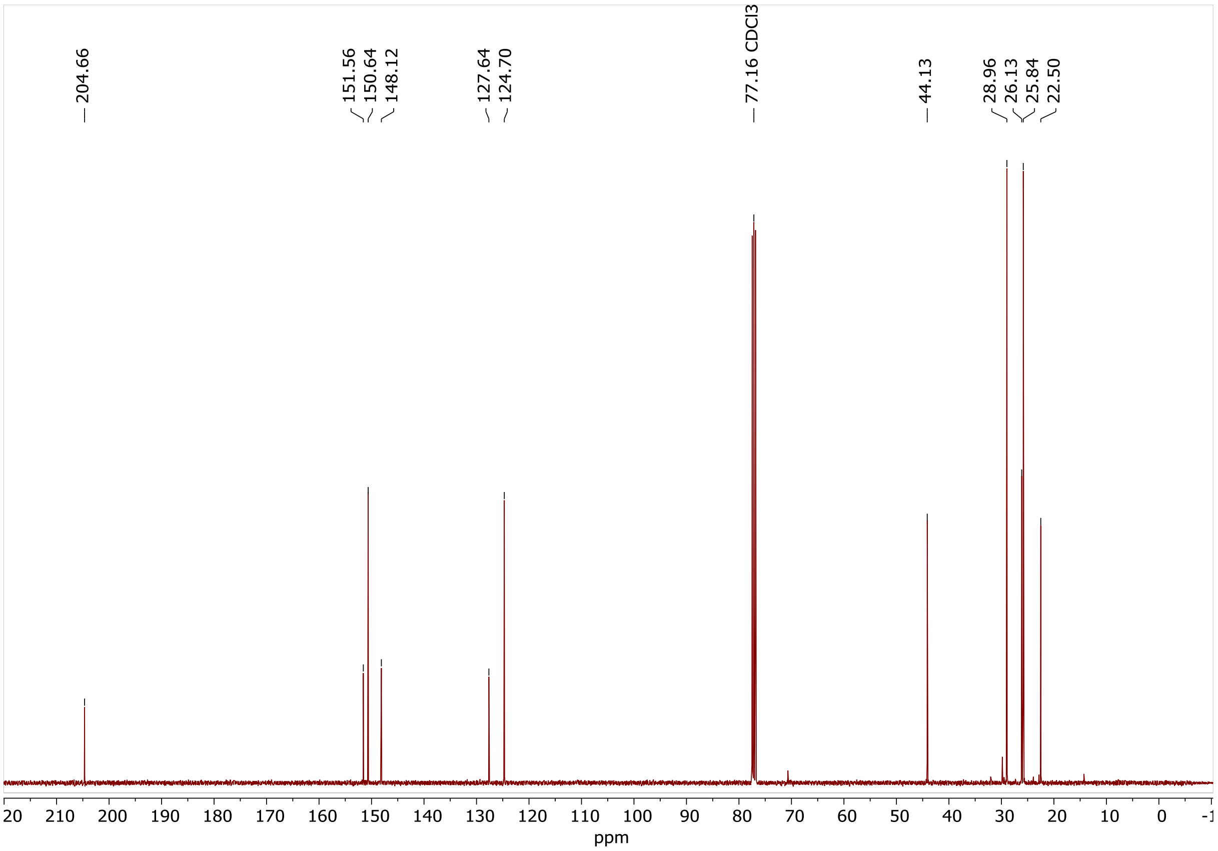
204.66
151.56
150.64
148.12
127.64
124.70
77.16 CDCl3
44.13
28.96
26.13
25.84
22.50
ppm

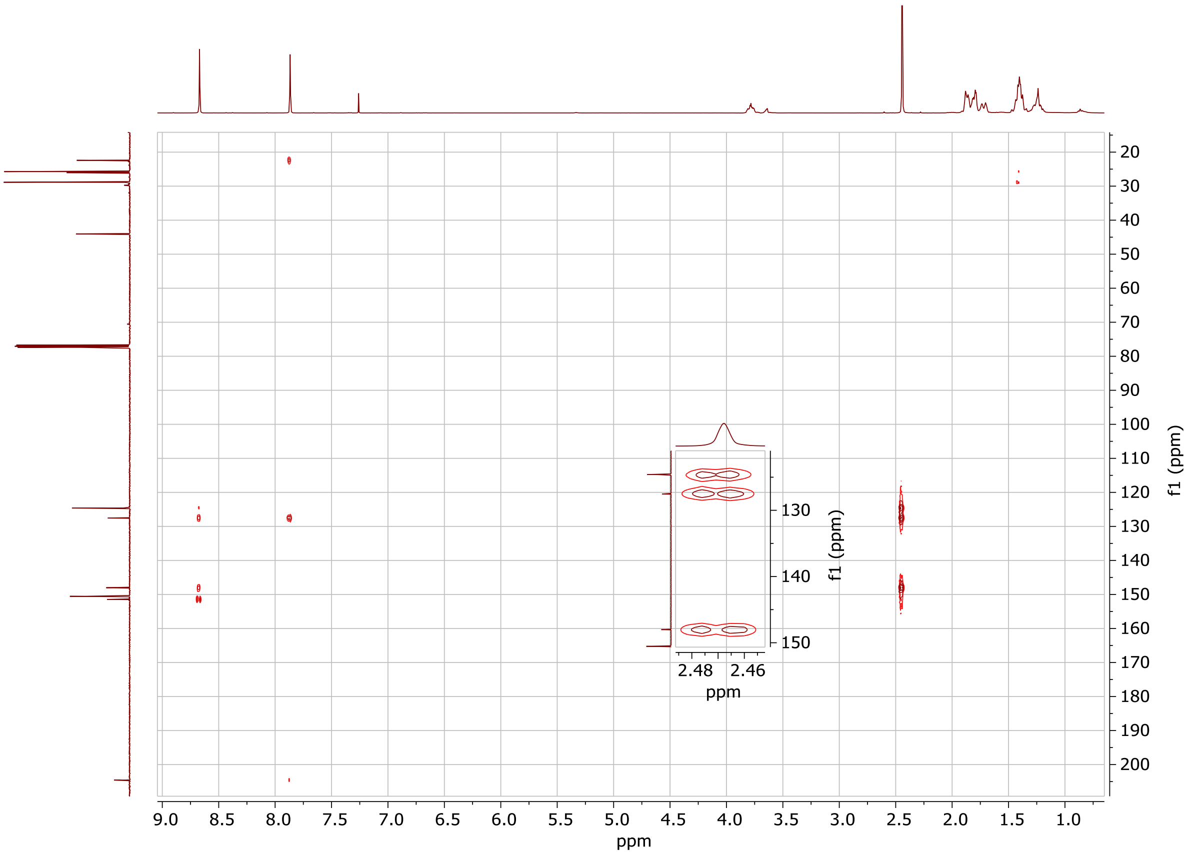
f1 (ppm)
ppm
2.48
2.46

^{1}H, ^{13}C and HMBC NMR spectra of compound **14d**

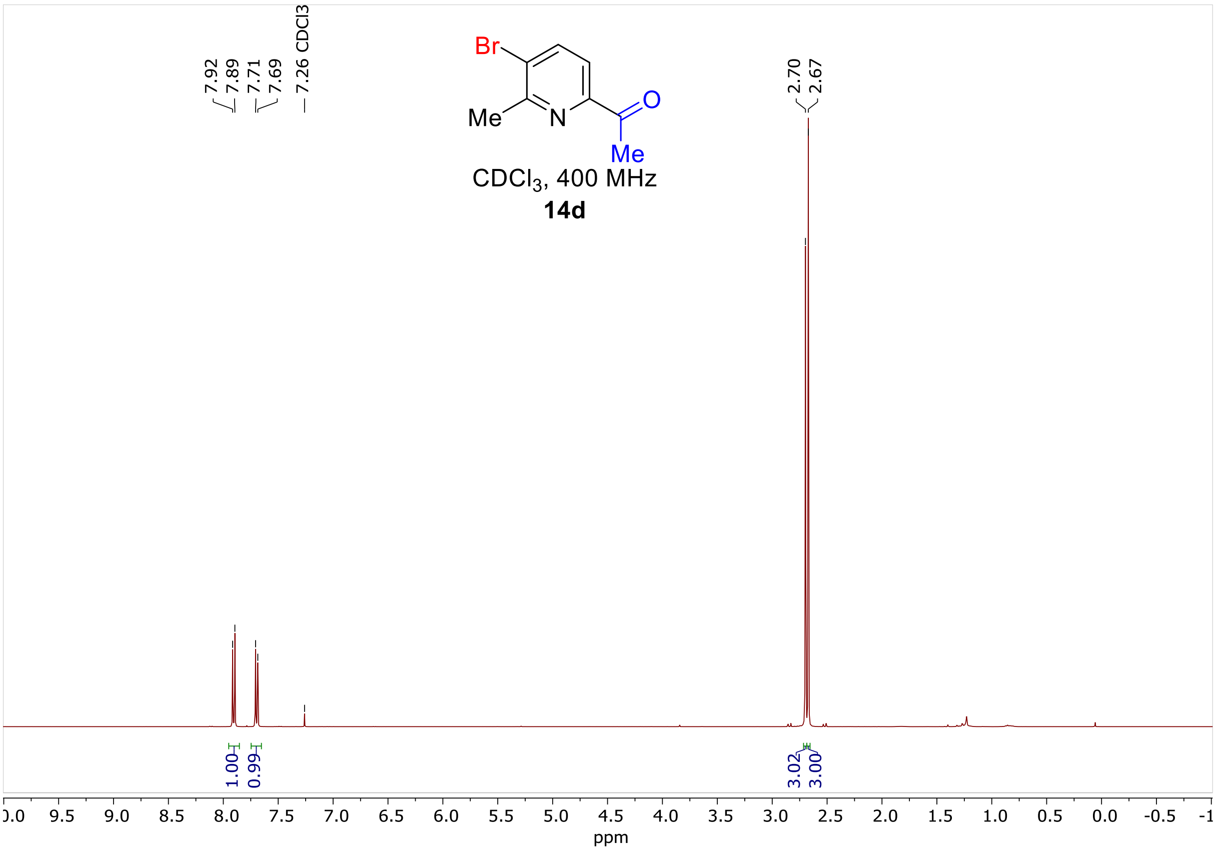

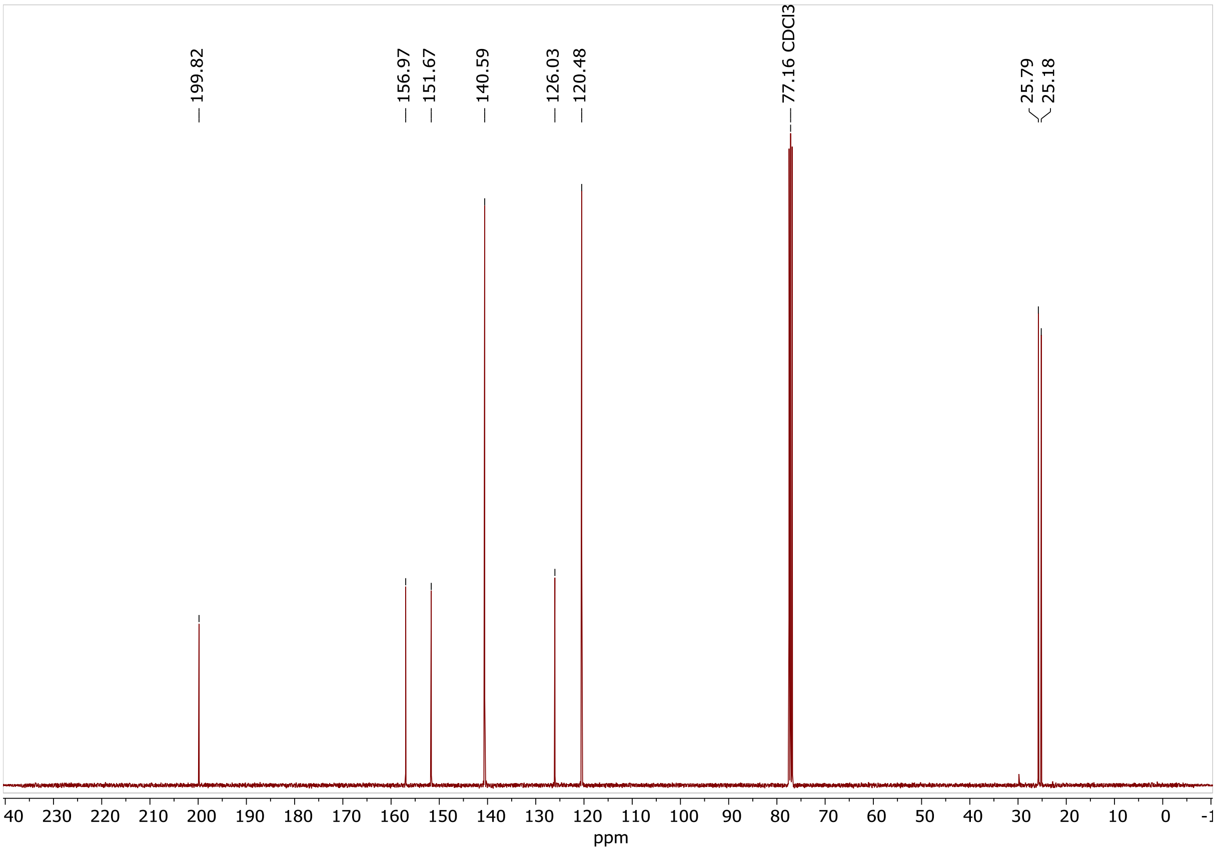

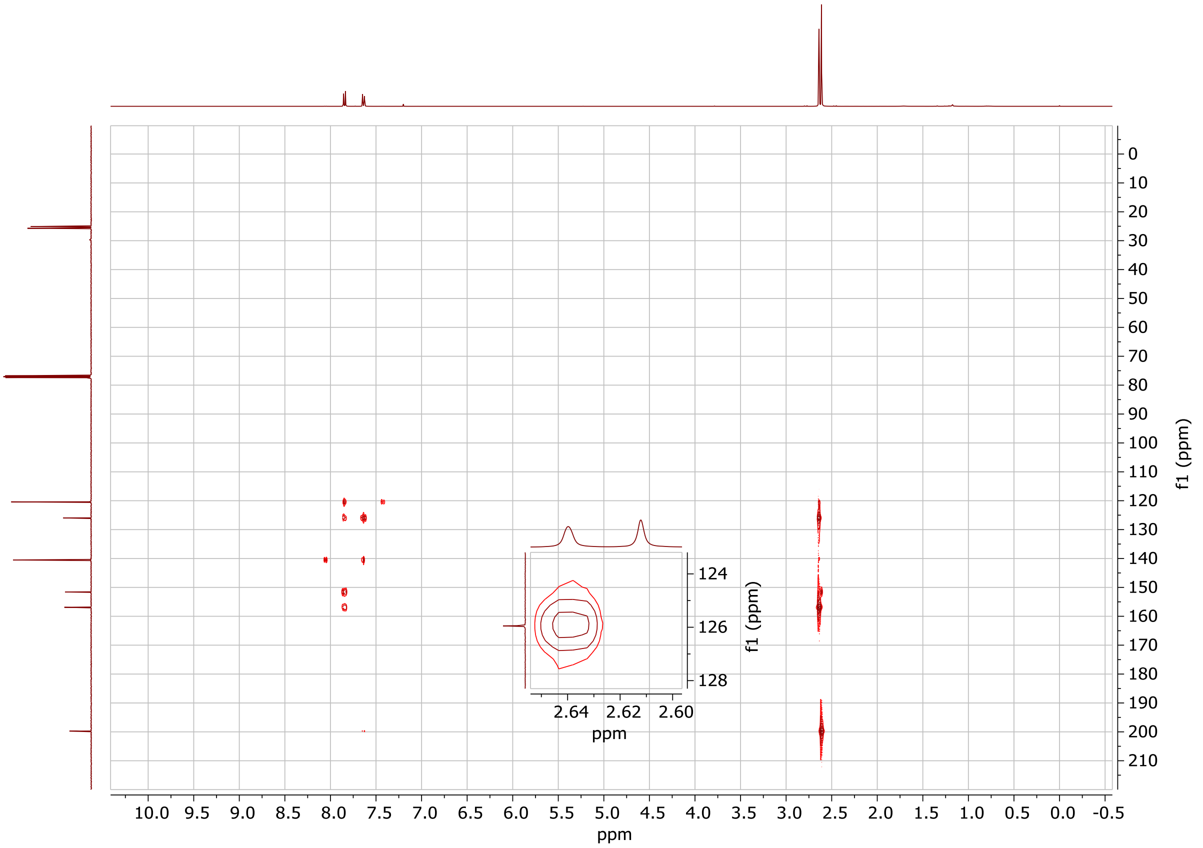

^1H, ^{13}C, ^{19}F and HMBC NMR spectra of compound **14e**

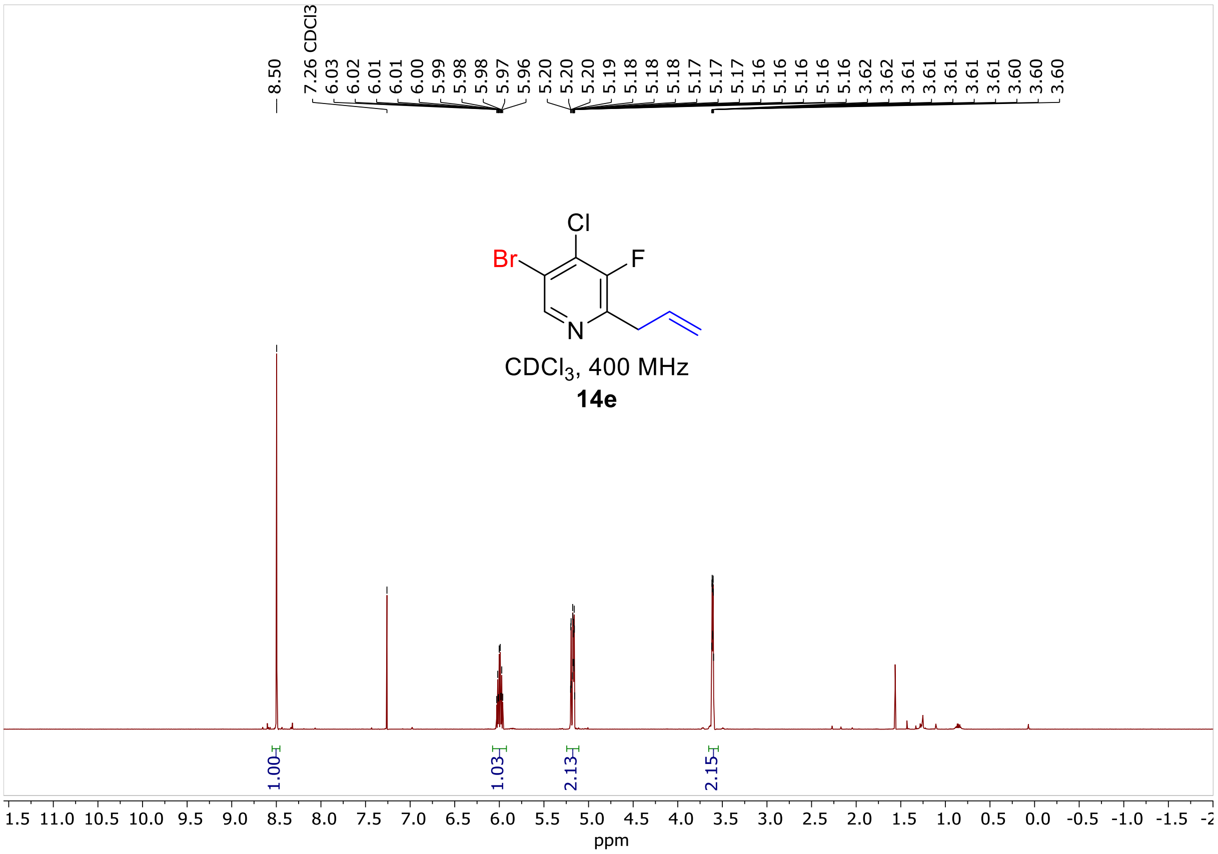

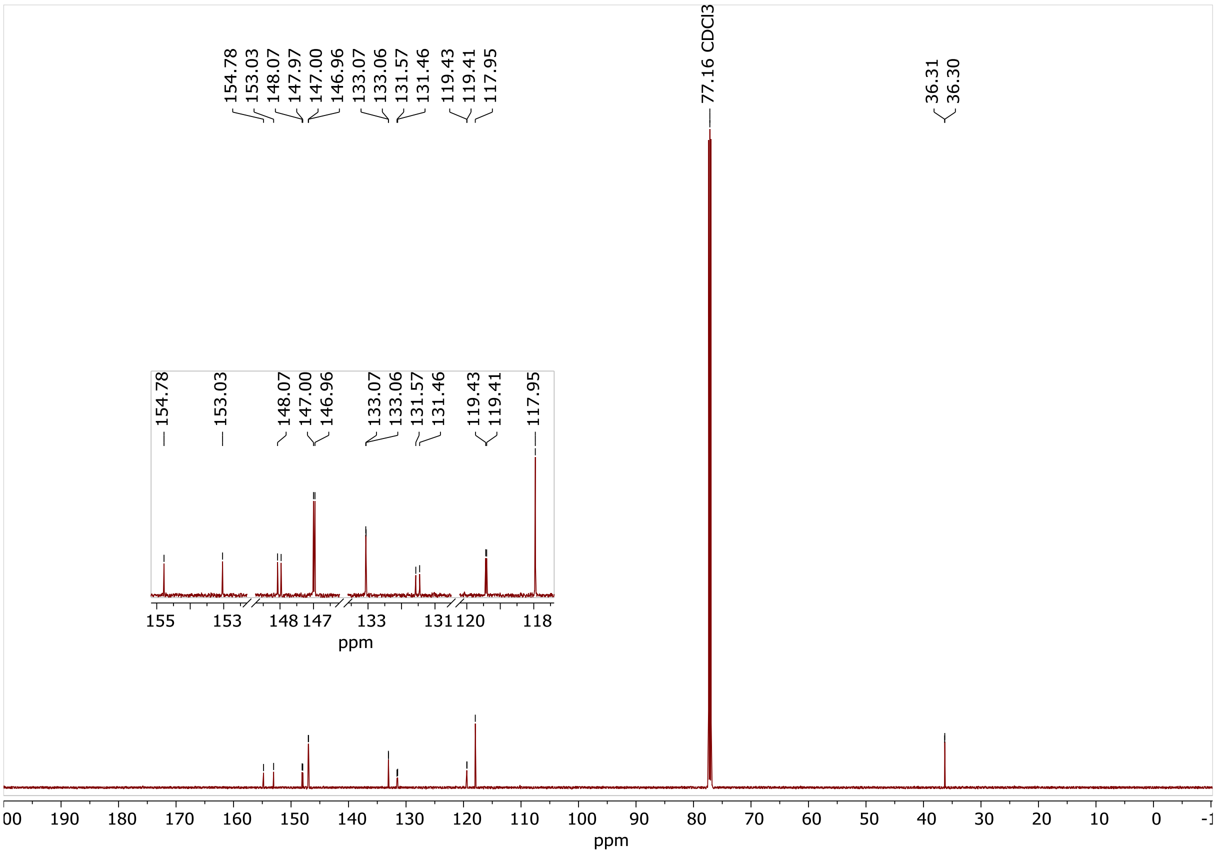

154.78
153.03
148.07
147.97
147.00
146.96
133.07
133.06
131.57
131.46
119.43
119.41
117.95
77.16 CDCl3
36.31
36.30
ppm

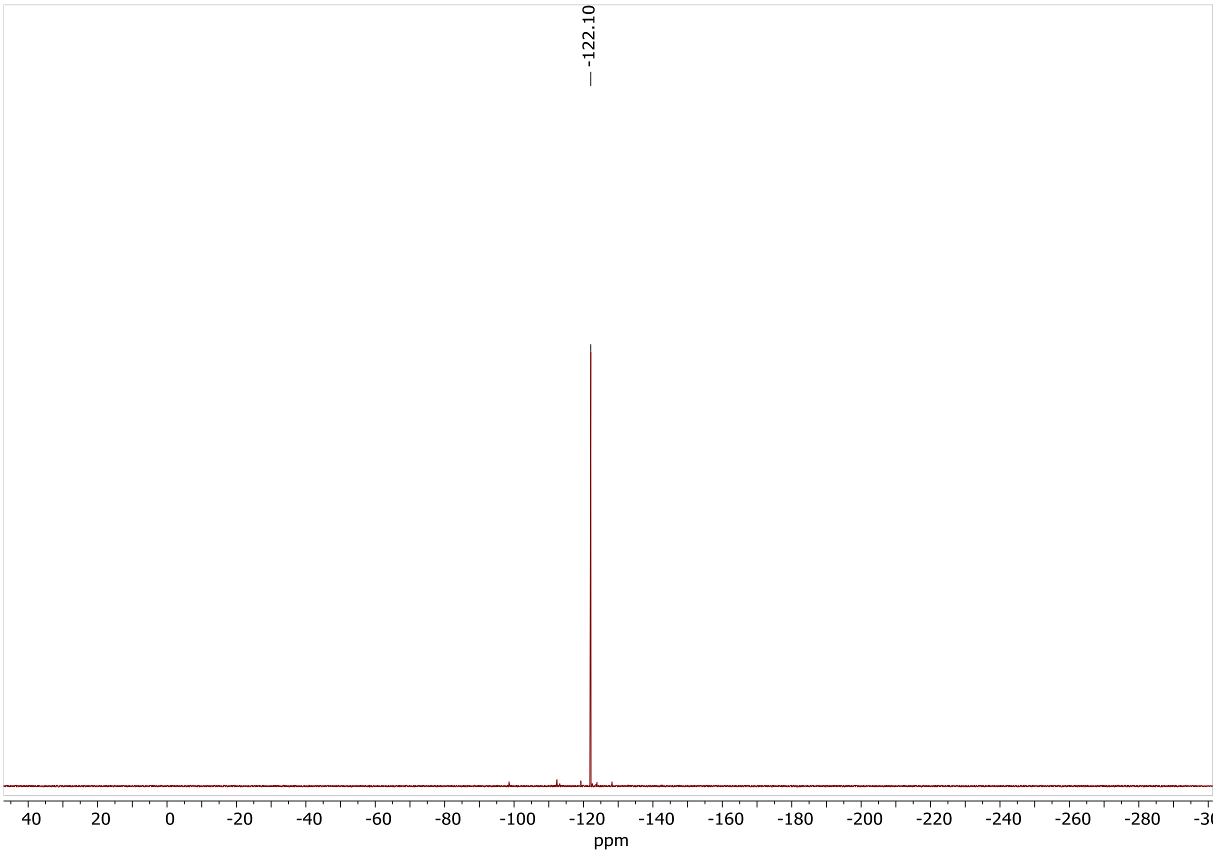

-122.10
ppm

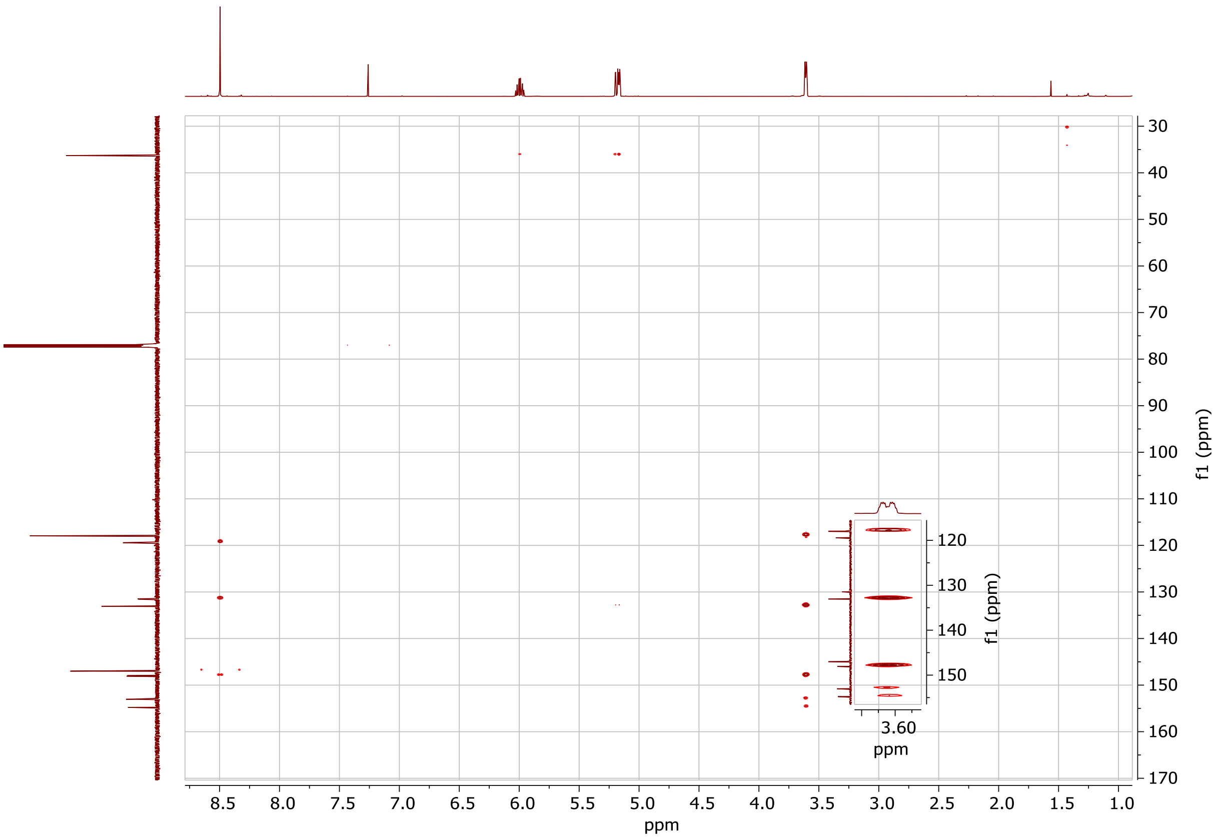

^{1}H, ^{13}C and NOESY NMR spectra of compound **14f**

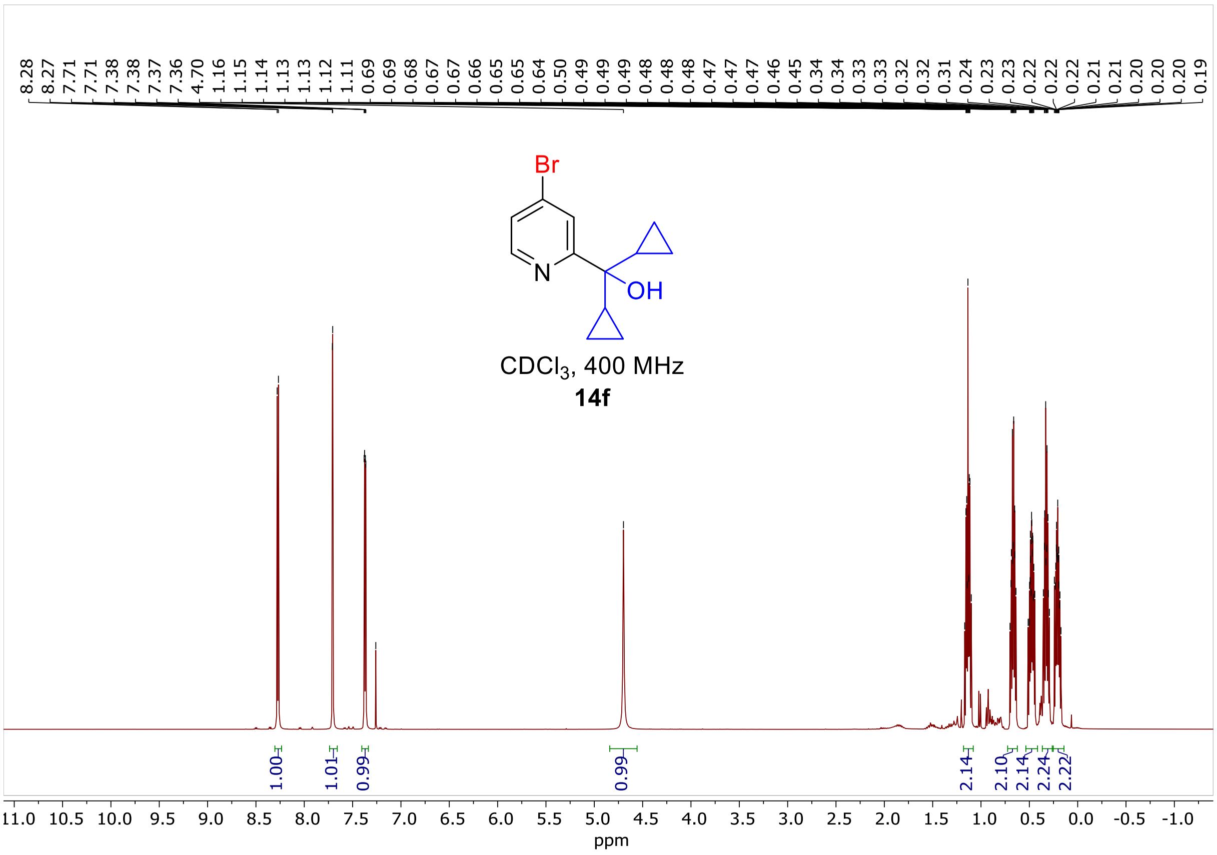

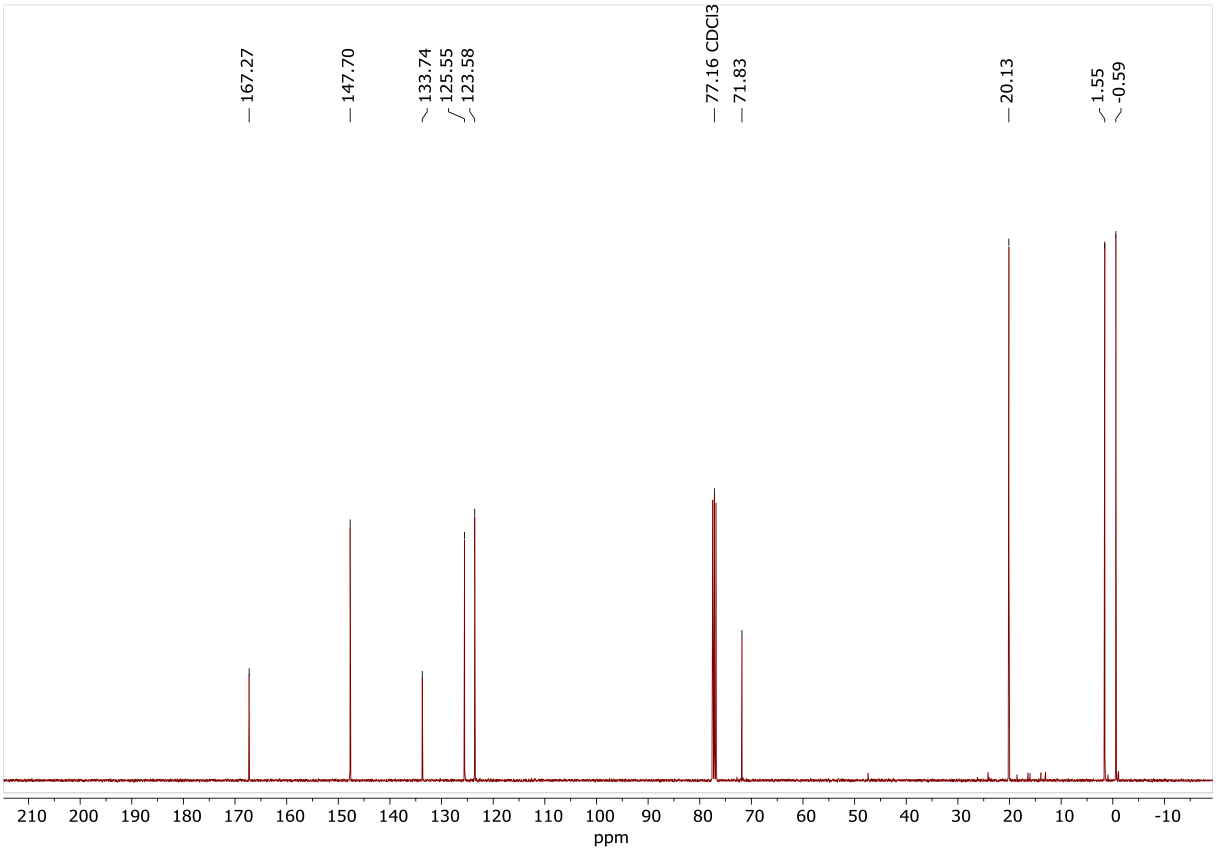

167.27
147.70
133.74
125.55
123.58
77.16 CDCl3
71.83
20.13
1.55
-0.59
ppm

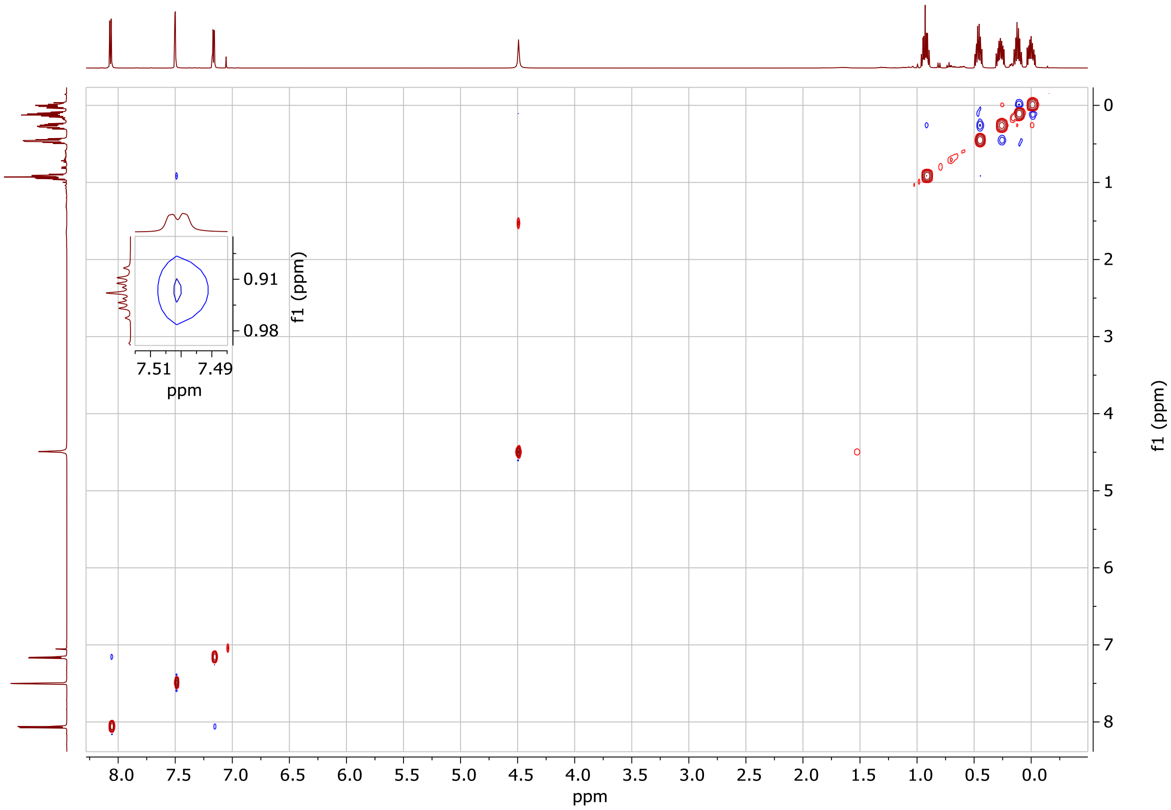

0.91
0.98
f1 (ppm)
7.51
7.49
ppm
f1 (ppm)
ppm

^{1}H, ^{13}C and HMBC NMR spectra of compound **14g**

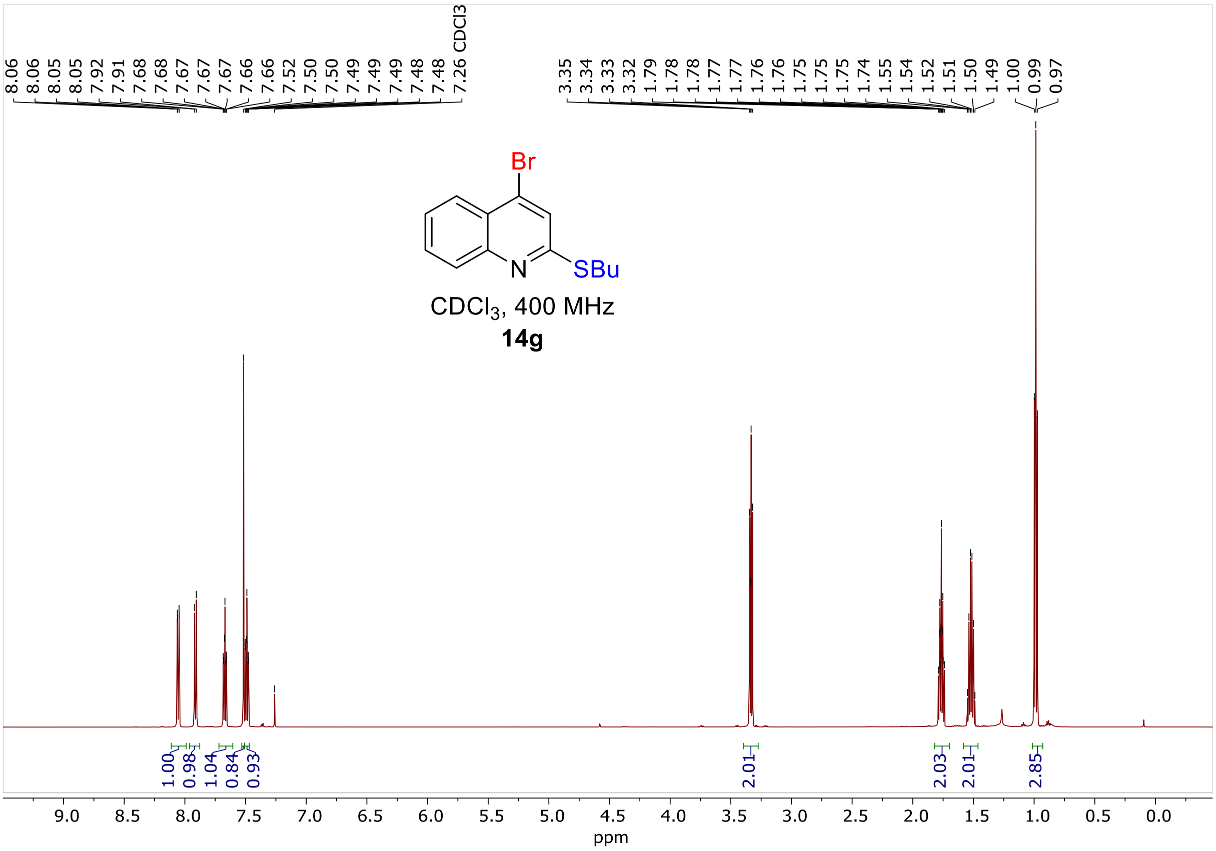

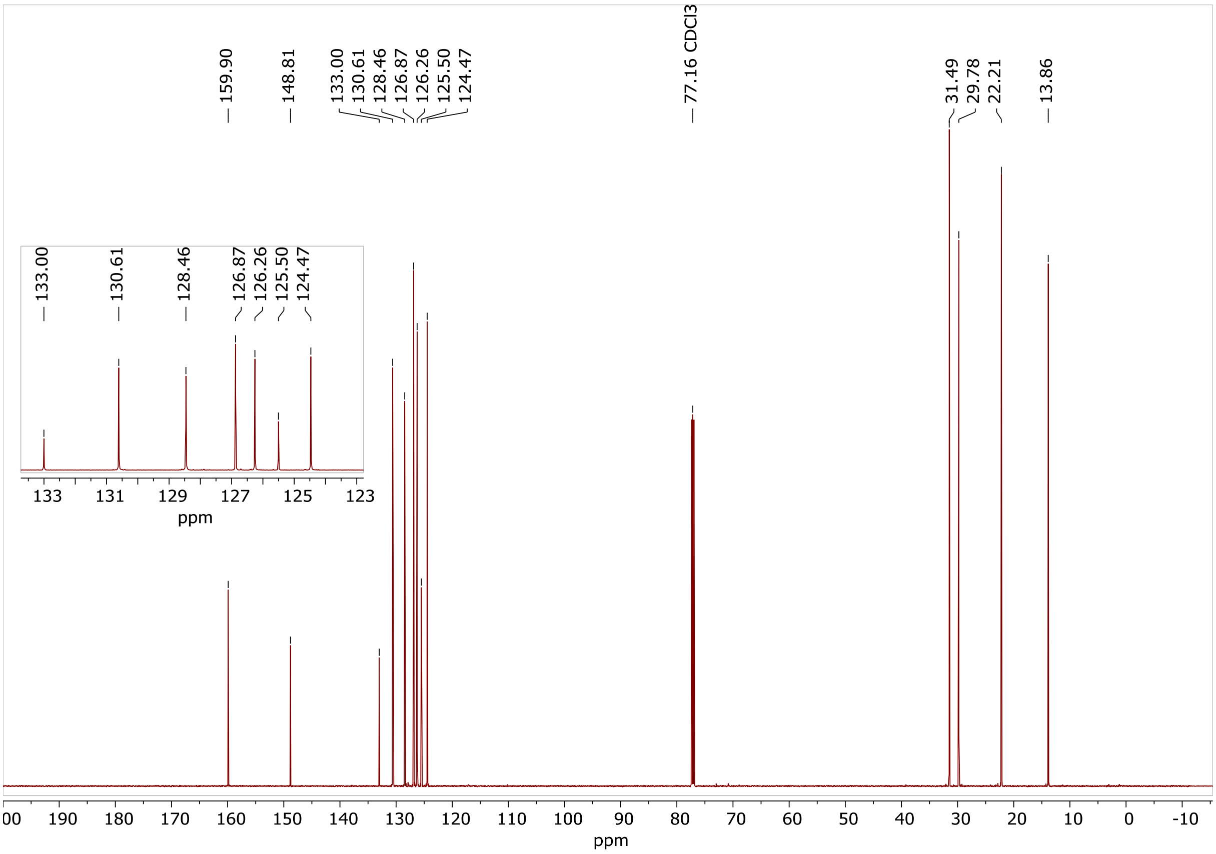

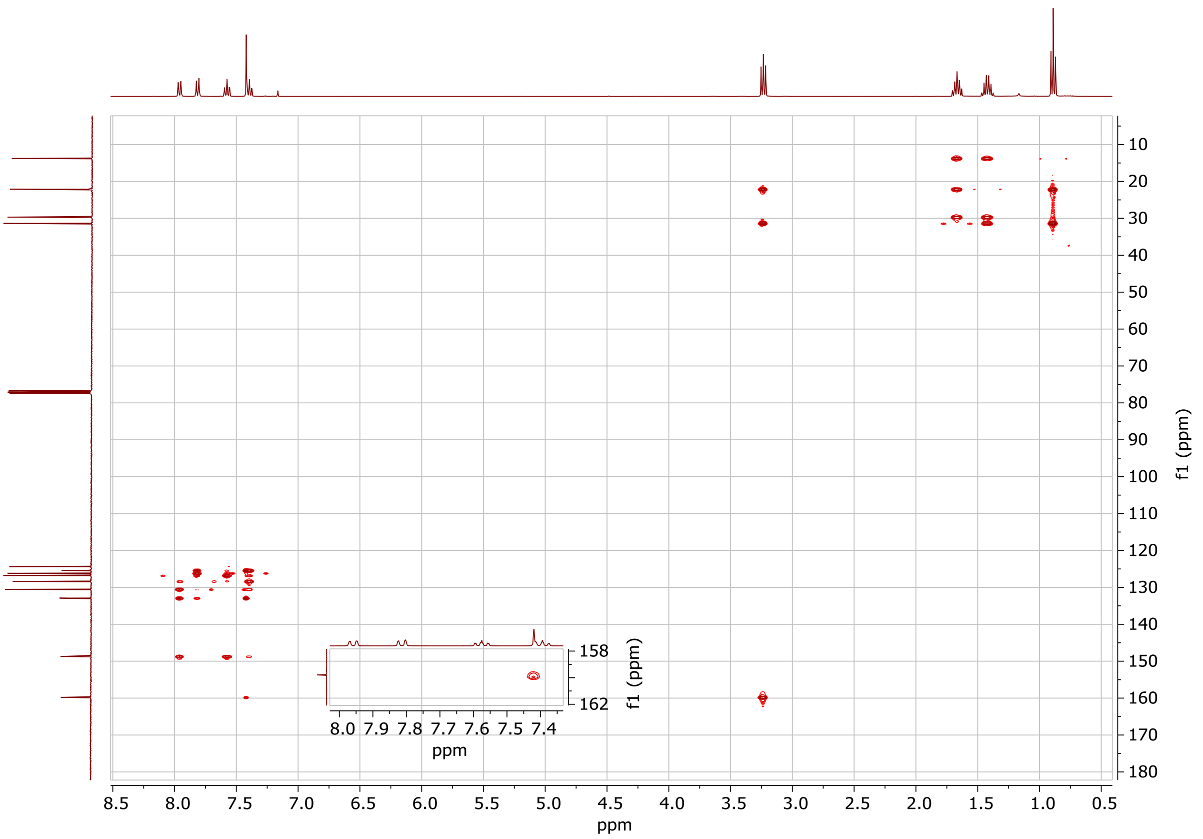

^1H and ^{13}C NMR spectra of compound **16**

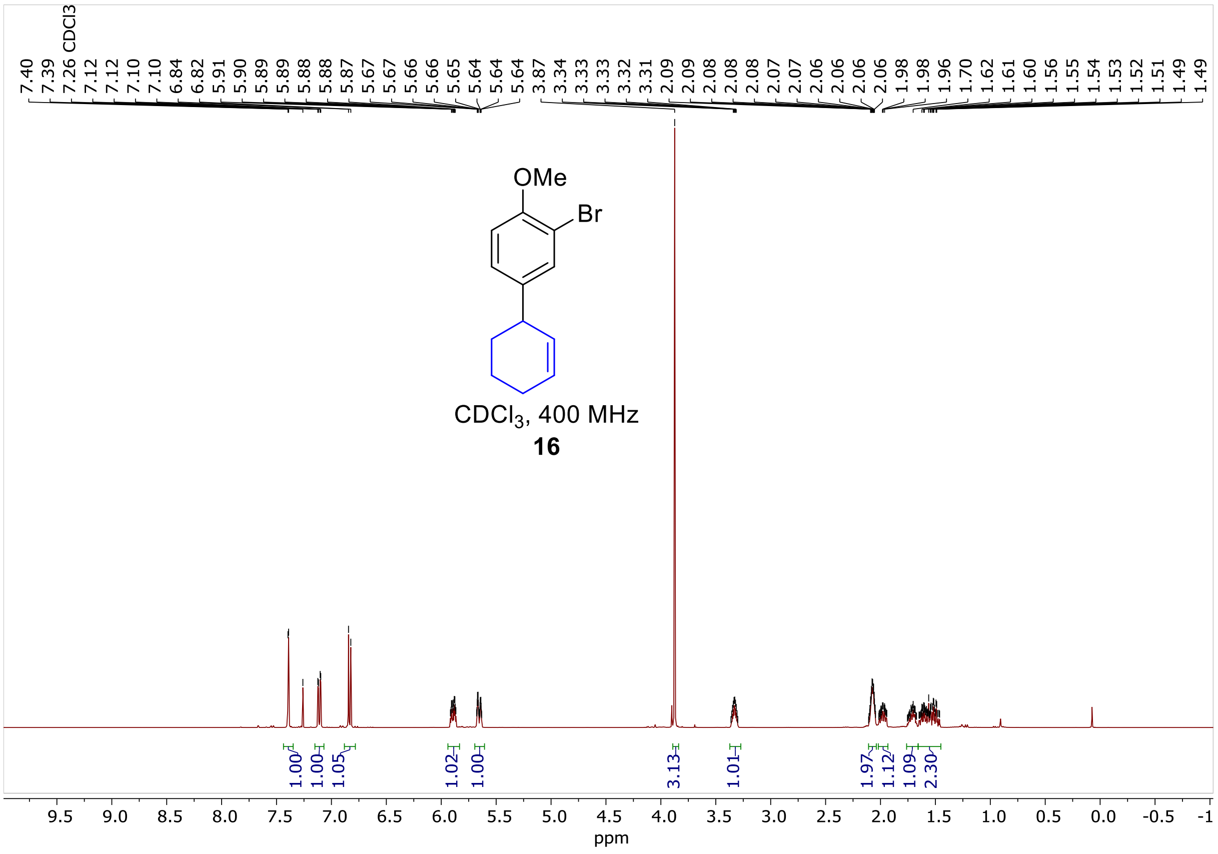

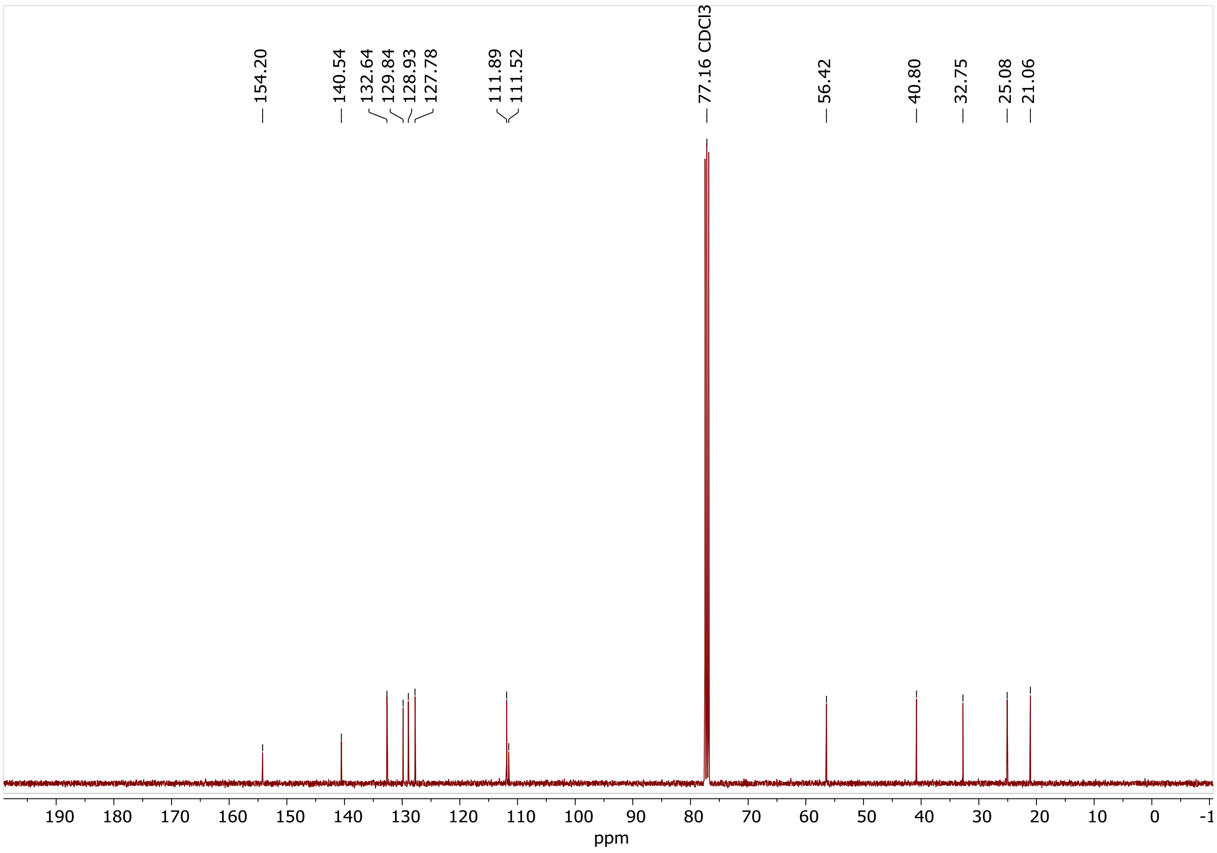

^{1}H and ^{13}C NMR spectra of compound **17**

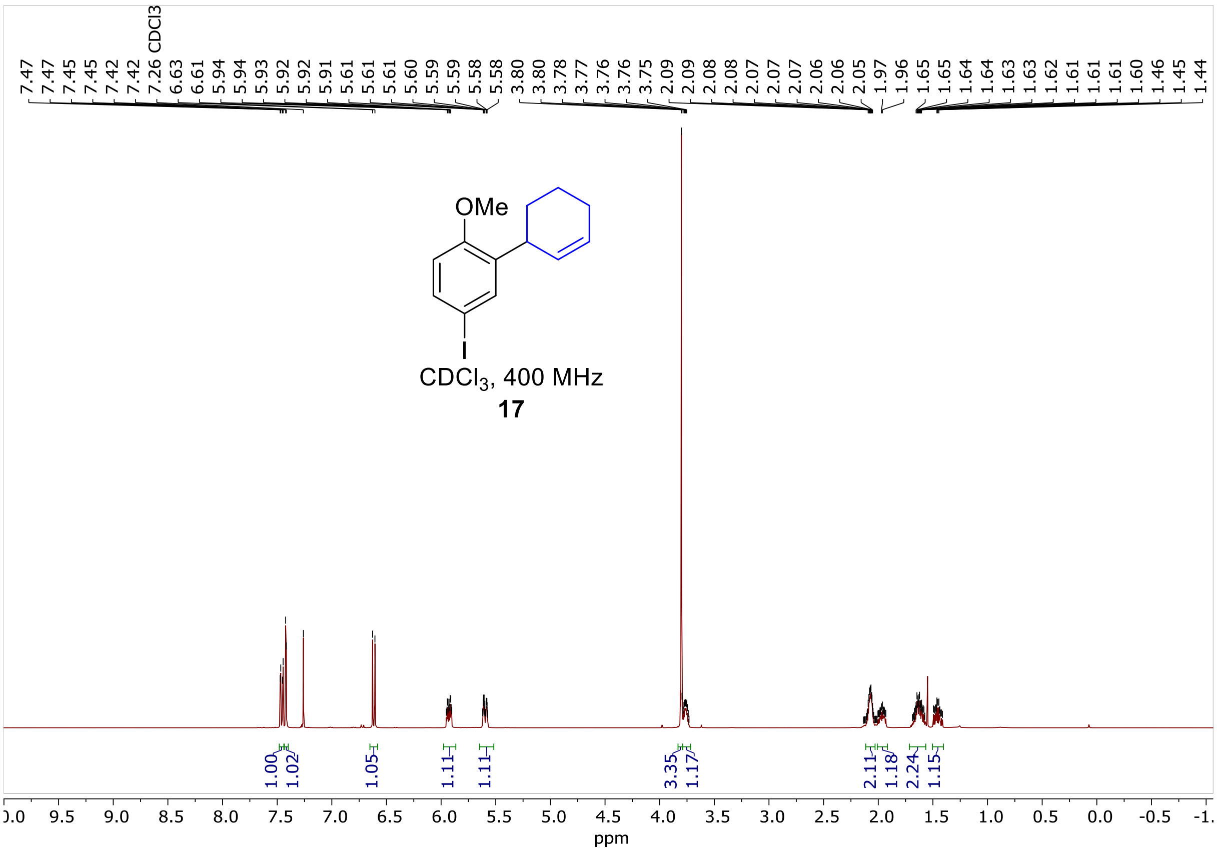

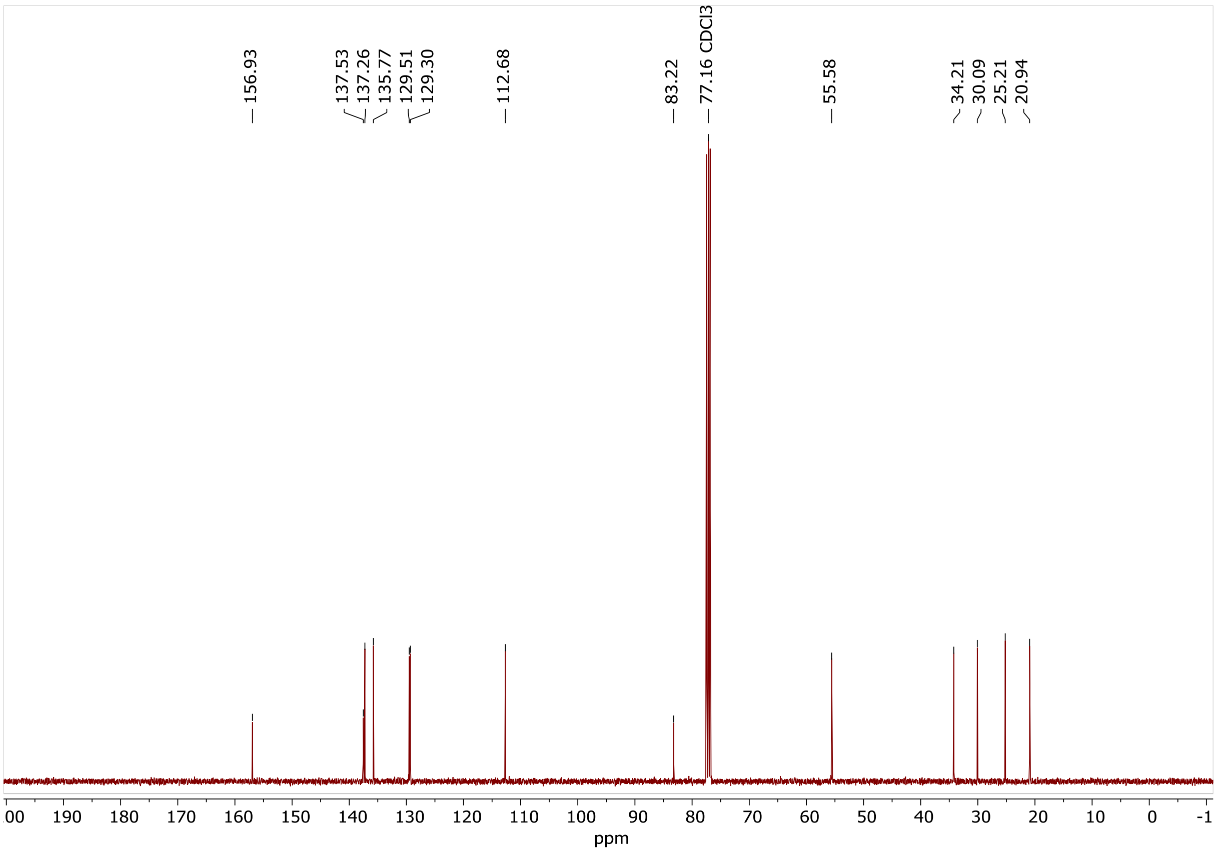

^{1}H and ^{13}C NMR spectra of compound **SM1**

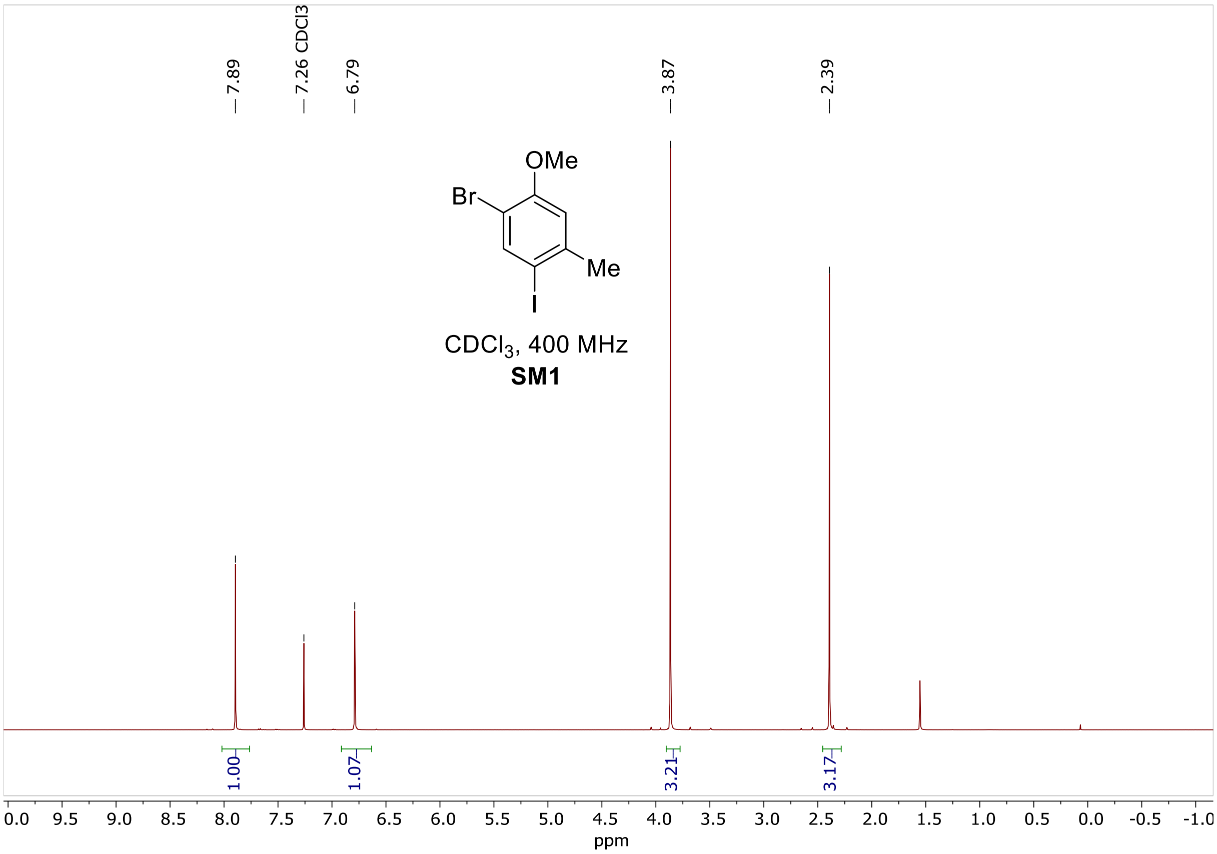

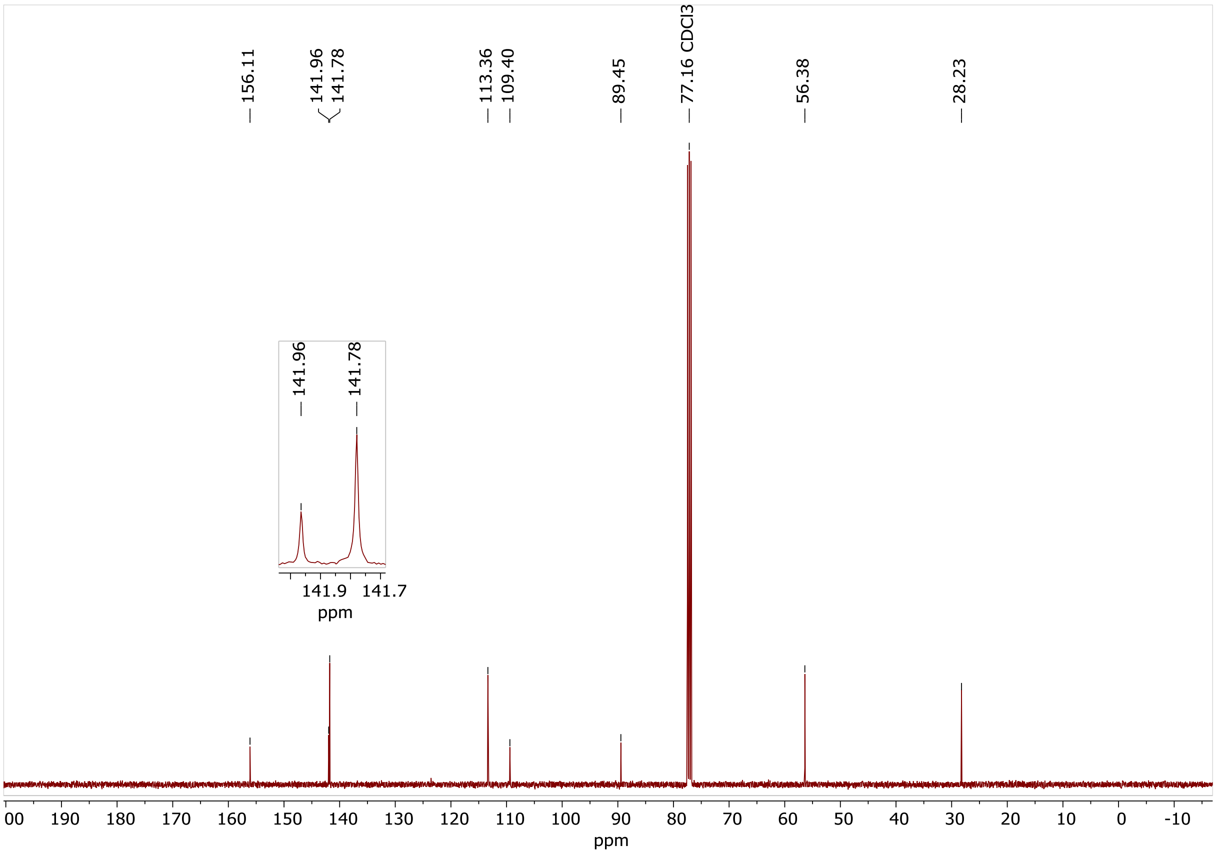

^{1}H and ^{13}C NMR spectra of compound **SM2**

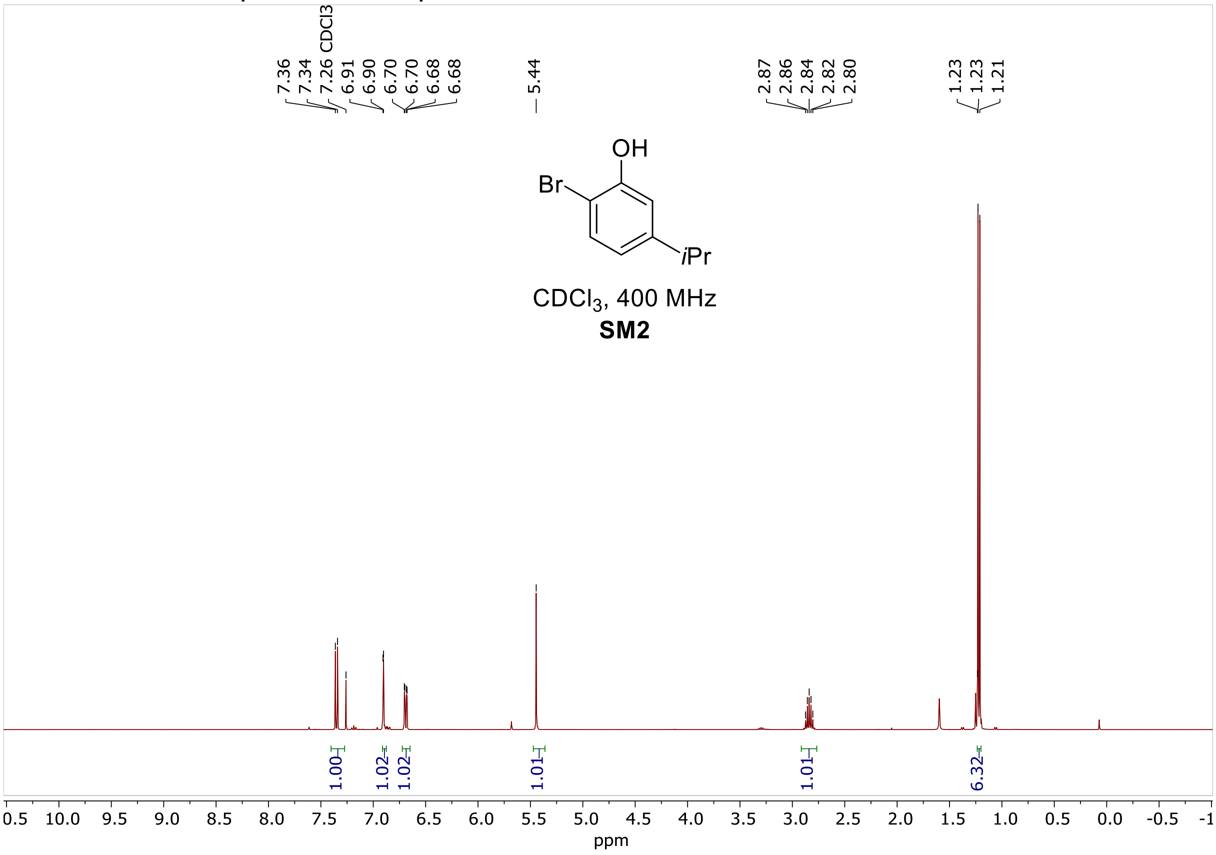

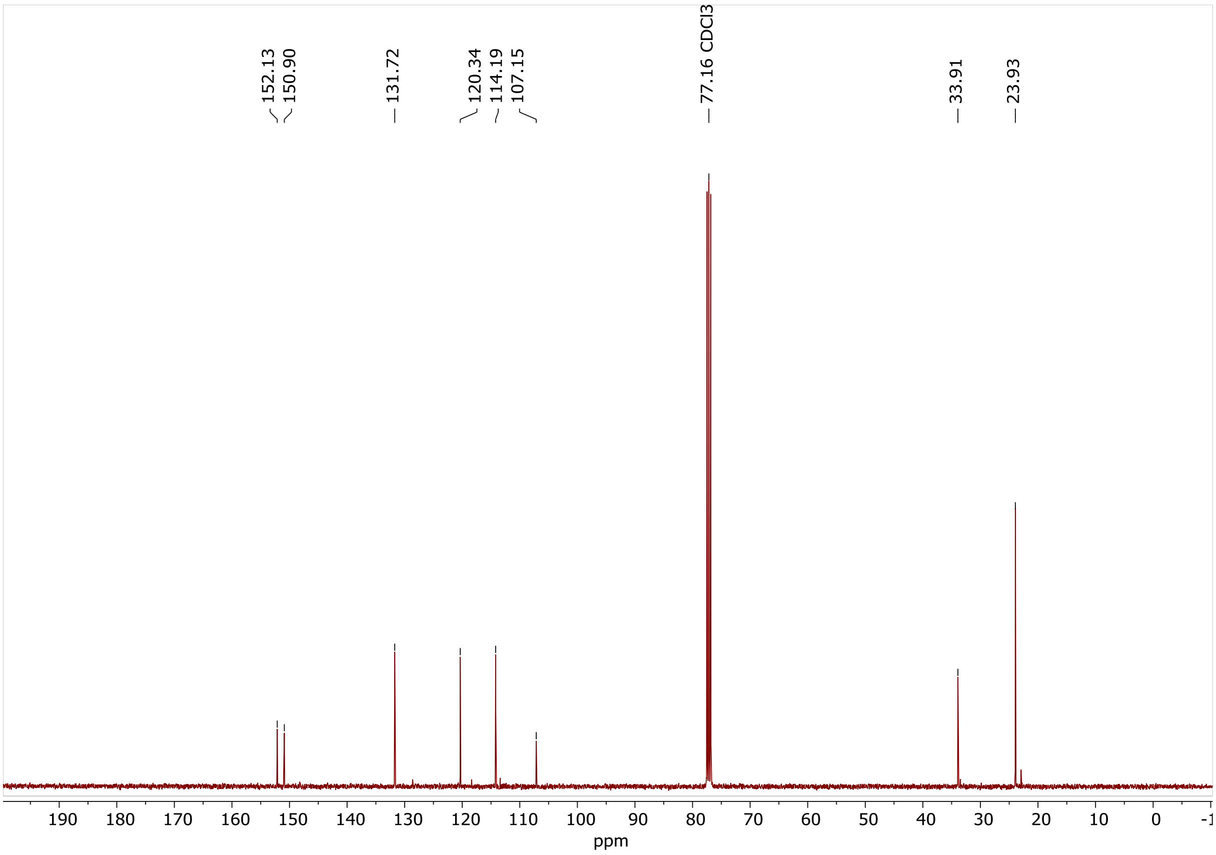

^{1}H and ^{13}C NMR spectra of compound **SM3**

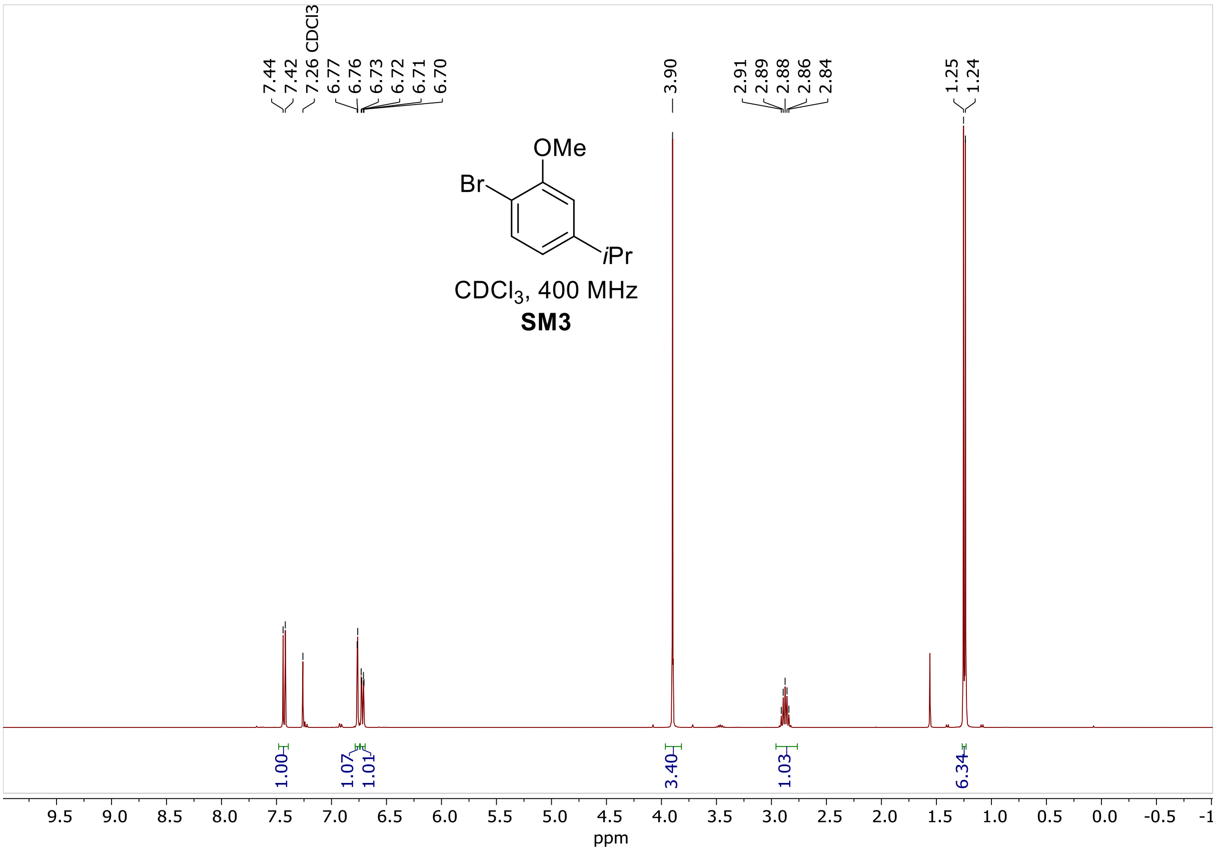

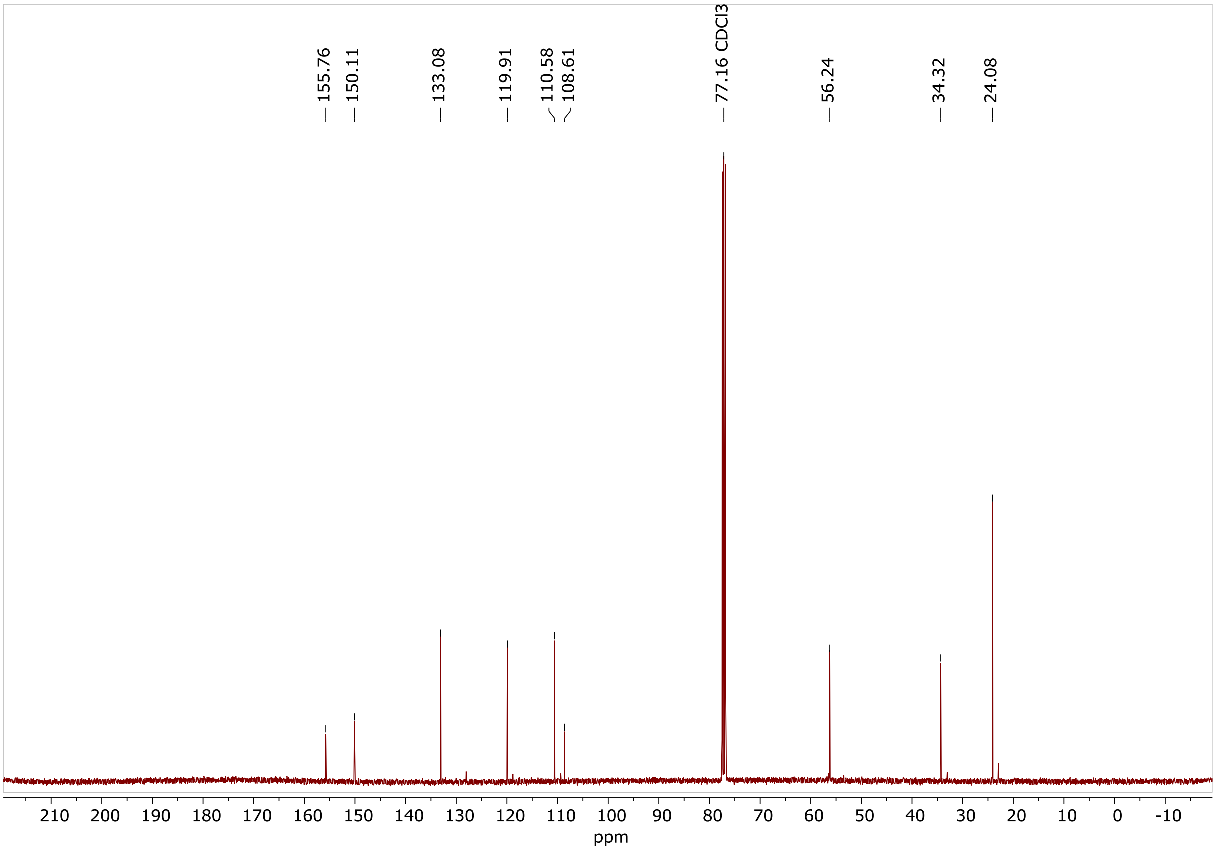

^{1}H and ^{13}C NMR spectra of compound **SM4**

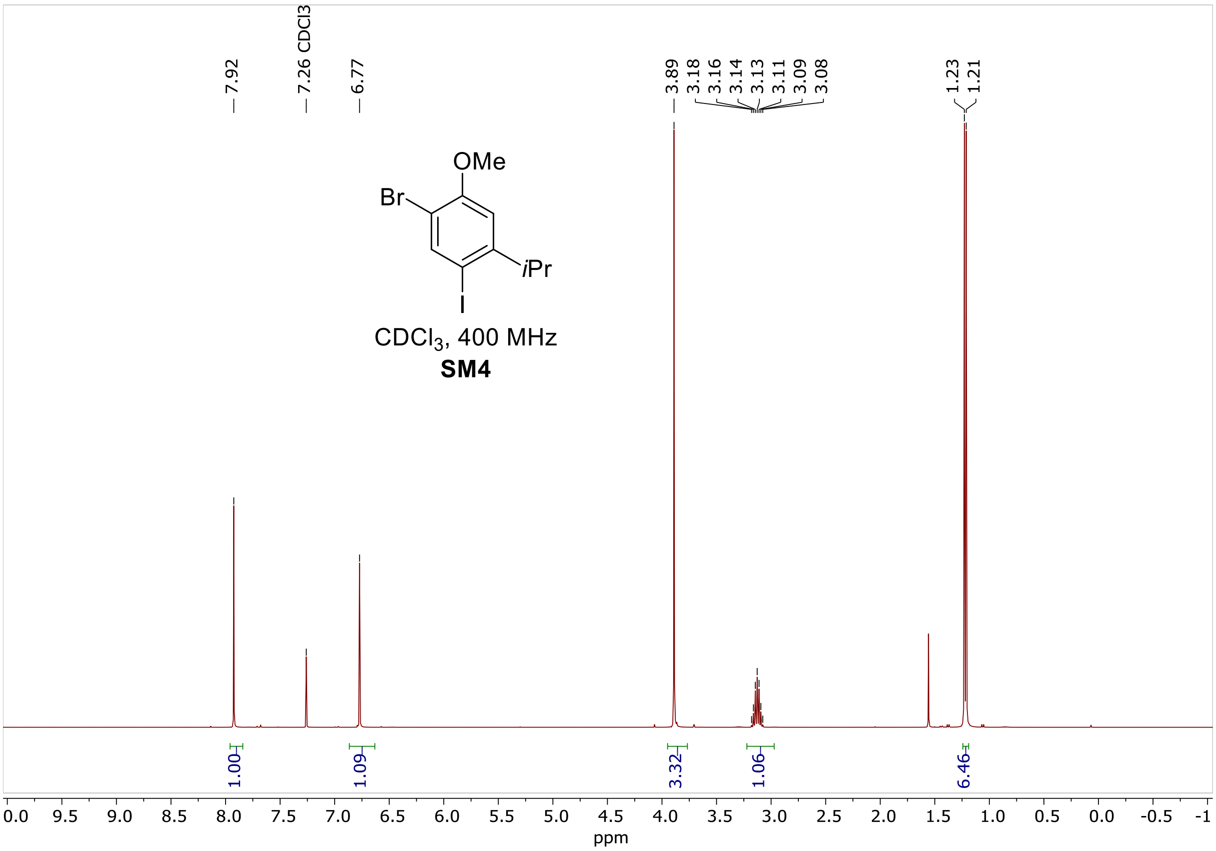

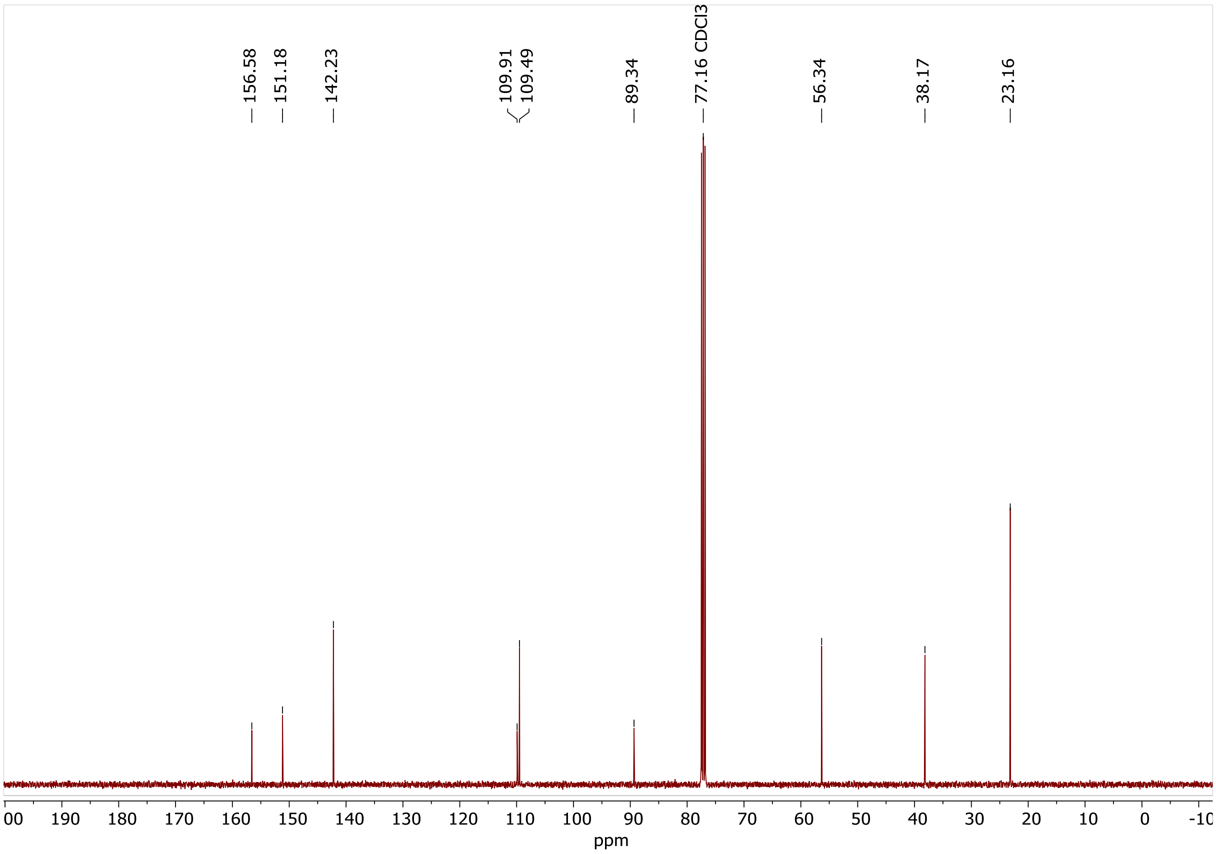

^{1}H and ^{13}C NMR spectra of compound **A**

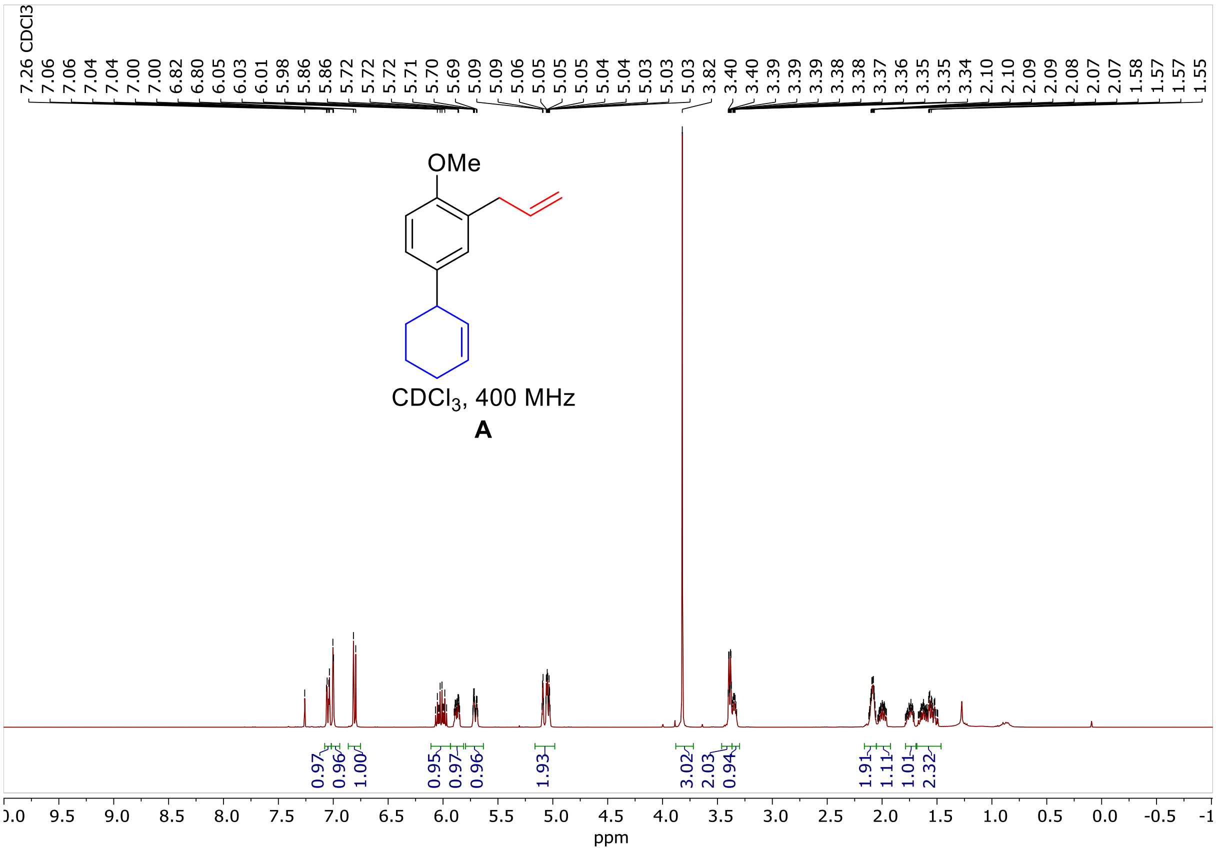

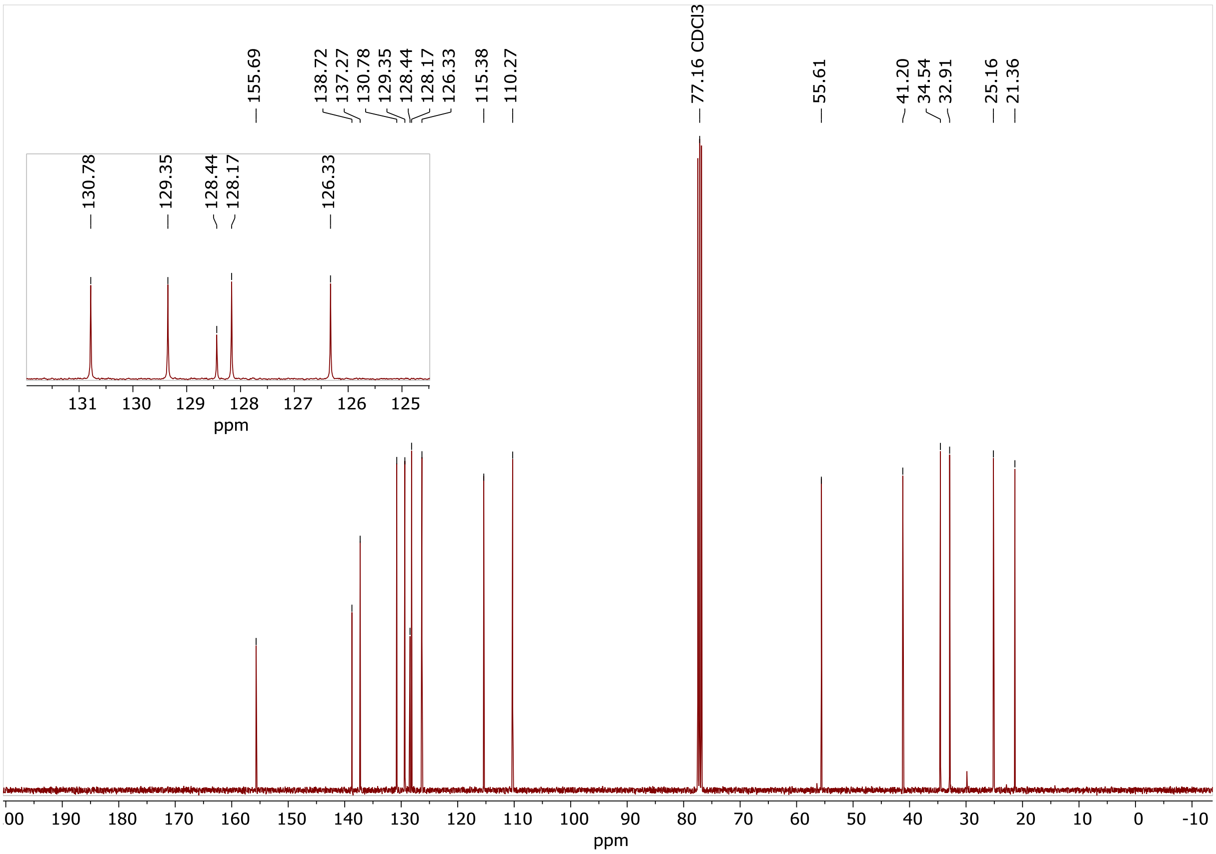

^{1}H and ^{13}C NMR spectra of compound **B**

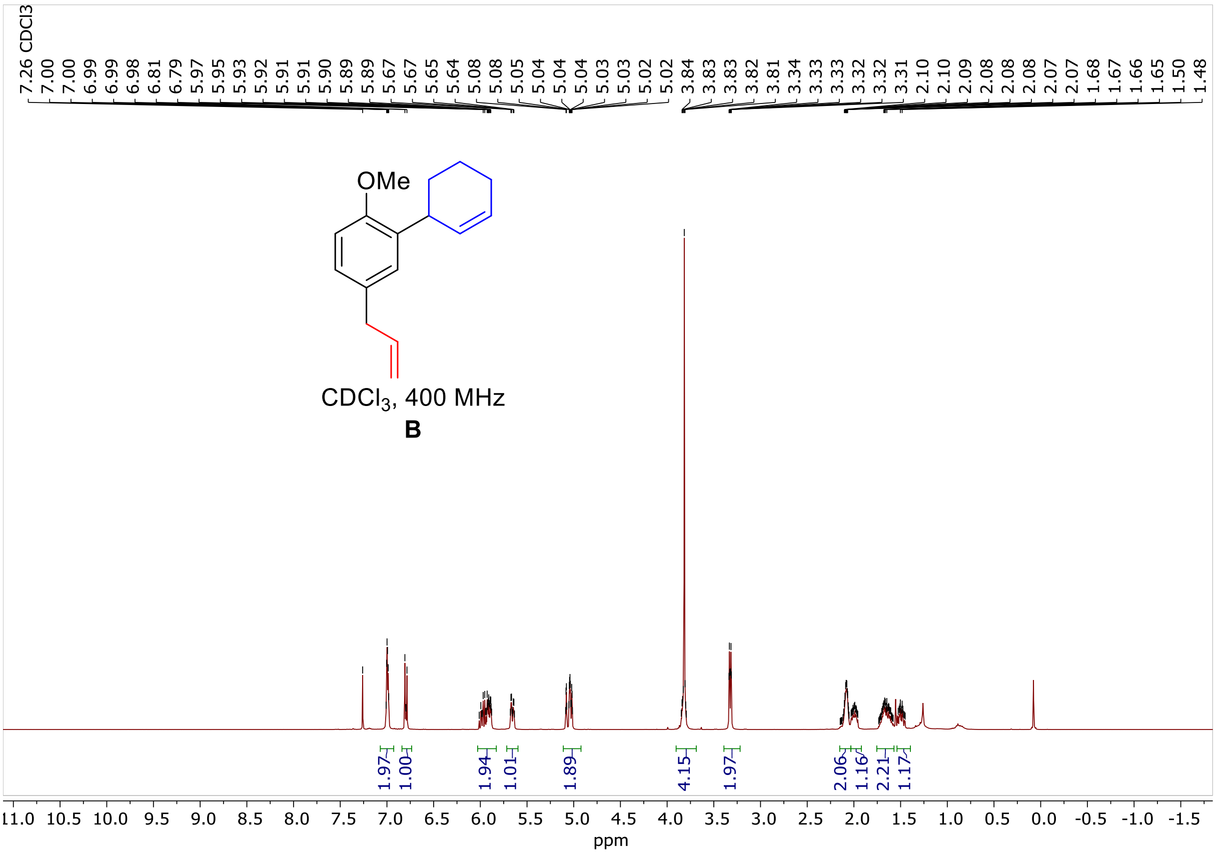

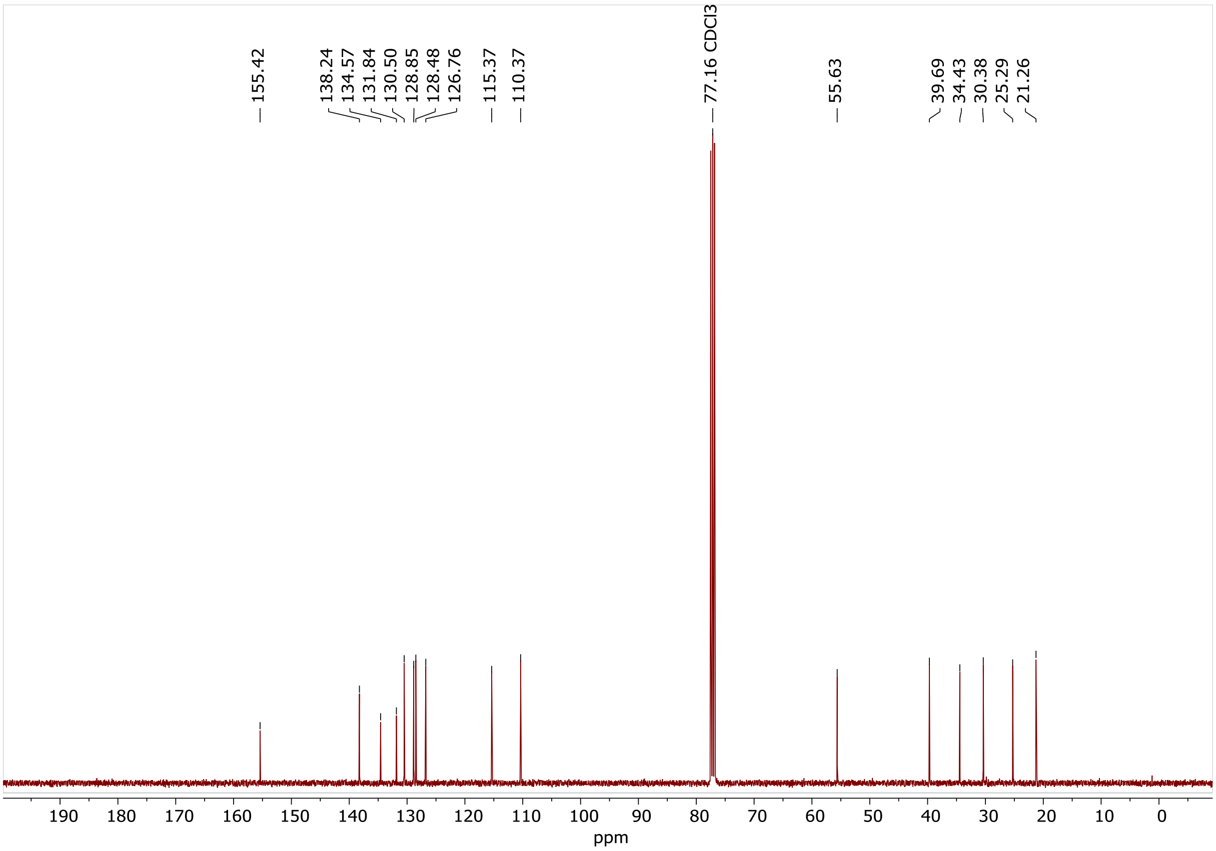

^{1}H and ^{13}C NMR spectra of compound **C**

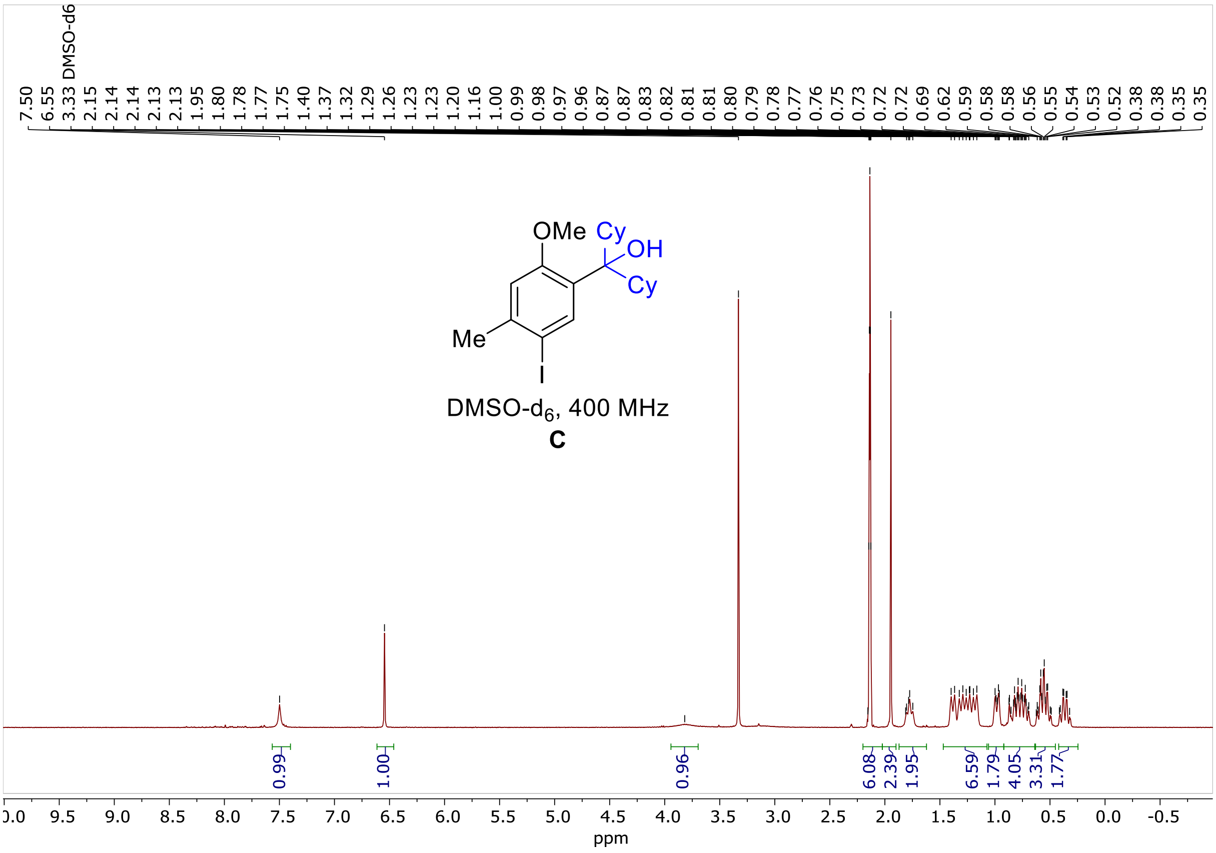

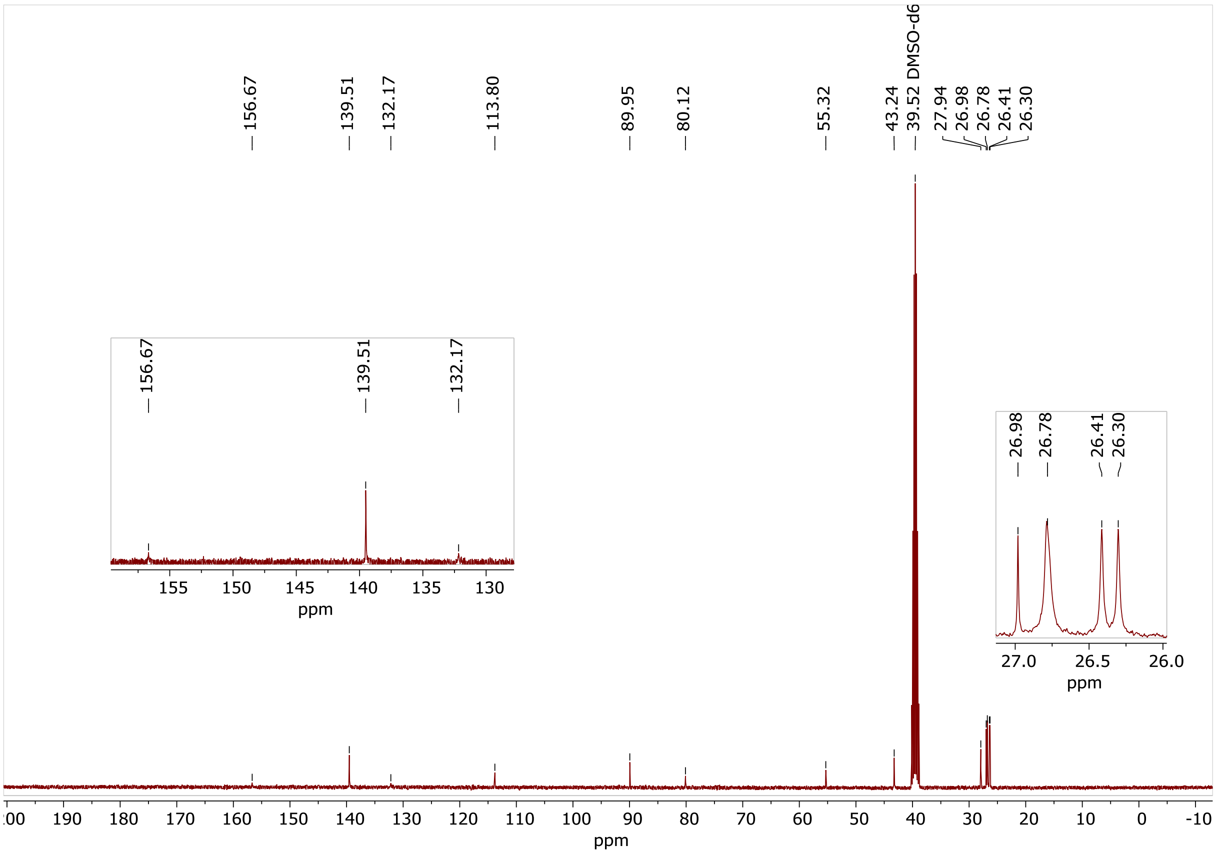

^{1}H, ^{13}C and ^{19}F NMR spectra of compound **D**

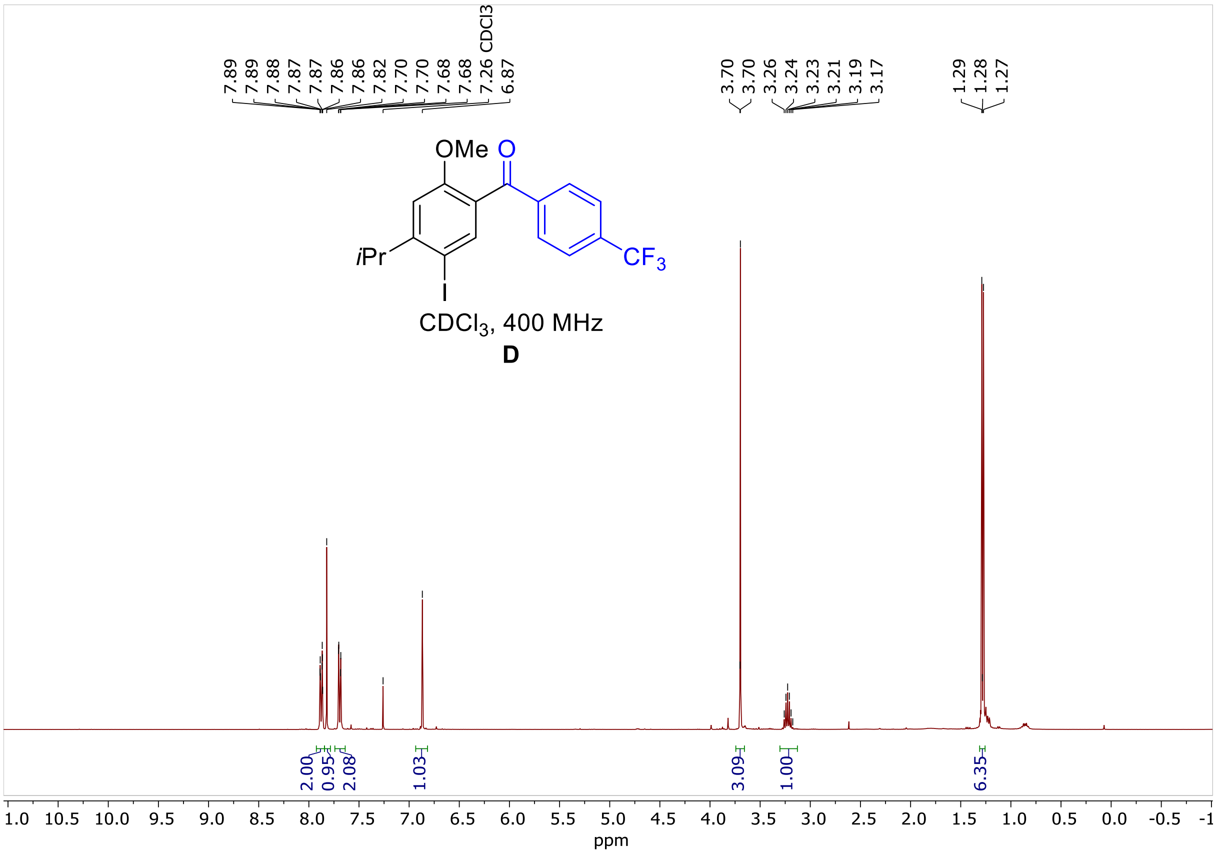

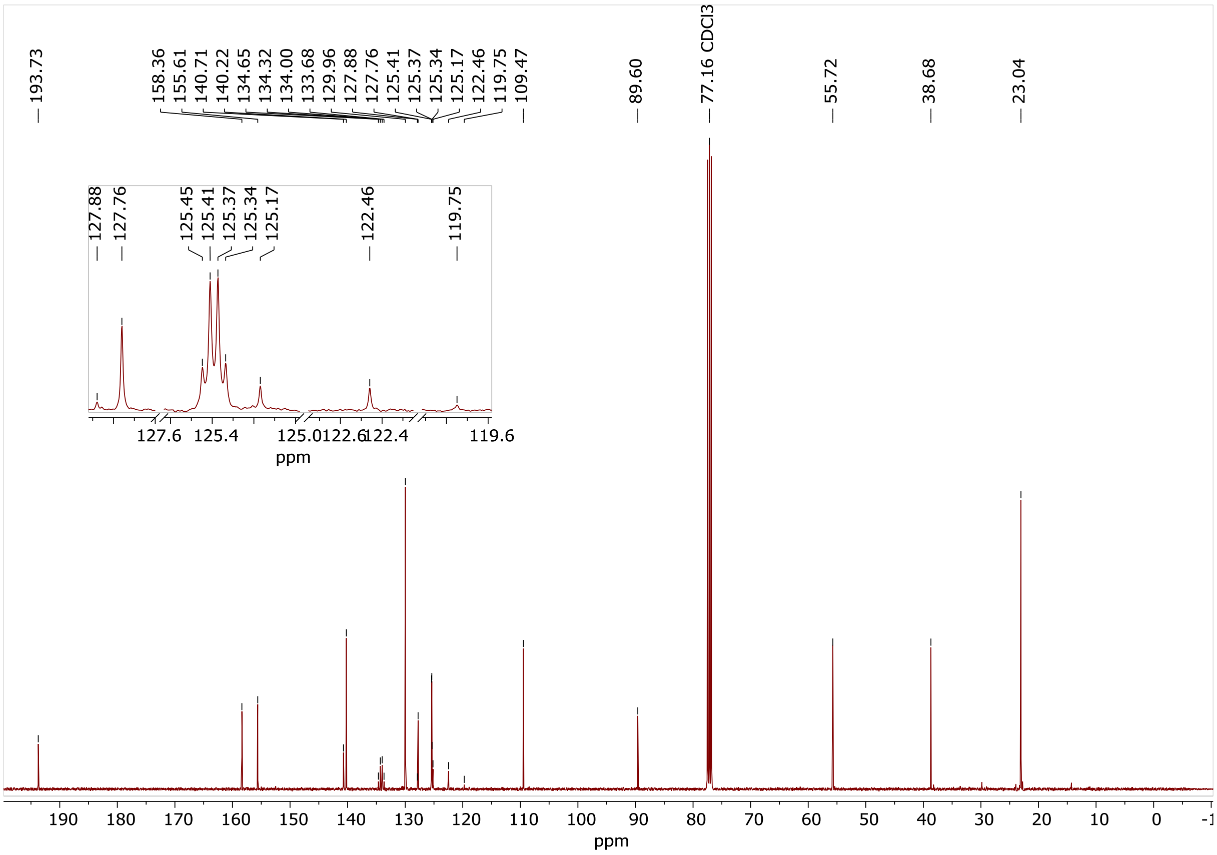

193.73
158.36
155.61
140.71
140.22
134.65
134.32
134.00
133.68
129.96
127.88
127.76
125.41
125.37
125.34
125.17
122.46
119.75
109.47
89.60
77.16 CDCl3
55.72
38.68
23.04
127.88
127.76
125.45
125.41
125.37
125.34
125.17
122.46
119.75
127.6 125.4 125.0122.6122.4 119.6
ppm
190 180 170 160 150 140 130 120 110 100 90 80 70 60 50 40 30 20 10 0
ppm

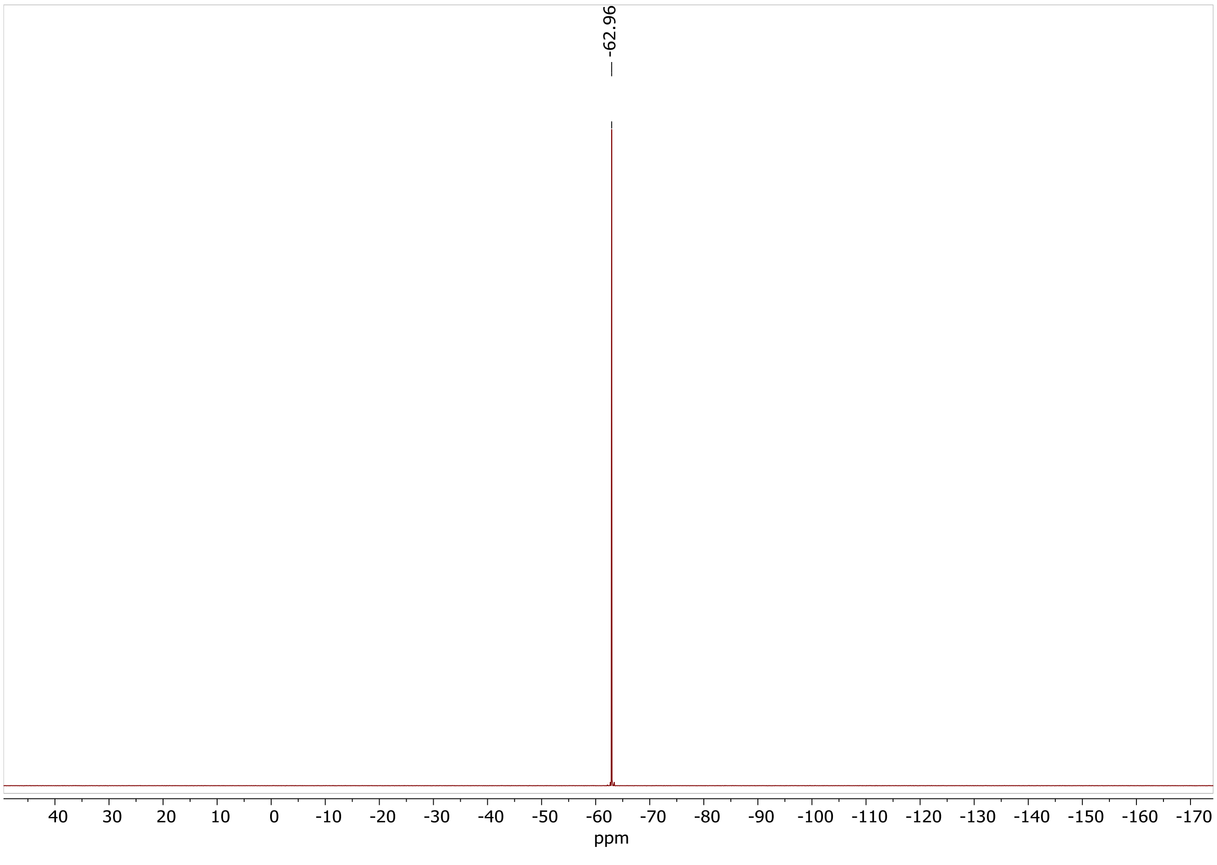

-62.96
40 30 20 10 0 -10 -20 -30 -40 -50 -60 -70 -80 -90 -100 -110 -120 -130 -140 -150 -160 -170
ppm
